# Genome-Wide Analyses of Nkx2-1 Binding to Transcriptional Target Genes Uncover Novel Regulatory Patterns Conserved in Lung Development and Tumors

**DOI:** 10.1371/journal.pone.0029907

**Published:** 2012-01-05

**Authors:** Jean-Bosco Tagne, Sumeet Gupta, Adam C. Gower, Steven S. Shen, Saaket Varma, Meenakshi Lakshminarayanan, Yuxia Cao, Avrum Spira, Thomas L. Volkert, Maria I. Ramirez

**Affiliations:** 1 Pulmonary Center, Boston University School of Medicine, Boston, Massachusetts, United States of America; 2 Center for Microarray Technology, Whitehead Institute for Biomedical Research, Cambridge, Massachusetts, United States of America; 3 Bioinformatics Program, Boston University, Boston, Massachusetts, United States of America; 4 Clinical and Translational Science Institute (CTSI), Boston University School of Medicine, Boston, Massachusetts, United States of America; Northwestern University Feinberg School of Medicine, United States of America

## Abstract

The homeodomain transcription factor Nkx2-1 is essential for normal lung development and homeostasis. In lung tumors, it is considered a lineage survival oncogene and prognostic factor depending on its expression levels. The target genes directly bound by Nkx2-1, that could be the primary effectors of its functions in the different cellular contexts where it is expressed, are mostly unknown. In embryonic day 11.5 (E11.5) mouse lung, epithelial cells expressing Nkx2-1 are predominantly expanding, and in E19.5 prenatal lungs, Nkx2-1-expressing cells are predominantly differentiating in preparation for birth. To evaluate Nkx2-1 regulated networks in these two cell contexts, we analyzed genome-wide binding of Nkx2-1 to DNA regulatory regions by chromatin immunoprecipitation followed by tiling array analysis, and intersected these data to expression data sets. We further determined expression patterns of Nkx2-1 developmental target genes in human lung tumors and correlated their expression levels to that of endogenous NKX2-1. In these studies we uncovered differential Nkx2-1 regulated networks in early and late lung development, and a direct function of Nkx2-1 in regulation of the cell cycle by controlling the expression of proliferation-related genes. New targets, validated in Nkx2-1 shRNA transduced cell lines, include E2f3, Cyclin B1, Cyclin B2, and c-Met. Expression levels of Nkx2-1 direct target genes identified in mouse development significantly correlate or anti-correlate to the levels of endogenous NKX2-1 in a dosage-dependent manner in multiple human lung tumor expression data sets, supporting alternative roles for Nkx2-1 as a transcriptional activator or repressor, and direct regulator of cell cycle progression in development and tumors.

## Introduction

Lineage-specific transcription factors play master roles in development and in maintenance of particular phenotypes in normal tissues and in disease [1]. NK2 homeobox 1 (Nkx2-1, Nkx2.1, Ttf-1, Titf1, T/ebp) is a transcription factor necessary for normal lung, thyroid and brain development [2]. In the lung, once the respiratory epithelial cell fate is established, Nkx2-1 participates in expansion and differentiation of epithelial progenitor cells to form the lung branches; later in development, its expression is restricted to a subset of bronchiolar and alveolar epithelial cells, where it contributes to maintain their normal phenotype. In tumors, variable levels of NKX2-1 expression are detected in 40–50% of non-small cell lung carcinomas (NSCLCs), being higher in lung adenocarcinomas than in squamous cell carcinomas, suggesting that levels of NKX2-1 expression are linked to tumor cell phenotypes [3], [4].

Previous studies showed the physiological importance of normal Nkx2-1 expression levels in development and its dysregulation in disease. In mouse lung, Nkx2-1 absence results in impaired branching morphogenesis, abnormal distal cell differentiation and neonatal death [5]; mutations that prevent Nkx2-1 phosphorylation result in relatively normal morphogenesis but lethal functional defects [6]; conversely, epithelial Nkx2-1 over-expression produces cell hyperplasia, disrupted alveolar septation and emphysema [7]. In human lung, NKX2-1 haplo-insufficiency causes respiratory dysfunction, abnormal airway and alveolar morphogenesis, abnormal surfactant protein expression and infections [2], [8]. In lung cancer, NKX2-1 has been proposed as a positive or negative prognostic factor depending on expression levels [3], [4]. Amplification of the 14q13 locus containing the NKX2-1 gene is observed in only 11–15% of adenocarcinomas [3], [9], [10]; DNA mutations in the open reading frame that may produce a mutated protein or truncations are rarely encountered [3], [10].

The functions elicited by Nkx2-1 expression in different cell contexts are primarily determined by the direct target genes transcriptionally regulated by Nkx2-1. In the lung, a few Nkx2-1 direct target genes have been identified by individual gene promoter analyses including surfactant proteins, secretoglobins, ABCA3 and Nkx2-1 itself [11]. Microarray expression analyses identified genes directly and indirectly regulated by the active phosphorylated form of Nkx2-1 in mice [6] and in human lung fetal cells [12]. The transcriptional program directly controlled by Nkx2-1 in early and late mouse lung development [11] that may explain its primary developmental effects, and the genes regulated by NKX2-1 in human lung cancer are unknown [2], [3].

To address these issues we have analyzed, by chromatin immunoprecipitation-chip and intersection with expression data sets, direct *in vivo* Nkx2-1 transcriptional targets in early vs. late lung development. The genes identified may serve as primary effectors of Nkx2-1 functions in different developmental cell contexts. We determined expression levels of Nkx2-1 target genes identified in development and correlated their expression to the level of NKX2-1 in more than ten public human lung tumor data sets. The regulatory networks discovered clarify the diverse biological roles of Nkx2-1 observed in development, and provide a rationale for the association of NKX2-1 levels and NSCLCs prognosis via its downstream targets.

## Results

### Selection of lung developmental stages for Nkx2-1 target analysis

**Figure 1:**
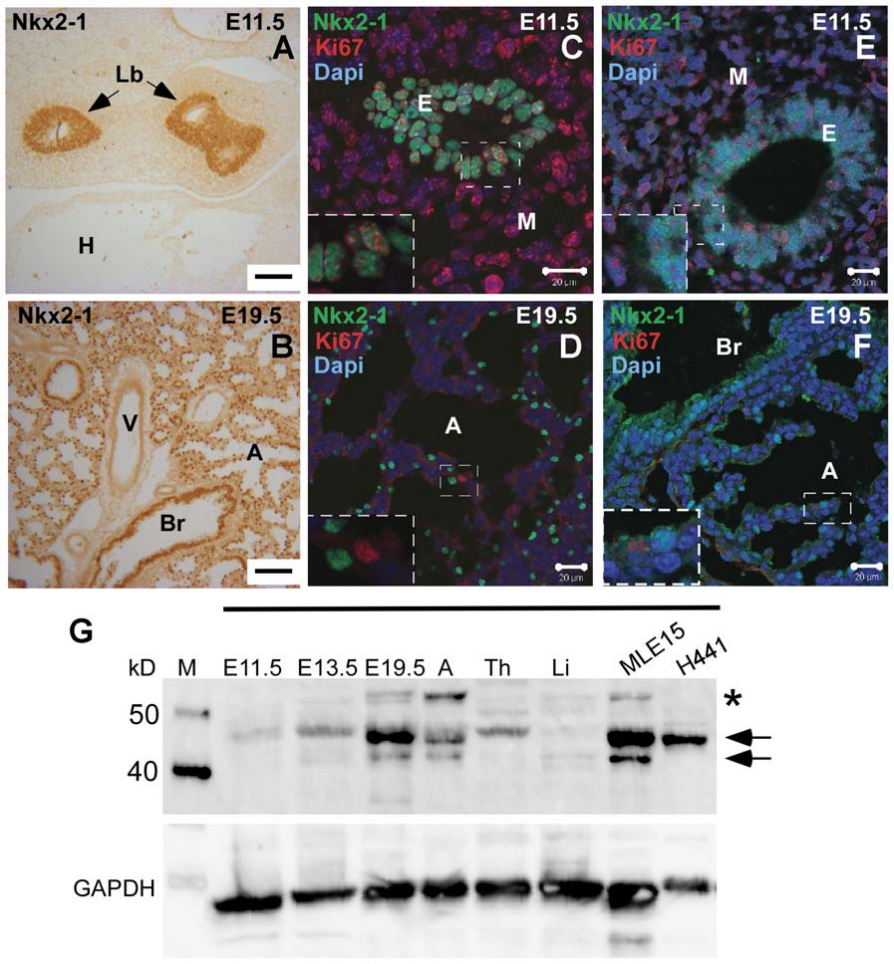
Spatial and temporal pattern of Nkx2-1 protein expression in developing mouse lung. Immunohistochemistry analyses of Nkx2-1 protein expression using the Nkx2-1 antibody (ab76013) in (*A*) E11.5 lung (bar = 100 µm)) and in (*B*) E19.5 lung (bar = 100 µm). Nkx2-1 is expressed in all lung epithelial cells at E11.5, but is restricted to bronchiolar and type II alveolar cells at E19.5. Confocal immunofluorescence co-localization analysis of Nkx2-1 (green) and Ki67 (red) proteins in (*C*) E11.5 (bar = 20 µm) and (*D*) E19.5 embryonic lung (bar = 20 µm) using the Nkx2-1 antibody (ab76013) and in (*E*) E11.5 (bar = 20 µm) and (*F*) E19.5 embryonic lung (bar = 20 µm) using the Nkx2-1 antibody (07-601). Nkx2-1 and Ki67 co-localized in most epithelial nuclei at E11.5 (*C,E* inserts) but are detected in different cells at E19.5 (*D,F* inserts). (*G*) Western blot analysis of Nkx2-1 protein expression using Nkx2-1 rabbit polyclonal antibody (07-601) in developing mouse lung at the indicated time points, and adult lung, thyroid and liver. MLE15 mouse epithelial cells and H441 human lung carcinoma cells were also analyzed. Actin was used as control. Two main bands between 40 and 50 kD are detected with a differential developmental pattern of expression (n = 3) (black arrows). Other minor bands of unknown identity are also detected (*) Lb (lung buds), V (blood vessels), Br (bronchioles), E (epithelium), M (mesenchyme), A (alveolus), green arrow.

To identify genes directly regulated by Nkx2-1 in different cell contexts during lung development we selected two developmental stages based on general differentiation characteristics of the epithelium, and Nkx2-1 expression patterns [13]. We analyzed Nkx2-1 target genes at E11.5, when Nkx2-1 is expressed in most epithelial cells as lung buds start branching (Figures 1A, 1C, and 1E) and at E19.5, when Nkx2-1-expressing cells in the distal lung are undergoing extensive differentiation preparing for the first breath at birth (Figures 1B, 1D and 1F). We observed, by confocal microscopy, using either the rabbit monoclonal Nkx2-1antibody (Figures 1A–D, ab76013; Abcam) or the rabbit polyclonal Nkx2-1 antibody (Figures 1E,F, 07-601; Millipore-Upstate), that Nkx2-1 and the proliferation marker Ki67 [14] co-localize in most epithelial nuclei at E11.5 (Figure 1C, >90%; n = 3, and Figure 1E and inserts). At E19.5, however, only a few cells in the distal lung express Ki67, and those cells do not express Nkx2-1 protein (Figure 1D, 1F and inserts). The monoclonal antibody detects nuclear Nkx2-1 protein expression while the polyclonal antibody detects signal both in the nucleus and cytoplasm. Detection of Nkx2-1 in the cytoplasm has been previously shown by other authors [15], [16], [17]. These results indicate that lung epithelial cells expressing Nkx2-1 in early and late lung development go through different biological processes; it is likely that Nkx2-1 elicits different functions by binding to specific target genes in these developmental stages. Context specific gene regulation controlled by Nkx2-1 has been shown in early and late brain development [13], [18], [19], [20], where Nkx2-1 regulates the specification of interneuron subtype in early proliferating telencephalic progenitors, and later, the migration and sorting of post mitotic neurons to different regions of the brain.

We also characterized, by western blots, Nkx2-1 protein expression patterns at different mouse lung developmental stages, thyroid, liver, and mouse MLE15 and human H441 cell lines. Using the rabbit polyclonal Nkx2-1 antibody (Millipore-Upstate), the same antibody used in ChIP assays, we detected two major bands between 40 and 50 kD in E13.5, E19.5, and adult lung, and in the MLE15 lung epithelial cell line. The higher molecular weight or lower mobility band was the major band detected at E11.5. Its abundance was increased from E13.5 to E19.5 consistent with previous reports [12], [21] (Figure 1G). Only one band corresponding to the lower mobility protein was detected in mouse thyroid and in the H441 human lung carcinoma cell line although their mobility is not identical. Two mouse Nkx2-1 transcripts (2.1 kb and 2.3 kb) differentially expressed during lung development [21] are translated in vitro into two proteins that share a common DNA binding domain but differ in their N-terminal domain. The function and regulation of these isoforms in lung development are unknown, although they differentially activate the surfactant protein C (Sftpc) promoter [12], [21]. Also Nkx2-1 posttranslational modifications such as phosphorylation, acetylation and redox state can modify Nkx2-1 proteins altering their molecular weight, mobility in electrophoresis assays and transcriptional activity [17], [22]; the lack of specific antibodies for each isoform or posttranslational modification precludes evaluating them in the present studies.

### Genome-wide analysis of Nkx2-1 target genes in lung development

Due to the differential pattern of Nkx2-1 protein expression, and proliferation state of expressing cells in E11.5 and E19.5 lung, we chose these developmental time points to identify genome-wide Nkx2-1 binding to target genes *in vivo*. Chromatin immunoprecipitation (ChIP) assays, ligation mediated-PCR (LM-PCR) amplification, and hybridization to promoter tiling arrays were performed to map Nkx2-1 genomic occupancy [23], [24], [25]. Nkx2-1 binding patterns on all mouse chromosomes (Supplementary Figure S1) at E11.5 and E19.5 showed comparable binding levels, despite the different cell context compared. The complete data set is available at GEO (GSE23043).

Bioinformatic analysis of the ChIP-chip data allowed us identifying unique interactions between Nkx2-1 and genomic DNA regions at high confidence. Nkx2-1 binds to ∼8,000 features/oligonucleotides on the promoter tiling arrays at E11.5, and ∼9,000 at E19.5 (p<0.001). Multiple features correspond to regulatory regions on the same gene. Comparison of Nkx2-1 binding features revealed that 25% of the targets were unique to E11.5 (3010 features), 32% were unique to E19.5 (3934 features) and 43% (5170 features) were common to both developmental stages. The Nkx2-1 bound features/oligonucleotides correspond to regulatory regions of >1300 independent genes at E11.5 and at E19.5 of the ∼17,000 represented in the array (Supplementary Table S1a and S1b). Comparison of common bound genes using the ∼1300 highest bound single probes representing individual genes showed that only 85 of the ∼1300 genes (6.5%) were common between E11.5 and E19.5. These findings suggest that most of the common probes found at both time points have significant but low binding signal. So most of the genes used in further studies were either highly bound at E11.5 or at E19.5.

**Figure 2:**
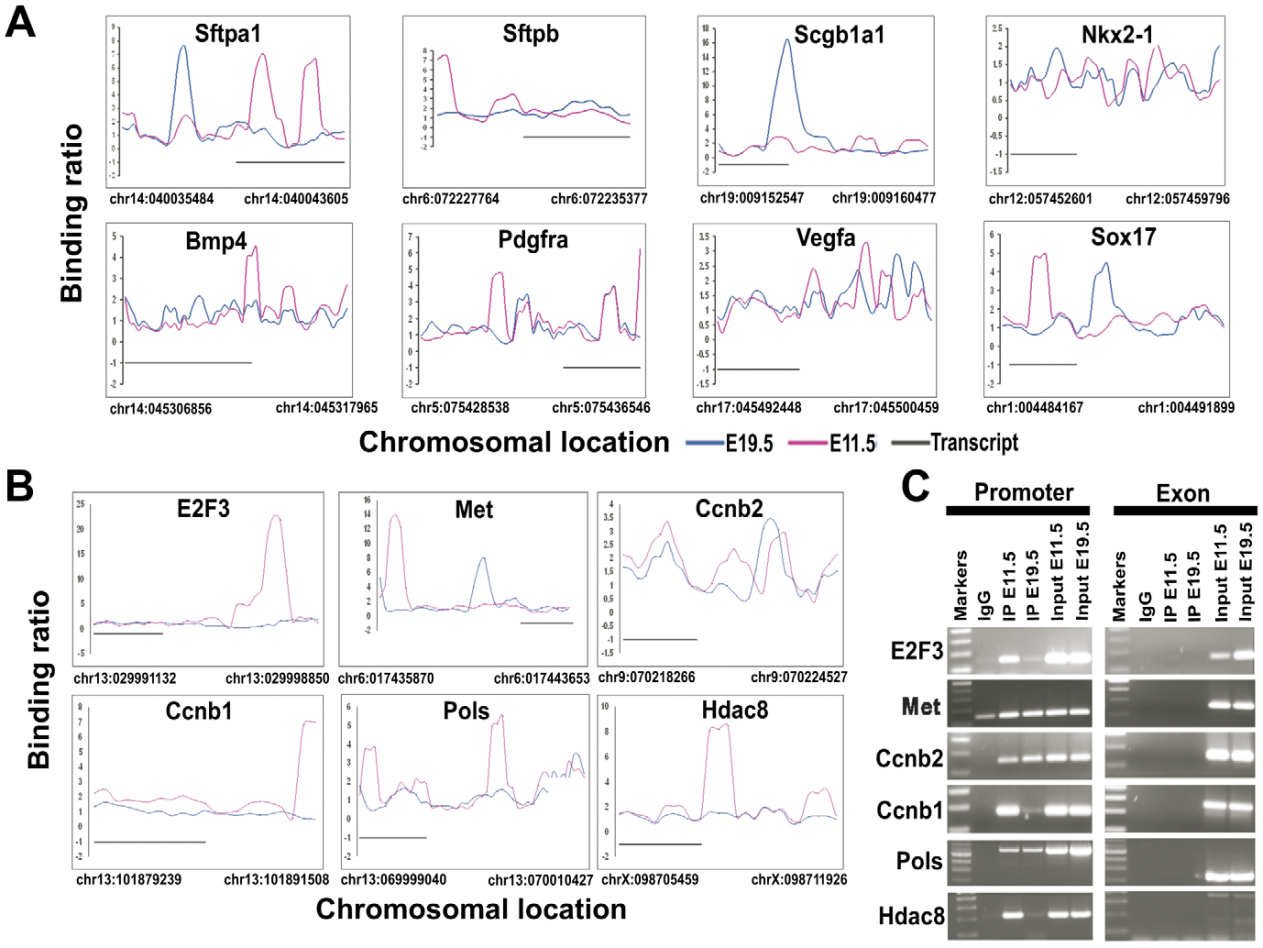
Nkx2-1 binding patterns to selected target genes in lung development. (*A*) Binding patterns of Nkx2-1 to known lung target genes. Binding ratios (IP/Input) are shown at different chromosomal locations. Pink (binding in E11.5 lung), blue (binding in E19.5 lung). Transcripts are indicated by a black line. (*B*) Binding patterns of Nkx2-1 to newly identified targets involved in cell proliferation. (*C*) Chromatin immunoprecipitation-PCR validation of Nkx2-1 binding to proliferation-related target genes. Nkx2-1 IP DNA from E11.5 and E19.5 lungs, Input and IgG immunoprecipated control were used in PCR analyses. Oligonucleotides in the promoter region were used to analyze binding of Nkx2-1, and at exon regions of the same gene as control, (n = 2).

We found Nkx2-1 protein bound to its own promoter, the promoters of surfactant proteins-A and –B (Sftpa, Sftpb), secretoglobin 1a1 (Scgb1a1) consistent with previous reports [2] (Figure 2A) and other targets (Figure 2B). By intersecting the ChIP-chip data and microarray expression data sets of mesenchyme-free freshly isolated E11.5 lung epithelium [26] we identified Nkx2-1 target genes expressed in E11.5 lung. Among 3154 genes called ‘present’ in E11.5 lung epithelium, 374 genes are Nkx2-1 targets (p≤0.001) (Supplementary Table S2). Of the 2611 genes called ‘present’ in a public E18 lung data set [27], 183 genes are Nkx2-1 targets at E19.5 (≤0.001) (Supplementary Table S2). Furthermore, ∼50% of the 95 top genes regulated by Nkx2-1 in human fetal epithelial cells are direct targets of Nkx2-1 (Supplementary Table S3), validating our findings.

### Validation of selected ChIP-chip targets by ChIP and gene specific PCR

We confirmed *in vivo* binding of Nkx2-1 to selected ChIP-chip predicted targets involved in cell proliferation by gene-specific ChIP-PCR in E11.5 and E19.5 lung (Figure 2C) using the same antibody than in the ChIP-chip analyses; we validated them as bona fide direct targets of Nkx2-1. Nkx2-1 regulated genes in other organs such as thyroglobulin in thyroid or Lhx6 and Olig3 in brain, not detected in lung development, are highly bound by Nkx2-1 in the lung (Supplementary Figure S2), indicating that binding does not imply functional activation of transcription; other mechanisms, such as chromatin remodeling or DNA methylation, may silence those genes in the lung. Genes expressed later in lung development are already bound by Nkx2-1 at E11.5. For example, Sftpa, not expressed at detectable levels in E11.5 lung, is bound by Nkx2-1 in regions 5′ and 3′ to the transcription initiation site (Figure 2A). At E19.5, increased Nkx2-1 binding is detected on the Sftpa promoter region correlating with higher transcription as previously described by classical promoter analyses [28].

### Biological processes and pathways targeted by Nkx2-1

To identify novel genes and pathways that may be effectors of Nkx2-1 functions during lung development, we performed Gene Ontology analyses using EASE [29] and ∼1300 individual genes bound by Nkx2-1 (log_2_ binding ratio >0.75; p≤0.001) at E11.5 or at E19.5. These analyses revealed significant enrichment in many biological processes at each time point (p<0.05) (Supplementary Table S4). Selected genes in the top biological processes at each time point are shown in Table 1. In particular, ‘positive regulation of cell proliferation’ is significantly enriched at E11.5 (p = 0.0038) but not at E19.5 (p = 0.9824), while ‘ion transport’ is enriched at E19.5 (p = 0.0001) but not at E11.5 (p = 0.1976), supporting a differential role for Nkx2-1 at each developmental stage [30]. We concentrated our studies in Nkx2-1 proliferation-related target genes because of their link to development and cancer. Although Nkx2-1 has been shown to control cell proliferation [7], [10], direct regulation of proliferation-related genes by Nkx2-1 has not been reported. Proliferation-related Nkx2-1-target genes expressed in early lung epithelium and/or human lung tumors are shown in Table 2. Overrepresented pathway analysis performed by Ingenuity Pathway Analysis at E11.5 and E19.5 (Supplementary Table S5) identified common Nkx2-1 targeted pathways at both stages, such as ‘mechanisms of cancer’ and ‘HGF signaling’ (Supplementary Table S6). Other pathways were uniquely overrepresented at E11.5, including ‘RAR signaling’ and ‘TR/RXR activation’, and at E19.5, including ‘renin-angiotensin signaling’. These Nkx2-1 regulated pathways have been previously linked to lung development and tumorigenesis [31], [32], [33].

**Table 1:** Selected genes in non-redundant over-represented biological processes among genes bound by Nkx2-1.

*E11.5*		
*Term*	*PValue*	*Genes*
Positive regulation of cell proliferation	3.77E-03	Odc1, Il3, Cdca7l, Prkcq, Notch1, Myc, Il12a, Recql4, Lig4, Nr2e1, Cnbp, Bcl2, Hcls1, Igf1, E2f3, St8sia1, Lamb1-1, Il4, Otp, Nodal, Il18, Cd3e, Ube2c, Edg3, Sp6,
Ras protein signal transduction	5.56E-03	Arhgef11, Arf6, Tbc1d20, D10Ertd610e, Fgd3, Rhob, Rasgrf2, Psd4, Arhgdia, D10Bwg1379e, Hrasls, Psd3, Fgd2, Nisch, Cdc42ep4, Hras1, Grit, Rasgrp3, Tbc1d10a, Pscd3, Rfxank, Abr, Plce1, Tbc1d2b,

*E19.5*		
*Term*	*PValue*	*Genes*
Ion transport	9.77E-05	C1qtnf3, Steap4, Bat3, Atp6v1g3, Kcne3, Slc41a2, Cacnb4, Col1a1, Kcnc2, Grid1, Itpr2, Pkd1, Slc39a11, Clca5, Atp6ap1, Kcnn2, Steap1, Slc6a8, Kcnv1, Kcnj8, Slco2b1, Kcnj16, Kcne2, Slc4a4, Kcnk9, Slc12a5, Slc34a1, Slc38a2, Tigd4, C1qtnf7, Cybb, Accn2, Gabra3, Slc13a1, Col8a1, Kctd13, Slc9a7, Slc11a1, Kcnh5, Kcnmb4, Fxyd3, Atp13a1, Rcvrn, Myb, Clcn1, Chrna1, P2rx1, Slc5a3, Kcnj15, Slc22a6, Sfxn4, P2rx7, Col6a2, Slc26a1, Adrb2, Stim1, Slc34a2, C1qa, Slc22a4, Ank, Rhbg, Kcnc4, Gm414, Fxyd2, Col17a1, Chrna7, Slc17a1, Clic5, Atp1a1, Steap3, Slc41a1, Slc12a8, Slc30a6, Col4a6, Kcne4, Kctd4, Sftpa1, C1qtnf5, Slc39a14, Atp6ap2, Atp2b3,
Response to wounding	4.67E-03	Saa3, Thbs1, Daf2, Tlr11, Tollip, Il17d, F7, AI182371, Cxcl10, C8a, F9, Ccl22, Ccl5, C4bp, Nfam1, Stab1, C1qa, Sod1, C8b, Fga, Ly86, Abhd2, S100b, Chrna7, F2rl3, Alox5, Chi3l3, Cd28, Serpinf2, Casp3, Pap, Il6, Tac1, Cd24a, F10,

**Table 2:** Cell proliferation related genes bound by Nkx2-1 and expressed in lung development and tumors.

*GeneName*	*SystematicName*	*Description*	*Signal*	*p value*	*Lung Expression* *E11.5 (Lu et al 2005)*	*E14.5 (GenePaint)*	*Tumors*	*PMID*
Ube2c	chr2:164463527-164463586	INSIDE	1.904	1.37E-20	P	E/M	lung tumors	15208666
Fkbp1a	chr2:151229364-151229423	PROMOTER	1.842	3.08E-22	P	**E**	metastasis	11517338
Erbb3	chr10:127991654-127991713	INSIDE	1.649	2.85E-09	P	**E**	NSCLC	12483526
Tff1	chr17:030889902-030889950	INSIDE	1.536	1.85E-13	P	**e**	adenocarcinoma	7917539
Map3k11	chr19:005690697-005690741	INSIDE	1.503	1.32E-18	P	e/M	solid tumors (lung)	18636107
Pik3ca	chr3:032629205-032629264	INSIDE	1.412	2.30E-18	A	e	lung tumors	19010912
Anln	chr9:022081918-022081977	DOWNSTREAM	1.330	2.67E-21	P	E/M	SCC	16357138
Rhob	chr12:008528273-008528332	PROMOTER	1.311	5.97E-21	P	E/M	lung tumors	15102679
Ncaph	chr2:126823595-126823654	INSIDE	1.301	3.06E-11	P	NA		
E2F3	chr13:029996944-029997003	PROMOTER	1.270	2.07E-07	A	**E**	NSCLC/sclc	16938365
Ccnb1	chr13:101891449-101891508	PROMOTER	1.253	7.30E-13	P	E/m	NSCLC	12883711
Fgfr3	chr5:034037899-034037943	PROMOTER	1.215	1.20E-19	P	E/m	NSCLC	17949785
Sox4	chr13:028964510-028964569	PROMOTER	1.209	2.98E-05	P	**E**	lung tumors	19153074
Odc1	chr12:017565992-017566050	PROMOTER	1.145	6.20E-07	P	E/M	NSCLC	20199977
Cdca7l	chr12:118283635-118283694	PROMOTER	1.143	1.64E-14	P	**E**		
Asns	chr6:007647542-007647601	PROMOTER	1.093	1.58E-04	P	**E**		
Racgap1	chr15:099483295-099483350	PROMOTER	1.090	2.00E-19	P	E/M		
Hras1	chr7:141042582-141042630	DOWNSTREAM	1.049	3.30E-08	P	**E**	NSCLC	10430091
Ptprf	chr4:117777704-117777758	INSIDE	1.026	7.95E-14	P	NA		
Msh2	chr17:087582981-087583033	INSIDE	0.997	4.92E-18	P	**E**	NSCLC	18646042
Pard3	chr8:130324465-130324524	INSIDE	0.976	6.74E-05	P	E/M	Tumors	20215515
Erh	chr12:081561122-081561181	INSIDE	0.973	2.04E-13	P	E/M	Breast cancer	18500978
Nek6	chr2:038335233-038335289	INSIDE	0.968	1.30E-04	P	**E**	lung tumors	20407017
Cdkn2c	chr4:109162876-109162934	INSIDE	0.946	2.11E-10	P	ND	mouse lung tumors	17409423
Prdx1	chr4:116184956-116185015	INSIDE	0.930	9.83E-05	P	E/m	NSCLC	18413821
Mfge8	chr7:079028025-079028075	PROMOTER	0.926	6.20E-06	P	E/M		
Furin	chr7:080274016-080274066	INSIDE	0.916	5.15E-13	P	E/m		
Bcl2	chr1:108538280-108538339	INSIDE	0.915	6.31E-05	P	E/M	NSCLC	19240654
Cnbp	chr6:087818676-087818735	PROMOTER	0.902	1.68E-11	P	E/M	NSCLC	17327219
Notch1	chr2:026326836-026326885	PROMOTER	0.902	2.47E-04	P	**E**	NSCLCs	20007775
Pawr	chr10:107733486-107733545	PROMOTER	0.894	3.17E-10	P	**E**	lung tumors	18650932
Hmox1	chr8:077991770-077991828	INSIDE	0.892	1.17E-12	P	ND	adenocarcinoma	15688187
Igf2	chr7:142465948-142466003	INSIDE	0.892	1.74E-13	P	**E**	adenocarcinoma	11536368
Mmp14	chr14:053385520-053385574	PROMOTER	0.884	1.63E-08	P	E/M	SCC	15036884
Ccnb2	chr9:070223337-070223396	PROMOTER	0.812	1.32E-15	P	e/m	NSCLC	15331390

E, high expression in lung epithelium; e, low expression, M, high expression in lung mesenchyme; m, low expression; ND, non-detected;

NA, non-available; P, present; A, absent.

### Reduced Nkx2-1 expression affects cell cycle progression and transcription of target genes

**Figure 3:**
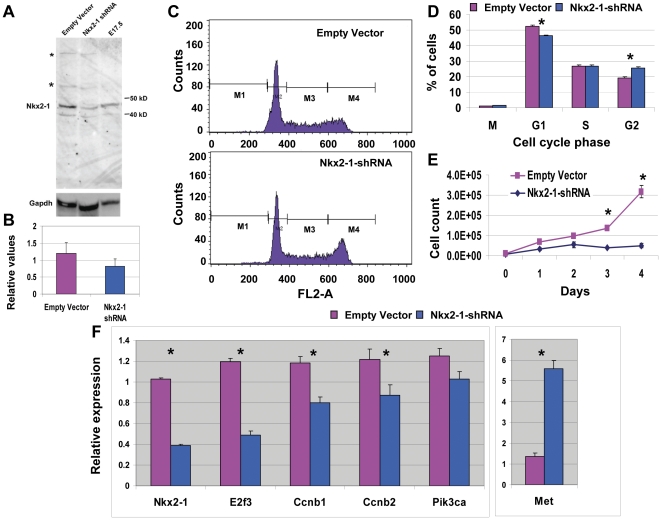
Downregulation of Nkx2-1 affects expression of proliferation-related genes in mouse lung epithelial cells. (*A*) Western blot analysis of Nkx2-1 downregulation by shRNA in MLE15 cells using the Nkx2-1 antibody 07-601 (Millipore-Upstate). Two bands between 40–50 kD are detected in mouse MLE15 cells and E17.5 lung. Densitometry analyses show a significant downregulation of Nkx2-1 major bands in shRNA transduced cells. Nkx2-1 downregulation by shRNA also reduces the level of minor bands of higher molecular weight (*, asterisks) (*B*) Representative flow cytometry analyses of DNA content and cell cycle progression in the same cells; n = 3. (*C*) Relative number of cells in each stage of the cell cycle. (D) Cell count analysis of MLE15 cells transduced with empty vector or Nkx2-1shRNA. 5000 cells were plated and grown in standard conditions for up to 4 days. Attached cells were trypzinized and counted; n = 6 (E) Real time RT-PCR analyses of Nkx2-1 mRNA expression levels in Nkx2-1 shRNA or empty vector transduced cells; n = 3; similar analyses were performed for selected targets; n = 6. Error bars represent s.e.m.; (*) p≤0.05.

To evaluate whether altered levels of Nkx2-1 expression can affect target gene transcription, we performed short hairpin RNA (shRNA)-mediated Nkx2-1 knockdown in the mouse lung epithelial cell line MLE-15. These cells express both forms of the Nkx2-1 protein identified in development (Figure 3A). Nkx2-1 message was reduced by 60% and average expression of both Nkx2-1 protein forms was reduced by 40% (Figures 3A and D). Nkx2-1s shRNA reduces expression of the two main bands between 40–50 kD but also of a faint band of higher molecular weight. These bands may represent modifications of Nkx2-1, although we have not confirmed their composition. Reduction of Nkx2-1 delayed cell cycle progression by halting cells in G2/M phase (Figures 3B and C). The moderate reduction in Nkx2-1 levels has a measurable impact on cell growth and target gene regulation. Cell count analyses performed at 24 h intervals for 4 days show a significant difference in total number of cells after 3 days in culture (Figure 3D). By qRT-PCR we determined downregulation of E2f3, Ccnb1, Ccnb2, and Pik3ca by reduction of Nkx2-1 (Figure 3E). Block of cell cycle in G2/S phase observed by down-regulation of Nkx2-1 in cell lines may be mediated by down-regulation of Cnnb1 and Cnnb2 which participate in transition of cells into the synthesis phase. c-Met, conversely, was highly upregulated, supporting a role of Nkx2-1 as a transcriptional repressor. Alternatively, indirect mechanisms controlled by Nkx2-1, besides Nkx2-1 binding to c-Met promoter, could control transcription of this gene.

### Correlation of NKX2-1 and developmental target gene expression levels in NSCLC

**Figure 4:**
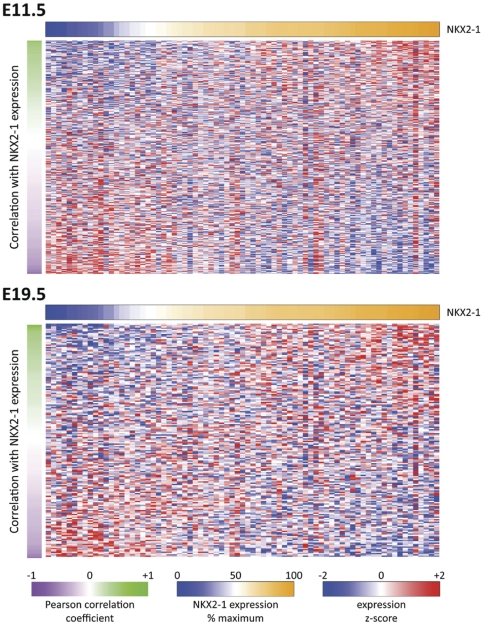
NKX2-1 levels in human lung tumors significantly correlate with expression of developmental Nkx2-1 target genes. Heatmaps of human lung tumor genes identified in GSE12667 database showing gene expression levels of the human homologues of Nkx2-1 target genes identified in mouse lung development at E11.5 (upper panel) and E19.5 (lower panel); genes are organized according to their Pearson correlation value (y axis) to relative NKX2-1 expression level (x axis).

Lung development and cancer have been linked by comparing embryonic and tumor gene expression profiles [34], [35]. Also, developmental gene programs active in human lung tumors have been explored as predictors of patient's survival [35], [36]. The effector genes responsible for these associations, however, are largely unknown. We evaluated expression of Nkx2-1 target genes identified in mouse development in human lung tumors, and their correlation with endogenous NKX2-1 levels (Figures 4 and S4). Sixteen human lung tumor data sets, available in GEO or the literature, including gene expression levels in lung adenocarcinomas and squamous cell carcinomas, were analyzed (Table 3). Human homologues corresponding to mouse Nkx2-1 target genes at E11.5 (350 genes) and at E19.5 (183 genes) were used in GSEA analyses. The expression of E11.5 developmental target genes significantly correlated or anti-correlated to NKX2-1 level in 10 out 16 data sets (p<0.05). The expression of E19.5 developmental target genes significantly correlated or anti- correlated to NKX2-1 level in 9 out 16 data sets (p<0.05). The anti-correlation of many Nkx2-1 target genes with NKX2-1 expression in tumors (Figures 4 and S4) and of c-Met with Nkx2-1 levels shown in MLE15 cells supports a role of Nkx2-1 as a transcriptional repressor.

**Table 3:** Human lung tumor data sets used in the correlation and GSEA studies.

				*GSEA p values*
*Set*	*PMID*	*Number of tumors*	*Cell type*	E11.5/E11.5*	E19.5/E18*
GSE12667	18948947	75	Adenocarcinoma	**0.017**	**0.003**
GSE10245	18486272	58	All	**<0.001**	**<0.001**
		40	Adenocarcinoma	0.432	**0.044**
		18	Squamous	**0.034**	0.062
GSE10799	19208797	16	Adenocarcinoma	0.228	0.222
GSE10445	19176396	72	Adenocarcinoma	**0.024**	**0.005**
Bild 2006	16273092	111	All	**<0.001**	**<0.001**
		58	Adenocarcinoma	**<0.001**	**<0.001**
		53	Squamous	**<0.001**	**<0.001**
GSE7670	17540040	27	Adenocarcinoma	0.124	0.821
Shedden DFCI 1	18641660	52	Adenocarcinoma	**<0.001**	0.118
Shedden DFCI 2		30	Adenocarcinoma	0.772	0.155
Shedden Moffitt		79	Adenocarcinoma	**0.02**	0.118
Shedden UMich		178	Adenocarcinoma	**0.009**	**0.001**
Shedden MSKCC		104	Adenocarcinoma	**0.043**	0.18
Lu U133A	17194181	18	Squamous	**0.003**	**0.005**
Bhattacharjee 2001	11707567	254	All	**0.001**	0.054
		197	Adeno and Squamous only	**<0.001**	**<0.001**
		176	Adenocarcinoma	**0.009**	**0.035**
		21	Squamous	0.063	**0.002**
Borczuk 2004	15087295	74	All	0.261	**0.036**
		17	Adeno and Squamous only	0.592	0.183
		10	Adenocarcinoma	0.457	0.626
		7	Squamous	0.537	0.704
Lu U95Av2	17194181	36	All	0.771	0.125
		32	Adeno and Squamous only	0.797	0.143
		14	Adenocarcinoma	0.435	0.038
		18	Squamous	0.961	0.717
Beer 2002	12118244	96	Adenocarcinoma	0.125	0.293

bold GSEA values <0.05.

* Bound/Expressed.

We also determined the enrichment of particular biological processes within the expression/correlation groups (Table 4). In the lung tumor datasets evaluated, there is a negative correlation between NKX2-1 and regulators of the cell cycle identified in early development, while there is a positive correlation with genes involved in transcriptional regulation. These links might be helpful in understanding the fact that patients with adenocarcinomas with higher levels of NKX2-1 expression have a better prognosis than those with lower or no expression of NKX2-1. For example, we determined that higher levels of NKX2-1 expression correlate to lower level of expression of the target genes Ccnb1, Ccnb2, Cdc2, Cdkn2c, and Mcm2.

**Table 4:** Selected GO categories over-represented within developmental genes correlated to NKX2-1 levels.

*GO*	*Gene Symbols*
*E11.5 positive correlation*	
Cellular metabolism	AASS AGPAT3 AHCYL1 APBB1 ASXL1 BCKDK CREBBP CRY2 CSTF3 CTSD CYB561 DDB1 DNAJC8 DPM2 EDF1 EIF4G1 ELAVL1 ENTPD4 EPC1 ERBB3 ERH ERO1LB FBXW5 FGFR3 FOXA1 FTS GNA11 GTF2H4 GUSB HADHB HERC2 HUWE1 KLF9 LONP METAP1 MLH1 MLLT3 MVK MXD4 MYBBP1A NEK6 NFIB NFYC NONO
Transcription	APBB1 ASXL1 CREBBP EDF1 EPC1 FOXA1 GTF2H4 KLF9 MLLT3 MXD4 MYBBP1A NFIB NFYC NOTCH1 PHF13 POLR2C RAB25 REST SMAD7 SMARCA5 SOX4 SP3 TCEB2 UBP1 UNR ZFP90 ZNF23 ZNF297

*E11.5 negative correlation*	
*GO*	*Gene Symbols*
Macromolecule metabolism	ACVR2 AKR1A1 ALK ARIH1 BAG1 BAG2 BPGM CACYBP CCT3 CDC2 CHAF1B CLK3 CTSL2 DAG1 DNAJB11 DNAJC1 DNAJC10 EIF2B5 EIF5 ERO1L FKBP1A FLJ25059 FLT3 FURIN H2AFJ H2AFX ICT1 IDE ITM1 MAP3K11 MAPK1 MMP14 MRPL16 MRPL30 MRPL48 MRPS17 MRPS18A NDUFAF1 NEDD4 NEDD8 NSMCE1
Cell proliferation	ANLN BCL2 BRRN1 CCNB1 CCNB2 CDC2 CDKN2C CHAF1B EMP1 FLT3 FTSJ3 GMNN KIF11 MAPK1 MCM2 MSH2 NR6A1 ORC2L PARD3 PAWR PRDX1 PSMD8 RBBP7 SMC2L1 SSBP1 TNFRSF7 UBE2C WARS
Cell death	BAG1 BAG2 BCAP31 BCL2 BNIP2 DAD1 DAP3 EI24 EMP1 LIMS1 MAPK1 PAWR SEMA4D TNFRSF7 TUBB TXNL1

*E19.5 positive correlation*	
*GO*	*Gene Symbols*
Signal transduction	ABR ADCY7 ADRB2 ANK3 ARRB1 ATM CLOCK DPYSL3 ENPP2 EPS8 GNA11 IL1R1 IRS1 LY6E NFKBIA NRP1 PKD1 PRKCZ RAB17 RAB24 RAC1 RASGRF1 RGL2 SCGB1A1 TM4SF6 TOLLIP VAPA

***E19.5 negative correlation***	
***GO***	***Gene Symbols***
Catabolism	ADAM17 ANAPC5 BLMH CASP3 CTSB DDX1 DNPEP DPEP1 F7 G6PD GLUD1 PAFAH1B3 PDHA1 PSMD8 PYGB UBE2L3

GO level ≥4; Bayes Factor ≥0; p≤0.03; number of genes≥10.

## Discussion

To identify direct transcriptional targets of Nkx2-1 that could be effectors of its developmental functions we performed ChIP-chip analyses in early and late developing mouse lung. The differential expression pattern of Nkx2-1 at these developmental stages allowed us to evaluate Nkx2-1 targets in cell populations undergoing proliferation at E11.5 and differentiation at E19.5. In this work, we identified a preferential role for Nkx2-1 in direct transcriptional regulation of proliferation-related genes in early development and of ion transport genes in late development.

Nkx2-1 regulation of lung cell proliferation and survival was previously shown in development and tumor cell lines [3], [7], [9], but the effector genes directly regulated by Nkx2-1 were largely unknown. Amongst several proliferation-related genes targeted by Nkx2-1, we identified E2f3, cyclins Ccnb1 and Ccnb2, and c-Met. E2f3 controls the rate of cell proliferation by controlling the G1/S transition and the initiation of DNA synthesis [37], [38] and is expressed in the lung epithelium in early development [39], [40]. Cyclins Ccnb1 and Ccnb2 regulate the G2/M phase transition and are ubiquitously expressed in the lung during development. Ccnb1, E2f3 and other proliferation genes are mostly bound by Nkx2-1 at E11.5 but not at E19.5. Binding of Nkx2-1 to the promoters of these genes correlates with increased expression (Figure S3), and with proliferative state of the epithelial cells in early lung development. c-Met is a proto-oncogene and the HGF receptor tyrosine kinase expressed in E13 mouse lung epithelium and thereafter, where it is involved in mitosis, migration and morphogenesis [41]. Reduction of Nkx2-1 expression in cell lines alters expression of these genes, and slows down cell cycle progression. In vivo, the absence of Nkx2-1 results in impaired lung epithelial lineage expansion and branching morphogenesis. These findings make us speculate that reduced expression of genes involved in cell proliferation and progression of the cell cycle may contribute to the hypomorphic lung phenotype observed in Nkx2-1 null embryos [5], [42], [43]. It will be interesting in the future to determine if altered expression of the genes identified precludes distal lung epithelial progenitor cells to proliferate and engage in the process of branching morphogenesis.

A different context is observed at E19.5, when Nkx2-1 expressing cells are differentiating and preparing for the rapid absorption of luminal fluid and for the first breath [44], [45]. Nkx2-1 binding to ion transport genes in distal lung epithelial cells at E19.5 suggests that Nkx2-1 participates in differentiation of the distal lung epithelium to perform these functions at birth. Gene expression analyses of E18 lungs harboring a Nkx2-1 phosphorylation-deficient mutant also show reduced expression of genes that regulate fluid and electrolyte transport [6] supporting a direct link between Nkx2-1 and these functions.

Our results may also have important implications for understanding NKX2-1 functions in lung cancer. A link between development and tumorigenesis has been suggested in different cancers and their corresponding organ of origin [34]. Genomic associations between human lung cancer subtypes and developing mouse lung indicated that tumors with genomic profiles similar to early lung development correlate to poorer patient's prognosis [35] while tumors with gene expression profiles similar to more differentiated lung cell phenotypes correlate to better patient's prognosis. Developmental genes expressed in tumors, such as NKX2-1 may underlie these associations. Multiple evidences support a dual role for NKX2-1 as a proto-oncogene and tumor suppressor gene in lung cancer. NKX2-1 is considered a lineage specific oncogene since its expression is increased or amplified in some lung tumors [3], [9], [46], [47]. In other analyses NKX2-1 is considered a good prognostic factor, since patients with NSCLC showing high levels of NKX2-1 or amplification of the locus have a better prognosis than those that have lost NKX2-1 expression [10], [48]. NKX2-1 was also proposed as a suppressor of lung adenocarcinoma progression in a mouse model of lung cancer [49]. NKX2-1 target genes, effectors of these functions in lung tumors are also unknown. NKX2-1 and some human homologues of the targets identified in development, including E2F3, CCNB1, CCNB2 and c-MET have been proposed as independent lung tumor markers and prognostic genes. E2F3 is overexpressed in 55–70% squamous cell carcinomas and 79% of adenocarcinomas of the lung. [50], and is associated with high Ki67 in invasive cancers [51]. Increased expression of CCNB1 in NSCLC was suggested as a poor prognostic parameter [52], [53], [54]. CCNB2 and c-MET are also over expressed in adenocarcinomas [55], [56], [57], [58]. Our findings point to NKX2-1 as a direct transcriptional regulator of these independent markers of lung tumorigenesis modulating their level of expression at different stages of tumor progression.

Comparison of mouse lung development and human lung cancer data sets identified cell cycle and proliferation as the largest gene categories involved in both processes. Since early development in most organs involves significant cell proliferation, it is not surprising that most similarities between NKX2-1 targets in early lung development and tumors are related to cell cycle and proliferation genes [59]. It is possible that tumor cells maintaining lung-lineage characteristics use tissue/cell specific factors including NKX2-1 to control proliferation and other functions. In addition to the genes identified in these studies, there may be other genes uniquely regulated by NKX2-1 in tumors and not in development; to identify those genes it will be necessary, in the future, to analyze direct NKX2-1 binding in primary tumors or alternatively in tumor cell lines.

It is intriguing that many cell proliferation genes inversely correlate to the levels of NKX2-1 in NSCLC. This inverse correlation may explain the poorer prognosis of patients with NSCLC with low levels of NKX2-1. To determine if the reverse correlation is due to repression by direct NKX2-1 binding, ChIP analyses may be performed in human tumor tissues or tumor cell lines. Alternatively molecular analyses of the promoters of these genes in cell lines may provide information about the repression of these genes by Nkx2-1 binding. These experiments will be the focus of our future studies. Adenocarcinomas sub-classification based on gene expression profiling was proposed to improve prediction of malignant potential and prognosis [35]. The associations identified in our studies may contribute to the molecular classification of these tumors and clarify NSCLC heterogeneity, holding great potential to increase the understanding of this disease.

Our findings point to potential molecular mechanisms by which Nkx2-1 may differentially regulate transcriptional activity. First, inverse correlation in expression level of NKX2-1 and targets in tumors, and of Nkx2-1 and c-Met in MLE15 cells suggests a more widespread role of Nkx2-1 in transcriptional repression. This effect could be by direct binding or, alternatively, by recruitment or activation of transcriptional repressors by Nkx2-1 to down-regulate particular genes. Nkx2-1 has been mostly linked to transcriptional activation in lung and other organs [2], although neuropilin-2 [20] and RAGE [60] have been reported to be down-regulated by direct binding of Nkx2-1 to a cis-element in their promoters strongly supporting Nkx2-1 repressor activity.

Second, there are target genes bound by Nkx2-1 at both developmental time points, whereas others are bound only at E11.5 or E19.5. Interactions with alternative co-factors differentially expressed at each time point might result in differential affinity and binding to alternative targets [21]. Different isoforms and/or modifications of Nkx2-1 proteins by phosphorylation, acetylation or oxidation may affect affinity for particular *cis*-elements or interactions to different co-factors at each time point [17], [22], [61]. Identification of different forms of Nkx2-1 protein at E11.5 and E19.5 will be necessary to fully understand the different targets in alternative cell contexts.

The specificity of Nkx2-1 binding has also been linked to promoter structure [62]. Differences in chromatin modifications surrounding these *cis*-elements in different cell contexts could affect affinity of Nkx2-1 proteins. For example binding of Nkx2-1 to the Sftpb promoter is prevented by DNA methylation of the Sftpb promoter in non-expressing tissues, such as thyroid [63]. To fully understand the differences in binding patterns in different cell contexts we will need to identify the consensus sequences and the chromatin modifications of the binding sites in genomic regions only bound at E11.5 or only bound at E19.5. Future analyses will be focused in discerning these alternatives and characterizing Nkx2-1 binding sites in different contexts.

Finally, we observed strong binding to brain and thyroid genes not expressed at detectable levels in developing lung, suggesting that binding of Nkx2-1 does not imply activation of transcription. In certain cases, binding may precede activation such as in the case of Sftpa and may prime the gene for activation upon recruitment of other transcription complexes and/or co-factors to the promoter. The identification of unique Nkx2-1 targets at E11.5 and E19.5 will facilitate the evaluation of possible mechanisms that control specificity.

Overall, we provide novel insights into biological processes regulated by Nkx2-1 in different cell contexts in development, and cancer. We identified Nkx2-1 direct target genes in mouse lung epithelium that are primary effectors of Nkx2-1 functions, in particular cell proliferation genes. We showed that expression levels of the target genes depend on NKX2-1 levels in NSCLC. NKX2-1 has been associated to longer, similar or shorter patient survival in NSCLC, depending on expression levels [10], [64]. Therefore, evaluation of NKX2-1 expression levels relative to its downstream targets will provide a way to sub-classify NSCLCs, and understand the mechanisms underlying associations to patient survival.

## Materials and Methods

### Antibodies

A rabbit polyclonal Nkx2-1 antibody (EMD-Millipore-Upstate 07-601) was used in ChIP assays, immunofluorescence analyses and western blots; a rabbit monoclonal Nkx2-1 antibody (Abcam, ab76013) was used in immunofluorescence analyses and western blots. The later did not work in ChIP assays in the conditions tested. Although some antibodies work well in experiments such as western blots, or immunocytochemistry, they may not necessarily work in ChIP assays since the fixative conditions used may mask or destroy some epitopes. Monoclonal antibodies, such as ab76013, have higher specificity than polyclonal sera, but polyclonal sera, such as 07-601, may recognize several epitopes of the target, increasing signal levels [65], [66].

We selected the rabbit polyclonal Nkx2-1 antibody for the ChIP-chip analyses based on our previous results [63] and additional experiments performed for this manuscript. We have previously shown specificity of this antibody in ChIP-PCR analyses in vitro and in vivo. Briefly MLE15 cells were transfected with a wild type or mutant Sftpb promoter construct containing mutations of four Nkx2-1 consensus sites. ChIP-PCR assays were performed with the rabbit polyclonal Nkx2-1 antibody or IgG. Mutation of the Nkx2-1 binding sites abolished binding of Nkx2-1 to the promoter and therefore no PCR band is observed when the mutant DNA is immunoprecipitated with the Nkx2-1 antibody. PCR with oligonucleotides in the β-actin locus were also used to indicate the absence of non-specific binding. In addition we showed using the same antibody that Nkx2-1 binds to the endogenous Sftpb promoter in the lung but not the thyroid where the DNA in this region is highly methylated. This same antibody shows binding of Nkx2-1 to the thyroglobulin gene promoter in both tissues but not to β-actin. Supporting experiments that show the specificity of the antibody are included in the results section where down regulation of Nkx2-1 gene expression by shRNAs results in reduction in intensity of the two major bands in Western blots. Those shRNAs do not reduce the levels of the non-specific protein β-actin.

### Chromatin immunoprecipitation

Lungs were dissected from CD1 mice (Charles River Laboratories), minced and immediately immersed in 2 volumes of 1×PBS. For independent immunoprecipitations (IP), we pooled ten E11.5 mouse lungs (n = 2) or five E19.5 mouse lungs (n = 3). Lungs were chemically cross linked by addition of one-tenth volume of fresh 11% formaldehyde solution, and incubated for 10 minutes at room temperature. Formaldehyde was quenched with 2.5 M glycine solution and tissues rinsed twice in 1×PBS. Crosslinked lung samples were flashed frozen in liquid nitrogen and stored at −80°C. To solubilize and shear crosslinked DNA, lungs were lysed and sonicated on ice in a Branson Ultrasonic Sonicator coupled to a Fisher Scientific Sonic dismembrator 500 power supply. Samples were sonicated at 90% amplitude for 12 cycles of 30 second pulses with 60 second pause between pulses, yielding fragments of about 500 bp. Part of the whole cell extract was saved as input material and the rest was incubated overnight at 4°C with 100 µl of Dynal Protein G magnetic beads (Invitrogen) pre-incubated with 10 µl of the Nkx2-1 antibody (07-601, Upstate-Millipore) or IgG (Santa Cruz Biotechnologies, Inc). Beads were washed 4–5 times with RIPA buffer and 1 time with TE containing 50 mM NaCl. Bound complexes were eluted from the beads by addition of elution buffer and by heating at 65°C for 15 minutes with 2 minutes interval of vortexing. Crosslinking in the IP and input samples was reversed by overnight incubation at 65°C. Because Nkx2-1 null mice form only a lung rudiment due to impaired branching morphogenesis we could not use those lungs as control in the ChIP-chip experiments.

### ChIP-chip sample preparation and hybridization

ChIP-chip experiments were performed as described previously [23]. Briefly, immunoprecipitated DNA (IP) and whole cell extract DNA (input) were purified by treatment with RNAse A, proteinase K and multiple phenol: chloroform: isoamyl alcohol extractions. Purified DNA was blunt-ended and ligated to linkers, and amplified using a two-stage PCR protocol. Amplified immunoprecipitated DNA (IP) was labeled with Cy5 fluorophore, and amplified whole cell extract DNA (input) with Cy3 fluorophore. Both IP and input were purified using Bioprime random primer labeling kits (Invitrogen). Cy5 and Cy3 labeled DNAs were mixed (∼5 µg of each) and hybridized to arrays in Agilent hybridization chambers for 40 hours at 40°C, washed and immediately scanned.

### Data Extraction, Normalization and Analysis

We used mouse promoter microarray sets (Agilent Technologies, AMADID: 014716 and 014717), consisting of 2 slides containing ∼244,000 60-mer oligonucleotides each, covering ∼17,000 mouse genes −5.5 kb upstream to +2.5 kb downstream from the transcriptional start sites. Replicate experiments were performed for each time point and each array was scanned using an Agilent scanner model G2565BA. The image analysis was performed using Agilent's Feature Extraction software v.9.5.3.1 set to the default ChIP protocol. To combine the replicates for each time point, the raw data was background subtracted and median normalized using limma library, part of the Bioconductor project, in the R statistical environment [67], [68]. The difference of binding between the IP and Input (Cy5/Cy3), for each probe/feature, was analyzed using empirical Bayes method implemented in the limma package. P-values obtained from the multiple comparison tests were corrected by false discovery rates. In addition, for the binding profiles, we calculated average binding ratio (Cy5/Cy3) for each probe by averaging the ratio between the candidate bound probe and the 2 closest neighboring probes. The complete data set is available at GEO Accession Number GSE23043.

### Biological process and pathway analyses

Probes at E11.5 and at E19.5 with p≤0.001 and log_2_ binding ratio ≥0.75 were selected to query the Expression Analysis Systematic Explorer (EASE) to discover enriched biological themes within the probe sets [29]. Multiple probes within a probe set representing binding to the same gene were consolidated by selection of the probe with the highest fold difference (corresponding to the peak of the binding region). Using median stringency settings we identified overrepresented functional annotation clusters (p≤0.05) for each list. We performed pathway enrichment analyses by using Ingenuity database through IPA interface. Enriched canonical pathways at significance level of p≤0.05 were compared between time points.

### Correlation analyses

A list of 374 unique mouse gene symbols corresponding to genes both bound by Nkx2-1 and expressed in E11.5 mouse lung epithelium was obtained by intersecting a list of 1362 unique gene symbols corresponding to significant Nkx2-1 binding peaks at day E11.5 (Dataset S1) and a list of genes called present (detection p value≤0.05) in all measurements in a microarray analysis of isolated lung epithelium at day E11.5 [26], processed in Affymetrix, MG-U74v2 set and Microarray Suite 5 (MAS5). Gene symbols were translated to 350 unique mouse Entrez Gene IDs using a table obtained from the Homologene resource at NCBI (http://www.ncbi.nlm.nih.gov/sites/entrez?db=homologene).

A list of 183 unique mouse gene symbols corresponding to genes both bound by Nkx2-1 at E19.5 and expressed in E18 mouse lung was obtained by intersecting a list of 1358 unique gene symbols corresponding to significant Nkx2-1 binding peaks at day E19.5 (Dataset S1), translated to mouse Entrez Gene IDs using the Homologene table, and 2611 unique Entrez Gene IDs called present in all measurements in E18 lung, obtained from GEO series GSE10889 [27], processed with MAS5 and normalized with mouse Entrez Gene-specific CDF. All mouse Entrez Gene IDs in both gene sets were then translated to the Entrez Gene IDs of their human homologues using the Homologene table.

The human lung tumor data sets used in this study were identified in a search of the literature and the Gene Expression Omnibus (GEO) for microarray expression profiles of primary lung adenocarcinomas and squamous carcinomas (Table 3). The search was limited to include only those experiments performed using Affymetrix microarray platforms and for which raw CEL files were publicly available. Gene-specific expression levels were obtained using the Robust Multiarray Average (RMA) [68] and a Chip Definition File (CDF) that collapses oligonucleotide probes to probesets corresponding to Entrez Gene IDs [69], obtained from http://brainarray.mbni.med.umich.edu/Brainarray/Database/CustomCDF/). All computations were performed in R (version 2.9.2). To correlate expression of Nkx2-1 target genes to the levels of Nkx2-1, the 350 human homologues at E11.5 and the 183 at E19.5 were ranked according to the absolute value of the Pearson correlation coefficient of its expression level and that of NKX2-1, within each human lung tumor dataset. GSEA was then performed to determine whether the genes in the E11.5 and E19.5 gene sets were significantly enriched toward the top of each ranked list. A total of 1000 random permutations were used to determine significance in each GSEA analysis. Those analyses for which no permutations had greater significance than the gene set being tested are denoted as p<0.001. To elucidate biological signatures from the developmental genes correlated or anti-correlated with NKX2-1 levels in human lung tumors we used GATHER tool [70].

### Lentivirus production and cell transduction

Mouse lung epithelial MLE 15 cells (a gift of Dr. Jeffrey A. Whitsett, Cincinnati Children's Hospital Medical Center) were cultured in modified conditions as described previously [71], and transduced with lentivirus expressing shRNAs targeting mouse and human Nkx2-1 as we described previously [63]. We used individual clones TRCN0000020449, TRCN0000020450, and TRCN0000086264 contained in shRNA sets RHS4533 and RMM4534 (Open Biosystems). Lentivirus empty vector (RHS4080, Open Biosystems) was used as non-silencing control.

### ChIP-PCR, ChIP-qPCR and qRT-PCR

E11.5 and E19.5 immunoprecipitated lung DNA (IP) using the Nkx2-1 antibody (07-601, Upstate-Millipore) or its corresponding IgG (Santa Cruz Biotechnologies), the input material and genomic DNA were analyzed by PCR, or by qPCR in a StepOnePlus (Applied Biosystems). Using equal amount of DNA from each sample, we performed PCR using polymerase (Qiagen) and primers for various selected targets of Nkx2-1 listed in Table S7. qPCR was performed using SYBR Green (Applied Biosystems). For expression analyses, isolated RNA (1 µg) was reverse transcribed (RT) using TaqMan reverse transcription reagents (Applied Biosystems) and Taqman assays on demand and Taqman Master Mix (Applied Biosystems) [63].

### Cell proliferation analyses

For cell cycle analysis by FACS, MLE15 cells transduced with Nkx2-1 shRNA or non silencing vector were harvested, washed twice with 1XPBS, and re-suspended in 500 µl 1XPBS, at 4°C. Ice-cold ethanol (5 ml) was added drop-wise to the cell suspension and incubated overnight at −20°C. Cells were stained in 1 ml propidium iodide (PI) solution (1XPBS +50 µg/ml PI +100 µg/ml RNAse A), filtered using a 40 µM filter (BD Biosciences) and incubated at 4°C in the dark for 20 minutes before analyzing them by flow cytometry (BD FACScan; BD Biosciences, and FlowJo analysis software, Treestar).

Cell growth was measured by counting cell numbers of MLE15 cells transduced with Nkx2-1 shRNA or non silencing vector at 24 hours intervals for 4 days. We initiated the cultures seeding 5000 cells in triplicate. Cultured cells were trypsinized at 24, 48, 72 and 96 hours resuspended in equal volume of media and counted using a Scepter handheld automated cell counter (Millipore).

### Western Blot Analyses

Mouse lungs and thyroid were dissected and homogenized in 1 ml of RIPA buffer (50 mM Hepes (pH 7.6), 1 mM EDTA, 0.7% Na deoxycholate, 1% NP-40, 0.5 M LiCl) plus Complete Protease Inhibitor Cocktail (Roche) and incubated in a rotator at 4°C for 2 hours. MLE15 and E10 mouse cells, and H441 and H661 human cells, grown in 10-cm diameter plates, were washed twice with 1XPBS, resuspended in 1 ml of RIPA buffer plus complete protease inhibitors cocktail and lysed by gentle vortexing every 5 minutes for 30 minutes. Samples were centrifuged at 14,000 rpm for 15 minutes, and the supernatant was collected and used in western blot analyses. Thirty micrograms of protein were electrophoresed in 12.5% Hydro Colorize gels (Bio-Rad) and electro-transferred to Immobilon-P Transfer membranes (Millipore). Proteins were detected with the Nkx2-1 antibody (07-601, EMD-Millipore-Upstate-), the Nkx2-1 antibody (ab76013; Abcam), β-Actin [63], and/or Gapdh (G9545, Sigma-Aldrich) and their corresponding secondary antibodies, in a LAS-4000 chemiluminescence image analyzer (Fuji). To compare the signals obtained by these antibodies and previously published western blots [12], [21] we used the 8G7G3/1 LabVision mouse monoclonal antibody (Thermo Fisher Scientific) following the manufacturer protocols. Data are shown in Supplementary Figure S5. Similar bands are detected between 40 and 50 kDa with the 07-601 rabbit polyclonal and the 8G7G3/1 mouse monoclonal antibodies. The ab76013rabbit monoclonal antibody only detects one band in that range.

### Immunohistochemistry

E11.5 whole embryos and E19.5 dissected lungs were fixed in freshly prepared 4% paraformaldehyde in 1XPBS, pH 7.4, at 4°C for 16 hours. For immunohistochemistry, tissues were embedded in paraffin following standard processing with ethanol dehydration. Tissue sections (6 µm) were deparaffinized and hydrated by standard methods. Antigen retrieval was done using an Antigen Unmasking solution (Vector Laboratories). Endogenous peroxidase was quenched with 3% H_2_O_2_ in methanol for 15 minutes. Blocking was performed with 2% normal goat serum in 1XPBS at room temperature for 1 hour. The tissues were incubated with the Nkx2-1 antibody (ab76013, Abcam, 1∶500) at 4°C for 16 hours, and then washed with 1XPBS (5 minutes, twice). Antibody binding was detected with the Vectastain Elite ABC kit (Vector Laboratories) and diaminobenzidine (DAB) as substrate. Images were taken using Leitz Aristoplan microscope. For immunofluorescence, tissues were washed in 1XPBS for 30 minutes, dehydrated in 7.5% sucrose/1XPBS, pH7.4 for 1 hour and then 30% sucrose/1XPBS, pH 7.4 at 4°C for 16 hours. They were then embedded with optimal cutting temperature (OCT) medium in tetra-fluoro-ethane (TFE), liquefied in liquid nitrogen. Sections (6 µm) of these tissues were washed with 1XPBS (5 minutes, twice), blocked in 0.5% goat serum in PBS (1 hour) and incubated for 16 hours at 4°C with a mixture of Nkx2-1 antibodies [(ab76013, Abcam, 1∶500) or (07-601, Upstate-Millipore, 1∶500)] and mouse anti-human Ki67 antibody (550609, BD Biosciences, 1∶250). They were washed with PBS (5 minutes, twice), incubated with M.O.M.™ Biotinylated Anti-Mouse IgG Reagent (FMK-2201, Vector Laboratories, 1∶200) for 30 minutes, washed with PBS (5 minutes, twice), and exposed to a mixture of Alexafluor 488 goat anti rabbit IgG (H+L) (A11008, Invitrogen, 1∶200) and Streptavidin conjugated Cy3 at (43-4315, Invitrogen, 1∶2000) for another 30 minutes. After washing in PBS (5 minutes, twice), the sections were air dried and cover slipped with Prolong Gold (P36935, Invitrogen). Images were taken using the LSM 510 Axiovert 200 M.

## Supporting Information

Figure S1 **Genome-wide patterns of Nkx2-1 binding.** Location of Nkx2-1 binding in all mouse chromosomes in E11.5 (red) and E19.5 (green) lungs. X axis (chromosomal location), y axis (binding signal intensity).
(TIF)

Figure S2 **Nkx2-1 binding patterns to selected target genes in lung development.** Binding profiles of Nkx2-1 to newly identified target genes (left panel). Chromatin immunoprecipitation-qPCR validation of Nkx2-1 binding to target genes (right panel). IP DNA from E11.5 and E19.5 lungs, input and IgG immunoprecipitated control were used in qPCR analyses. Oligonucleotides in the promoter region were used to analyze binding of Nkx2-1, (n = 3). Data are expressed relative to the input.
(TIF)

Figure S3 **Relative expression of selected Nkx2-1 target genes in E12 and E18 developing mouse lung extracted from the expression microarray dataset GEO series GSE 10889 (27).**
(TIF)

Figure S4 **Nkx2-1 levels in human lung tumors significantly correlate with expression of developmental Nkx2-1 target genes.** Additional heatmaps of human lung tumor genes identified in GSE 12667 dataset showing gene expression levels of the human homologues of Nkx2-1 target genes identified in mouse lung development at E11.5 (upper panel) and E19.5 (lower panel); genes are organized in the same order as in Figure 4, according to the Pearson correlation value (y axis) to NKX2-1 expression (x axis).
(TIF)

Figure S5 **Comparison of three commercial Nkx2-1 antibodies.** Western blot experiments were performed using MLE15 lung epithelial cell protein extracts. Nkx2-1 rabbit polyclonal antibody (EMD-Millipore-Upstate), rabbit monoclonal antibody (Abcam) and mouse monoclonal antibody (LabVision, Fisher Scientific) detect a strong band between 40–45 kD (upper black arrow). Bands of lower intensity are detected around 40 kD with the rabbit polyclonal and the mouse monoclonal antibodies (lower black arrow). Other bands of minor intensity are detected (*) but their identity is unknown. The mouse IgG light chain is detected using the mouse monoclonal antibody (**).
(TIF)

Table S1 (a). Target genes at E11.5 (log(2) >0.75, p≤0.001). (b) Target genes at E19.5 (log(2) >0.75, p≤0.001).
(DOC)

Table S2 Nkx2-1 target genes expressed in lung development and correlated to NKX2-1 levels in human lung tumor datasets.
(DOC)

Table S3 Genes bound and regulated by Nkx2-1 in human fetal lung epithelial cells.
(DOC)

Table S4 (a) E11.5 overrepresented biological processes identified by EASE analysis p<0.05. (b) E19.5 overrepresented biological processes identified by EASE analysis (p<0.05).
(DOC)

Table S5 Overrepresented canonical pathways identified by Ingenuity Pathway Analysis Software.
(DOC)

Table S6 Nkx2-1 target genes genes included in Cancer pathways identified by IPA.
(DOC)

Table S7 PCR and qPCR Oligonucleotide sequences.
(DOC)
