# Supplementary figures and images for: Genome-Wide Analyses of Nkx2-1 Binding to Transcriptional Target Genes Uncover Novel Regulatory Patterns Conserved in Lung Development and Tumors
Source: PLoS One. 2012 Jan 5;7(1):e29907. doi: 10.1371/journal.pone.0029907 (PMC3252372; doi:10.1371/journal.pone.0029907)

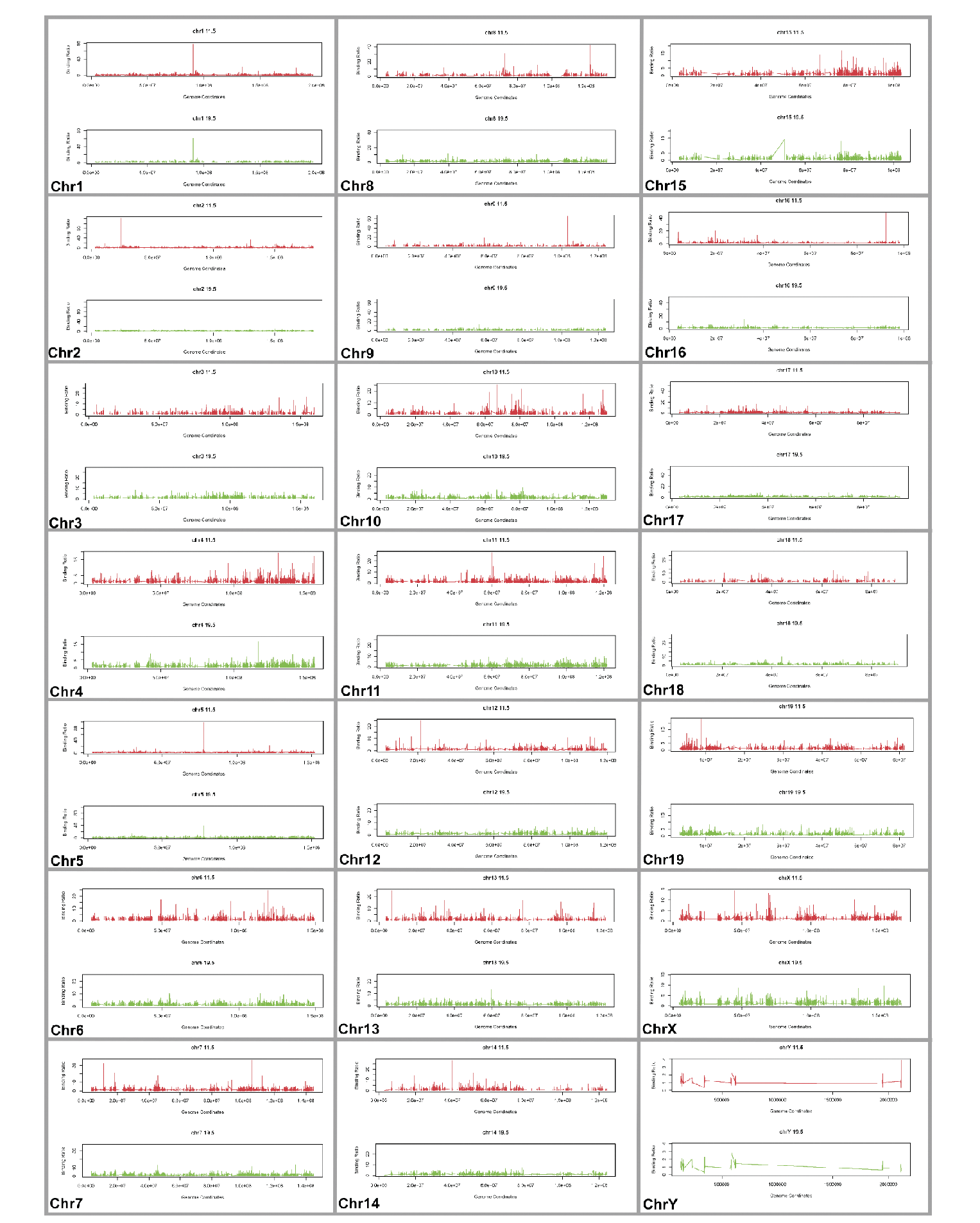

Supplement: Figure S1 — Genome-wide patterns of Nkx2-1 binding. Location of Nkx2-1 binding in all mouse chromosomes in E11.5 (red) and E19.5 (green) lungs. X axis (chromosomal location), y axis (binding signal intensity). (TIF) [file pone.0029907.s001.tif]

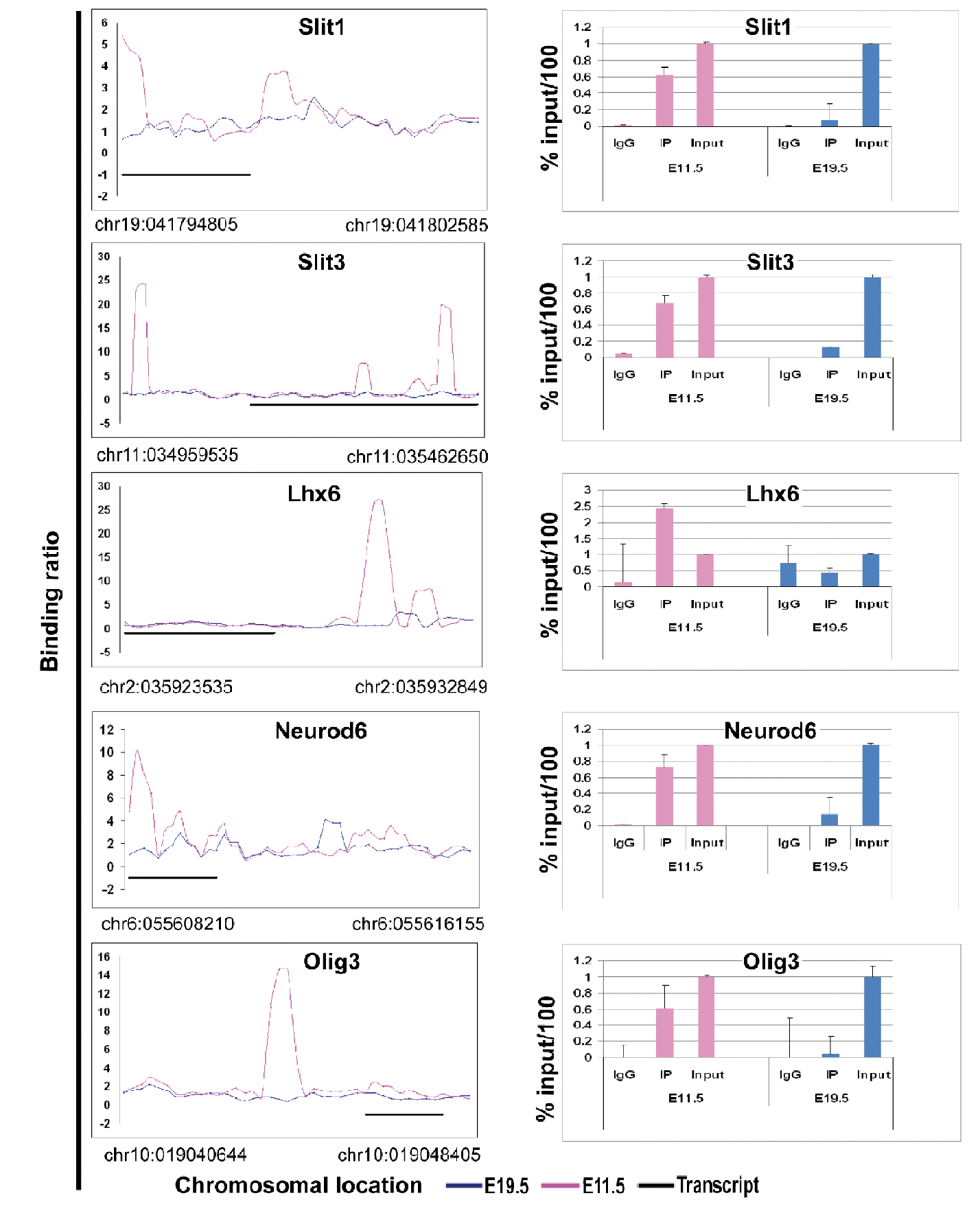

Supplement: Figure S2 — Nkx2-1 binding patterns to selected target genes in lung development. Binding profiles of Nkx2-1 to newly identified target genes (left panel). Chromatin immunoprecipitation-qPCR validation of Nkx2-1 binding to target genes (right panel). IP DNA from E11.5 and E19.5 lungs, input and IgG immunoprecipitated control were used in qPCR analyses. Oligonucleotides in the promoter region were used to analyze binding of Nkx2-1, (n = 3). Data are expressed relative to the input. (TIF) [file pone.0029907.s002.tif]

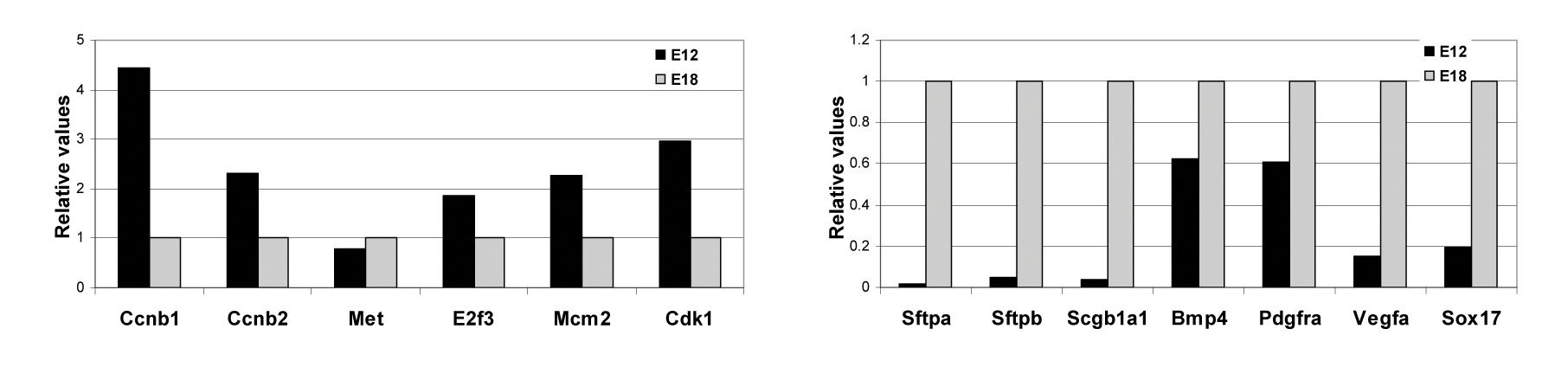

Supplement: Figure S3 — Relative expression of selected Nkx2-1 target genes in E12 and E18 developing mouse lung extracted from the expression microarray dataset GEO series GSE 10889 (27). (TIF) [file pone.0029907.s003.tif]

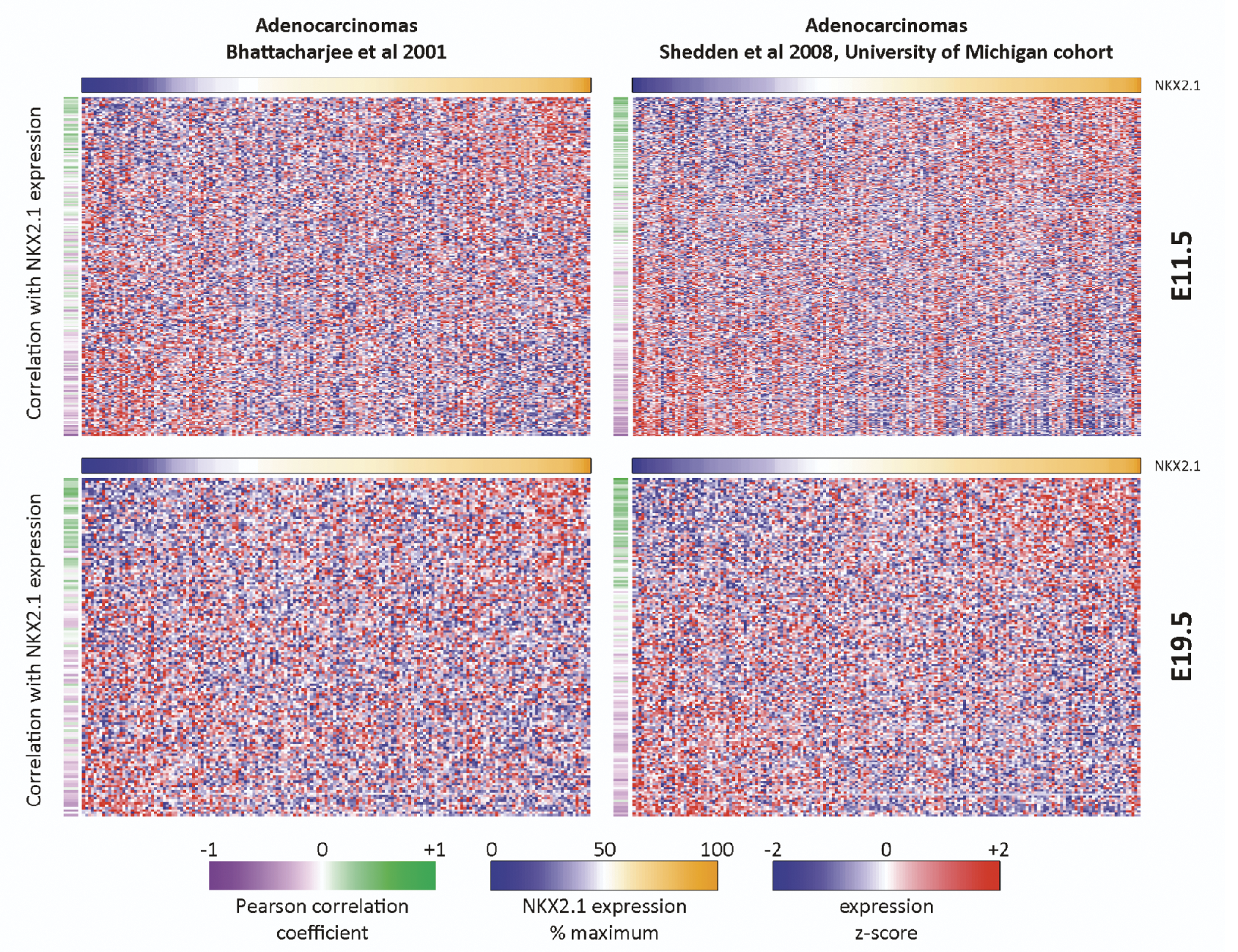

Supplement: Figure S4 — Nkx2-1 levels in human lung tumors significantly correlate with expression of developmental Nkx2-1 target genes. Additional heatmaps of human lung tumor genes identified in GSE 12667 dataset showing gene expression levels of the human homologues of Nkx2-1 target genes identified in mouse lung development at E11.5 (upper panel) and E19.5 (lower panel); genes are organized in the same order as in Figure 4, according to the Pearson correlation value (y axis) to NKX2-1 expression (x axis). (TIF) [file pone.0029907.s004.tif]

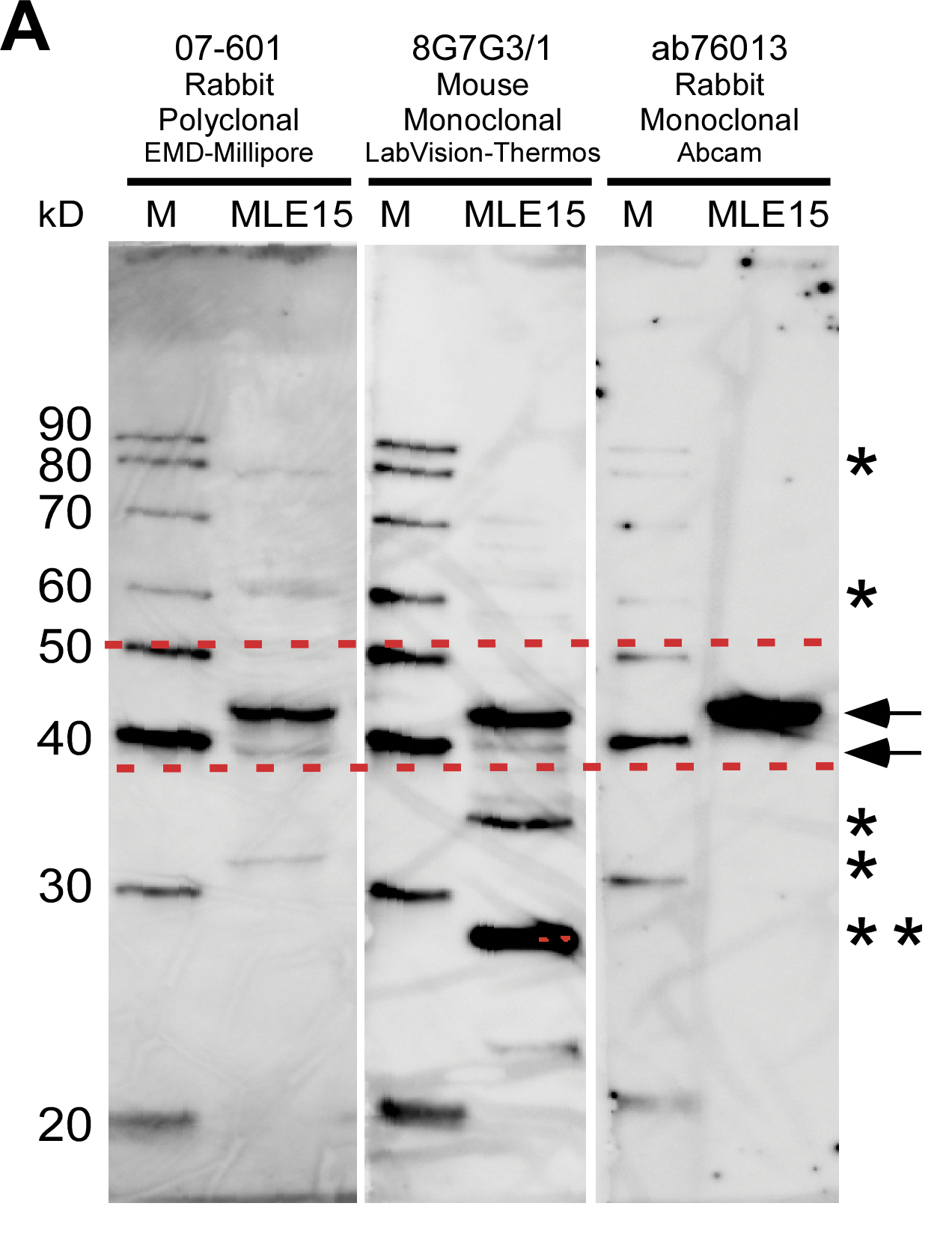

Supplement: Figure S5 — Comparison of three commercial Nkx2-1 antibodies. Western blot experiments were performed using MLE15 lung epithelial cell protein extracts. Nkx2-1 rabbit polyclonal antibody (EMD-Millipore-Upstate), rabbit monoclonal antibody (Abcam) and mouse monoclonal antibody (LabVision, Fisher Scientific) detect a strong band between 40–45 kD (upper black arrow). Bands of lower intensity are detected around 40 kD with the rabbit polyclonal and the mouse monoclonal antibodies (lower black arrow). Other bands of minor intensity are detected (*) but their identity is unknown. The mouse IgG light chain is detected using the mouse monoclonal antibody (**). (TIF) [file pone.0029907.s005.tif]
