# Supplementary material for: Genome-Wide Analyses of Nkx2-1 Binding to Transcriptional Target Genes Uncover Novel Regulatory Patterns Conserved in Lung Development and Tumors
Source: PLoS One. 2012 Jan 5;7(1):e29907. doi: 10.1371/journal.pone.0029907 (PMC3252372; doi:10.1371/journal.pone.0029907)
Supplement: Table S1 — (a). Target genes at E11.5 (log(2) >0.75, p≤0.001). (b) Target genes at E19.5 (log(2) >0.75, p≤0.001). (DOC) [file pone.0029907.s006.doc]

|  |  |  |  |  |  |  |
| --- | --- | --- | --- | --- | --- | --- |
| *Table S1a. Target genes at E11.5 (log(2) >0.75, p*≤ *0.001)* | | | | | | |
| ***ProbeUID*** | ***ProbeName*** | ***GeneName*** | ***SystematicName*** | ***Description*** | ***AverageLog*** | ***AveragePV*** |
| 38686 | A_68_P23815278 | Clock | chr5:077379328-077379372 | PROMOTER | 2.020158946 | 4.335E-23 |
| 105591 | A_68_P21065497 | Psd4 | chr2:024205514-024205566 | PROMOTER | 2.207039257 | 4.495E-23 |
| 120370 | A_68_P26783912 | Rab6b | chr9:102971566-102971612 | INSIDE | 1.975705129 | 4.67E-23 |
| 213748 | A_68_P20425265 | Arl4c | chr1:090532783-090532827 | INSIDE | 1.965935785 | 4.67E-23 |
| 50286 | A_68_P23885799 | Cxcl2 | chr5:091977027-091977086 | PROMOTER | 2.211559624 | 5.945E-23 |
| 169498 | A_68_P31102748 | Ube2i | chr17:025000746-025000790 | INSIDE | 1.809592093 | 7.53E-23 |
| 28036 | A_68_P24991144 | Rshl1 | chr7:018208949-018209008 | PROMOTER | 1.915612186 | 7.785E-23 |
| 57278 | A_68_P28280361 | Adam17 | chr12:021618930-021618989 | INSIDE | 2.027995801 | 8.8E-23 |
| 16833 | A_68_P24971245 | V1rg3 | chr7:011094521-011094574 | PROMOTER | 1.99939957 | 8.945E-23 |
| 90547 | A_68_P26214348 | Gm587 | chr8:122391488-122391540 | PROMOTER | 1.766664238 | 9.195E-23 |
| 67170 | A_68_P25948079 | Atp13a1 | chr8:072736065-072736109 | INSIDE | 1.653916936 | 1.45E-22 |
| 154236 | A_68_P28903598 | Prlpm | chr13:027804012-027804071 | PROMOTER | 1.676097278 | 1.556E-22 |
| 173281 | A_68_P31775674 | Fech | chr18:064616649-064616708 | PROMOTER | 1.97635108 | 1.686E-22 |
| 44380 | A_68_P26290478 | Yap1 | chr9:008008792-008008851 | PROMOTER | 1.96444809 | 1.714E-22 |
| 184756 | A_68_P21630582 | Avp | chr2:130275593-130275637 | PROMOTER | 1.613344937 | 1.81E-22 |
| 13551 | A_68_P23303523 | Matn1 | chr4:130217977-130218026 | INSIDE | 1.64017375 | 2.175E-22 |
| 144973 | A_68_P20066014 | Tcfap2d | chr1:019083843-019083902 | PROMOTER | 1.754883834 | 2.188E-22 |
| 185962 | A_68_P24854730 | Tas2r117 | chr6:132763229-132763277 | PROMOTER | 1.641629777 | 2.243E-22 |
| 29658 | A_68_P28802641 | Pitrm1 | chr13:006548885-006548933 | INSIDE | 1.826505719 | 2.647E-22 |
| 71541 | A_68_P23127479 | Cyp2j11 | chr4:095840539-095840585 | INSIDE | 1.630497878 | 2.815E-22 |
| 125057 | A_68_P21744391 | Fkbp1a | chr2:151229364-151229423 | PROMOTER | 1.841954363 | 3.084E-22 |
| 39897 | A_68_P27095740 | Nr2e1 | chr10:042276206-042276260 | PROMOTER | 1.647035601 | 4.36E-22 |
| 70903 | A_68_P24792534 | Lrtm2 | chr6:119300696-119300740 | PROMOTER | 1.482150277 | 5.1E-22 |
| 54759 | A_68_P31619641 | Dnajc18 | chr18:035830212-035830261 | PROMOTER | 1.895065044 | 5.573E-22 |
| 237961 | A_68_P28265938 | Ntsr2 | chr12:016675674-016675723 | PROMOTER | 1.492141586 | 6.11E-22 |
| 134774 | A_68_P29561120 | Dydc2 | chr14:039983293-039983348 | PROMOTER | 1.651942816 | 7.186E-22 |
| 49662 | A_68_P26289755 | Birc3 | chr9:007873962-007874021 | PROMOTER | 1.589529478 | 7.505E-22 |
| 152658 | A_68_P30349978 | Ppp1r16a | chr15:076520367-076520411 | INSIDE | 1.414453884 | 7.57E-22 |
| 120131 | A_68_P21612924 | Adra2b | chr2:127051393-127051437 | PROMOTER | 1.556825058 | 8.477E-22 |
| 9479 | A_68_P27269896 | Krtap12-1 | chr10:077161955-077162013 | PROMOTER | 1.676136899 | 9.205E-22 |
| 70376 | A_68_P24044685 | Erp29-Tmem116 | chr5:121716368-121716427 | DIVERGENT_PROMOTER | 1.886157515 | 9.967E-22 |
| 226563 | A_68_P30576384 | Tssk2 | chr16:017809969-017810018 | PROMOTER | 1.457005195 | 1.205E-21 |
| 10670 | A_68_P23656658 | Qdpr | chr5:045737932-045737985 | INSIDE | 1.505478661 | 1.286E-21 |
| 210919 | A_68_P26542008 | Scamp5 | chr9:057265644-057265688 | INSIDE | 1.399064031 | 1.357E-21 |
| 21075 | A_68_P27844767 | Smcr8 | chr11:060598639-060598694 | DOWNSTREAM | 1.494776161 | 1.406E-21 |
| 8662 | A_68_P31159691 | Cdsn | chr17:035159681-035159725 | PROMOTER | 1.368101922 | 1.715E-21 |
| 180566 | A_68_P27190675 | Sirt1 | chr10:062737974-062738025 | PROMOTER | 1.58996105 | 1.718E-21 |
| 188511 | A_68_P23359981 | Padi4 | chr4:140018271-140018316 | INSIDE | 1.448388172 | 2.112E-21 |
| 32342 | A_68_P30325057 | Kcnk9 | chr15:072378206-072378255 | PROMOTER | 1.99330515 | 2.405E-21 |
| 217849 | A_68_P26477029 | Sidt2 | chr9:045710204-045710263 | PROMOTER | 1.325245217 | 2.465E-21 |
| 226254 | A_68_P28181805 | Aatk | chr11:119836249-119836293 | INSIDE | 1.535776925 | 2.524E-21 |
| 155748 | A_68_P24657031 | Slc25a26 | chr6:094461139-094461198 | PROMOTER | 1.637036164 | 2.536E-21 |
| 71905 | A_68_P26351350 | Anln | chr9:022081918-022081977 | DOWNSTREAM | 1.330220028 | 2.67E-21 |
| 35668 | A_68_P25971576 | Tom1 | chr8:077931506-077931565 | INSIDE | 1.969504663 | 2.858E-21 |
| 49208 | A_68_P25465167 | Thumpd1 | chr7:119511103-119511162 | INSIDE | 1.814392163 | 2.878E-21 |
| 157853 | A_68_P22549107 | Tacr3 | chr3:134766555-134766599 | INSIDE | 1.440585061 | 2.992E-21 |
| 78038 | A_68_P24517707 | V1rc15 | chr6:066486921-066486980 | INSIDE | 1.6093377 | 3.121E-21 |
| 216452 | A_68_P29227291 | EG218444 | chr13:094170174-094170233 | PROMOTER | 1.690197896 | 3.122E-21 |
| 72985 | A_68_P22662044 | Lhx8 | chr3:154271083-154271131 | PROMOTER | 1.30850691 | 3.259E-21 |
| 215852 | A_68_P30465078 | Slc38a2 | chr15:096527624-096527683 | INSIDE | 1.32345895 | 3.44E-21 |
| 161471 | A_68_P27212408 | Egr2 | chr10:066928853-066928912 | PROMOTER | 1.877039316 | 3.901E-21 |
| 156962 | A_68_P21888775 | Phactr3 | chr2:178069529-178069583 | PROMOTER | 1.517756566 | 3.936E-21 |
| 9588 | A_68_P31929995 | Pcnxl3 | chr19:005664905-005664954 | INSIDE | 1.396260097 | 4.835E-21 |
| 94371 | A_68_P21816747 | Wfdc12 | chr2:163883991-163884037 | PROMOTER | 1.369992223 | 5.058E-21 |
| 145569 | A_68_P23981048 | Noc4l | chr5:110889482-110889529 | INSIDE | 1.339171398 | 5.634E-21 |
| 114329 | A_68_P22347262 | Vps45 | chr3:096148580-096148639 | PROMOTER | 1.791579584 | 5.818E-21 |
| 37589 | A_68_P28223018 | Rhob | chr12:008528273-008528332 | PROMOTER | 1.310603922 | 5.965E-21 |
| 180575 | A_68_P29242093 | F2rl2 | chr13:096798771-096798820 | INSIDE | 1.473949864 | 5.996E-21 |
| 22333 | A_68_P30584203 | Olfr168 | chr16:019442121-019442178 | DOWNSTREAM | 1.63356994 | 6.069E-21 |
| 68149 | A_68_P31131047 | Zfand3 | chr17:029729782-029729830 | PROMOTER | 1.231384101 | 6.36E-21 |
| 168076 | A_68_P26824807 | Nradd | chr9:110472805-110472849 | PROMOTER | 1.263251181 | 6.52E-21 |
| 35958 | A_68_P27272537 | Pwp2 | chr10:077578272-077578316 | INSIDE | 1.281918067 | 7.36E-21 |
| 94061 | A_68_P28915529 | Mboat1 | chr13:030142081-030142125 | PROMOTER | 1.31345852 | 7.835E-21 |
| 25078 | A_68_P21517763 | Lin7c | chr2:109687736-109687795 | PROMOTER | 1.468788667 | 8.39E-21 |
| 83168 | A_68_P31161229 | Tubb5 | chr17:035451579-035451638 | PROMOTER | 1.592161923 | 8.531E-21 |
| 83785 | A_68_P21775789 | Rbl1 | chr2:156900766-156900821 | PROMOTER | 1.500621973 | 8.897E-21 |
| 215054 | A_68_P31023608 | Rnaset2 | chr17:006830301-006830359 | INSIDE | 1.296629177 | 9.415E-21 |
| 126613 | A_68_P26342102 | Olfr873 | chr9:020050879-020050938 | INSIDE | 1.299085911 | 9.604E-21 |
| 213099 | A_68_P27478155 | Gm239 | chr10:116299660-116299719 | INSIDE | 1.605557057 | 1.057E-20 |
| 71743 | A_68_P26886369 | Cyp8b1 | chr9:121767380-121767439 | PROMOTER | 1.675647375 | 1.104E-20 |
| 10267 | A_68_P29152221 | Rhobtb3 | chr13:076410102-076410161 | PROMOTER | 1.636573782 | 1.247E-20 |
| 117826 | A_68_P25394194 | Tpp1 | chr7:105630361-105630413 | PROMOTER | 1.76082744 | 1.286E-20 |
| 227599 | A_68_P21819651 | Ube2c | chr2:164463527-164463586 | INSIDE | 1.904357558 | 1.371E-20 |
| 107815 | A_68_P31940702 | Hrasls5 | chr19:007679817-007679865 | INSIDE | 1.195951661 | 1.409E-20 |
| 180616 | A_68_P24992481 | Rtn2 | chr7:018446072-018446126 | PROMOTER | 1.435022191 | 1.691E-20 |
| 83462 | A_68_P22281932 | Lrat | chr3:082988816-082988866 | INSIDE | 1.153088545 | 2.185E-20 |
| 195616 | A_68_P31348383 | Ehd3 | chr17:073709906-073709960 | INSIDE | 1.201071541 | 2.216E-20 |
| 106331 | A_68_P27365481 | D10Ertd322e-Ube2n | chr10:094936840-094936899 | DIVERGENT_PROMOTER | 1.75064844 | 2.247E-20 |
| 122882 | A_68_P25528067 | Pstk | chr7:131163188-131163247 | INSIDE | 1.366663514 | 2.271E-20 |
| 27630 | A_68_P29247507 | Hmgcr | chr13:097769584-097769643 | INSIDE | 1.563367575 | 2.293E-20 |
| 51026 | A_68_P32569086 | Hdac8 | chrX:098708007-098708066 | PROMOTER | 1.335961119 | 2.294E-20 |
| 222631 | A_68_P29509911 | Nt5dc2 | chr14:029962584-029962634 | PROMOTER | 1.366021246 | 2.332E-20 |
| 37617 | A_68_P27534479 | D10Ertd610e | chr10:126588533-126588581 | INSIDE | 1.158103302 | 2.414E-20 |
| 185834 | A_68_P25950555 | Sfrs14 | chr8:073158412-073158463 | PROMOTER | 1.226218011 | 2.42E-20 |
| 109829 | A_68_P21489876 | Cstf3 | chr2:104392967-104393026 | INSIDE | 1.240182167 | 2.481E-20 |
| 210017 | A_68_P24449578 | Hoxa13 | chr6:052195772-052195831 | PROMOTER | 1.606144569 | 2.623E-20 |
| 34515 | A_68_P22348381 | Hist2h2aa1-Hist2h2aa1 | chr3:096330089-096330148 | DIVERGENT_PROMOTER | 1.538723933 | 2.739E-20 |
| 42015 | A_68_P26078844 | Arl2bp | chr8:097550477-097550536 | PROMOTER | 1.229753843 | 2.868E-20 |
| 176885 | A_68_P27540596 | Il23a-Usp52 | chr10:127701446-127701496 | DIVERGENT_PROMOTER | 1.336307045 | 2.941E-20 |
| 202467 | A_68_P29388244 | Pdhb | chr14:006969436-006969495 | PROMOTER | 1.262421594 | 3.048E-20 |
| 207199 | A_68_P28596592 | Pnma1 | chr12:085036332-085036376 | INSIDE | 1.147545573 | 3.34E-20 |
| 42865 | A_68_P32192474 | Tectb | chr19:055236053-055236110 | INSIDE | 1.25016471 | 3.445E-20 |
| 171333 | A_68_P31934623 | Nrxn2 | chr19:006423807-006423866 | PROMOTER | 1.328049589 | 3.621E-20 |
| 82498 | A_68_P24803338 | Slc6a13 | chr6:121263630-121263689 | PROMOTER | 1.812485906 | 3.787E-20 |
| 80646 | A_68_P29047290 | Sfxn1 | chr13:054078869-054078928 | PROMOTER | 1.875567278 | 3.859E-20 |
| 122182 | A_68_P24844322 | Klre1 | chr6:129542731-129542785 | PROMOTER | 1.458157466 | 5.156E-20 |
| 62549 | A_68_P32560521 | Kif4 | chrX:096827093-096827150 | PROMOTER | 1.415076044 | 5.415E-20 |
| 141742 | A_68_P32462874 | Bgn | chrX:069732909-069732967 | PROMOTER | 1.293244725 | 5.438E-20 |
| 170215 | A_68_P23582130 | Fosl2 | chr5:032408604-032408659 | PROMOTER | 1.568056644 | 5.502E-20 |
| 125797 | A_68_P29530893 | Ldb3 | chr14:033416297-033416353 | INSIDE | 1.305665922 | 5.613E-20 |
| 12358 | A_68_P25094559 | Car11 | chr7:045570035-045570085 | INSIDE | 1.31470522 | 5.911E-20 |
| 100103 | A_68_P27769518 | Havcr1 | chr11:046596433-046596492 | INSIDE | 1.398728909 | 6.011E-20 |
| 131162 | A_68_P20977941 | Echdc3 | chr2:006128136-006128195 | INSIDE | 1.748332503 | 6.557E-20 |
| 226430 | A_68_P29692180 | Adam28 | chr14:067608957-067609009 | INSIDE | 1.257387573 | 7.172E-20 |
| 120375 | A_68_P25262838 | Plin | chr7:079604675-079604724 | INSIDE | 1.274414299 | 7.799E-20 |
| 103389 | A_68_P30380958 | D15Wsu75e | chr15:081843167-081843211 | INSIDE | 1.402089672 | 8.205E-20 |
| 69796 | A_68_P23984904 | Ttc28 | chr5:111524720-111524767 | INSIDE | 1.123709756 | 8.24E-20 |
| 173659 | A_68_P26571787 | Itga11 | chr9:062492254-062492307 | INSIDE | 1.247759863 | 9.391E-20 |
| 100779 | A_68_P30570176 | Olfr19 | chr16:016587375-016587425 | INSIDE | 1.55184737 | 9.453E-20 |
| 226259 | A_68_P29339075 | Gzmk | chr13:114301636-114301695 | INSIDE | 1.739150556 | 9.602E-20 |
| 60709 | A_68_P29501776 | Cacna2d3 | chr14:028553775-028553827 | PROMOTER | 1.363179932 | 1.02E-19 |
| 234432 | A_68_P26977722 | Perp | chr10:018531062-018531114 | PROMOTER | 1.344497744 | 1.021E-19 |
| 200037 | A_68_P31607813 | D0H4S114 | chr18:033592982-033593038 | PROMOTER | 1.297213481 | 1.108E-19 |
| 140899 | A_68_P21004675 | Prkcq | chr2:011090075-011090119 | PROMOTER | 1.345414378 | 1.136E-19 |
| 53537 | A_68_P23590773 | Fgfr3 | chr5:034037899-034037943 | PROMOTER | 1.215269129 | 1.199E-19 |
| 136255 | A_68_P21419575 | Kbtbd4 | chr2:090706559-090706615 | INSIDE | 1.081471077 | 1.23E-19 |
| 223641 | A_68_P28066027 | Krt35 | chr11:099914263-099914311 | PROMOTER | 1.228133706 | 1.459E-19 |
| 151804 | A_68_P21740480 | Vsx1 | chr2:150383257-150383314 | PROMOTER | 1.762094678 | 1.475E-19 |
| 34000 | A_68_P31930316 | Kcnk7 | chr19:005706803-005706850 | INSIDE | 1.255123404 | 1.587E-19 |
| 141357 | A_68_P31121135 | Def6 | chr17:027936364-027936416 | INSIDE | 1.532367709 | 1.59E-19 |
| 181451 | A_68_P25949958 | Cspg3 | chr8:073048623-073048680 | INSIDE | 1.212166249 | 1.619E-19 |
| 98892 | A_68_P31929619 | Rnaseh2c | chr19:005600763-005600818 | PROMOTER | 1.553687014 | 1.71E-19 |
| 167391 | A_68_P27556390 | Tbc1d10a | chr11:004114184-004114243 | INSIDE | 1.39062739 | 1.826E-19 |
| 117576 | A_68_P30497081 | Espl1 | chr15:102124450-102124494 | INSIDE | 1.319932446 | 1.941E-19 |
| 141959 | A_68_P25367922 | Ucp3 | chr7:100345412-100345465 | PROMOTER | 1.169727425 | 1.989E-19 |
| 140763 | A_68_P30481524 | Racgap1 | chr15:099483295-099483350 | PROMOTER | 1.09027273 | 2.001E-19 |
| 184037 | A_68_P27306462 | Fhl4 | chr10:084528745-084528795 | INSIDE | 1.123062181 | 2.099E-19 |
| 49139 | A_68_P23523185 | Tmem60 | chr5:020397863-020397922 | INSIDE | 1.059233676 | 2.133E-19 |
| 23880 | A_68_P24540941 | Sftpb | chr6:072227764-072227821 | PROMOTER | 1.207870244 | 2.744E-19 |
| 135580 | A_68_P20439604 | Rab17 | chr1:092802563-092802618 | PROMOTER | 1.21043047 | 2.783E-19 |
| 214897 | A_68_P22606581 | Clca5 | chr3:145040610-145040669 | PROMOTER | 1.627076878 | 2.82E-19 |
| 118055 | A_68_P31663459 | Scgb3a2 | chr18:043889279-043889338 | PROMOTER | 1.192270479 | 3.085E-19 |
| 95007 | A_68_P25370684 | P2ry6 | chr7:100840968-100841016 | PROMOTER | 1.085283287 | 3.24E-19 |
| 137238 | A_68_P27385167 | Wdr51b | chr10:098556699-098556746 | INSIDE | 1.281858688 | 3.243E-19 |
| 214635 | A_68_P29448127 | Myoz1 | chr14:019444370-019444426 | INSIDE | 1.444515811 | 3.281E-19 |
| 5586 | A_68_P25584543 | Olfr541 | chr7:140550902-140550955 | PROMOTER | 1.062750664 | 3.728E-19 |
| 139896 | A_68_P29639604 | Tmem46 | chr14:058579933-058579983 | INSIDE | 1.291699163 | 3.886E-19 |
| 40558 | A_68_P26023302 | Tnpo2 | chr8:087922112-087922166 | PROMOTER | 1.388967343 | 3.916E-19 |
| 68524 | A_68_P20619958 | Lemd1 | chr1:134026331-134026384 | INSIDE | 1.459868237 | 4.13E-19 |
| 205767 | A_68_P20454675 | Mterfd2 | chr1:095134395-095134454 | INSIDE | 1.555989416 | 4.141E-19 |
| 88083 | A_68_P23441635 | C1qdc2 | chr4:154806822-154806874 | INSIDE | 1.453981741 | 4.355E-19 |
| 110342 | A_68_P23316686 | Sytl1 | chr4:132537765-132537809 | PROMOTER | 1.168790042 | 4.621E-19 |
| 56431 | A_68_P24307311 | Gpr37 | chr6:025637781-025637840 | INSIDE | 1.476263087 | 4.875E-19 |
| 956 | A_68_P31844954 | Ier3ip1 | chr18:077135059-077135112 | INSIDE | 1.632739257 | 4.88E-19 |
| 58370 | A_68_P23199328 | Osbpl9 | chr4:108604310-108604369 | PROMOTER | 1.197458376 | 5.061E-19 |
| 116059 | A_68_P25586781 | Trp53i5 | chr7:140960433-140960484 | PROMOTER | 0.980393517 | 5.435E-19 |
| 32603 | A_68_P26130515 | Ces2 | chr8:107734797-107734844 | PROMOTER | 1.191906085 | 6.044E-19 |
| 24504 | A_68_P31216975 | Trem2 | chr17:047812467-047812516 | INSIDE | 1.511796654 | 6.55E-19 |
| 9355 | A_68_P27996605 | Mks1 | chr11:087671890-087671943 | INSIDE | 1.731227115 | 6.65E-19 |
| 95893 | A_68_P32101931 | Plce1 | chr19:038584855-038584914 | PROMOTER | 1.415164798 | 6.874E-19 |
| 221806 | A_68_P22612842 | Mcoln2 | chr3:146089487-146089540 | INSIDE | 1.688126561 | 7.35E-19 |
| 13915 | A_68_P27271833 | Dnmt3l | chr10:077455573-077455629 | INSIDE | 1.043172227 | 7.752E-19 |
| 100038 | A_68_P24471005 | Neurod6 | chr6:055608482-055608538 | INSIDE | 1.194028668 | 8.462E-19 |
| 74520 | A_68_P29571997 | Ddhd1 | chr14:044581824-044581883 | PROMOTER | 1.499326031 | 8.601E-19 |
| 71571 | A_68_P25313085 | Sytl2 | chr7:090223546-090223604 | PROMOTER | 1.681920371 | 8.7E-19 |
| 206598 | A_68_P26467410 | Slc37a4 | chr9:044150828-044150872 | INSIDE | 1.182108646 | 9.304E-19 |
| 4628 | A_68_P31086078 | Zfp51 | chr17:021154636-021154695 | PROMOTER | 1.634285713 | 1.06E-18 |
| 158243 | A_68_P27172920 | Chst3 | chr10:059582127-059582173 | INSIDE | 1.037731859 | 1.144E-18 |
| 97199 | A_68_P29144098 | Cep72 | chr13:074532856-074532910 | PROMOTER | 1.090278033 | 1.182E-18 |
| 55470 | A_68_P24874527 | H2afj | chr6:136774748-136774801 | DOWNSTREAM | 1.054628565 | 1.228E-18 |
| 37072 | A_68_P23239805 | Hyi | chr4:117857754-117857807 | PROMOTER | 1.388519998 | 1.25E-18 |
| 49916 | A_68_P31140543 | Pknox1 | chr17:031307222-031307266 | PROMOTER | 1.363337038 | 1.28E-18 |
| 86497 | A_68_P31930198 | Map3k11 | chr19:005690697-005690741 | INSIDE | 1.502776479 | 1.32E-18 |
| 162741 | A_68_P21614160 | Prom2 | chr2:127231764-127231812 | INSIDE | 1.303196264 | 1.395E-18 |
| 188598 | A_68_P25809796 | Fgl1 | chr8:042717328-042717373 | PROMOTER | 1.144188309 | 1.509E-18 |
| 84784 | A_68_P23342119 | Akp2 | chr4:137070076-137070124 | PROMOTER | 1.163549123 | 1.57E-18 |
| 229550 | A_68_P21585727 | Duoxa1 | chr2:122010506-122010551 | PROMOTER | 0.945089953 | 1.589E-18 |
| 118721 | A_68_P24533033 | Rpia | chr6:070723752-070723806 | PROMOTER | 1.201025462 | 1.651E-18 |
| 135899 | A_68_P20085294 | Rims1 | chr1:022764394-022764453 | PROMOTER | 1.166109991 | 1.718E-18 |
| 153924 | A_68_P30358263 | Rabl4 | chr15:078002089-078002148 | PROMOTER | 1.030315601 | 1.785E-18 |
| 222736 | A_68_P27932299 | Abr | chr11:076288950-076289006 | INSIDE | 0.971549422 | 1.915E-18 |
| 104902 | A_68_P30678092 | Hcls1 | chr16:036848858-036848917 | PROMOTER | 1.176372214 | 2.036E-18 |
| 191143 | A_68_P23365093 | Slc25a34 | chr4:140899954-140899998 | PROMOTER | 1.39697901 | 2.145E-18 |
| 207508 | A_68_P25269255 | Nmb | chr7:080776907-080776951 | INSIDE | 0.983707172 | 2.189E-18 |
| 217296 | A_68_P22038051 | Pik3ca | chr3:032629205-032629264 | INSIDE | 1.411960319 | 2.301E-18 |
| 28505 | A_68_P22663497 | Tyw3 | chr3:154529969-154530028 | INSIDE | 1.173544852 | 2.356E-18 |
| 35641 | A_68_P31619319 | Slc23a1 | chr18:035752537-035752581 | INSIDE | 1.117677928 | 2.396E-18 |
| 71052 | A_68_P21635733 | Adra1d | chr2:131257649-131257702 | PROMOTER | 1.25962958 | 2.695E-18 |
| 24401 | A_68_P21601141 | Dut | chr2:124935701-124935760 | PROMOTER | 1.197315031 | 2.851E-18 |
| 133933 | A_68_P26184239 | Wwox | chr8:117325816-117325860 | INSIDE | 1.009154027 | 2.956E-18 |
| 287 | A_68_P22165675 | Pfn2 | chr3:057939657-057939716 | PROMOTER | 1.614899316 | 3.02E-18 |
| 193126 | A_68_P23239400 | Ptprf | chr4:117777847-117777891 | INSIDE | 1.097571475 | 3.106E-18 |
| 115842 | A_68_P28340223 | Atxn7l4 | chr12:033730866-033730925 | PROMOTER | 1.610105457 | 3.27E-18 |
| 173122 | A_68_P29024500 | Fgd3 | chr13:049321518-049321569 | PROMOTER | 1.105278486 | 3.292E-18 |
| 213199 | A_68_P31963196 | Olfr1450 | chr19:013019971-013020030 | PROMOTER | 1.404831979 | 3.31E-18 |
| 7913 | A_68_P20782638 | Prrx1 | chr1:165149775-165149834 | INSIDE | 1.03398185 | 3.655E-18 |
| 227224 | A_68_P30422450 | Mlc1-Mov10l1 | chr15:088809524-088809583 | DIVERGENT_PROMOTER | 1.151868737 | 3.748E-18 |
| 135983 | A_68_P28710212 | Bdkrb1 | chr12:106006215-106006271 | INSIDE | 1.359646296 | 3.797E-18 |
| 213788 | A_68_P31214196 | Tcfeb | chr17:047243532-047243581 | PROMOTER | 0.937831061 | 4.13E-18 |
| 101478 | A_68_P25729970 | D8Ertd354e | chr8:027444134-027444193 | PROMOTER | 0.942557301 | 4.32E-18 |
| 179104 | A_68_P23086991 | Mllt3 | chr4:087280767-087280826 | PROMOTER | 1.218196432 | 4.775E-18 |
| 189987 | A_68_P27536345 | Shmt2 | chr10:126923282-126923326 | INSIDE | 1.048604728 | 4.893E-18 |
| 32404 | A_68_P25392938 | Smpd1 | chr7:105429536-105429581 | INSIDE | 1.010711235 | 4.918E-18 |
| 25109 | A_68_P31424473 | Msh2 | chr17:087582981-087583033 | INSIDE | 0.996651617 | 4.921E-18 |
| 15813 | A_68_P24430639 | Gpnmb | chr6:048965613-048965672 | PROMOTER | 1.763490339 | 4.95E-18 |
| 95949 | A_68_P23253161 | Nfyc | chr4:120264893-120264947 | INSIDE | 1.582399057 | 5.35E-18 |
| 63782 | A_68_P27288406 | Matk | chr10:080663591-080663645 | INSIDE | 0.923253338 | 5.825E-18 |
| 93956 | A_68_P32465318 | Avpr2 | chrX:070146120-070146165 | INSIDE | 1.364836693 | 5.9E-18 |
| 38009 | A_68_P21072630 | Fbxw5 | chr2:025326064-025326116 | INSIDE | 0.974148947 | 6.343E-18 |
| 59916 | A_68_P29868209 | Ednrb | chr14:102731219-102731275 | PROMOTER | 1.126714108 | 7.151E-18 |
| 211397 | A_68_P30668290 | Adcy5 | chr16:035077525-035077569 | INSIDE | 1.067599576 | 7.604E-18 |
| 104151 | A_68_P28744621 | Eif5 | chr12:111980752-111980807 | PROMOTER | 1.310127217 | 8.353E-18 |
| 195735 | A_68_P25095706 | Syngr4 | chr7:045761530-045761574 | INSIDE | 1.074266701 | 9.004E-18 |
| 40074 | A_68_P32104667 | Cyp2c55 | chr19:039068227-039068281 | PROMOTER | 1.286737705 | 9.102E-18 |
| 75652 | A_68_P28932525 | Serpinb9b | chr13:033033771-033033830 | PROMOTER | 1.205168725 | 9.347E-18 |
| 171509 | A_68_P27278714 | Fgf22 | chr10:079158598-079158648 | INSIDE | 1.40112281 | 9.5E-18 |
| 86282 | A_68_P21774535 | Ndrg3 | chr2:156683283-156683327 | INSIDE | 1.127887636 | 9.8E-18 |
| 125514 | A_68_P32459839 | Nsdhl | chrX:069173755-069173811 | INSIDE | 1.070719053 | 1.086E-17 |
| 140040 | A_68_P25100418 | Sergef | chr7:046512022-046512079 | PROMOTER | 1.308639665 | 1.105E-17 |
| 72889 | A_68_P27911493 | Mybbp1a | chr11:072259500-072259559 | INSIDE | 0.919135316 | 1.113E-17 |
| 114563 | A_68_P28832917 | Ero1lb | chr13:012621663-012621722 | INSIDE | 0.940930587 | 1.129E-17 |
| 5190 | A_68_P25065983 | Plekhf1 | chr7:037937876-037937935 | PROMOTER | 1.000201071 | 1.199E-17 |
| 225099 | A_68_P31126960 | Ppil1 | chr17:028990075-028990134 | INSIDE | 1.075057007 | 1.207E-17 |
| 78879 | A_68_P30293055 | Tg | chr15:066498306-066498365 | PROMOTER | 1.566883741 | 1.22E-17 |
| 135300 | A_68_P27258626 | Gstt3 | chr10:075227175-075227219 | PROMOTER | 1.34095063 | 1.245E-17 |
| 216350 | A_68_P27956338 | Myo1d | chr11:080596601-080596656 | PROMOTER | 0.93991037 | 1.309E-17 |
| 122838 | A_68_P31139411 | Pde9a | chr17:031113581-031113625 | PROMOTER | 1.357706959 | 1.32E-17 |
| 130463 | A_68_P29054279 | Nsd1 | chr13:055325968-055326027 | INSIDE | 0.929859642 | 1.35E-17 |
| 167787 | A_68_P21079743 | Egfl7 | chr2:026401933-026401983 | PROMOTER | 1.278322328 | 1.545E-17 |
| 207478 | A_68_P24629852 | V1rb10 | chr6:089708198-089708253 | PROMOTER | 1.347842068 | 1.61E-17 |
| 76661 | A_68_P26473364 | Fxyd2 | chr9:045153440-045153484 | PROMOTER | 0.984705213 | 1.626E-17 |
| 117398 | A_68_P21073846 | Lcn13 | chr2:025522562-025522612 | INSIDE | 1.589824616 | 1.655E-17 |
| 87011 | A_68_P31098767 | Slc9a3r2 | chr17:024378642-024378701 | PROMOTER | 1.016051124 | 1.756E-17 |
| 148232 | A_68_P25782121 | Cldn23 | chr8:037298880-037298935 | PROMOTER | 1.35654189 | 1.835E-17 |
| 122055 | A_68_P22265837 | Tmem144 | chr3:079933796-079933855 | PROMOTER | 1.505350333 | 1.84E-17 |
| 95443 | A_68_P32467386 | Atp6ap1 | chrX:070556614-070556671 | INSIDE | 0.86433887 | 1.85E-17 |
| 182386 | A_68_P28902659 | Prlpc2 | chr13:027573025-027573084 | PROMOTER | 0.924381761 | 1.883E-17 |
| 96898 | A_68_P27539603 | Baz2a | chr10:127513411-127513470 | INSIDE | 2.089316946 | 1.925E-17 |
| 90863 | A_68_P30403536 | Trmu | chr15:085706615-085706660 | PROMOTER | 1.016292715 | 1.97E-17 |
| 89473 | A_68_P31212633 | Tbn | chr17:046968027-046968074 | PROMOTER | 0.873307013 | 1.973E-17 |
| 46744 | A_68_P21350852 | Osbpl6 | chr2:076351108-076351167 | INSIDE | 1.23224685 | 2.07E-17 |
| 103652 | A_68_P23621621 | Wdr1 | chr5:038851762-038851821 | PROMOTER | 1.15996271 | 2.19E-17 |
| 48696 | A_68_P28603604 | Nek9 | chr12:086227698-086227757 | INSIDE | 0.882641953 | 2.236E-17 |
| 14147 | A_68_P26238511 | Pcoln3 | chr8:126095970-126096029 | INSIDE | 1.515842261 | 2.255E-17 |
| 203892 | A_68_P28957982 | Ssr1 | chr13:038002887-038002942 | PROMOTER | 1.329802755 | 2.285E-17 |
| 71353 | A_68_P26620444 | Aqp9 | chr9:070964470-070964529 | PROMOTER | 1.260638801 | 2.344E-17 |
| 236568 | A_68_P22348199 | Hist2h2ab | chr3:096302409-096302461 | PROMOTER | 0.886490442 | 2.6E-17 |
| 18575 | A_68_P21833112 | Ddx27 | chr2:166702400-166702459 | PROMOTER | 0.918157498 | 2.672E-17 |
| 134977 | A_68_P22412725 | Sars | chr3:108572694-108572753 | INSIDE | 0.910375827 | 2.71E-17 |
| 206269 | A_68_P32752139 | Rragb | chrX:148478871-148478930 | PROMOTER | 1.040316191 | 2.822E-17 |
| 80619 | A_68_P31123086 | Clps | chr17:028286021-028286070 | INSIDE | 0.948426077 | 2.834E-17 |
| 146304 | A_68_P29262721 | Ptcd2 | chr13:100441113-100441165 | INSIDE | 1.271233935 | 3.03E-17 |
| 108095 | A_68_P20632275 | Adora1 | chr1:136055423-136055481 | PROMOTER | 0.862255862 | 3.17E-17 |
| 221336 | A_68_P28535277 | Six1 | chr12:073965881-073965931 | PROMOTER | 0.942308689 | 3.187E-17 |
| 9153 | A_68_P28118819 | Slc16a6 | chr11:109293183-109293237 | PROMOTER | 1.072109906 | 3.295E-17 |
| 184108 | A_68_P32142333 | Pitx3-Gbf1 | chr19:046205044-046205103 | DIVERGENT_PROMOTER | 0.927796101 | 3.297E-17 |
| 62603 | A_68_P29678841 | Stmn4 | chr14:065298462-065298510 | INSIDE | 1.427301451 | 3.35E-17 |
| 156915 | A_68_P28242319 | D12Ertd553e | chr12:012297408-012297467 | PROMOTER | 1.500525045 | 3.36E-17 |
| 50052 | A_68_P27969061 | Unc45b | chr11:082728718-082728775 | INSIDE | 1.246512959 | 3.535E-17 |
| 55527 | A_68_P27278028 | Gm1157 | chr10:079047357-079047416 | INSIDE | 1.022259687 | 3.617E-17 |
| 31591 | A_68_P23814373 | Srd5a2l | chr5:077216732-077216784 | INSIDE | 1.35124882 | 3.815E-17 |
| 151066 | A_68_P32721216 | Lhfpl1 | chrX:140593104-140593156 | INSIDE | 0.936779289 | 3.86E-17 |
| 134242 | A_68_P22970060 | AI597013 | chr4:062862986-062863045 | INSIDE | 1.211633204 | 4.11E-17 |
| 59752 | A_68_P29448673 | Ndst2 | chr14:019518612-019518659 | INSIDE | 1.37961119 | 4.125E-17 |
| 62036 | A_68_P31061556 | Thbs2 | chr17:014431512-014431556 | PROMOTER | 1.317133174 | 4.185E-17 |
| 66493 | A_68_P32145766 | Cnnm2 | chr19:046816217-046816268 | INSIDE | 0.872961236 | 4.191E-17 |
| 204780 | A_68_P31152916 | H2-Ab1 | chr17:033868381-033868440 | PROMOTER | 1.158712783 | 4.365E-17 |
| 86199 | A_68_P21482974 | BC016548 | chr2:103191505-103191558 | PROMOTER | 0.941150461 | 4.541E-17 |
| 79603 | A_68_P30474338 | Olfr283 | chr15:098212242-098212301 | PROMOTER | 1.180812112 | 4.694E-17 |
| 38567 | A_68_P31259492 | Pspn | chr17:056684873-056684924 | INSIDE | 1.613368214 | 4.7E-17 |
| 41048 | A_68_P32116938 | Slit1 | chr19:041795058-041795109 | INSIDE | 1.046207507 | 4.733E-17 |
| 47513 | A_68_P32766339 | Phex | chrX:152763601-152763660 | PROMOTER | 0.943643828 | 4.907E-17 |
| 208195 | A_68_P23592891 | Mxd4 | chr5:034505088-034505132 | PROMOTER | 0.966652815 | 4.909E-17 |
| 18917 | A_68_P28121727 | Abca8b | chr11:109811301-109811360 | INSIDE | 1.292997206 | 5.1E-17 |
| 195283 | A_68_P24630102 | V1ra6 | chr6:089815054-089815103 | PROMOTER | 1.19519499 | 5.25E-17 |
| 215393 | A_68_P21908072 | Chrna4 | chr2:180973264-180973309 | PROMOTER | 1.278631493 | 5.45E-17 |
| 13388 | A_68_P32468554 | Olfr1325 | chrX:070848996-070849055 | DOWNSTREAM | 0.893416358 | 5.554E-17 |
| 40817 | A_68_P27970635 | Pex12-Ap2b1 | chr11:083118477-083118535 | DIVERGENT_PROMOTER | 1.27474484 | 5.6E-17 |
| 163785 | A_68_P23241833 | Olfr1341 | chr4:118203468-118203527 | PROMOTER | 0.889541677 | 5.715E-17 |
| 209275 | A_68_P32704851 | Irs4 | chrX:136969777-136969835 | INSIDE | 0.872312578 | 5.785E-17 |
| 173841 | A_68_P30579047 | D16H22S680E | chr16:018238826-018238881 | PROMOTER | 0.968035505 | 5.852E-17 |
| 231310 | A_68_P22435328 | Amy1 | chr3:113571826-113571885 | PROMOTER | 0.921299297 | 6.775E-17 |
| 163046 | A_68_P22410445 | Ampd2-Gnat2 | chr3:108216794-108216853 | DIVERGENT_PROMOTER | 0.99838955 | 6.936E-17 |
| 172072 | A_68_P24043168 | Ptpn11-Rpl6 | chr5:121461172-121461231 | DIVERGENT_PROMOTER | 0.961744832 | 7.192E-17 |
| 166061 | A_68_P20449771 | Olfr1412 | chr1:094417977-094418022 | PROMOTER | 1.253426132 | 7.3E-17 |
| 209177 | A_68_P30350094 | Recql4 | chr15:076532966-076533010 | INSIDE | 1.241268338 | 7.55E-17 |
| 72805 | A_68_P31106498 | Wdr24 | chr17:025551069-025551126 | PROMOTER | 0.914373607 | 7.91E-17 |
| 208418 | A_68_P30681024 | Gtf2e1 | chr16:037454860-037454919 | INSIDE | 1.709210328 | 7.95E-17 |
| 183365 | A_68_P20451548 | Gpc1 | chr1:094682327-094682374 | INSIDE | 1.270031112 | 8.7E-17 |
| 91454 | A_68_P27552375 | Pib5pa-Selm | chr11:003408335-003408388 | DIVERGENT_PROMOTER | 1.117646467 | 8.9E-17 |
| 88828 | A_68_P20350260 | Prkag3 | chr1:074686356-074686402 | PROMOTER | 0.869067486 | 9.29E-17 |
| 69105 | A_68_P27833640 | Hist3h2ba | chr11:058758835-058758889 | PROMOTER | 0.895467876 | 9.405E-17 |
| 63665 | A_68_P24483204 | Herc3 | chr6:058759516-058759567 | PROMOTER | 1.463496205 | 9.7E-17 |
| 164943 | A_68_P22831610 | Orc3l | chr4:034799159-034799218 | INSIDE | 1.064380163 | 9.756E-17 |
| 181636 | A_68_P25612549 | Mrgprd | chr7:145120237-145120296 | PROMOTER | 1.204718514 | 9.856E-17 |
| 28240 | A_68_P27262825 | Ftcd | chr10:076017957-076018014 | PROMOTER | 0.87417023 | 1.236E-16 |
| 21962 | A_68_P26560887 | Uaca | chr9:060587227-060587279 | PROMOTER | 1.004105497 | 1.303E-16 |
| 54750 | A_68_P31100643 | Hn1l | chr17:024690190-024690244 | PROMOTER | 1.066586687 | 1.345E-16 |
| 3218 | A_68_P31106057 | Msln | chr17:025483286-025483333 | PROMOTER | 0.867080097 | 1.381E-16 |
| 192234 | A_68_P31937328 | BC032204 | chr19:007084700-007084749 | INSIDE | 1.179021966 | 1.405E-16 |
| 57211 | A_68_P30678378 | Fbxo40 | chr16:036901766-036901825 | PROMOTER | 1.365064923 | 1.41E-16 |
| 126047 | A_68_P29057321 | Pitx1 | chr13:055843715-055843774 | PROMOTER | 1.078393402 | 1.565E-16 |
| 226803 | A_68_P21117851 | Stxbp1 | chr2:032668000-032668059 | INSIDE | 0.92363828 | 1.598E-16 |
| 234091 | A_68_P27547381 | Olfr811 | chr10:129206388-129206447 | PROMOTER | 0.910318331 | 1.668E-16 |
| 71554 | A_68_P28031637 | Wfikkn2 | chr11:094058198-094058246 | INSIDE | 0.884170551 | 1.705E-16 |
| 159749 | A_68_P23269998 | Inpp5b | chr4:124239717-124239767 | PROMOTER | 1.18337431 | 1.81E-16 |
| 104258 | A_68_P28552890 | Tex21-Mthfd1 | chr12:077174224-077174268 | DIVERGENT_PROMOTER | 0.882634716 | 1.837E-16 |
| 79227 | A_68_P32363503 | Zdhhc9 | chrX:044456643-044456702 | PROMOTER | 1.948241733 | 1.965E-16 |
| 15623 | A_68_P22615158 | Bxdc5 | chr3:146462814-146462873 | PROMOTER | 1.332125005 | 2.075E-16 |
| 87262 | A_68_P27848722 | Mapk7-Eppb9 | chr11:061317035-061317094 | DIVERGENT_PROMOTER | 1.179185058 | 2.14E-16 |
| 190659 | A_68_P25283400 | Tmc3 | chr7:083459897-083459948 | PROMOTER | 0.879421041 | 2.226E-16 |
| 76253 | A_68_P21419644 | Ptpmt1 | chr2:090717507-090717566 | INSIDE | 1.535848198 | 2.3E-16 |
| 202137 | A_68_P25511571 | Inpp5f | chr7:128403365-128403424 | INSIDE | 1.164775911 | 2.32E-16 |
| 6258 | A_68_P26132800 | Exoc3l | chr8:108184009-108184059 | INSIDE | 1.75697941 | 2.415E-16 |
| 158992 | A_68_P25724824 | Adam9 | chr8:026480362-026480421 | INSIDE | 0.895111777 | 2.675E-16 |
| 128532 | A_68_P23999944 | Sart3 | chr5:114029027-114029076 | INSIDE | 0.917647041 | 2.682E-16 |
| 23251 | A_68_P30338813 | Arc | chr15:074501483-074501540 | PROMOTER | 0.937630623 | 2.741E-16 |
| 227774 | A_68_P26953744 | Aig1 | chr10:013562472-013562531 | PROMOTER | 0.962515093 | 2.79E-16 |
| 14898 | A_68_P24619496 | Rab7 | chr6:088015009-088015068 | PROMOTER | 0.915164878 | 2.85E-16 |
| 93810 | A_68_P25584145 | Olfr538 | chr7:140427320-140427379 | DOWNSTREAM | 1.267714592 | 2.875E-16 |
| 185429 | A_68_P32790770 | Rbbp7 | chrX:158100926-158100979 | PROMOTER | 0.947222737 | 2.941E-16 |
| 97105 | A_68_P27898488 | Acadvl | chr11:069826378-069826437 | INSIDE | 0.880273509 | 3.096E-16 |
| 135004 | A_68_P30795091 | Olfr204 | chr16:059259438-059259497 | PROMOTER | 0.962725382 | 3.665E-16 |
| 223457 | A_68_P31208052 | Klc4 | chr17:046105235-046105280 | INSIDE | 1.053219618 | 3.665E-16 |
| 207672 | A_68_P30213276 | Eif3s3 | chr15:051693767-051693826 | INSIDE | 1.101161291 | 3.69E-16 |
| 64376 | A_68_P27786459 | Rnf130 | chr11:049913913-049913967 | INSIDE | 1.081274427 | 3.701E-16 |
| 145170 | A_68_P31351280 | Slc30a6 | chr17:074296582-074296635 | PROMOTER | 1.121255391 | 3.95E-16 |
| 62737 | A_68_P31127549 | Fgd2 | chr17:029088255-029088299 | INSIDE | 0.958510444 | 4.29E-16 |
| 57282 | A_68_P30383949 | BC018285 | chr15:082448765-082448812 | PROMOTER | 0.920798873 | 4.536E-16 |
| 227206 | A_68_P30634630 | Hrasls | chr16:029125505-029125564 | PROMOTER | 0.896876087 | 4.805E-16 |
| 26840 | A_68_P29116272 | Pols | chr13:070004562-070004619 | PROMOTER | 1.046555794 | 4.965E-16 |
| 137261 | A_68_P30998355 | Bace2 | chr16:097460416-097460472 | PROMOTER | 1.221193999 | 5.05E-16 |
| 101717 | A_68_P26648638 | Hcrtr2 | chr9:076111868-076111927 | PROMOTER | 0.999536722 | 5.092E-16 |
| 194781 | A_68_P25383521 | Olfr609 | chr7:103366230-103366274 | INSIDE | 1.05202553 | 5.25E-16 |
| 16023 | A_68_P29613423 | Cdh24 | chr14:053593516-053593560 | INSIDE | 0.991639383 | 5.35E-16 |
| 28654 | A_68_P23491421 | Sema3e | chr5:014032686-014032745 | INSIDE | 0.991740347 | 5.655E-16 |
| 177766 | A_68_P24002273 | Acacb | chr5:114422711-114422768 | PROMOTER | 1.044971554 | 5.66E-16 |
| 143688 | A_68_P21065259 | Il1rn | chr2:024158569-024158628 | PROMOTER | 1.216634322 | 5.85E-16 |
| 22286 | A_68_P22311427 | Gpatc4 | chr3:088125339-088125392 | PROMOTER | 0.883642327 | 6.224E-16 |
| 70197 | A_68_P20839938 | Mnda | chr1:175748494-175748552 | INSIDE | 0.86309957 | 6.306E-16 |
| 12348 | A_68_P27943747 | Og9x | chr11:078322144-078322203 | DOWNSTREAM | 0.960982527 | 6.486E-16 |
| 113873 | A_68_P31422215 | Ttc7 | chr17:087191870-087191919 | INSIDE | 1.065412244 | 6.605E-16 |
| 41082 | A_68_P26313285 | Mtmr2 | chr9:013498193-013498252 | PROMOTER | 0.952989824 | 6.825E-16 |
| 123443 | A_68_P22965388 | Rgs3 | chr4:062101371-062101429 | PROMOTER | 1.06883305 | 7.05E-16 |
| 63044 | A_68_P22886654 | Tmod1 | chr4:046080929-046080988 | PROMOTER | 0.909698882 | 7.082E-16 |
| 166029 | A_68_P22324922 | Slc27a3 | chr3:090473485-090473529 | INSIDE | 1.061353676 | 7.25E-16 |
| 22517 | A_68_P30340803 | Ly6c | chr15:074874470-074874519 | INSIDE | 1.033089335 | 7.815E-16 |
| 43004 | A_68_P27920030 | Olfr411 | chr11:074169013-074169072 | PROMOTER | 1.284517224 | 8.205E-16 |
| 35620 | A_68_P30362261 | Mfng | chr15:078602129-078602173 | PROMOTER | 0.961594144 | 8.35E-16 |
| 70271 | A_68_P27562427 | Kremen1 | chr11:005165695-005165754 | PROMOTER | 0.974022876 | 9.1E-16 |
| 13435 | A_68_P31123043 | Gm749 | chr17:028278477-028278534 | INSIDE | 0.951781172 | 9.15E-16 |
| 180575 | A_68_P22018667 | Slc2a2 | chr3:028890308-028890367 | INSIDE | 1.530272455 | 9.152E-16 |
| 147068 | A_68_P32260281 | Bcor | chrX:011238099-011238143 | PROMOTER | 1.040012867 | 9.3E-16 |
| 155618 | A_68_P28533647 | Ppm1a | chr12:073680462-073680506 | INSIDE | 0.919479658 | 9.45E-16 |
| 155662 | A_68_P25386219 | Olfr639 | chr7:103893828-103893887 | PROMOTER | 0.970709426 | 1.066E-15 |
| 36489 | A_68_P30600203 | Dnajb11 | chr16:022774211-022774268 | INSIDE | 1.013296982 | 1.08E-15 |
| 27271 | A_68_P28152818 | Ush1g | chr11:115143386-115143431 | PROMOTER | 1.439825897 | 1.155E-15 |
| 136276 | A_68_P20600415 | Dars | chr1:130246047-130246106 | PROMOTER | 0.952670219 | 1.193E-15 |
| 160819 | A_68_P24636888 | Nup210 | chr6:091080446-091080505 | INSIDE | 1.253230702 | 1.331E-15 |
| 119617 | A_68_P23081866 | Asah3l | chr4:086340765-086340824 | PROMOTER | 0.915867019 | 1.415E-15 |
| 34284 | A_68_P31947774 | Best1 | chr19:010065209-010065257 | PROMOTER | 1.158495132 | 1.465E-15 |
| 104971 | A_68_P25089845 | Ptov1 | chr7:044741462-044741516 | PROMOTER | 1.412724575 | 1.48E-15 |
| 20683 | A_68_P28706992 | Glrx5 | chr12:105429771-105429830 | PROMOTER | 1.732810549 | 1.545E-15 |
| 51156 | A_68_P25470610 | Eef2k | chr7:120630970-120631029 | PROMOTER | 1.009973456 | 1.55E-15 |
| 161704 | A_68_P27707626 | Slit3 | chr11:035460998-035461057 | INSIDE | 1.650586047 | 1.62E-15 |
| 64271 | A_68_P24024063 | Tesc | chr5:118287301-118287347 | PROMOTER | 1.152839522 | 1.67E-15 |
| 23746 | A_68_P23594772 | Tnip2-Sh3bp2 | chr5:034837301-034837353 | DIVERGENT_PROMOTER | 1.108917134 | 1.67E-15 |
| 238514 | A_68_P29961725 | EG328479 | chr14:121584840-121584896 | INSIDE | 0.963285441 | 1.68E-15 |
| 176847 | A_68_P30588870 | Eif4g1 | chr16:020588796-020588855 | INSIDE | 1.005688149 | 1.75E-15 |
| 111299 | A_68_P24989923 | Psg19 | chr7:017955019-017955070 | INSIDE | 1.142828997 | 1.78E-15 |
| 133882 | A_68_P23979121 | P2rx2 | chr5:110581453-110581503 | INSIDE | 0.892318879 | 1.84E-15 |
| 93361 | A_68_P20153589 | Inpp4a | chr1:037315752-037315797 | INSIDE | 0.974787597 | 1.9E-15 |
| 46499 | A_68_P21752984 | Hck | chr2:152796593-152796652 | PROMOTER | 1.20072353 | 1.91E-15 |
| 222346 | A_68_P21867072 | Rae1 | chr2:172644610-172644663 | INSIDE | 1.030324722 | 2.095E-15 |
| 239832 | A_68_P26819114 | Fbxw14 | chr9:109148820-109148879 | PROMOTER | 0.867334569 | 2.135E-15 |
| 71181 | A_68_P32684303 | Esx1 | chrX:132469053-132469112 | PROMOTER | 0.974598919 | 2.182E-15 |
| 91252 | A_68_P25035738 | Apbh | chr7:030999525-030999579 | INSIDE | 1.524279312 | 2.21E-15 |
| 3904 | A_68_P28066195 | Krt13 | chr11:099935867-099935926 | INSIDE | 1.023816334 | 2.212E-15 |
| 155641 | A_68_P28074570 | Nbr1 | chr11:101372791-101372838 | INSIDE | 0.938707649 | 2.235E-15 |
| 39122 | A_68_P32572438 | Chic1 | chrX:099558432-099558489 | PROMOTER | 1.240442704 | 2.255E-15 |
| 216153 | A_68_P24851630 | Tas2r106 | chr6:131643559-131643618 | DOWNSTREAM | 1.229017378 | 2.265E-15 |
| 11844 | A_68_P31099174 | Rnf151 | chr17:024444054-024444099 | INSIDE | 1.01597138 | 2.545E-15 |
| 160905 | A_68_P26814371 | Usp4 | chr9:108198590-108198634 | PROMOTER | 1.096171553 | 3.155E-15 |
| 177125 | A_68_P28143878 | Cdc42ep4 | chr11:113571766-113571816 | PROMOTER | 0.8878111 | 3.268E-15 |
| 124112 | A_68_P24605763 | Alms1 | chr6:085551973-085552032 | PROMOTER | 0.895817691 | 3.305E-15 |
| 68582 | A_68_P31017292 | Zdhhc14 | chr17:005448694-005448741 | PROMOTER | 0.869424898 | 3.392E-15 |
| 116821 | A_68_P20875126 | EG277333 | chr1:182169851-182169898 | PROMOTER | 1.450454021 | 3.47E-15 |
| 182705 | A_68_P29606338 | Olfr1510 | chr14:051333101-051333160 | PROMOTER | 1.754457618 | 3.625E-15 |
| 18057 | A_68_P28538309 | Prkch | chr12:074502623-074502676 | PROMOTER | 0.919793501 | 3.629E-15 |
| 179910 | A_68_P26543690 | Clk3 | chr9:057564569-057564620 | PROMOTER | 0.975817079 | 3.645E-15 |
| 229900 | A_68_P28314286 | Rnaseh1 | chr12:029235435-029235494 | PROMOTER | 1.486878488 | 3.645E-15 |
| 101387 | A_68_P25430335 | Pth | chr7:113181341-113181400 | PROMOTER | 1.186337002 | 3.802E-15 |
| 177055 | A_68_P21102158 | Lrrc8a | chr2:030084320-030084369 | INSIDE | 1.127619284 | 3.825E-15 |
| 112973 | A_68_P31202043 | Slc29a1 | chr17:045057107-045057158 | PROMOTER | 1.181770249 | 4.055E-15 |
| 189384 | A_68_P23188550 | Cdcp2 | chr4:106592026-106592070 | PROMOTER | 1.195622006 | 4.41E-15 |
| 71054 | A_68_P27915085 | Itgae | chr11:072902292-072902351 | PROMOTER | 1.292286247 | 4.505E-15 |
| 29607 | A_68_P26494620 | Htr3a | chr9:048667687-048667746 | PROMOTER | 0.888961308 | 4.808E-15 |
| 168149 | A_68_P23325530 | Tmem50a-D4Wsu53e | chr4:134189093-134189152 | DIVERGENT_PROMOTER | 1.337505449 | 4.833E-15 |
| 209599 | A_68_P27194560 | Lrrtm3 | chr10:063490287-063490346 | PROMOTER | 0.918680763 | 4.843E-15 |
| 214631 | A_68_P22399848 | Adora3 | chr3:106029888-106029947 | PROMOTER | 0.925518415 | 4.92E-15 |
| 81235 | A_68_P31564361 | AW554918 | chr18:025315063-025315122 | INSIDE | 1.223100008 | 5.25E-15 |
| 8098 | A_68_P20156492 | Mrpl30 | chr1:037840385-037840438 | INSIDE | 1.151802548 | 5.45E-15 |
| 66554 | A_68_P31965935 | Olfr1499 | chr19:013880706-013880765 | DOWNSTREAM | 1.10226571 | 5.45E-15 |
| 216756 | A_68_P24541369 | Vamp5 | chr6:072301108-072301158 | INSIDE | 1.0657978 | 5.45E-15 |
| 207334 | A_68_P31094809 | Prss22 | chr17:023727005-023727058 | PROMOTER | 1.178024137 | 5.55E-15 |
| 177896 | A_68_P21118738 | Garnl3 | chr2:032808597-032808654 | INSIDE | 0.92051055 | 5.767E-15 |
| 91464 | A_68_P27552223 | Pla2g3 | chr11:003385962-003386013 | PROMOTER | 1.161052399 | 6.35E-15 |
| 58593 | A_68_P31921661 | Tbc1d10c-Ppp1ca | chr19:004191238-004191297 | DIVERGENT_PROMOTER | 1.058216456 | 6.36E-15 |
| 219921 | A_68_P23231151 | Gloxd1 | chr4:116323926-116323976 | PROMOTER | 1.422427311 | 6.65E-15 |
| 122813 | A_68_P22659852 | Acadm | chr3:153885152-153885211 | PROMOTER | 1.065494744 | 6.9E-15 |
| 174113 | A_68_P31731178 | Rnuxa | chr18:056711142-056711201 | INSIDE | 1.348324016 | 6.94E-15 |
| 19482 | A_68_P31926397 | Slc29a2 | chr19:005025698-005025742 | PROMOTER | 0.879627897 | 8.05E-15 |
| 66092 | A_68_P23225280 | Cyp4b1 | chr4:115143527-115143581 | INSIDE | 1.282347734 | 8.45E-15 |
| 86341 | A_68_P23394446 | Frap1 | chr4:147293781-147293840 | INSIDE | 1.158235373 | 8.5E-15 |
| 3265 | A_68_P30437764 | Muc19 | chr15:091663674-091663733 | PROMOTER | 1.293526601 | 8.55E-15 |
| 133039 | A_68_P23435663 | Plcl4 | chr4:153854781-153854826 | PROMOTER | 1.299178648 | 8.65E-15 |
| 92067 | A_68_P28233106 | Nt5c1b | chr12:010396554-010396613 | INSIDE | 1.382296674 | 8.65E-15 |
| 30344 | A_68_P29625874 | Gja3 | chr14:056013116-056013175 | PROMOTER | 0.920265287 | 9.66E-15 |
| 216469 | A_68_P24791577 | Dcp1b | chr6:119136695-119136748 | PROMOTER | 1.218473887 | 1.07E-14 |
| 155745 | A_68_P26782312 | Ryk | chr9:102695545-102695599 | INSIDE | 0.883470745 | 1.07E-14 |
| 59039 | A_68_P20441686 | Espnl | chr1:093153655-093153708 | INSIDE | 1.255929238 | 1.085E-14 |
| 221560 | A_68_P28791477 | Akr1c18 | chr13:004150817-004150876 | PROMOTER | 1.058266044 | 1.13E-14 |
| 26923 | A_68_P30225981 | Tnfrsf11b | chr15:054108294-054108338 | INSIDE | 0.965014522 | 1.185E-14 |
| 195190 | A_68_P30176288 | Ttc35 | chr15:043309031-043309090 | INSIDE | 0.938107336 | 1.36E-14 |
| 220086 | A_68_P24394919 | Trpv6 | chr6:041567228-041567284 | PROMOTER | 0.958542501 | 1.455E-14 |
| 74687 | A_68_P25581298 | Kndc1 | chr7:139790711-139790764 | INSIDE | 1.401613195 | 1.475E-14 |
| 51739 | A_68_P29034473 | Sema4d | chr13:051809357-051809416 | PROMOTER | 1.130386957 | 1.555E-14 |
| 126677 | A_68_P21819611 | Dnttip1 | chr2:164456498-164456545 | INSIDE | 0.908173898 | 1.585E-14 |
| 2488 | A_68_P31251166 | St6gal2 | chr17:054922527-054922586 | INSIDE | 0.875569323 | 1.615E-14 |
| 198681 | A_68_P24793281 | Wnt5b | chr6:119430577-119430636 | INSIDE | 0.883410345 | 1.643E-14 |
| 149034 | A_68_P28776165 | Cdca7l | chr12:118283635-118283694 | PROMOTER | 1.142625709 | 1.644E-14 |
| 179558 | A_68_P24596113 | Zfml | chr6:083881356-083881412 | INSIDE | 0.891869396 | 1.711E-14 |
| 198810 | A_68_P30458556 | Nell2 | chr15:095353884-095353943 | INSIDE | 1.592325057 | 1.8E-14 |
| 170650 | A_68_P21909291 | BC051628 | chr2:181151854-181151913 | INSIDE | 1.233027866 | 1.96E-14 |
| 144267 | A_68_P30603546 | Masp1 | chr16:023437349-023437405 | PROMOTER | 0.877651223 | 1.98E-14 |
| 2892 | A_68_P23428207 | BC046331 | chr4:152824024-152824068 | INSIDE | 1.201700542 | 2.05E-14 |
| 2487 | A_68_P24127799 | Pilra | chr5:138064685-138064741 | INSIDE | 1.080652123 | 2.205E-14 |
| 194445 | A_68_P23798714 | Sgcb | chr5:073914683-073914735 | INSIDE | 1.066121781 | 2.275E-14 |
| 111058 | A_68_P29006688 | Dynlt1 | chr17:006414302-006414253 | INSIDE | 1.013197642 | 2.305E-14 |
| 218822 | A_68_P22531302 | Hadh | chr3:131261509-131261568 | PROMOTER | 1.295488516 | 2.525E-14 |
| 101961 | A_68_P26398426 | Zbtb44 | chr9:030817442-030817501 | INSIDE | 1.104449042 | 2.95E-14 |
| 231841 | A_68_P24817185 | Usp5 | chr6:124791978-124792026 | INSIDE | 1.086748083 | 3.1E-14 |
| 175670 | A_68_P31943150 | Slc3a2 | chr19:008780044-008780097 | INSIDE | 1.553967268 | 3.355E-14 |
| 224876 | A_68_P28034253 | Xylt2 | chr11:094484946-094485001 | INSIDE | 1.158315254 | 3.54E-14 |
| 189701 | A_68_P30793605 | Olfr177 | chr16:058816001-058816060 | PROMOTER | 0.983061685 | 3.56E-14 |
| 73427 | A_68_P21527938 | Nola3 | chr2:112062927-112062984 | INSIDE | 0.913974385 | 3.68E-14 |
| 169575 | A_68_P22346174 | BC028528 | chr3:095970178-095970237 | INSIDE | 1.50760295 | 3.81E-14 |
| 17374 | A_68_P32121677 | D19Ertd386e | chr19:042570536-042570588 | PROMOTER | 1.006211652 | 3.82E-14 |
| 9994 | A_68_P30385388 | Tcf20 | chr15:082738144-082738196 | INSIDE | 1.31643349 | 3.825E-14 |
| 9082 | A_68_P24971933 | Zfp551 | chr7:011328986-011329041 | PROMOTER | 0.860144482 | 3.965E-14 |
| 221317 | A_68_P27183533 | Col13a1 | chr10:061377834-061377893 | PROMOTER | 1.174375709 | 4.005E-14 |
| 92679 | A_68_P22859754 | Nfx1 | chr4:041155897-041155956 | PROMOTER | 0.929012528 | 4.17E-14 |
| 205076 | A_68_P31542242 | Rnf125 | chr18:021088149-021088208 | INSIDE | 0.890223155 | 4.171E-14 |
| 72754 | A_68_P31665917 | Npy6r | chr18:044397858-044397917 | INSIDE | 0.992251691 | 4.34E-14 |
| 170624 | A_68_P21941374 | Fabp4 | chr3:010189837-010189896 | INSIDE | 1.108323331 | 4.36E-14 |
| 140000 | A_68_P28990450 | Ranbp9 | chr13:043496776-043496833 | PROMOTER | 1.142255611 | 4.585E-14 |
| 24819 | A_68_P26435138 | Olfr910 | chr9:038284165-038284224 | PROMOTER | 0.952810058 | 5.382E-14 |
| 171487 | A_68_P31406968 | Lrpprc | chr17:084699428-084699487 | PROMOTER | 0.919135331 | 5.45E-14 |
| 123451 | A_68_P28513101 | Rps29 | chr12:070077092-070077151 | INSIDE | 0.899306991 | 5.575E-14 |
| 137954 | A_68_P27281269 | Midn | chr10:079549868-079549920 | PROMOTER | 0.910015744 | 5.95E-14 |
| 61326 | A_68_P28184063 | Fscn2 | chr11:120179023-120179077 | INSIDE | 1.046423737 | 6.05E-14 |
| 91113 | A_68_P31923320 | Syt12 | chr19:004475642-004475693 | INSIDE | 1.37788671 | 6.15E-14 |
| 115107 | A_68_P25258788 | Agc1 | chr7:078928152-078928209 | INSIDE | 0.968536902 | 6.25E-14 |
| 220600 | A_68_P22217036 | Il12a | chr3:068778996-068779055 | INSIDE | 0.871276281 | 6.518E-14 |
| 228867 | A_68_P27540114 | Gls2 | chr10:127612821-127612880 | INSIDE | 0.894503517 | 6.75E-14 |
| 93877 | A_68_P23231932 | Hectd3 | chr4:116496640-116496699 | INSIDE | 0.903672249 | 6.9E-14 |
| 74504 | A_68_P30267524 | Myc | chr15:061816157-061816216 | INSIDE | 1.083757317 | 7.445E-14 |
| 3487 | A_68_P30587804 | Eif2b5 | chr16:020409821-020409874 | PROMOTER | 1.162436222 | 7.45E-14 |
| 179269 | A_68_P30494514 | Krt77 | chr15:101700165-101700217 | PROMOTER | 1.509782003 | 7.7E-14 |
| 122744 | A_68_P23239399 | Ptprf | chr4:117777704-117777758 | INSIDE | 1.02589085 | 7.95E-14 |
| 116343 | A_68_P31166970 | Gabbr1 | chr17:036656074-036656133 | INSIDE | 0.988786974 | 8.25E-14 |
| 197139 | A_68_P26402587 | Barx2 | chr9:031668035-031668094 | PROMOTER | 1.089455434 | 8.5E-14 |
| 1849 | A_68_P21779879 | Gm691 | chr2:157607158-157607210 | INSIDE | 1.252471862 | 8.85E-14 |
| 95270 | A_68_P21753688 | Plagl2 | chr2:152920812-152920860 | INSIDE | 1.057913785 | 9.2E-14 |
| 83681 | A_68_P21111649 | Fibcd1 | chr2:031666569-031666613 | INSIDE | 1.062657431 | 9.8E-14 |
| 234463 | A_68_P26554624 | Pkm2 | chr9:059454201-059454250 | PROMOTER | 1.014274338 | 1.037E-13 |
| 212321 | A_68_P31955282 | Ms4a1 | chr19:011337435-011337494 | PROMOTER | 0.925830727 | 1.086E-13 |
| 209540 | A_68_P27274076 | Nnp1 | chr10:077813872-077813925 | INSIDE | 1.107341413 | 1.095E-13 |
| 110700 | A_68_P22413531 | Wdr47 | chr3:108720613-108720672 | INSIDE | 1.520231731 | 1.18E-13 |
| 74379 | A_68_P26615109 | Myo1e | chr9:070002840-070002894 | PROMOTER | 1.514543914 | 1.26E-13 |
| 235480 | A_68_P24326696 | Atp6v1f | chr6:029415002-029415052 | PROMOTER | 0.886045855 | 1.275E-13 |
| 215517 | A_68_P32321795 | Upf3b | chrX:033543224-033543283 | PROMOTER | 0.957733518 | 1.28E-13 |
| 150812 | A_68_P31488601 | Rock1 | chr18:010179838-010179897 | INSIDE | 0.88822372 | 1.296E-13 |
| 213994 | A_68_P27259083 | Mif | chr10:075305628-075305685 | PROMOTER | 1.194555975 | 1.325E-13 |
| 123101 | A_68_P21320153 | Dlx2 | chr2:071349206-071349256 | PROMOTER | 1.155347672 | 1.355E-13 |
| 191591 | A_68_P23126032 | Hook1 | chr4:095456257-095456316 | PROMOTER | 0.928140076 | 1.39E-13 |
| 6917 | A_68_P31103238 | Tpsg1 | chr17:025097651-025097695 | PROMOTER | 1.205654265 | 1.44E-13 |
| 118713 | A_68_P27997663 | Vezf1 | chr11:087886797-087886856 | INSIDE | 0.895125578 | 1.475E-13 |
| 178070 | A_68_P27548671 | Olfr826 | chr10:129586233-129586292 | PROMOTER | 0.927212603 | 1.486E-13 |
| 13357 | A_68_P21776942 | Src | chr2:157117312-157117359 | INSIDE | 1.053233784 | 1.715E-13 |
| 17145 | A_68_P25596336 | Igf2 | chr7:142465948-142466003 | INSIDE | 0.891626579 | 1.735E-13 |
| 113092 | A_68_P30493938 | Krt72 | chr15:101619597-101619648 | PROMOTER | 1.08651252 | 1.78E-13 |
| 14604 | A_68_P30947763 | Krtap13 | chr16:088639929-088639986 | INSIDE | 1.674936885 | 1.81E-13 |
| 151079 | A_68_P21853394 | Pfdn4 | chr2:170184372-170184431 | PROMOTER | 0.92673729 | 1.83E-13 |
| 154893 | A_68_P22900907 | Tmeff1 | chr4:048601649-048601705 | PROMOTER | 1.520325402 | 1.84E-13 |
| 13063 | A_68_P31138219 | Tff1 | chr17:030889902-030889950 | INSIDE | 1.53558343 | 1.85E-13 |
| 51553 | A_68_P30880692 | Stch | chr16:075654471-075654530 | PROMOTER | 0.872621787 | 1.86E-13 |
| 95226 | A_68_P20442319 | Hes6 | chr1:093241845-093241898 | DOWNSTREAM | 1.023875121 | 1.97E-13 |
| 138691 | A_68_P23328106 | Srrm1 | chr4:134629632-134629687 | PROMOTER | 1.176203522 | 2.015E-13 |
| 135881 | A_68_P28576800 | Erh | chr12:081561122-081561181 | INSIDE | 0.973101159 | 2.042E-13 |
| 135604 | A_68_P31796917 | Mc5r | chr18:068464131-068464183 | INSIDE | 1.189377336 | 2.09E-13 |
| 50996 | A_68_P29904236 | Slitrk5 | chr14:110553714-110553773 | PROMOTER | 1.133685344 | 2.159E-13 |
| 112991 | A_68_P22315051 | Rxfp4 | chr3:088738233-088738280 | INSIDE | 1.173116363 | 2.23E-13 |
| 106945 | A_68_P24090530 | Gbas | chr5:130035547-130035606 | PROMOTER | 1.182231794 | 2.29E-13 |
| 216488 | A_68_P26224538 | Mthfsd | chr8:123992888-123992942 | INSIDE | 0.967369498 | 2.42E-13 |
| 215617 | A_68_P30345297 | Mapk15 | chr15:075813375-075813419 | PROMOTER | 0.872890017 | 2.585E-13 |
| 44768 | A_68_P29330066 | Mier3 | chr13:112806438-112806497 | PROMOTER | 0.876589446 | 2.61E-13 |
| 199123 | A_68_P28052585 | Ccdc49 | chr11:097582549-097582593 | INSIDE | 0.923982809 | 2.65E-13 |
| 106827 | A_68_P23347627 | Ubxd3 | chr4:138009858-138009915 | PROMOTER | 0.892737015 | 2.69E-13 |
| 68697 | A_68_P25184071 | Tm2d3 | chr7:065566041-065566085 | PROMOTER | 0.891810495 | 2.73E-13 |
| 99843 | A_68_P26398870 | St14 | chr9:030884507-030884564 | PROMOTER | 1.109609745 | 3.16E-13 |
| 115622 | A_68_P20938870 | Dtl | chr1:193274982-193275037 | INSIDE | 0.968570757 | 3.234E-13 |
| 58085 | A_68_P30284283 | Adcy8 | chr15:064754215-064754274 | PROMOTER | 0.859901803 | 3.375E-13 |
| 194113 | A_68_P21812411 | Gdap1l1 | chr2:163125904-163125958 | PROMOTER | 1.171628425 | 3.425E-13 |
| 37593 | A_68_P21570810 | Ndufaf1 | chr2:119354647-119354695 | PROMOTER | 0.968803969 | 3.65E-13 |
| 214683 | A_68_P21767364 | Procr | chr2:155440044-155440103 | PROMOTER | 1.140073494 | 3.84E-13 |
| 47375 | A_68_P21399024 | Olfr1009 | chr2:085521818-085521877 | PROMOTER | 0.907167459 | 3.88E-13 |
| 96338 | A_68_P22366405 | Spag17 | chr3:100009391-100009446 | PROMOTER | 1.182139014 | 4.145E-13 |
| 4431 | A_68_P21633064 | Adam33 | chr2:130757068-130757127 | PROMOTER | 1.340318618 | 4.265E-13 |
| 173200 | A_68_P23270819 | Epha10 | chr4:124381591-124381650 | PROMOTER | 0.965447222 | 4.35E-13 |
| 220342 | A_68_P32082703 | Pank1 | chr19:034947584-034947637 | PROMOTER | 0.930813779 | 4.645E-13 |
| 118841 | A_68_P30999101 | Mx1 | chr16:097573055-097573109 | PROMOTER | 1.297767873 | 4.73E-13 |
| 68339 | A_68_P25266537 | Furin | chr7:080274016-080274066 | INSIDE | 0.915614036 | 5.15E-13 |
| 27802 | A_68_P23892512 | Vdp | chr5:093214327-093214386 | INSIDE | 1.585915785 | 5.2E-13 |
| 219535 | A_68_P25591639 | Tollip | chr7:141714095-141714154 | PROMOTER | 1.103873715 | 5.69E-13 |
| 213250 | A_68_P30234998 | Sntb1 | chr15:055738397-055738456 | PROMOTER | 0.872166547 | 5.75E-13 |
| 27945 | A_68_P29598118 | Olfr748 | chr14:049630671-049630730 | PROMOTER | 1.070556488 | 5.952E-13 |
| 13310 | A_68_P30180502 | Eny2 | chr15:044258921-044258980 | INSIDE | 0.908205102 | 6.357E-13 |
| 72702 | A_68_P32051841 | Jak2 | chr19:029319281-029319329 | INSIDE | 1.020467646 | 6.45E-13 |
| 166869 | A_68_P28223691 | Pum2 | chr12:008698003-008698061 | PROMOTER | 0.935781822 | 6.6E-13 |
| 46402 | A_68_P24658370 | Lrig1 | chr6:094663645-094663704 | INSIDE | 1.293084407 | 7.05E-13 |
| 24100 | A_68_P22409545 | Gstm7 | chr3:108058237-108058290 | INSIDE | 0.908260202 | 7.05E-13 |
| 221457 | A_68_P29982410 | Prkaa1 | chr15:005128329-005128388 | INSIDE | 0.925744857 | 7.11E-13 |
| 52200 | A_68_P23145509 | Foxd3 | chr4:099143988-099144047 | PROMOTER | 1.107484678 | 7.14E-13 |
| 119571 | A_68_P28772605 | Ptprn2 | chr12:117689815-117689863 | INSIDE | 1.071673667 | 7.3E-13 |
| 86382 | A_68_P29270124 | Ccnb1 | chr13:101891449-101891508 | PROMOTER | 1.252680484 | 7.3E-13 |
| 87178 | A_68_P24854158 | Tas2r120 | chr6:132621242-132621301 | PROMOTER | 0.908511976 | 7.451E-13 |
| 163875 | A_68_P21854636 | Dok5 | chr2:170419519-170419571 | PROMOTER | 1.167324918 | 7.7E-13 |
| 98426 | A_68_P20998749 | Atp5c1 | chr2:009997924-009997968 | INSIDE | 0.943432731 | 8E-13 |
| 112228 | A_68_P30495014 | Krt79 | chr15:101771831-101771884 | PROMOTER | 1.065320203 | 8.05E-13 |
| 121896 | A_68_P23577631 | Gckr | chr5:031574738-031574785 | INSIDE | 1.055937363 | 8.25E-13 |
| 192766 | A_68_P31157503 | Bat5 | chr17:034698394-034698442 | INSIDE | 0.973000754 | 8.25E-13 |
| 232060 | A_68_P32130009 | Cyp2c44 | chr19:044084426-044084483 | PROMOTER | 1.616507224 | 9.2E-13 |
| 137290 | A_68_P30294304 | Sla | chr15:066666657-066666712 | PROMOTER | 1.519046406 | 9.55E-13 |
| 127792 | A_68_P29021894 | Phf2 | chr13:048885167-048885211 | PROMOTER | 1.010666207 | 1.115E-12 |
| 84438 | A_68_P25971965 | Hmox1 | chr8:077991770-077991828 | INSIDE | 0.892045847 | 1.165E-12 |
| 230790 | A_68_P26242229 | Acta1 | chr8:126783398-126783451 | PROMOTER | 1.223418354 | 1.17E-12 |
| 186214 | A_68_P21757609 | Bpil3 | chr2:153586892-153586950 | PROMOTER | 0.882709066 | 1.206E-12 |
| 176229 | A_68_P30742748 | Retnla | chr16:048759990-048760042 | PROMOTER | 0.890249718 | 1.215E-12 |
| 181007 | A_68_P28186297 | Stra13 | chr11:120526784-120526829 | DOWNSTREAM | 0.890110179 | 1.24E-12 |
| 194821 | A_68_P28100854 | Cd79b | chr11:106130735-106130779 | INSIDE | 0.86468566 | 1.27E-12 |
| 219114 | A_68_P31160364 | Gtf2h4 | chr17:035281835-035281889 | PROMOTER | 0.876606592 | 1.288E-12 |
| 88454 | A_68_P32802226 | Glra2 | chrX:160670201-160670260 | INSIDE | 0.95847359 | 1.314E-12 |
| 180280 | A_68_P25879797 | Spata4 | chr8:056098759-056098818 | PROMOTER | 0.970492518 | 1.365E-12 |
| 234881 | A_68_P22614649 | Ctbs | chr3:146386339-146386385 | PROMOTER | 1.006265609 | 1.39E-12 |
| 223082 | A_68_P25715762 | Golga7 | chr8:024725780-024725839 | PROMOTER | 1.678066332 | 1.415E-12 |
| 91447 | A_68_P28669142 | Galc-Gpr65 | chr12:098663761-098663820 | DIVERGENT_PROMOTER | 1.58690326 | 1.465E-12 |
| 118891 | A_68_P21072941 | Edf1 | chr2:025380171-025380215 | INSIDE | 1.046421478 | 1.48E-12 |
| 199892 | A_68_P29597010 | Olfr734 | chr14:049242278-049242331 | INSIDE | 1.229515102 | 1.505E-12 |
| 66359 | A_68_P22615555 | Uox | chr3:146534340-146534399 | PROMOTER | 0.904106135 | 1.664E-12 |
| 11966 | A_68_P24331371 | Ube2h | chr6:030257993-030258052 | PROMOTER | 0.935345494 | 1.68E-12 |
| 166480 | A_68_P22818597 | Map3k7 | chr4:032289531-032289583 | PROMOTER | 0.891931456 | 1.77E-12 |
| 204448 | A_68_P25464968 | BC048390 | chr7:119481436-119481489 | INSIDE | 1.02041733 | 1.94E-12 |
| 136102 | A_68_P24925827 | Sspn | chr6:145886633-145886692 | PROMOTER | 0.914926628 | 2.096E-12 |
| 13949 | A_68_P31851427 | Slc14a1 | chr18:078290513-078290572 | PROMOTER | 1.885961428 | 2.11E-12 |
| 130148 | A_68_P21303552 | Stk39 | chr2:068278318-068278377 | PROMOTER | 0.96122304 | 2.148E-12 |
| 146699 | A_68_P28933121 | Serpinb9c | chr13:033166897-033166949 | INSIDE | 1.010334097 | 2.19E-12 |
| 186864 | A_68_P21699909 | Otor | chr2:142768141-142768194 | PROMOTER | 1.330322846 | 2.21E-12 |
| 227392 | A_68_P28081703 | Higd1b | chr11:102650523-102650570 | PROMOTER | 0.879884573 | 2.305E-12 |
| 12767 | A_68_P21404374 | Olfr1102 | chr2:086801435-086801494 | PROMOTER | 0.901847904 | 2.43E-12 |
| 28181 | A_68_P32119629 | Sfrp5 | chr19:042253548-042253607 | INSIDE | 1.178460647 | 2.542E-12 |
| 166940 | A_68_P30388440 | Pacsin2 | chr15:083264422-083264474 | PROMOTER | 0.91220385 | 2.67E-12 |
| 140961 | A_68_P31107328 | Pigq | chr17:025667563-025667618 | INSIDE | 0.904934396 | 2.785E-12 |
| 218813 | A_68_P20191538 | Tpp2 | chr1:043877311-043877370 | PROMOTER | 0.901684813 | 2.827E-12 |
| 182190 | A_68_P22308564 | Etv3 | chr3:087613736-087613787 | INSIDE | 1.04704742 | 2.995E-12 |
| 189597 | A_68_P21749781 | Defb29 | chr2:152231849-152231908 | PROMOTER | 1.4027733 | 3.015E-12 |
| 164246 | A_68_P31947864 | Rab3il1 | chr19:010081757-010081816 | PROMOTER | 1.042461581 | 3.246E-12 |
| 27837 | A_68_P23401304 | Clstn1 | chr4:148427251-148427310 | PROMOTER | 0.92544524 | 3.478E-12 |
| 190464 | A_68_P28872854 | Zfp187 | chr13:021448994-021449053 | DOWNSTREAM | 1.183497865 | 3.71E-12 |
| 203308 | A_68_P23441920 | Tnfrsf18 | chr4:154870431-154870483 | INSIDE | 0.896580849 | 3.74E-12 |
| 126648 | A_68_P25380528 | Olfr560 | chr7:102632993-102633052 | PROMOTER | 0.980886017 | 3.82E-12 |
| 207584 | A_68_P30328252 | Chrac1 | chr15:072916305-072916355 | PROMOTER | 1.031979512 | 4.085E-12 |
| 62084 | A_68_P23331215 | Cnr2 | chr4:135190842-135190901 | INSIDE | 1.004618201 | 4.23E-12 |
| 44094 | A_68_P23336226 | Ephb2 | chr4:136105521-136105580 | INSIDE | 0.881939995 | 4.385E-12 |
| 835 | A_68_P23069095 | Bnc2 | chr4:084021114-084021169 | PROMOTER | 1.184598938 | 4.625E-12 |
| 123194 | A_68_P30725580 | Btla | chr16:045142512-045142561 | PROMOTER | 0.958674987 | 4.725E-12 |
| 116725 | A_68_P23441845 | Sdf4 | chr4:154854243-154854302 | INSIDE | 1.304978907 | 4.845E-12 |
| 227102 | A_68_P23981128 | Ddx51 | chr5:110898276-110898335 | INSIDE | 0.969415885 | 4.975E-12 |
| 195543 | A_68_P25678413 | BB014433 | chr8:015045254-015045313 | INSIDE | 1.024832048 | 4.99E-12 |
| 149264 | A_68_P29329276 | Gpbp1 | chr13:112613595-112613650 | PROMOTER | 0.880794935 | 5.1E-12 |
| 227291 | A_68_P32751781 | Mageh1 | chrX:148382741-148382800 | PROMOTER | 0.990863392 | 5.25E-12 |
| 28352 | A_68_P26403979 | Grit | chr9:031955834-031955893 | PROMOTER | 1.317466087 | 5.26E-12 |
| 6361 | A_68_P30718320 | Qtrtd1 | chr16:043812716-043812775 | PROMOTER | 0.907700202 | 5.35E-12 |
| 120059 | A_68_P20634521 | Syt2 | chr1:136458492-136458540 | PROMOTER | 0.868646384 | 5.45E-12 |
| 231040 | A_68_P28330102 | Lamb1-1 | chr12:031847430-031847489 | PROMOTER | 1.269806393 | 5.85E-12 |
| 66897 | A_68_P24448662 | Hoxa1 | chr6:052087518-052087569 | INSIDE | 0.924962722 | 6.251E-12 |
| 145313 | A_68_P23599435 | Adra2c | chr5:035593133-035593191 | PROMOTER | 1.098598143 | 6.4E-12 |
| 1079 | A_68_P32564144 | Nono | chrX:097648786-097648845 | INSIDE | 0.891665056 | 6.6E-12 |
| 17213 | A_68_P24866490 | Emp1 | chr6:135323700-135323757 | PROMOTER | 0.979655217 | 7.35E-12 |
| 79977 | A_68_P24429274 | Gimap3 | chr6:048700897-048700949 | PROMOTER | 0.920001681 | 7.35E-12 |
| 67778 | A_68_P30305879 | Khdrbs3 | chr15:068754861-068754920 | PROMOTER | 0.900296575 | 7.35E-12 |
| 155285 | A_68_P20786806 | Sele | chr1:165894081-165894140 | INSIDE | 0.945167471 | 8.3E-12 |
| 189470 | A_68_P30723415 | Cd200r1 | chr16:044682411-044682469 | PROMOTER | 0.943085753 | 8.6E-12 |
| 36408 | A_68_P24535055 | Fabp1 | chr6:071125173-071125231 | PROMOTER | 0.977943944 | 8.75E-12 |
| 198875 | A_68_P21405702 | Olfr1121 | chr2:087172315-087172374 | PROMOTER | 1.040443348 | 9.074E-12 |
| 182325 | A_68_P32682750 | Plp1 | chrX:132169123-132169182 | PROMOTER | 1.02395745 | 9.75E-12 |
| 206330 | A_68_P20470053 | Slco6b1 | chr1:098832770-098832829 | PROMOTER | 1.052440609 | 1.025E-11 |
| 90015 | A_68_P26440446 | Olfr973 | chr9:039655246-039655305 | PROMOTER | 0.962995341 | 1.065E-11 |
| 183536 | A_68_P26343216 | Fbxl12 | chr9:020391018-020391077 | INSIDE | 0.87326718 | 1.1E-11 |
| 161929 | A_68_P28884729 | Scgn | chr13:024001104-024001163 | PROMOTER | 0.962379434 | 1.12E-11 |
| 22483 | A_68_P26849178 | Stt3b | chr9:115162999-115163058 | PROMOTER | 1.348724668 | 1.17E-11 |
| 227066 | A_68_P26079364 | Cx3cl1 | chr8:097659020-097659064 | PROMOTER | 1.100381094 | 1.185E-11 |
| 177402 | A_68_P22962265 | Mup5 | chr4:061323732-061323785 | PROMOTER | 1.100283556 | 1.2E-11 |
| 178190 | A_68_P31357213 | Rasgrp3 | chr17:075366681-075366740 | PROMOTER | 0.916203326 | 1.211E-11 |
| 40248 | A_68_P25379116 | Olfr545 | chr7:102373588-102373636 | PROMOTER | 0.978947492 | 1.245E-11 |
| 184397 | A_68_P23310889 | Gmeb1 | chr4:131523572-131523622 | INSIDE | 0.947099765 | 1.395E-11 |
| 118496 | A_68_P28103543 | Ccdc45 | chr11:106607038-106607097 | INSIDE | 1.008390818 | 1.45E-11 |
| 71742 | A_68_P31632697 | Pcdh1 | chr18:038337109-038337157 | PROMOTER | 0.921614419 | 1.46E-11 |
| 27783 | A_68_P23597111 | Rgs12 | chr5:035261545-035261602 | PROMOTER | 1.163381794 | 1.51E-11 |
| 228980 | A_68_P31598815 | Slc25a46 | chr18:031755682-031755741 | PROMOTER | 1.264866556 | 1.515E-11 |
| 216104 | A_68_P26144647 | Nqo1 | chr8:110297377-110297427 | PROMOTER | 0.935849695 | 1.515E-11 |
| 176551 | A_68_P24618523 | Cnbp | chr6:087818676-087818735 | PROMOTER | 0.902392393 | 1.676E-11 |
| 158747 | A_68_P27996819 | Olfr462 | chr11:087704182-087704241 | DOWNSTREAM | 0.936977468 | 1.7E-11 |
| 59912 | A_68_P21074139 | Lcn3 | chr2:025588920-025588972 | INSIDE | 0.952754995 | 1.725E-11 |
| 175557 | A_68_P32117475 | Frat2 | chr19:041899297-041899356 | INSIDE | 1.05042683 | 1.966E-11 |
| 36622 | A_68_P30427172 | Syt10 | chr15:089669894-089669953 | PROMOTER | 0.870531099 | 1.968E-11 |
| 150512 | A_68_P28547072 | Kcnh5 | chr12:076094472-076094531 | INSIDE | 0.984304046 | 2.03E-11 |
| 201256 | A_68_P29275497 | Pik3r1 | chr13:102871387-102871446 | PROMOTER | 1.196100875 | 2.03E-11 |
| 181818 | A_68_P25274349 | Tm6sf1 | chr7:081728575-081728634 | PROMOTER | 1.221998106 | 2.055E-11 |
| 130081 | A_68_P28548333 | Gphb5 | chr12:076334275-076334334 | INSIDE | 0.876211588 | 2.145E-11 |
| 121979 | A_68_P21968985 | Bhlhb5 | chr3:018241863-018241922 | PROMOTER | 1.165445423 | 2.215E-11 |
| 160948 | A_68_P32217995 | Kcnk18 | chr19:059273914-059273970 | INSIDE | 0.913147028 | 2.23E-11 |
| 146847 | A_68_P20765441 | Cacybp | chr1:162054266-162054325 | PROMOTER | 0.982745421 | 2.275E-11 |
| 62405 | A_68_P26543325 | Edc3 | chr9:057502104-057502157 | PROMOTER | 1.477027534 | 2.28E-11 |
| 122482 | A_68_P32536233 | Pdk3 | chrX:090089316-090089375 | PROMOTER | 1.033548264 | 2.33E-11 |
| 153294 | A_68_P32249046 | Sytl5 | chrX:009117005-009117064 | PROMOTER | 0.890604526 | 2.43E-11 |
| 19805 | A_68_P27614837 | AI553587 | chr11:017113286-017113345 | INSIDE | 1.081177164 | 2.46E-11 |
| 130664 | A_68_P27919158 | Olfr401 | chr11:073936863-073936922 | PROMOTER | 0.864631885 | 2.51E-11 |
| 174800 | A_68_P28030437 | Spag9 | chr11:093862690-093862749 | INSIDE | 1.618492015 | 2.535E-11 |
| 238425 | A_68_P25025708 | Spred3 | chr7:028875382-028875430 | INSIDE | 1.169248513 | 2.7E-11 |
| 97332 | A_68_P31677650 | Ticam2 | chr18:046698591-046698650 | INSIDE | 0.858472487 | 2.915E-11 |
| 14660 | A_68_P21611752 | Ncaph | chr2:126823595-126823654 | INSIDE | 1.300920365 | 3.06E-11 |
| 65467 | A_68_P32013850 | Trpm3 | chr19:022817405-022817463 | INSIDE | 1.15789262 | 3.07E-11 |
| 123504 | A_68_P30999294 | ORF9 | chr16:097659739-097659786 | INSIDE | 0.916794571 | 3.545E-11 |
| 173180 | A_68_P22317694 | Scamp3 | chr3:089266374-089266433 | INSIDE | 0.859455781 | 3.995E-11 |
| 106543 | A_68_P26465917 | Mcam | chr9:043886836-043886880 | INSIDE | 1.054184423 | 4.02E-11 |
| 212366 | A_68_P30398225 | Upk3a | chr15:084843324-084843383 | PROMOTER | 0.905065712 | 4.23E-11 |
| 142131 | A_68_P28188882 | Tex19 | chr11:120963497-120963542 | INSIDE | 0.860704137 | 4.41E-11 |
| 213996 | A_68_P26906301 | Myct1 | chr10:004754483-004754541 | PROMOTER | 0.879501352 | 4.6E-11 |
| 191860 | A_68_P31301870 | Txndc2 | chr17:065544177-065544236 | PROMOTER | 1.067968953 | 4.635E-11 |
| 200115 | A_68_P25553075 | Clrn3 | chr7:135364811-135364870 | INSIDE | 1.168806659 | 4.645E-11 |
| 34870 | A_68_P21912225 | Oprl1 | chr2:181639381-181639440 | PROMOTER | 1.109257515 | 4.695E-11 |
| 208004 | A_68_P24126384 | Actl6b | chr5:137794567-137794619 | INSIDE | 1.124194866 | 4.925E-11 |
| 154381 | A_68_P23272366 | Zc3h12a | chr4:124635596-124635642 | PROMOTER | 1.055109526 | 4.995E-11 |
| 131011 | A_68_P27505077 | BC048403 | chr10:121144726-121144785 | INSIDE | 1.246002784 | 5.5E-11 |
| 92926 | A_68_P23293619 | Trim62 | chr4:128383829-128383888 | PROMOTER | 0.967844524 | 5.61E-11 |
| 60725 | A_68_P31956968 | Ms4a6d | chr19:011677221-011677280 | PROMOTER | 1.040017125 | 5.7E-11 |
| 81468 | A_68_P27501027 | Wif1 | chr10:120437675-120437734 | INSIDE | 0.874970217 | 5.701E-11 |
| 238863 | A_68_P31540758 | Ttr | chr18:020805219-020805278 | PROMOTER | 1.479512415 | 5.95E-11 |
| 36984 | A_68_P26508170 | Pou2af1 | chr9:050961187-050961246 | PROMOTER | 1.294856903 | 6.5E-11 |
| 62846 | A_68_P21368956 | Neurod1 | chr2:079261032-079261090 | PROMOTER | 0.904648466 | 6.75E-11 |
| 9424 | A_68_P21080400 | Lcn4 | chr2:026498719-026498775 | PROMOTER | 1.28996695 | 6.9E-11 |
| 91230 | A_68_P31141138 | Cryaa | chr17:031405776-031405829 | PROMOTER | 1.349216075 | 7.05E-11 |
| 139303 | A_68_P31111062 | Ergic1 | chr17:026291544-026291595 | INSIDE | 0.906460157 | 7.7E-11 |
| 113318 | A_68_P31083843 | V1re3 | chr17:020481586-020481645 | INSIDE | 0.863283241 | 7.75E-11 |
| 50263 | A_68_P23417023 | Acot7 | chr4:151023949-151023996 | INSIDE | 0.985727022 | 7.95E-11 |
| 191808 | A_68_P21568386 | Dnajc17 | chr2:118899507-118899566 | INSIDE | 0.899890231 | 8.05E-11 |
| 167927 | A_68_P27217190 | Arid5b | chr10:067678609-067678662 | PROMOTER | 1.042330977 | 8.2E-11 |
| 68858 | A_68_P32557867 | Eda | chrX:096180862-096180921 | INSIDE | 0.949797624 | 8.85E-11 |
| 17670 | A_68_P31490847 | Snrpd1 | chr18:010616627-010616686 | PROMOTER | 0.872734301 | 8.918E-11 |
| 211346 | A_68_P30574176 | Lztr1 | chr16:017423694-017423753 | INSIDE | 0.925058373 | 9.101E-11 |
| 220405 | A_68_P21920283 | Zfhx4 | chr3:005225463-005225517 | INSIDE | 1.437455772 | 9.45E-11 |
| 69985 | A_68_P21562953 | Fsip1 | chr2:117950981-117951040 | PROMOTER | 0.989879381 | 9.85E-11 |
| 107154 | A_68_P20512335 | Serpinb11 | chr1:109189483-109189542 | PROMOTER | 0.97156969 | 1.15E-10 |
| 99931 | A_68_P23296648 | Zbtb8 | chr4:128943538-128943588 | PROMOTER | 0.879963437 | 1.16E-10 |
| 96 | A_68_P29001547 | Mylip | chr13:045396492-045396541 | PROMOTER | 0.905204653 | 1.19E-10 |
| 170091 | A_68_P20836735 | Olfr1404 | chr1:175049052-175049110 | PROMOTER | 0.90128755 | 1.285E-10 |
| 53664 | A_68_P32479513 | Prkx | chrX:074049363-074049407 | PROMOTER | 0.951893974 | 1.315E-10 |
| 31066 | A_68_P24854492 | Tas2r124 | chr6:132718015-132718074 | PROMOTER | 0.899204452 | 1.341E-10 |
| 177821 | A_68_P31190589 | Slc25a27 | chr17:043128490-043128539 | INSIDE | 0.89198923 | 1.435E-10 |
| 17735 | A_68_P30611435 | Lpp | chr16:024740396-024740452 | INSIDE | 1.175870811 | 1.475E-10 |
| 211934 | A_68_P24164143 | Arpc1a | chr5:145335205-145335264 | PROMOTER | 1.131202787 | 1.485E-10 |
| 29541 | A_68_P28517164 | Spg3a | chr12:070812162-070812220 | INSIDE | 1.092501398 | 1.515E-10 |
| 124794 | A_68_P20840488 | Ifi202b-Olfr433 | chr1:175873149-175873206 | DIVERGENT_PROMOTER | 1.019436355 | 1.555E-10 |
| 112503 | A_68_P32682227 | BC065397 | chrX:132092214-132092273 | DOWNSTREAM | 0.945025013 | 1.736E-10 |
| 233175 | A_68_P20788019 | Slc19a2 | chr1:166086861-166086920 | INSIDE | 0.879994104 | 1.765E-10 |
| 29029 | A_68_P28157933 | Acox1 | chr11:116014112-116014171 | INSIDE | 1.421770283 | 1.78E-10 |
| 230959 | A_68_P25584749 | Cyp2e1 | chr7:140615106-140615163 | PROMOTER | 0.904442605 | 2.023E-10 |
| 229828 | A_68_P23201993 | Cdkn2c | chr4:109162876-109162934 | INSIDE | 0.945515295 | 2.105E-10 |
| 143740 | A_68_P27924684 | Ovca2 | chr11:074997232-074997287 | PROMOTER | 1.071851709 | 2.185E-10 |
| 110742 | A_68_P24808243 | Phc1 | chr6:122309587-122309637 | PROMOTER | 1.28669447 | 2.395E-10 |
| 106074 | A_68_P28185410 | Arhgdia | chr11:120393867-120393915 | INSIDE | 1.197616454 | 2.52E-10 |
| 19823 | A_68_P29619186 | Nedd8 | chr14:054622153-054622211 | INSIDE | 0.976298973 | 2.52E-10 |
| 144457 | A_68_P23883559 | Afm | chr5:091595714-091595773 | INSIDE | 1.037349992 | 2.593E-10 |
| 192024 | A_68_P31371514 | Fez2 | chr17:078320743-078320795 | INSIDE | 0.982769045 | 2.84E-10 |
| 151439 | A_68_P27431013 | Pawr | chr10:107733486-107733545 | PROMOTER | 0.894088136 | 3.165E-10 |
| 97028 | A_68_P20353089 | Resp18 | chr1:075160843-075160888 | INSIDE | 1.052494927 | 3.195E-10 |
| 20649 | A_68_P31959627 | Olfr76 | chr19:012190207-012190266 | PROMOTER | 0.999946139 | 3.278E-10 |
| 177703 | A_68_P26350539 | Elof1 | chr9:021870011-021870070 | PROMOTER | 0.910456623 | 3.38E-10 |
| 16836 | A_68_P32460622 | Pnma3 | chrX:069319098-069319157 | INSIDE | 1.689321023 | 3.54E-10 |
| 196147 | A_68_P22409831 | Gstm3 | chr3:108098693-108098742 | PROMOTER | 0.985126479 | 3.675E-10 |
| 141901 | A_68_P22653957 | St6galnac5 | chr3:152923519-152923567 | PROMOTER | 1.168202686 | 3.76E-10 |
| 235901 | A_68_P32055916 | Trpd52l3 | chr19:030064287-030064346 | PROMOTER | 1.301296695 | 3.795E-10 |
| 9055 | A_68_P22387700 | Olfml3 | chr3:103866512-103866566 | INSIDE | 0.949041356 | 3.835E-10 |
| 111087 | A_68_P31093921 | Tceb2 | chr17:023559922-023559970 | PROMOTER | 0.94078337 | 3.87E-10 |
| 204445 | A_68_P25500658 | Atp2a1 | chr7:126257877-126257935 | PROMOTER | 0.922887477 | 3.974E-10 |
| 29678 | A_68_P26433681 | Olfr889 | chr9:037866262-037866321 | PROMOTER | 0.972791531 | 3.981E-10 |
| 55983 | A_68_P30380528 | Polr3h-Csdc2 | chr15:081760260-081760316 | DIVERGENT_PROMOTER | 0.90465666 | 4.06E-10 |
| 146041 | A_68_P23155624 | Dnajc6 | chr4:101050140-101050199 | INSIDE | 0.914250663 | 4.13E-10 |
| 216685 | A_68_P30346988 | Plec1 | chr15:076060990-076061034 | PROMOTER | 0.909624396 | 4.61E-10 |
| 15939 | A_68_P27258631 | Gstt1 | chr10:075229885-075229944 | INSIDE | 1.042775593 | 4.66E-10 |
| 79079 | A_68_P27687860 | Mare-Hba-x | chr11:032171962-032172021 | DIVERGENT_PROMOTER | 0.911184278 | 4.668E-10 |
| 132235 | A_68_P20510999 | Serpinb12 | chr1:108758746-108758805 | PROMOTER | 0.926011786 | 4.715E-10 |
| 163653 | A_68_P25736417 | Gpr124 | chr8:028547176-028547235 | PROMOTER | 1.030792961 | 4.756E-10 |
| 31788 | A_68_P31606716 | Stard4 | chr18:033339763-033339816 | PROMOTER | 1.039650085 | 4.87E-10 |
| 71903 | A_68_P20045416 | Eya1 | chr1:014296301-014296360 | PROMOTER | 1.29985564 | 5.3E-10 |
| 131780 | A_68_P26217168 | Crispld2 | chr8:122874680-122874739 | PROMOTER | 0.927566098 | 5.36E-10 |
| 28331 | A_68_P32370559 | Olfr1322 | chrX:046134514-046134571 | PROMOTER | 0.978503285 | 5.425E-10 |
| 22515 | A_68_P29654724 | Dleu7 | chr14:061250485-061250544 | PROMOTER | 0.964608573 | 5.5E-10 |
| 145105 | A_68_P30496296 | Soat2 | chr15:101978711-101978755 | INSIDE | 0.883725961 | 5.7E-10 |
| 178693 | A_68_P29529665 | Mmrn2 | chr14:033206674-033206726 | INSIDE | 1.213576788 | 5.8E-10 |
| 142144 | A_68_P21140534 | Olfr356 | chr2:036759485-036759533 | INSIDE | 0.876614207 | 5.95E-10 |
| 178098 | A_68_P21428345 | Cry2 | chr2:092207045-092207093 | INSIDE | 0.971236881 | 6.55E-10 |
| 64184 | A_68_P32143095 | Nfkb2 | chr19:046361159-046361215 | INSIDE | 0.907055356 | 7.3E-10 |
| 12253 | A_68_P21099483 | Trub2 | chr2:029614457-029614516 | PROMOTER | 0.870805262 | 7.4E-10 |
| 123710 | A_68_P26980642 | Olig3 | chr10:019044276-019044328 | PROMOTER | 1.312160916 | 7.5E-10 |
| 53369 | A_68_P20447253 | Hdac4 | chr1:093977373-093977432 | INSIDE | 1.217243188 | 7.6E-10 |
| 43159 | A_68_P28168303 | Socs3 | chr11:117786656-117786704 | PROMOTER | 0.933224115 | 7.75E-10 |
| 15606 | A_68_P32300591 | Wdr44 | chrX:022847259-022847318 | PROMOTER | 1.302828106 | 8.1E-10 |
| 54215 | A_68_P28671861 | Ptpn21-Zc3h14 | chr12:099142094-099142152 | DIVERGENT_PROMOTER | 0.87888927 | 8.1E-10 |
| 79170 | A_68_P22410919 | Gpr61 | chr3:108283573-108283632 | PROMOTER | 1.101118609 | 8.45E-10 |
| 25661 | A_68_P24333624 | Tsga14 | chr6:030644882-030644941 | PROMOTER | 1.322574158 | 8.55E-10 |
| 132506 | A_68_P24994426 | Relb | chr7:018788548-018788607 | PROMOTER | 0.995499611 | 8.55E-10 |
| 139656 | A_68_P31375034 | Prkcn | chr17:078921359-078921418 | PROMOTER | 1.166409395 | 8.75E-10 |
| 145643 | A_68_P30484204 | Dip2b | chr15:099979807-099979859 | PROMOTER | 1.189147733 | 8.75E-10 |
| 217700 | A_68_P25089148 | Vrk3 | chr7:044617223-044617282 | INSIDE | 0.9709653 | 1.124E-09 |
| 220426 | A_68_P25391267 | Olfr686 | chr7:105079625-105079684 | PROMOTER | 1.01521927 | 1.155E-09 |
| 4754 | A_68_P25005012 | Plaur | chr7:024173225-024173272 | INSIDE | 1.106917079 | 1.185E-09 |
| 142653 | A_68_P26717027 | Tbc1d2b | chr9:090070803-090070856 | PROMOTER | 1.196443415 | 1.185E-09 |
| 17102 | A_68_P21750128 | Defb27-Defb36 | chr2:152295349-152295395 | DIVERGENT_PROMOTER | 0.85806788 | 1.26E-09 |
| 26903 | A_68_P29667783 | Msra | chr14:063399265-063399317 | INSIDE | 1.228114373 | 1.295E-09 |
| 5491 | A_68_P31613990 | Cdc23 | chr18:034774757-034774806 | INSIDE | 1.592214131 | 1.305E-09 |
| 93572 | A_68_P32796671 | Ace2 | chrX:159479449-159479508 | PROMOTER | 0.869380131 | 1.325E-09 |
| 151571 | A_68_P20594520 | Mgat5 | chr1:129132341-129132385 | PROMOTER | 1.32695277 | 1.345E-09 |
| 17558 | A_68_P31963501 | Olfr1454 | chr19:013131873-013131932 | DOWNSTREAM | 1.118576028 | 1.369E-09 |
| 40743 | A_68_P27543993 | Itga7 | chr10:128337953-128338008 | INSIDE | 1.453149181 | 1.52E-09 |
| 4875 | A_68_P24394650 | Ephb6 | chr6:041534151-041534204 | PROMOTER | 0.924414746 | 1.565E-09 |
| 143656 | A_68_P30494344 | Krt1 | chr15:101678920-101678977 | PROMOTER | 0.920865369 | 1.58E-09 |
| 149188 | A_68_P32140301 | Npm3 | chr19:045804618-045804675 | PROMOTER | 0.894102215 | 1.585E-09 |
| 11094 | A_68_P27134076 | Gprc6a | chr10:051319601-051319660 | INSIDE | 0.955453402 | 1.7E-09 |
| 129443 | A_68_P23455513 | Pftk1 | chr5:005388679-005388738 | PROMOTER | 0.876010662 | 1.78E-09 |
| 55657 | A_68_P26702559 | Cyb5r4 | chr9:086816940-086816999 | PROMOTER | 1.408998014 | 1.85E-09 |
| 137852 | A_68_P23435062 | Tnfrsf14 | chr4:153773756-153773802 | PROMOTER | 0.925437035 | 1.895E-09 |
| 229054 | A_68_P21127586 | Mapkap1 | chr2:034253584-034253643 | PROMOTER | 0.98465877 | 2.158E-09 |
| 70684 | A_68_P22363110 | Tbx15 | chr3:099380513-099380568 | PROMOTER | 1.161556711 | 2.185E-09 |
| 100248 | A_68_P24811533 | Clec4a2 | chr6:123089301-123089360 | INSIDE | 0.982513546 | 2.255E-09 |
| 63239 | A_68_P24156258 | Grid2ip | chr5:143636861-143636913 | INSIDE | 0.941416071 | 2.26E-09 |
| 8187 | A_68_P30339467 | Ly6d | chr15:074589588-074589637 | INSIDE | 1.233021533 | 2.285E-09 |
| 28281 | A_68_P21096017 | Ntng2 | chr2:029067623-029067677 | INSIDE | 1.081194003 | 2.39E-09 |
| 19608 | A_68_P26139237 | Smpd3 | chr8:109232033-109232091 | PROMOTER | 1.629885543 | 2.41E-09 |
| 106057 | A_68_P21117424 | Ptrh1 | chr2:032599218-032599262 | INSIDE | 0.871515885 | 2.43E-09 |
| 87914 | A_68_P22393162 | Rhoc | chr3:104915962-104916021 | PROMOTER | 0.900616841 | 2.475E-09 |
| 63297 | A_68_P20271624 | Ctla4 | chr1:060853927-060853983 | INSIDE | 1.621963387 | 2.485E-09 |
| 22322 | A_68_P31926952 | Rab1b | chr19:005104825-005104873 | INSIDE | 1.244903128 | 2.5E-09 |
| 48680 | A_68_P28513207 | Rpl36al | chr12:070100422-070100481 | DOWNSTREAM | 1.003330668 | 2.53E-09 |
| 10295 | A_68_P27513168 | Usp15 | chr10:122601816-122601875 | PROMOTER | 1.16882763 | 2.565E-09 |
| 215908 | A_68_P27783254 | Olfr1384 | chr11:049353293-049353350 | PROMOTER | 0.916942429 | 2.695E-09 |
| 63759 | A_68_P20633680 | Rabif | chr1:136309411-136309470 | PROMOTER | 1.428767278 | 2.72E-09 |
| 78760 | A_68_P27542221 | Erbb3 | chr10:127991654-127991713 | INSIDE | 1.648524104 | 2.85E-09 |
| 97133 | A_68_P22359350 | Hmgcs2 | chr3:098365683-098365742 | PROMOTER | 1.177678374 | 2.85E-09 |
| 138670 | A_68_P30478826 | Kcnh3 | chr15:099053211-099053259 | INSIDE | 0.902300244 | 2.895E-09 |
| 30328 | A_68_P23561082 | En2 | chr5:028493270-028493321 | PROMOTER | 0.891516781 | 3.03E-09 |
| 97608 | A_68_P21141519 | Olfr368 | chr2:037154731-037154788 | DOWNSTREAM | 1.013452521 | 3.135E-09 |
| 94050 | A_68_P26759324 | Copb2 | chr9:098373981-098374040 | INSIDE | 1.886229369 | 3.28E-09 |
| 11119 | A_68_P27352242 | Pctk2 | chr10:092634735-092634794 | PROMOTER | 0.88454369 | 3.285E-09 |
| 235790 | A_68_P30494863 | Krt4 | chr15:101751519-101751578 | INSIDE | 0.974017999 | 3.285E-09 |
| 237154 | A_68_P27310802 | Pwp1 | chr10:085303994-085304053 | INSIDE | 1.141277882 | 3.285E-09 |
| 26021 | A_68_P31145171 | Akap8l | chr17:032086884-032086941 | PROMOTER | 1.098001266 | 3.31E-09 |
| 128501 | A_68_P24613264 | Aak1 | chr6:086959881-086959940 | DOWNSTREAM | 0.878467028 | 3.419E-09 |
| 46325 | A_68_P28070034 | Ghdc | chr11:100583104-100583152 | INSIDE | 0.952585685 | 3.56E-09 |
| 55567 | A_68_P24429128 | Gimap5 | chr6:048674056-048674115 | PROMOTER | 1.076508498 | 3.81E-09 |
| 205648 | A_68_P27532826 | Xrcc6bp1 | chr10:126302141-126302200 | INSIDE | 1.310729347 | 3.81E-09 |
| 25811 | A_68_P21908941 | Eef1a2 | chr2:181087375-181087428 | PROMOTER | 1.080172887 | 3.85E-09 |
| 41185 | A_68_P24005522 | Gltp | chr5:114952317-114952364 | PROMOTER | 0.871790562 | 3.905E-09 |
| 177370 | A_68_P20451899 | Ankmy1 | chr1:094732972-094733031 | INSIDE | 1.028595433 | 4.005E-09 |
| 32053 | A_68_P28963736 | Slc35b3 | chr13:038972603-038972657 | PROMOTER | 1.053680615 | 4.035E-09 |
| 176844 | A_68_P21571386 | Ltk | chr2:119452274-119452324 | PROMOTER | 0.936881651 | 4.055E-09 |
| 205036 | A_68_P29055531 | Pdlim7 | chr13:055526823-055526879 | PROMOTER | 0.865996611 | 4.15E-09 |
| 89701 | A_68_P21771106 | Epb4.1l1 | chr2:156109562-156109618 | PROMOTER | 1.125271915 | 4.34E-09 |
| 76993 | A_68_P28431149 | Ap4s1 | chr12:052612406-052612465 | PROMOTER | 1.250912465 | 4.44E-09 |
| 200242 | A_68_P20393462 | Col4a4 | chr1:082465435-082465490 | INSIDE | 0.932883727 | 4.725E-09 |
| 19475 | A_68_P24164303 | Arpc1b | chr5:145365884-145365935 | PROMOTER | 1.009297241 | 4.81E-09 |
| 181511 | A_68_P26504353 | Il18 | chr9:050320046-050320105 | INSIDE | 0.922425575 | 4.855E-09 |
| 112519 | A_68_P30387873 | Arfgap3 | chr15:083183238-083183297 | PROMOTER | 1.740106108 | 5.15E-09 |
| 153622 | A_68_P21754275 | Asxl1 | chr2:153033259-153033312 | PROMOTER | 1.006310668 | 5.2E-09 |
| 129134 | A_68_P22970114 | AI597013 | chr4:062869778-062869830 | INSIDE | 1.073811834 | 5.45E-09 |
| 86200 | A_68_P21970134 | Cyp7b1 | chr3:018438340-018438399 | PROMOTER | 1.780530856 | 5.5E-09 |
| 215007 | A_68_P28737506 | Dio3 | chr12:110722051-110722106 | PROMOTER | 1.612597685 | 5.85E-09 |
| 8005 | A_68_P31201730 | Gm323 | chr17:045012554-045012598 | INSIDE | 1.243873459 | 5.85E-09 |
| 144324 | A_68_P28604987 | Jundm2 | chr12:086485296-086485355 | PROMOTER | 1.310584498 | 6.05E-09 |
| 45860 | A_68_P30721010 | Ccdc52 | chr16:044262447-044262506 | PROMOTER | 1.015202704 | 6.1E-09 |
| 235415 | A_68_P26472059 | Tmprss4 | chr9:044958102-044958159 | PROMOTER | 1.207351318 | 6.2E-09 |
| 58749 | A_68_P28078031 | Lsm12-G6pc3 | chr11:102005727-102005771 | DIVERGENT_PROMOTER | 0.956703561 | 6.7E-09 |
| 64168 | A_68_P29599171 | Tmem55b | chr14:049850278-049850337 | INSIDE | 0.872500787 | 7.1E-09 |
| 69127 | A_68_P25359994 | Uvrag | chr7:099014535-099014594 | INSIDE | 0.934072216 | 7.85E-09 |
| 138220 | A_68_P26492098 | Rbm7 | chr9:048242001-048242060 | INSIDE | 1.051750013 | 7.98E-09 |
| 98090 | A_68_P24569766 | Pap | chr6:078296381-078296440 | PROMOTER | 1.061372587 | 8E-09 |
| 103087 | A_68_P30752359 | Ccdc54 | chr16:050508918-050508977 | DOWNSTREAM | 0.916706465 | 1.045E-08 |
| 45064 | A_68_P32049550 | Slc1a1 | chr19:028903464-028903523 | INSIDE | 1.081486063 | 1.09E-08 |
| 191517 | A_68_P20349527 | Stk36 | chr1:074534907-074534951 | INSIDE | 0.871067672 | 1.16E-08 |
| 187147 | A_68_P32470776 | Mpp1 | chrX:071382922-071382981 | INSIDE | 0.954684173 | 1.21E-08 |
| 74823 | A_68_P24707921 | Chl1 | chr6:103478820-103478879 | INSIDE | 1.174115052 | 1.22E-08 |
| 18510 | A_68_P25101055 | Saa1-Saa2 | chr7:046615693-046615752 | DIVERGENT_PROMOTER | 1.060257163 | 1.225E-08 |
| 162535 | A_68_P25950075 | Rfxank | chr8:073069723-073069769 | PROMOTER | 0.928204355 | 1.29E-08 |
| 125861 | A_68_P23325576 | D4Wsu53e | chr4:134197913-134197971 | INSIDE | 1.081888016 | 1.315E-08 |
| 223803 | A_68_P25701546 | Xkr5 | chr8:018954810-018954869 | PROMOTER | 0.914506538 | 1.325E-08 |
| 37241 | A_68_P23617886 | Nsg1 | chr5:038449335-038449388 | PROMOTER | 0.97834711 | 1.355E-08 |
| 215423 | A_68_P25025838 | Psmd8 | chr7:028894328-028894375 | PROMOTER | 1.044180349 | 1.38E-08 |
| 23471 | A_68_P26440712 | Olfr976 | chr9:039710343-039710402 | PROMOTER | 1.065127182 | 1.545E-08 |
| 143697 | A_68_P29055742 | BC021381 | chr13:055557224-055557278 | INSIDE | 0.995934626 | 1.56E-08 |
| 158468 | A_68_P25348869 | Gab2 | chr7:096952361-096952419 | PROMOTER | 1.459099831 | 1.615E-08 |
| 209397 | A_68_P29612386 | Mmp14 | chr14:053385520-053385574 | PROMOTER | 0.883949277 | 1.63E-08 |
| 158331 | A_68_P21752132 | Dusp15-Ttll9 | chr2:152645962-152646011 | DIVERGENT_PROMOTER | 0.940719934 | 1.675E-08 |
| 7711 | A_68_P20982093 | Cugbp2 | chr2:006804779-006804838 | PROMOTER | 0.982630257 | 1.787E-08 |
| 104020 | A_68_P25925439 | Sc4mol | chr8:067628367-067628421 | PROMOTER | 1.458441347 | 1.855E-08 |
| 62598 | A_68_P30490101 | Acvr1b | chr15:101003931-101003990 | INSIDE | 0.904113428 | 1.935E-08 |
| 237531 | A_68_P26005873 | Inpp4b | chr8:084605738-084605797 | PROMOTER | 1.288694515 | 2.015E-08 |
| 152993 | A_68_P21699767 | Snrpb2 | chr2:142749168-142749227 | PROMOTER | 0.924352506 | 2.06E-08 |
| 157987 | A_68_P31783755 | Grp | chr18:066000691-066000744 | INSIDE | 1.11428698 | 2.315E-08 |
| 233167 | A_68_P20769359 | Serpinc1 | chr1:162818953-162819012 | PROMOTER | 1.452884794 | 2.37E-08 |
| 130466 | A_68_P31414326 | Srbd1 | chr17:085953148-085953204 | PROMOTER | 1.186808471 | 2.49E-08 |
| 185048 | A_68_P29542539 | Gcap14 | chr14:035882214-035882273 | PROMOTER | 1.191903609 | 2.496E-08 |
| 188222 | A_68_P26341787 | Olfr869 | chr9:019882915-019882974 | PROMOTER | 0.884346168 | 2.608E-08 |
| 22437 | A_68_P22873673 | Olfr159 | chr4:043790410-043790461 | DOWNSTREAM | 1.057434861 | 2.665E-08 |
| 23305 | A_68_P32239625 | Pdzx | chrX:006832617-006832671 | INSIDE | 1.168389545 | 2.665E-08 |
| 65579 | A_68_P28542605 | Dbpht2 | chr12:075216320-075216379 | INSIDE | 0.868075682 | 2.765E-08 |
| 229470 | A_68_P29011239 | Tpmt | chr13:047049427-047049486 | INSIDE | 0.902932023 | 2.79E-08 |
| 6669 | A_68_P27282868 | Rkhd1 | chr10:079789588-079789632 | INSIDE | 0.922653474 | 2.87E-08 |
| 76876 | A_68_P22695684 | Rdhe2 | chr4:003946133-003946192 | INSIDE | 1.269544855 | 3E-08 |
| 165112 | A_68_P23325207 | Tmem57 | chr4:134123989-134124048 | INSIDE | 1.021007752 | 3.035E-08 |
| 31045 | A_68_P32215367 | Hspa12a | chr19:058849173-058849232 | INSIDE | 1.061833676 | 3.2E-08 |
| 8601 | A_68_P27546117 | Olfr790 | chr10:128905649-128905708 | DOWNSTREAM | 0.907270782 | 3.28E-08 |
| 6166 | A_68_P25587272 | Hras1 | chr7:141042582-141042630 | DOWNSTREAM | 1.048802827 | 3.295E-08 |
| 95024 | A_68_P21626139 | Pdyn | chr2:129396148-129396201 | PROMOTER | 1.294379028 | 3.395E-08 |
| 78159 | A_68_P24907691 | St8sia1 | chr6:142923349-142923408 | PROMOTER | 1.007887536 | 3.425E-08 |
| 49702 | A_68_P22829245 | Cnr1 | chr4:034250704-034250763 | PROMOTER | 1.029213367 | 3.44E-08 |
| 152935 | A_68_P21143395 | Strbp | chr2:037474548-037474607 | INSIDE | 0.976368815 | 3.525E-08 |
| 108893 | A_68_P29631124 | Efha1 | chr14:056955227-056955286 | PROMOTER | 1.153001411 | 3.545E-08 |
| 65322 | A_68_P32056999 | Gldc | chr19:030245596-030245655 | PROMOTER | 0.946756447 | 3.645E-08 |
| 213794 | A_68_P28879970 | V1rh14 | chr13:023124796-023124855 | PROMOTER | 1.018958644 | 3.82E-08 |
| 109511 | A_68_P23350147 | Capzb | chr4:138461359-138461418 | PROMOTER | 1.261455631 | 3.915E-08 |
| 161267 | A_68_P27672196 | Efemp1 | chr11:028769158-028769217 | INSIDE | 0.955342226 | 3.965E-08 |
| 194817 | A_68_P31150964 | Zbtb22 | chr17:033527576-033527621 | INSIDE | 0.894341705 | 4.005E-08 |
| 177242 | A_68_P26129684 | Tlm | chr8:107550256-107550313 | PROMOTER | 1.331799119 | 4.01E-08 |
| 11870 | A_68_P27901224 | Pld2 | chr11:070356625-070356669 | INSIDE | 1.037362929 | 4.2E-08 |
| 175435 | A_68_P21010248 | Itga8 | chr2:012218212-012218271 | INSIDE | 1.019254734 | 4.227E-08 |
| 209247 | A_68_P32111300 | Ccnj | chr19:040880929-040880978 | PROMOTER | 1.098537345 | 4.315E-08 |
| 95310 | A_68_P27286262 | Lmnb2 | chr10:080309383-080309432 | INSIDE | 1.076335068 | 4.32E-08 |
| 216938 | A_68_P24953513 | V1rd11 | chr7:005488631-005488690 | PROMOTER | 1.019268794 | 4.385E-08 |
| 237083 | A_68_P24402012 | Olfr435 | chr6:043129134-043129193 | PROMOTER | 0.947696694 | 4.431E-08 |
| 121702 | A_68_P29611634 | Dad1 | chr14:053211600-053211651 | INSIDE | 0.903713283 | 4.475E-08 |
| 182119 | A_68_P23893606 | Cxcl9 | chr5:093402456-093402515 | INSIDE | 1.211449905 | 4.495E-08 |
| 32366 | A_68_P21416574 | Olfr1273 | chr2:090098436-090098495 | PROMOTER | 1.012705848 | 4.515E-08 |
| 99735 | A_68_P26972797 | Heca | chr10:017640277-017640336 | PROMOTER | 0.957079049 | 4.58E-08 |
| 167433 | A_68_P20331584 | Atic | chr1:071488218-071488277 | PROMOTER | 1.13855066 | 4.745E-08 |
| 157898 | A_68_P28156344 | Sap30bp | chr11:115754178-115754230 | INSIDE | 1.024415465 | 4.92E-08 |
| 199769 | A_68_P22331539 | Sprr3 | chr3:092544391-092544450 | PROMOTER | 1.077662617 | 5.05E-08 |
| 87595 | A_68_P22386708 | Syt6 | chr3:103700679-103700738 | PROMOTER | 0.925477813 | 5.056E-08 |
| 63585 | A_68_P26595498 | Aph1c | chr9:066638414-066638473 | PROMOTER | 1.373853032 | 5.25E-08 |
| 181905 | A_68_P27848459 | Zfp179 | chr11:061269378-061269428 | INSIDE | 0.913347381 | 5.35E-08 |
| 120174 | A_68_P27846347 | Kcnj12 | chr11:060884189-060884243 | INSIDE | 1.244581831 | 5.7E-08 |
| 238581 | A_68_P21199146 | Acvr2a | chr2:048638456-048638509 | INSIDE | 0.927703344 | 5.8E-08 |
| 42383 | A_68_P30479852 | Tegt | chr15:099216621-099216680 | PROMOTER | 1.128501362 | 6.06E-08 |
| 38424 | A_68_P22292322 | Tmem154 | chr3:084748490-084748549 | PROMOTER | 1.007694757 | 6.102E-08 |
| 65428 | A_68_P30474630 | Olfr281 | chr15:098280731-098280781 | PROMOTER | 1.198594652 | 6.15E-08 |
| 5548 | A_68_P30642628 | Lsg1-BC022623 | chr16:030514275-030514333 | DIVERGENT_PROMOTER | 0.982325674 | 6.235E-08 |
| 108934 | A_68_P25382970 | Olfr600 | chr7:103222570-103222629 | PROMOTER | 0.858212394 | 6.485E-08 |
| 59165 | A_68_P28184849 | Hgs | chr11:120300032-120300091 | INSIDE | 1.038210023 | 6.5E-08 |
| 100752 | A_68_P21815203 | Rims4 | chr2:163610883-163610938 | PROMOTER | 0.862085608 | 6.65E-08 |
| 172680 | A_68_P26624026 | Cgnl1 | chr9:071571148-071571207 | PROMOTER | 0.920521685 | 7.15E-08 |
| 234451 | A_68_P23141907 | Angptl3 | chr4:098522388-098522447 | PROMOTER | 1.037503832 | 7.25E-08 |
| 118445 | A_68_P24449438 | Hoxa11 | chr6:052179438-052179497 | PROMOTER | 1.113215342 | 7.3E-08 |
| 47855 | A_68_P25103276 | Ptpn5 | chr7:047003662-047003710 | PROMOTER | 0.919634371 | 7.3E-08 |
| 218469 | A_68_P30488504 | Scn8a | chr15:100758291-100758350 | INSIDE | 1.129115617 | 7.6E-08 |
| 36505 | A_68_P21973833 | Armc1 | chr3:019357272-019357331 | PROMOTER | 1.081067648 | 7.925E-08 |
| 152037 | A_68_P30578499 | Zdhhc8 | chr16:018149043-018149095 | PROMOTER | 1.32204894 | 8.15E-08 |
| 99744 | A_68_P27482581 | Mdm2 | chr10:117116704-117116756 | PROMOTER | 0.986255997 | 8.25E-08 |
| 220065 | A_68_P30984776 | Kcnj6 | chr16:095110816-095110875 | PROMOTER | 0.929726525 | 8.45E-08 |
| 181485 | A_68_P29250255 | Enc1 | chr13:098343614-098343673 | INSIDE | 0.949067547 | 8.45E-08 |
| 60263 | A_68_P25004270 | Zfp94 | chr7:024027267-024027319 | PROMOTER | 0.882309197 | 8.5E-08 |
| 42318 | A_68_P22834498 | Ifnk | chr4:035335853-035335912 | PROMOTER | 0.889855668 | 8.61E-08 |
| 575 | A_68_P23090680 | Ptplad2 | chr4:087911701-087911760 | PROMOTER | 1.06928767 | 8.95E-08 |
| 125295 | A_68_P24777675 | Rassf4 | chr6:116642498-116642553 | PROMOTER | 1.2046656 | 9E-08 |
| 14976 | A_68_P24815590 | C1rl | chr6:124455647-124455691 | PROMOTER | 1.079471206 | 9.1E-08 |
| 224641 | A_68_P29032292 | Edg3 | chr13:051418877-051418928 | PROMOTER | 1.050646262 | 9.25E-08 |
| 111207 | A_68_P23532208 | Orc5l | chr5:022064537-022064588 | PROMOTER | 0.892332467 | 9.25E-08 |
| 208870 | A_68_P29512035 | Sh3bp5 | chr14:030262500-030262559 | INSIDE | 0.886191665 | 9.301E-08 |
| 78527 | A_68_P27565764 | Gck | chr11:005848132-005848185 | INSIDE | 1.115974786 | 9.35E-08 |
| 73686 | A_68_P23887074 | Areg | chr5:092213053-092213112 | PROMOTER | 1.080038587 | 9.659E-08 |
| 170306 | A_68_P29510426 | Nisch | chr14:030032926-030032976 | INSIDE | 1.065150752 | 1.055E-07 |
| 139122 | A_68_P21554578 | Tmco5 | chr2:116568888-116568947 | PROMOTER | 0.88144494 | 1.13E-07 |
| 32222 | A_68_P27805749 | Il4 | chr11:053465791-053465846 | PROMOTER | 1.020350092 | 1.15E-07 |
| 115746 | A_68_P20137144 | Dst | chr1:034105332-034105381 | INSIDE | 0.973639995 | 1.19E-07 |
| 138314 | A_68_P32453961 | Cd99l2 | chrX:067752807-067752866 | INSIDE | 1.260284542 | 1.195E-07 |
| 44370 | A_68_P32227594 | Sfxn4 | chr19:060915842-060915889 | INSIDE | 0.94561506 | 1.23E-07 |
| 110888 | A_68_P25030888 | Alkbh6 | chr7:030016730-030016789 | PROMOTER | 1.182575946 | 1.235E-07 |
| 201680 | A_68_P32288222 | Rp2h | chrX:019521942-019521986 | INSIDE | 0.898716327 | 1.245E-07 |
| 196986 | A_68_P31932863 | Snx15 | chr19:006127020-006127079 | INSIDE | 1.00830399 | 1.245E-07 |
| 99377 | A_68_P22690587 | Gpr177 | chr3:159777084-159777137 | INSIDE | 1.25295457 | 1.25E-07 |
| 187611 | A_68_P32219570 | Emx2 | chr19:059512568-059512612 | INSIDE | 1.165161955 | 1.26E-07 |
| 158633 | A_68_P25937490 | Nat1 | chr8:070420230-070420286 | INSIDE | 0.890222236 | 1.29E-07 |
| 90108 | A_68_P22276375 | Ctso | chr3:082020740-082020799 | INSIDE | 0.899701662 | 1.3E-07 |
| 205820 | A_68_P27367713 | Eea1 | chr10:095367473-095367532 | PROMOTER | 1.309635074 | 1.37E-07 |
| 145330 | A_68_P27902157 | AI842396 | chr11:070506442-070506501 | INSIDE | 1.576101512 | 1.38E-07 |
| 201297 | A_68_P22659669 | Rabggtb | chr3:153851050-153851109 | PROMOTER | 1.19267508 | 1.405E-07 |
| 109774 | A_68_P30014081 | Adamts12 | chr15:011006094-011006152 | PROMOTER | 0.859901825 | 1.405E-07 |
| 198703 | A_68_P22182556 | Rap2b | chr3:061449454-061449513 | PROMOTER | 1.615126633 | 1.415E-07 |
| 143792 | A_68_P24294797 | Aass | chr6:023088102-023088161 | PROMOTER | 0.978480662 | 1.464E-07 |
| 160283 | A_68_P25616732 | Map2k7 | chr8:004239356-004239415 | INSIDE | 1.347883381 | 1.6E-07 |
| 30770 | A_68_P31928910 | Efemp2 | chr19:005475396-005475440 | INSIDE | 0.8668645 | 1.665E-07 |
| 36937 | A_68_P32036050 | Smarca2 | chr19:026669170-026669229 | PROMOTER | 0.859802439 | 1.82E-07 |
| 60276 | A_68_P23092714 | Ifne1 | chr4:088352079-088352138 | PROMOTER | 0.976309648 | 1.86E-07 |
| 55033 | A_68_P26434021 | Olfr894 | chr9:037966332-037966391 | PROMOTER | 1.006915929 | 1.865E-07 |
| 19967 | A_68_P24010202 | Pxn | chr5:115816636-115816681 | INSIDE | 0.893588363 | 1.89E-07 |
| 96561 | A_68_P27947431 | Ksr1 | chr11:078965837-078965892 | PROMOTER | 0.974264035 | 1.895E-07 |
| 13921 | A_68_P27027645 | Ptprk | chr10:027765845-027765904 | INSIDE | 0.993931787 | 2.015E-07 |
| 148169 | A_68_P28914816 | E2f3 | chr13:029996944-029997003 | PROMOTER | 1.269766565 | 2.065E-07 |
| 37661 | A_68_P30718946 | Gramd1c | chr16:043902597-043902656 | INSIDE | 1.230216848 | 2.085E-07 |
| 10993 | A_68_P26643318 | Gnb5 | chr9:075094707-075094758 | PROMOTER | 1.234597185 | 2.14E-07 |
| 160921 | A_68_P21481073 | Pdhx | chr2:102873354-102873413 | INSIDE | 1.134671515 | 2.225E-07 |
| 150051 | A_68_P28357583 | Sostdc1 | chr12:036819354-036819413 | PROMOTER | 0.907339412 | 2.252E-07 |
| 128752 | A_68_P21776612 | Manbal | chr2:157056828-157056887 | PROMOTER | 0.886689862 | 2.36E-07 |
| 222336 | A_68_P25313867 | Tmem126b | chr7:090356650-090356709 | PROMOTER | 0.98783398 | 2.41E-07 |
| 233807 | A_68_P27900267 | Alox15 | chr11:070167363-070167407 | INSIDE | 0.990959686 | 2.415E-07 |
| 22391 | A_68_P27896317 | Sox15 | chr11:069470662-069470721 | PROMOTER | 0.87087505 | 2.501E-07 |
| 57943 | A_68_P28048145 | Sp6 | chr11:096829519-096829568 | PROMOTER | 1.163309112 | 2.62E-07 |
| 29913 | A_68_P24482323 | Abcg2 | chr6:058524602-058524653 | PROMOTER | 0.876157069 | 2.805E-07 |
| 179825 | A_68_P32694911 | Rnf128 | chrX:134953398-134953457 | PROMOTER | 0.92349115 | 2.835E-07 |
| 200851 | A_68_P26139646 | Zfp90 | chr8:109299439-109299498 | PROMOTER | 0.88745153 | 2.88E-07 |
| 111623 | A_68_P26553651 | Arih1 | chr9:059286968-059287027 | PROMOTER | 1.520536911 | 2.9E-07 |
| 120828 | A_68_P32457118 | Magea4 | chrX:068474158-068474211 | PROMOTER | 0.877368501 | 3.07E-07 |
| 232624 | A_68_P30723345 | Gtpbp8 | chr16:044667103-044667159 | PROMOTER | 1.080459844 | 3.125E-07 |
| 31469 | A_68_P29596018 | Olfr722 | chr14:048825494-048825547 | PROMOTER | 0.965604533 | 3.145E-07 |
| 195299 | A_68_P28517736 | Sav1 | chr12:070910140-070910199 | PROMOTER | 0.903941665 | 3.19E-07 |
| 47401 | A_68_P27006483 | Taar7f | chr10:023738487-023738541 | PROMOTER | 0.928138747 | 3.295E-07 |
| 85653 | A_68_P29952583 | Ranbp5 | chr14:120047472-120047531 | INSIDE | 0.982347411 | 3.455E-07 |
| 121379 | A_68_P25026459 | Neud4 | chr7:029012354-029012413 | PROMOTER | 1.099873492 | 3.47E-07 |
| 58991 | A_68_P30587609 | Abcc5 | chr16:020339357-020339416 | INSIDE | 0.920082626 | 3.565E-07 |
| 117794 | A_68_P25366098 | Kcne3 | chr7:100049723-100049775 | PROMOTER | 1.077954876 | 3.715E-07 |
| 55800 | A_68_P32226965 | Nanos1 | chr19:060805806-060805865 | PROMOTER | 0.93231172 | 3.735E-07 |
| 201455 | A_68_P26235229 | Cbfa2t3h | chr8:125583417-125583471 | INSIDE | 1.081533813 | 3.775E-07 |
| 48144 | A_68_P30964458 | Donson | chr16:091579678-091579737 | PROMOTER | 0.860430812 | 3.782E-07 |
| 20729 | A_68_P27484969 | Mdm1 | chr10:117548037-117548094 | INSIDE | 0.941031648 | 3.86E-07 |
| 189960 | A_68_P22347862 | Otud7b | chr3:096246362-096246415 | INSIDE | 0.872476131 | 3.865E-07 |
| 91564 | A_68_P20352175 | Slc23a3 | chr1:075015624-075015683 | INSIDE | 1.244714064 | 4.03E-07 |
| 32597 | A_68_P27537991 | Admr-Rdh1 | chr10:127162557-127162611 | DIVERGENT_PROMOTER | 1.198537865 | 4.05E-07 |
| 182494 | A_68_P30332148 | Gpr20 | chr15:073538386-073538440 | PROMOTER | 0.960848089 | 4.32E-07 |
| 58686 | A_68_P25083657 | Klk14 | chr7:043558871-043558929 | INSIDE | 1.268781066 | 4.49E-07 |
| 225705 | A_68_P31161693 | Mrps18b | chr17:035523795-035523844 | INSIDE | 0.86291477 | 4.565E-07 |
| 217881 | A_68_P26891489 | D9Ertd402e | chr9:122649575-122649634 | PROMOTER | 1.29369972 | 4.72E-07 |
| 145235 | A_68_P30792747 | Cpox | chr16:058614171-058614230 | INSIDE | 1.027886086 | 4.815E-07 |
| 134932 | A_68_P24113789 | Wbscr28 | chr5:135189022-135189073 | INSIDE | 1.059270574 | 4.885E-07 |
| 37329 | A_68_P23610968 | Wfs1 | chr5:037280481-037280540 | PROMOTER | 1.008316523 | 4.976E-07 |
| 6618 | A_68_P27964636 | Ccl1 | chr11:081998376-081998435 | PROMOTER | 1.087216021 | 5.05E-07 |
| 78989 | A_68_P30594921 | Map3k13 | chr16:021801599-021801646 | PROMOTER | 1.053351344 | 5.1E-07 |
| 235021 | A_68_P27944875 | Nlk | chr11:078511969-078512018 | INSIDE | 0.932400179 | 5.1E-07 |
| 86414 | A_68_P25665735 | F10 | chr8:013038893-013038947 | INSIDE | 0.934136748 | 5.2E-07 |
| 63475 | A_68_P32030703 | Dmrt3 | chr19:025675374-025675433 | PROMOTER | 0.940782319 | 5.203E-07 |
| 56143 | A_68_P21747908 | Tcf15 | chr2:151835378-151835422 | INSIDE | 1.192867145 | 5.35E-07 |
| 211194 | A_68_P30365844 | Maff | chr15:079169538-079169595 | PROMOTER | 1.339952638 | 5.4E-07 |
| 163768 | A_68_P28187052 | Fasn | chr11:120642641-120642692 | PROMOTER | 0.940995541 | 5.4E-07 |
| 86113 | A_68_P31708173 | Lox | chr18:052652655-052652714 | INSIDE | 0.950193202 | 5.533E-07 |
| 148664 | A_68_P21732439 | Cst13 | chr2:148521793-148521852 | INSIDE | 0.959336491 | 5.79E-07 |
| 201502 | A_68_P28698644 | Serpina10 | chr12:104031074-104031124 | PROMOTER | 0.865887873 | 5.826E-07 |
| 103750 | A_68_P21814251 | Ada | chr2:163442177-163442233 | PROMOTER | 1.117490507 | 5.9E-07 |
| 9172 | A_68_P27809142 | Il3 | chr11:054110512-054110567 | INSIDE | 1.326156274 | 0.0000006 |
| 153723 | A_68_P20828417 | Cd244 | chr1:173392051-173392110 | PROMOTER | 1.041438396 | 6.1E-07 |
| 132887 | A_68_P29675619 | Pbk | chr14:064759297-064759356 | PROMOTER | 1.262150503 | 6.15E-07 |
| 204602 | A_68_P23440866 | Mxra8 | chr4:154677374-154677430 | PROMOTER | 1.407132749 | 6.2E-07 |
| 130442 | A_68_P28271063 | Odc1 | chr12:017565992-017566050 | PROMOTER | 1.144632599 | 6.2E-07 |
| 212510 | A_68_P23895026 | D5Ertd593e | chr5:093677284-093677337 | PROMOTER | 0.92518025 | 6.25E-07 |
| 92777 | A_68_P26811545 | Rbm6 | chr9:107730353-107730404 | INSIDE | 0.906583772 | 6.3E-07 |
| 129958 | A_68_P21744637 | Sdcbp2 | chr2:151266286-151266340 | INSIDE | 1.063292023 | 6.4E-07 |
| 13232 | A_68_P25401624 | Olfr472 | chr7:107691486-107691427 | PROMOTER | 1.125062697 | 6.5E-07 |
| 108817 | A_68_P29145899 | AI595366 | chr13:074832928-074832987 | PROMOTER | 1.600162347 | 6.55E-07 |
| 41948 | A_68_P20612396 | Il24 | chr1:132715731-132715784 | PROMOTER | 1.03244339 | 6.55E-07 |
| 140429 | A_68_P22359904 | Zfp697 | chr3:098465583-098465640 | PROMOTER | 0.990327661 | 6.7E-07 |
| 154555 | A_68_P25603203 | Nadsyn1 | chr7:143630829-143630888 | INSIDE | 0.957883524 | 6.8E-07 |
| 219437 | A_68_P28931354 | Wrnip1 | chr13:032804994-032805046 | PROMOTER | 0.86278803 | 6.9E-07 |
| 132578 | A_68_P20442005 | BC056923 | chr1:093196510-093196566 | PROMOTER | 0.948805156 | 7.2E-07 |
| 142818 | A_68_P31099860 | Hagh | chr17:024578614-024578673 | INSIDE | 1.107670949 | 7.215E-07 |
| 152500 | A_68_P22412262 | Psrc1 | chr3:108509870-108509929 | PROMOTER | 1.082916589 | 7.215E-07 |
| 58946 | A_68_P29661161 | Blk | chr14:062370973-062371019 | INSIDE | 1.088036013 | 7.3E-07 |
| 33034 | A_68_P28877507 | V1rh4 | chr13:022389066-022389125 | PROMOTER | 0.872105345 | 7.3E-07 |
| 108896 | A_68_P27294754 | Chst11 | chr10:082413222-082413277 | PROMOTER | 1.176935293 | 7.55E-07 |
| 177267 | A_68_P28947087 | Lyrm4 | chr13:036120103-036120162 | INSIDE | 1.09494112 | 7.65E-07 |
| 95047 | A_68_P21630880 | Prosapip1 | chr2:130328543-130328587 | INSIDE | 0.956174836 | 8.1E-07 |
| 89634 | A_68_P30343344 | Top1mt | chr15:075509932-075509986 | PROMOTER | 0.93831126 | 8.3E-07 |
| 85538 | A_68_P31153527 | Btnl1 | chr17:033982930-033982989 | PROMOTER | 1.10433262 | 8.6E-07 |
| 26137 | A_68_P31144781 | Brd4 | chr17:032018388-032018447 | PROMOTER | 0.914966149 | 8.851E-07 |
| 108078 | A_68_P30481643 | Accn2 | chr15:099500104-099500153 | INSIDE | 0.946264646 | 8.95E-07 |
| 22073 | A_68_P21666862 | Jag1 | chr2:136809559-136809618 | PROMOTER | 1.0099476 | 9.1E-07 |
| 20946 | A_68_P25188307 | Aldh1a3 | chr7:066309247-066309306 | PROMOTER | 1.089480139 | 9.136E-07 |
| 176266 | A_68_P21942775 | Snx16 | chr3:010425660-010425719 | PROMOTER | 1.508318899 | 9.7E-07 |
| 161246 | A_68_P29080232 | Agtpbp1 | chr13:059572126-059572183 | PROMOTER | 1.039392934 | 9.8E-07 |
| 238190 | A_68_P21732440 | Cst9 | chr2:148521979-148522038 | PROMOTER | 1.1624167 | 9.8E-07 |
| 49367 | A_68_P25725406 | Plekha2 | chr8:026570750-026570809 | PROMOTER | 0.878808999 | 9.95E-07 |
| 5045 | A_68_P26317479 | Jmjd2d | chr9:014249396-014249455 | INSIDE | 0.985784887 | 1.02E-06 |
| 88201 | A_68_P32095046 | Ide | chr19:037400747-037400806 | PROMOTER | 0.894223421 | 1.025E-06 |
| 133588 | A_68_P28071201 | Ptrf | chr11:100784353-100784405 | INSIDE | 1.041092982 | 1.03E-06 |
| 207267 | A_68_P20633046 | Tmem183a | chr1:136182672-136182731 | PROMOTER | 0.859489677 | 1.039E-06 |
| 154078 | A_68_P26426674 | Stt3a | chr9:036516266-036516325 | INSIDE | 1.164772621 | 1.05E-06 |
| 149585 | A_68_P32057208 | Mbl2 | chr19:030299405-030299464 | INSIDE | 1.047228058 | 1.07E-06 |
| 134847 | A_68_P30512773 | Mgrn1 | chr16:004853028-004853074 | INSIDE | 0.890983982 | 1.135E-06 |
| 40203 | A_68_P23249582 | Edn2 | chr4:119654847-119654906 | PROMOTER | 0.972292665 | 1.142E-06 |
| 130016 | A_68_P32193347 | Zdhhc6 | chr19:055365396-055365444 | INSIDE | 0.862206731 | 1.17E-06 |
| 169572 | A_68_P31178245 | Cenpq-Mut | chr17:040399965-040400024 | DIVERGENT_PROMOTER | 0.87682785 | 1.17E-06 |
| 53405 | A_68_P30485942 | Letmd1 | chr15:100296414-100296470 | PROMOTER | 1.487667863 | 1.215E-06 |
| 153754 | A_68_P26470851 | Cd3e | chr9:044759507-044759563 | INSIDE | 1.152966493 | 1.22E-06 |
| 8624 | A_68_P24486177 | Gprin3 | chr6:059357102-059357161 | PROMOTER | 1.164672289 | 1.29E-06 |
| 47403 | A_68_P27186939 | Ddx21 | chr10:062001692-062001751 | PROMOTER | 1.355638276 | 1.345E-06 |
| 134039 | A_68_P23269481 | Pou3f1 | chr4:124155200-124155254 | PROMOTER | 1.069535155 | 1.365E-06 |
| 218897 | A_68_P31187104 | Tnfrsf21 | chr17:042474535-042474586 | PROMOTER | 1.335763287 | 1.375E-06 |
| 94486 | A_68_P25956643 | Nr2f6 | chr8:074311920-074311967 | PROMOTER | 1.043497215 | 0.0000014 |
| 144023 | A_68_P32643824 | Gm382 | chrX:122505525-122505584 | PROMOTER | 0.965115743 | 1.4E-06 |
| 221355 | A_68_P31928866 | Ctsw | chr19:005467936-005467986 | INSIDE | 1.236010212 | 1.43E-06 |
| 183745 | A_68_P24828604 | Kcna6 | chr6:126704155-126704202 | INSIDE | 1.012740176 | 1.43E-06 |
| 207565 | A_68_P23537563 | Srpk2 | chr5:023133664-023133723 | PROMOTER | 0.999476375 | 1.445E-06 |
| 96961 | A_68_P26030513 | Lonp2 | chr8:089515421-089515480 | INSIDE | 1.331906882 | 1.5E-06 |
| 7865 | A_68_P24986981 | Ceacam11 | chr7:017128455-017128514 | PROMOTER | 0.906501813 | 1.535E-06 |
| 131825 | A_68_P30577213 | Dgcr6 | chr16:017965545-017965604 | PROMOTER | 1.151512301 | 1.54E-06 |
| 132815 | A_68_P24771672 | Tsen2 | chr6:115507878-115507937 | PROMOTER | 1.424595461 | 1.59E-06 |
| 85512 | A_68_P23415865 | Phf13 | chr4:150836965-150837024 | PROMOTER | 1.106744918 | 1.6E-06 |
| 111556 | A_68_P21614044 | Kcnip3 | chr2:127216761-127216816 | PROMOTER | 1.046201799 | 1.615E-06 |
| 57282 | A_68_P25939698 | Psd3 | chr8:070840731-070840786 | PROMOTER | 1.09305358 | 1.65E-06 |
| 9768 | A_68_P29617973 | Dhrs4 | chr14:054434247-054434292 | INSIDE | 1.002491712 | 1.655E-06 |
| 40874 | A_68_P28326647 | Tmem18 | chr12:031168873-031168931 | PROMOTER | 1.631306001 | 1.665E-06 |
| 53454 | A_68_P31537135 | Dsc3 | chr18:020148822-020148881 | PROMOTER | 0.984920483 | 1.665E-06 |
| 13392 | A_68_P24367067 | Chrm2 | chr6:036450543-036450602 | PROMOTER | 0.989935077 | 1.69E-06 |
| 46120 | A_68_P27081767 | BC021785 | chr10:039680795-039680849 | PROMOTER | 0.876313837 | 1.695E-06 |
| 218485 | A_68_P31095217 | Pdpk1 | chr17:023801855-023801914 | INSIDE | 1.097399108 | 1.725E-06 |
| 52262 | A_68_P31539536 | Dsg4 | chr18:020580513-020580570 | INSIDE | 1.20907274 | 1.775E-06 |
| 224044 | A_68_P23265203 | Mycbp | chr4:123410279-123410338 | INSIDE | 0.858431228 | 1.775E-06 |
| 144683 | A_68_P24777367 | Zfp422 | chr6:116598799-116598853 | PROMOTER | 0.987643691 | 1.85E-06 |
| 43496 | A_68_P21140433 | Olfr354 | chr2:036729574-036729630 | INSIDE | 0.887001169 | 1.865E-06 |
| 125989 | A_68_P28744672 | Eif5 | chr12:111989444-111989503 | INSIDE | 1.40969608 | 1.945E-06 |
| 75437 | A_68_P30728911 | Phldb2 | chr16:045762291-045762350 | INSIDE | 0.884699111 | 2.106E-06 |
| 172167 | A_68_P27377020 | Kera | chr10:097035672-097035731 | PROMOTER | 1.044438938 | 2.11E-06 |
| 26747 | A_68_P27784443 | Cnot6 | chr11:049554716-049554772 | INSIDE | 0.880783459 | 2.115E-06 |
| 22072 | A_68_P21405658 | Olfr1120 | chr2:087158226-087158285 | PROMOTER | 1.018417935 | 2.15E-06 |
| 15578 | A_68_P23970982 | Dr1 | chr5:108510362-108510416 | INSIDE | 1.120546444 | 2.245E-06 |
| 84560 | A_68_P27102654 | Rtn4ip1 | chr10:043585869-043585921 | PROMOTER | 0.858949978 | 2.245E-06 |
| 209569 | A_68_P22450150 | Sass6 | chr3:116585485-116585544 | PROMOTER | 0.872935518 | 2.246E-06 |
| 4983 | A_68_P26587617 | Ostb | chr9:065222281-065222336 | PROMOTER | 0.98251556 | 2.255E-06 |
| 68570 | A_68_P30146855 | Rrm2b | chr15:037909294-037909353 | PROMOTER | 0.970262474 | 2.28E-06 |
| 19106 | A_68_P20192545 | Kdelc1 | chr1:044052137-044052196 | INSIDE | 0.88497948 | 2.458E-06 |
| 78047 | A_68_P28060054 | Igfbp4 | chr11:098853299-098853358 | PROMOTER | 0.890429565 | 2.489E-06 |
| 48053 | A_68_P32825351 | Eif2s3y | chrY:000345336-000345395 | PROMOTER | 0.977534404 | 2.551E-06 |
| 180234 | A_68_P29232475 | Lhfpl2 | chr13:095157675-095157730 | PROMOTER | 0.86856978 | 2.62E-06 |
| 92041 | A_68_P23237404 | St3gal3 | chr4:117431182-117431234 | INSIDE | 0.900803553 | 2.66E-06 |
| 13092 | A_68_P22370662 | Man1a2 | chr3:100812613-100812672 | INSIDE | 0.940993001 | 2.676E-06 |
| 227559 | A_68_P20304065 | Rpe | chr1:066630281-066630340 | PROMOTER | 1.038133976 | 2.813E-06 |
| 91238 | A_68_P26392285 | Hnt | chr9:029713788-029713842 | PROMOTER | 0.972392439 | 2.845E-06 |
| 3206 | A_68_P24476808 | Kbtbd2 | chr6:056710757-056710816 | PROMOTER | 1.108581314 | 2.9E-06 |
| 96184 | A_68_P29230414 | Bhmt2 | chr13:094772867-094772926 | INSIDE | 1.103105667 | 2.94E-06 |
| 14021 | A_68_P31169349 | Olfr115 | chr17:037219842-037219901 | PROMOTER | 1.235914716 | 2.99E-06 |
| 153886 | A_68_P26239258 | Fanca-Spire2 | chr8:126214069-126214126 | DIVERGENT_PROMOTER | 0.975516498 | 3.095E-06 |
| 105792 | A_68_P27273726 | Agpat3 | chr10:077754670-077754729 | INSIDE | 0.952954572 | 3.14E-06 |
| 202680 | A_68_P32016059 | Klf9 | chr19:023204849-023204908 | PROMOTER | 1.067059306 | 3.185E-06 |
| 99750 | A_68_P32119352 | Marveld1 | chr19:042211557-042211611 | DOWNSTREAM | 1.19556782 | 3.24E-06 |
| 180258 | A_68_P31296445 | Man2a1 | chr17:064494651-064494710 | PROMOTER | 0.923385457 | 3.27E-06 |
| 208051 | A_68_P23392723 | Fbxo44 | chr4:146999857-146999912 | INSIDE | 1.064414819 | 3.355E-06 |
| 124305 | A_68_P26560503 | Larp6 | chr9:060512720-060512771 | INSIDE | 1.183797847 | 0.0000034 |
| 76499 | A_68_P24195261 | Stard13 | chr5:151460755-151460814 | PROMOTER | 1.258468255 | 3.51E-06 |
| 79709 | A_68_P23194812 | Echdc2 | chr4:107663768-107663812 | INSIDE | 1.122380295 | 0.0000039 |
| 63979 | A_68_P29145370 | Ahrr | chr13:074756115-074756167 | INSIDE | 0.895869116 | 3.91E-06 |
| 43886 | A_68_P28223205 | Slc7a15 | chr12:008566357-008566416 | PROMOTER | 1.059139421 | 3.92E-06 |
| 189877 | A_68_P30393140 | Samm50 | chr15:084018878-084018937 | PROMOTER | 0.963819086 | 3.98E-06 |
| 183259 | A_68_P25501458 | Nupr1 | chr7:126419398-126419457 | PROMOTER | 0.959765865 | 4.04E-06 |
| 100968 | A_68_P27863357 | Cox10 | chr11:063896504-063896563 | PROMOTER | 1.153957603 | 4.285E-06 |
| 32328 | A_68_P21642516 | Crls1 | chr2:132537783-132537842 | PROMOTER | 1.035371127 | 4.41E-06 |
| 183711 | A_68_P24973502 | Zscan22 | chr7:011796764-011796818 | PROMOTER | 1.109012862 | 4.415E-06 |
| 154655 | A_68_P25508431 | Fus | chr7:127772156-127772215 | INSIDE | 1.005644639 | 4.465E-06 |
| 109193 | A_68_P23244570 | Cldn19 | chr4:118755897-118755955 | INSIDE | 1.032274314 | 4.495E-06 |
| 87032 | A_68_P23223350 | Pdzk1ip1 | chr4:114585473-114585528 | PROMOTER | 1.234138648 | 4.635E-06 |
| 207986 | A_68_P24170903 | Gtf3a | chr5:147253659-147253707 | PROMOTER | 0.902161425 | 4.805E-06 |
| 150260 | A_68_P25367899 | AU020772 | chr7:100342673-100342720 | INSIDE | 0.877821519 | 4.84E-06 |
| 113722 | A_68_P26469694 | BC021608 | chr9:044559043-044559094 | PROMOTER | 1.006172194 | 5.05E-06 |
| 13577 | A_68_P28149422 | Dnaic2 | chr11:114546891-114546947 | INSIDE | 1.045457536 | 5.25E-06 |
| 152234 | A_68_P26756465 | Clstn2 | chr9:097846049-097846108 | PROMOTER | 0.883290617 | 5.25E-06 |
| 163287 | A_68_P22294039 | Fbxw7 | chr3:085037196-085037255 | PROMOTER | 0.962534962 | 5.28E-06 |
| 86028 | A_68_P30453197 | Twf1 | chr15:094416914-094416973 | INSIDE | 0.971913402 | 5.445E-06 |
| 107624 | A_68_P28614762 | Tmed8 | chr12:088090855-088090912 | PROMOTER | 1.181425419 | 5.5E-06 |
| 4972 | A_68_P28063045 | Krtap3-3 | chr11:099367491-099367550 | PROMOTER | 0.977239242 | 5.575E-06 |
| 144913 | A_68_P31020000 | Snx9 | chr17:005896694-005896738 | DOWNSTREAM | 1.069971137 | 5.85E-06 |
| 179601 | A_68_P27687518 | Rhbdf1-Mpg | chr11:032124352-032124402 | DIVERGENT_PROMOTER | 0.963743077 | 6.05E-06 |
| 109778 | A_68_P25402175 | Olfr480 | chr7:107858358-107858417 | PROMOTER | 0.86520686 | 6.119E-06 |
| 184956 | A_68_P25259463 | Mfge8 | chr7:079028025-079028075 | PROMOTER | 0.926141475 | 6.2E-06 |
| 78587 | A_68_P21568411 | Zfyve19 | chr2:118902702-118902746 | INSIDE | 1.012156492 | 6.55E-06 |
| 217700 | A_68_P27862284 | Hs3st3b1 | chr11:063736665-063736714 | INSIDE | 0.894170616 | 6.55E-06 |
| 90125 | A_68_P21828034 | Sulf2 | chr2:165847011-165847057 | PROMOTER | 1.038533317 | 0.0000066 |
| 123445 | A_68_P29387737 | Pxk | chr14:006886093-006886152 | PROMOTER | 0.914004211 | 6.65E-06 |
| 31858 | A_68_P21132670 | Gsn | chr2:035103384-035103437 | PROMOTER | 1.180106015 | 6.75E-06 |
| 101050 | A_68_P27543368 | Ormdl2 | chr10:128223504-128223563 | INSIDE | 0.928053954 | 6.85E-06 |
| 20643 | A_68_P27547865 | Olfr818 | chr10:129350274-129350333 | PROMOTER | 0.910218539 | 7.05E-06 |
| 45512 | A_68_P25467414 | Zp2 | chr7:119937128-119937187 | PROMOTER | 1.499533799 | 0.0000072 |
| 189870 | A_68_P28194553 | Rab10 | chr12:003311299-003311358 | PROMOTER | 1.106055106 | 0.0000077 |
| 161468 | A_68_P20439222 | Mlph | chr1:092747638-092747688 | INSIDE | 0.957185182 | 0.0000079 |
| 125013 | A_68_P27610142 | Vstm2 | chr11:016153988-016154036 | PROMOTER | 0.896406229 | 7.95E-06 |
| 73963 | A_68_P21975403 | Dnajc5b | chr3:019698797-019698853 | PROMOTER | 1.005948845 | 7.95E-06 |
| 15729 | A_68_P30010864 | Rad1 | chr15:010435477-010435536 | INSIDE | 1.207868732 | 8.25E-06 |
| 199416 | A_68_P21403595 | Olfr1093 | chr2:086584585-086584644 | PROMOTER | 1.011012985 | 8.319E-06 |
| 187037 | A_68_P26512447 | Fdx1 | chr9:051719516-051719573 | PROMOTER | 1.30633042 | 8.45E-06 |
| 155199 | A_68_P28067141 | Krt42 | chr11:100085009-100085068 | INSIDE | 1.111038682 | 8.6E-06 |
| 131436 | A_68_P26242968 | AK122209 | chr8:126911248-126911307 | INSIDE | 1.148740349 | 8.75E-06 |
| 233948 | A_68_P22488138 | Prss12 | chr3:123439803-123439862 | INSIDE | 1.091792852 | 0.0000089 |
| 1264 | A_68_P20970765 | Sephs1 | chr2:004798246-004798305 | PROMOTER | 1.31555692 | 0.000009 |
| 230039 | A_68_P23760665 | Nsun7 | chr5:066536330-066536389 | PROMOTER | 0.862272094 | 9.65E-06 |
| 121957 | A_68_P26665733 | Col12a1 | chr9:079508097-079508152 | PROMOTER | 0.931323074 | 9.9E-06 |
| 175145 | A_68_P26528704 | Chrna5 | chr9:054775258-054775317 | PROMOTER | 1.015886599 | 9.9E-06 |
| 5775 | A_68_P22199746 | Ssr3 | chr3:065484451-065484502 | PROMOTER | 0.877727564 | 9.95E-06 |
| 96685 | A_68_P24759886 | Prrt3 | chr6:113466572-113466624 | INSIDE | 0.965106728 | 1.015E-05 |
| 104057 | A_68_P29715854 | Rcbtb2 | chr14:071872614-071872673 | PROMOTER | 1.609313723 | 0.0000102 |
| 24399 | A_68_P25411552 | Zfp143 | chr7:109853579-109853638 | INSIDE | 0.905146153 | 1.024E-05 |
| 22833 | A_68_P23644461 | Cpeb2 | chr5:043524697-043524756 | INSIDE | 0.864041306 | 1.055E-05 |
| 240412 | A_68_P28127598 | Kcnj2 | chr11:110877391-110877450 | PROMOTER | 1.032990776 | 1.06E-05 |
| 163298 | A_68_P20441538 | Scly | chr1:093130027-093130084 | INSIDE | 0.898383892 | 1.07E-05 |
| 85932 | A_68_P23893826 | Cxcl11 | chr5:093440486-093440545 | PROMOTER | 0.946665694 | 1.092E-05 |
| 116713 | A_68_P22415307 | Prpf38b | chr3:109044395-109044452 | PROMOTER | 0.955566289 | 1.115E-05 |
| 74137 | A_68_P29510547 | Sema3g | chr14:030048851-030048910 | INSIDE | 1.176842261 | 1.13E-05 |
| 146859 | A_68_P32651766 | Diap2 | chrX:125091302-125091361 | PROMOTER | 0.87397866 | 0.0000115 |
| 18686 | A_68_P26287701 | Mmp12 | chr9:007344424-007344483 | PROMOTER | 0.956979327 | 0.0000116 |
| 72606 | A_68_P26680211 | Irak1bp1 | chr9:082625022-082625081 | PROMOTER | 0.971152358 | 1.19E-05 |
| 113428 | A_68_P24170294 | Rpl21 | chr5:147140267-147140326 | PROMOTER | 1.237129234 | 0.0000122 |
| 163249 | A_68_P21748549 | Tbc1d20 | chr2:151984445-151984504 | PROMOTER | 0.94576932 | 1.231E-05 |
| 19873 | A_68_P23920306 | Bmp3 | chr5:099090745-099090804 | PROMOTER | 0.958116781 | 1.273E-05 |
| 162395 | A_68_P25667177 | Adprhl1 | chr8:013259209-013259268 | PROMOTER | 1.062580597 | 1.285E-05 |
| 98878 | A_68_P29507756 | Sfmbt1 | chr14:029590928-029590987 | PROMOTER | 1.196175419 | 1.345E-05 |
| 20322 | A_68_P22446808 | Slc30a7 | chr3:115997690-115997749 | INSIDE | 1.089564907 | 1.365E-05 |
| 152474 | A_68_P23295139 | Fndc5 | chr4:128638565-128638615 | PROMOTER | 0.991122281 | 0.0000137 |
| 26758 | A_68_P29361863 | Emb | chr13:118336111-118336170 | PROMOTER | 0.989922887 | 1.4E-05 |
| 221756 | A_68_P30359680 | Tst | chr15:078226289-078226333 | DOWNSTREAM | 0.927501695 | 1.405E-05 |
| 126538 | A_68_P20422199 | Dnajb3-Ugt1a1 | chr1:090042776-090042835 | DIVERGENT_PROMOTER | 1.08964676 | 0.0000142 |
| 68917 | A_68_P28613628 | Tmem63c | chr12:087917992-087918050 | INSIDE | 1.409954666 | 1.505E-05 |
| 64500 | A_68_P27996763 | Epx | chr11:087693464-087693515 | PROMOTER | 0.906747062 | 0.0000151 |
| 29079 | A_68_P31101040 | Ift140 | chr17:024748060-024748115 | INSIDE | 1.345797557 | 0.0000151 |
| 68125 | A_68_P21550205 | Mrg1 | chr2:115760725-115760784 | PROMOTER | 0.866852561 | 0.0000154 |
| 186377 | A_68_P32506222 | Map3k7ip3 | chrX:081828056-081828115 | INSIDE | 0.861973844 | 1.552E-05 |
| 233863 | A_68_P24019907 | BC023744 | chr5:117578309-117578368 | PROMOTER | 1.298515525 | 0.0000156 |
| 223771 | A_68_P28574328 | Actn1 | chr12:081107155-081107214 | INSIDE | 0.864633321 | 1.655E-05 |
| 110280 | A_68_P24971050 | V1rg7 | chr7:011059192-011059248 | INSIDE | 0.976366082 | 1.665E-05 |
| 129472 | A_68_P20759134 | Rfwd2 | chr1:161067274-161067333 | PROMOTER | 1.000270281 | 1.665E-05 |
| 77638 | A_68_P26470991 | Eva1 | chr9:044790413-044790472 | PROMOTER | 0.903093954 | 0.0000171 |
| 78137 | A_68_P24993670 | Bloc1s3-Trappc6a | chr7:018666977-018667036 | DIVERGENT_PROMOTER | 0.94734606 | 1.734E-05 |
| 176485 | A_68_P24644024 | Trh | chr6:092211247-092211295 | PROMOTER | 0.898297787 | 1.75E-05 |
| 170151 | A_68_P21817116 | Svp2-Svs3 | chr2:163975952-163976010 | DIVERGENT_PROMOTER | 1.044645228 | 0.000018 |
| 95930 | A_68_P24467542 | Crhr2 | chr6:055067572-055067624 | PROMOTER | 0.972835699 | 0.0000186 |
| 186014 | A_68_P28952468 | F13a1 | chr13:037055325-037055384 | INSIDE | 1.33077862 | 0.0000186 |
| 211704 | A_68_P27547445 | Olfr812 | chr10:129243634-129243693 | DOWNSTREAM | 1.063456365 | 0.0000189 |
| 194700 | A_68_P30089755 | Fbxl7 | chr15:026838548-026838607 | INSIDE | 0.865472828 | 1.891E-05 |
| 208146 | A_68_P23967762 | Glmn | chr5:107833896-107833955 | INSIDE | 0.925005399 | 1.905E-05 |
| 66195 | A_68_P21061703 | Mastl | chr2:022976855-022976914 | INSIDE | 1.522262873 | 0.0000191 |
| 223630 | A_68_P32252906 | Mid1ip1 | chrX:009876490-009876549 | INSIDE | 1.070259311 | 1.925E-05 |
| 158733 | A_68_P24207301 | Ppp1r9a | chr6:004848415-004848474 | PROMOTER | 1.228518711 | 1.925E-05 |
| 185006 | A_68_P22408072 | Ahcyl1 | chr3:107826241-107826300 | PROMOTER | 0.987915832 | 1.955E-05 |
| 238707 | A_68_P31170909 | Olfr129-Olfr130 | chr17:037667804-037667854 | DIVERGENT_PROMOTER | 0.916484846 | 2.025E-05 |
| 137022 | A_68_P22449728 | Rtcd1-Dbt | chr3:116503870-116503929 | DIVERGENT_PROMOTER | 0.904040843 | 2.094E-05 |
| 40123 | A_68_P31177502 | Rhag | chr17:040271797-040271856 | PROMOTER | 1.3303975 | 0.0000214 |
| 16225 | A_68_P27306511 | AI597468 | chr10:084535374-084535433 | INSIDE | 1.07989153 | 2.191E-05 |
| 120135 | A_68_P25654543 | Col4a1 | chr8:011307618-011307672 | INSIDE | 0.873822843 | 0.0000223 |
| 71962 | A_68_P30137120 | Spag1 | chr15:036126495-036126551 | INSIDE | 1.046598771 | 0.0000223 |
| 13050 | A_68_P31910805 | Cd226 | chr18:089333133-089333192 | INSIDE | 1.134712254 | 2.265E-05 |
| 173273 | A_68_P21750085 | Defb27 | chr2:152287508-152287567 | INSIDE | 1.027435693 | 2.335E-05 |
| 78158 | A_68_P32683825 | BC031748 | chrX:132391381-132391440 | PROMOTER | 0.954817558 | 2.355E-05 |
| 142589 | A_68_P25051016 | Dpy19l3 | chr7:035464468-035464527 | PROMOTER | 1.333440416 | 0.0000239 |
| 105948 | A_68_P30499403 | Calcoco1 | chr15:102552828-102552883 | PROMOTER | 0.960108898 | 0.000024 |
| 40107 | A_68_P27552419 | Selm | chr11:003415746-003415795 | INSIDE | 0.953638109 | 2.475E-05 |
| 232474 | A_68_P31247164 | Slc5a7 | chr17:053777902-053777958 | PROMOTER | 1.038019694 | 2.535E-05 |
| 201506 | A_68_P24518593 | Gng12 | chr6:066822293-066822352 | PROMOTER | 0.984402818 | 2.541E-05 |
| 181146 | A_68_P27554708 | Tcn2 | chr11:003817695-003817743 | INSIDE | 0.887097371 | 0.0000256 |
| 97556 | A_68_P23546070 | Galntl5 | chr5:024693992-024694051 | INSIDE | 1.368936015 | 2.565E-05 |
| 17803 | A_68_P32563384 | Nlgn3 | chrX:097501053-097501112 | PROMOTER | 0.870558728 | 2.585E-05 |
| 104653 | A_68_P23966408 | Brdt | chr5:107573723-107573782 | INSIDE | 1.126724165 | 0.0000267 |
| 232253 | A_68_P24845270 | Klri2 | chr6:129706959-129707018 | PROMOTER | 0.971317882 | 2.715E-05 |
| 62472 | A_68_P30624720 | Ostn | chr16:027221535-027221590 | PROMOTER | 0.897339151 | 0.0000273 |
| 199019 | A_68_P20285552 | Gpr1 | chr1:063116281-063116340 | DOWNSTREAM | 0.883049496 | 2.875E-05 |
| 17795 | A_68_P26699991 | Pgm3 | chr9:086365723-086365772 | INSIDE | 1.283695705 | 0.0000288 |
| 152576 | A_68_P25378982 | Olfr543 | chr7:102351530-102351586 | INSIDE | 0.944571438 | 2.965E-05 |
| 112999 | A_68_P28909004 | Sox4 | chr13:028964510-028964569 | PROMOTER | 1.208768975 | 0.0000298 |
| 52444 | A_68_P23954954 | EG634650 | chr5:105591310-105591361 | PROMOTER | 0.902828871 | 0.0000307 |
| 101152 | A_68_P30425983 | Acr | chr15:089395831-089395880 | PROMOTER | 0.978685706 | 3.075E-05 |
| 191129 | A_68_P20962326 | Meig1 | chr2:003339874-003339933 | INSIDE | 1.020831041 | 3.105E-05 |
| 212134 | A_68_P25702170 | EG546038 | chr8:019142972-019143031 | DOWNSTREAM | 1.212039054 | 3.165E-05 |
| 182150 | A_68_P28859774 | Amph | chr13:018957605-018957664 | INSIDE | 0.971173364 | 3.175E-05 |
| 109397 | A_68_P21584672 | B2m | chr2:121835575-121835633 | PROMOTER | 1.1874804 | 0.0000321 |
| 191693 | A_68_P24355894 | Bpgm | chr6:034405486-034405544 | PROMOTER | 1.097153512 | 3.215E-05 |
| 42694 | A_68_P25730801 | Hook3 | chr8:027582434-027582493 | INSIDE | 1.083764908 | 3.305E-05 |
| 61766 | A_68_P24842689 | Cd69 | chr6:129242310-129242367 | PROMOTER | 0.912267547 | 3.355E-05 |
| 191699 | A_68_P28877817 | V1ri4 | chr13:022483526-022483585 | DOWNSTREAM | 1.489472033 | 3.385E-05 |
| 137827 | A_68_P24264416 | Tcfec | chr6:016852139-016852198 | PROMOTER | 0.975727675 | 3.554E-05 |
| 18324 | A_68_P27856240 | Fam18b | chr11:062697528-062697579 | INSIDE | 0.923611355 | 3.565E-05 |
| 83735 | A_68_P27322171 | Igf1 | chr10:087286032-087286091 | PROMOTER | 1.052687869 | 0.0000357 |
| 220303 | A_68_P32062134 | Cstf2t | chr19:031147508-031147567 | PROMOTER | 0.923470521 | 3.593E-05 |
| 171772 | A_68_P26905194 | Mtrf1l | chr10:004517906-004517965 | PROMOTER | 1.191944948 | 0.0000361 |
| 135965 | A_68_P26293530 | Pgr | chr9:008860110-008860169 | PROMOTER | 1.023186842 | 0.0000374 |
| 208656 | A_68_P20618254 | Nucks1 | chr1:133736910-133736969 | PROMOTER | 1.224231627 | 0.000038 |
| 130180 | A_68_P28025162 | Car10 | chr11:092918596-092918655 | INSIDE | 1.255128424 | 0.0000389 |
| 149298 | A_68_P31659133 | Ppp2r2b | chr18:043184197-043184256 | INSIDE | 1.146734781 | 3.91E-05 |
| 195311 | A_68_P21478569 | Slc1a2 | chr2:102503557-102503616 | PROMOTER | 0.891232628 | 0.0000399 |
| 9852 | A_68_P29689065 | Nefl | chr14:067036224-067036271 | PROMOTER | 0.881459108 | 4.01E-05 |
| 126626 | A_68_P21940389 | Fabp5 | chr3:009996299-009996355 | INSIDE | 0.918719487 | 4.045E-05 |
| 185482 | A_68_P29567889 | Ptgdr | chr14:043780534-043780585 | INSIDE | 1.220847096 | 4.045E-05 |
| 96198 | A_68_P25960699 | Klf2 | chr8:075250095-075250151 | INSIDE | 1.012054082 | 4.055E-05 |
| 96042 | A_68_P29222301 | Rasgrf2 | chr13:093233525-093233580 | PROMOTER | 1.075069464 | 4.065E-05 |
| 223067 | A_68_P20457743 | Neu4 | chr1:095846193-095846252 | PROMOTER | 1.117614086 | 4.175E-05 |
| 143436 | A_68_P26349147 | EG624219 | chr9:021580943-021580999 | PROMOTER | 1.018573284 | 4.195E-05 |
| 122434 | A_68_P26586459 | Parp16 | chr9:065008507-065008563 | PROMOTER | 0.895295969 | 4.215E-05 |
| 155323 | A_68_P30929929 | Cyyr1 | chr16:085437168-085437221 | INSIDE | 1.030761205 | 0.0000423 |
| 161250 | A_68_P31178206 | Cenpq | chr17:040395044-040395103 | INSIDE | 1.013277001 | 0.0000443 |
| 108046 | A_68_P21395369 | Slc43a3 | chr2:084732219-084732278 | PROMOTER | 0.867515739 | 4.51E-05 |
| 2106 | A_68_P32543260 | Las1l | chrX:092160155-092160214 | PROMOTER | 1.12788533 | 0.0000453 |
| 108159 | A_68_P23601213 | Cpz | chr5:035846750-035846804 | PROMOTER | 1.002355378 | 0.0000458 |
| 34468 | A_68_P31163349 | Trim39 | chr17:035868496-035868555 | INSIDE | 0.940374638 | 0.0000458 |
| 179505 | A_68_P23269852 | Sf3a3 | chr4:124213531-124213590 | PROMOTER | 1.614801009 | 0.0000463 |
| 51044 | A_68_P24931605 | Ppfibp1 | chr6:146915343-146915397 | INSIDE | 1.101844278 | 4.805E-05 |
| 214168 | A_68_P23231000 | Tesk2 | chr4:116301286-116301337 | INSIDE | 0.928931882 | 0.0000485 |
| 181562 | A_68_P30621558 | Il1rap | chr16:026499088-026499147 | INSIDE | 0.949946886 | 0.0000488 |
| 99658 | A_68_P22485839 | Myoz2 | chr3:123031059-123031112 | PROMOTER | 1.029410734 | 0.0000492 |
| 187730 | A_68_P29152763 | Gpr150 | chr13:076523716-076523775 | PROMOTER | 0.943493858 | 5.236E-05 |
| 133021 | A_68_P25402974 | Olfr491 | chr7:108107102-108107161 | PROMOTER | 0.991503032 | 5.35E-05 |
| 201748 | A_68_P25494901 | Nsmce1 | chr7:125280769-125280828 | INSIDE | 0.8868279 | 5.351E-05 |
| 119530 | A_68_P21568755 | Rhov | chr2:118967460-118967519 | PROMOTER | 0.949283232 | 5.352E-05 |
| 205229 | A_68_P28161626 | Sfrs2 | chr11:116667056-116667115 | INSIDE | 0.860350489 | 5.559E-05 |
| 148461 | A_68_P31964162 | Olfr1466 | chr19:013407196-013407255 | PROMOTER | 0.944937531 | 5.623E-05 |
| 197919 | A_68_P28565689 | Mpp5 | chr12:079664236-079664295 | PROMOTER | 1.29526898 | 5.739E-05 |
| 12341 | A_68_P31778620 | Nedd4l | chr18:065149941-065149998 | INSIDE | 0.999822361 | 5.823E-05 |
| 53700 | A_68_P32804781 | Gemin8 | chrX:161513903-161513962 | PROMOTER | 0.909330454 | 6.075E-05 |
| 184949 | A_68_P30179425 | Trhr | chr15:044023784-044023835 | PROMOTER | 0.918860776 | 6.116E-05 |
| 6418 | A_68_P28958189 | Cage1-Riok1 | chr13:038041233-038041292 | DIVERGENT_PROMOTER | 1.123386356 | 6.203E-05 |
| 129501 | A_68_P20509903 | Bcl2 | chr1:108538280-108538339 | INSIDE | 0.915062581 | 6.308E-05 |
| 56188 | A_68_P22889019 | Nans | chr4:046510204-046510262 | PROMOTER | 1.297190419 | 6.52E-05 |
| 120770 | A_68_P31791026 | Impa2 | chr18:067415748-067415807 | INSIDE | 1.218014927 | 6.579E-05 |
| 199938 | A_68_P28835573 | Gng4 | chr13:013571027-013571080 | PROMOTER | 0.956735823 | 6.583E-05 |
| 145022 | A_68_P26263537 | Pard3 | chr8:130324465-130324524 | INSIDE | 0.975514955 | 6.744E-05 |
| 216865 | A_68_P32006556 | Gda | chr19:021538743-021538802 | INSIDE | 0.97927743 | 6.778E-05 |
| 138096 | A_68_P28190982 | Zfp750 | chr11:121334512-121334567 | INSIDE | 0.874633826 | 6.991E-05 |
| 12794 | A_68_P24477821 | V1rc32 | chr6:056961674-056961733 | PROMOTER | 1.310154017 | 7E-05 |
| 74533 | A_68_P28883327 | Hist1h4b | chr13:023763063-023763122 | PROMOTER | 1.383260338 | 7.024E-05 |
| 112422 | A_68_P21868006 | Pck1 | chr2:172794305-172794364 | PROMOTER | 1.079936919 | 7.083E-05 |
| 173416 | A_68_P24157338 | Pscd3 | chr5:143879256-143879312 | INSIDE | 0.897454048 | 7.171E-05 |
| 177079 | A_68_P23391662 | Nppb | chr4:146825752-146825802 | PROMOTER | 0.940507964 | 7.172E-05 |
| 42838 | A_68_P26436164 | Olfr922 | chr9:038565638-038565697 | PROMOTER | 1.026503272 | 7.251E-05 |
| 134705 | A_68_P26168110 | Fa2h | chr8:114282768-114282826 | PROMOTER | 0.902197933 | 7.58E-05 |
| 24076 | A_68_P25645851 | Lig4 | chr8:009979655-009979714 | PROMOTER | 1.439764519 | 7.751E-05 |
| 173764 | A_68_P28091906 | Itgb3 | chr11:104425840-104425898 | INSIDE | 1.036116285 | 7.8E-05 |
| 114266 | A_68_P29659626 | Fdft1 | chr14:062131013-062131072 | INSIDE | 0.96492125 | 8.057E-05 |
| 139452 | A_68_P29988336 | C9 | chr15:006393932-006393989 | INSIDE | 0.892422925 | 8.161E-05 |
| 97867 | A_68_P22373294 | Ptgfrn | chr3:101242322-101242381 | PROMOTER | 1.234160193 | 8.199E-05 |
| 87631 | A_68_P20599275 | R3hdm1 | chr1:130031897-130031956 | INSIDE | 0.859940863 | 8.337E-05 |
| 102880 | A_68_P25368710 | Mrpl48 | chr7:100486171-100486230 | PROMOTER | 0.927719076 | 8.411E-05 |
| 113998 | A_68_P26233564 | Snai3 | chr8:125352001-125352048 | PROMOTER | 1.098597839 | 8.685E-05 |
| 153346 | A_68_P24357871 | Agbl3 | chr6:034709752-034709811 | PROMOTER | 0.99083661 | 8.724E-05 |
| 184895 | A_68_P26466797 | Abcg4 | chr9:044040449-044040508 | PROMOTER | 1.242538384 | 8.984E-05 |
| 152052 | A_68_P22829968 | Spaca1 | chr4:034377496-034377555 | INSIDE | 1.274689395 | 9.033E-05 |
| 37899 | A_68_P32696412 | Morc4 | chrX:135221221-135221280 | PROMOTER | 0.953596048 | 9.217E-05 |
| 138139 | A_68_P25023742 | Hnrpl | chr7:028529039-028529094 | INSIDE | 0.963529976 | 9.423E-05 |
| 209013 | A_68_P26591039 | Snx22 | chr9:065872112-065872170 | PROMOTER | 0.978975417 | 9.478E-05 |
| 18063 | A_68_P31379824 | Arl6ip2 | chr17:079767444-079767503 | INSIDE | 1.100810266 | 9.598E-05 |
| 206718 | A_68_P27847137 | Aldh3a1 | chr11:061023803-061023859 | PROMOTER | 1.022454084 | 9.649E-05 |
| 185547 | A_68_P23230446 | Prdx1 | chr4:116184956-116185015 | INSIDE | 0.929935761 | 9.827E-05 |
| 238383 | A_68_P28514153 | Arf6 | chr12:070285751-070285810 | PROMOTER | 0.995236791 | 9.83E-05 |
| 43475 | A_68_P30395507 | Ldoc1l | chr15:084383686-084383744 | INSIDE | 0.972561607 | 9.853E-05 |
| 191902 | A_68_P29702579 | Epb4.9 | chr14:069368913-069368968 | PROMOTER | 0.869708415 | 9.946E-05 |
| 117808 | A_68_P30794423 | Olfr193 | chr16:059061375-059061434 | PROMOTER | 0.912555562 | 0.0001025 |
| 26250 | A_68_P27354587 | Ccdc38 | chr10:093023452-093023511 | DOWNSTREAM | 1.006152277 | 0.0001038 |
| 168770 | A_68_P23367594 | Tmem51 | chr4:141355759-141355814 | INSIDE | 1.091884028 | 0.0001046 |
| 55506 | A_68_P25049956 | Nudt19 | chr7:035265891-035265950 | PROMOTER | 0.913895184 | 0.000108 |
| 156479 | A_68_P27452750 | Caps2 | chr10:111563524-111563582 | PROMOTER | 1.009825897 | 0.00011 |
| 173334 | A_68_P23873929 | Dck | chr5:089841826-089841881 | INSIDE | 0.902229911 | 0.0001112 |
| 112035 | A_68_P24810163 | Slc2a3 | chr6:122706108-122706157 | INSIDE | 0.983690816 | 0.000112 |
| 86430 | A_68_P31208891 | Rpl7l1 | chr17:046244888-046244947 | INSIDE | 0.971319762 | 0.0001124 |
| 129513 | A_68_P27554397 | Osbp2 | chr11:003762005-003762064 | INSIDE | 1.081176956 | 0.0001177 |
| 146674 | A_68_P23242371 | Olfr1333 | chr4:118326184-118326241 | DOWNSTREAM | 0.910035794 | 0.000118 |
| 5086 | A_68_P26465726 | C1qtnf5 | chr9:043856183-043856227 | PROMOTER | 0.995204131 | 0.0001185 |
| 114656 | A_68_P21741405 | Abhd12 | chr2:150539259-150539318 | PROMOTER | 0.977155989 | 0.00012 |
| 93761 | A_68_P27180366 | Nodal | chr10:060808400-060808459 | PROMOTER | 0.928216607 | 0.0001216 |
| 82903 | A_68_P27831440 | Olfr330 | chr11:058346933-058346992 | PROMOTER | 1.014733069 | 0.0001226 |
| 159600 | A_68_P21245569 | Pscdbp | chr2:057978946-057979004 | PROMOTER | 0.929557096 | 0.0001234 |
| 152936 | A_68_P23541284 | Nos3 | chr5:023875409-023875468 | INSIDE | 1.063646876 | 0.0001282 |
| 143828 | A_68_P21148594 | Nek6 | chr2:038335233-038335289 | INSIDE | 0.967678727 | 0.0001297 |
| 110653 | A_68_P30501457 | Hoxc5 | chr15:102842740-102842799 | INSIDE | 0.932187962 | 0.0001344 |
| 49531 | A_68_P23571077 | Hadhb | chr5:030487629-030487688 | INSIDE | 0.872089053 | 0.0001348 |
| 211166 | A_68_P32105643 | Cyp2c38 | chr19:039516112-039516171 | INSIDE | 1.157433285 | 0.0001352 |
| 106140 | A_68_P26744839 | Xrn1 | chr9:095765545-095765604 | INSIDE | 0.861024267 | 0.0001357 |
| 12698 | A_68_P21897002 | Cdh4 | chr2:179368190-179368242 | PROMOTER | 1.070321376 | 0.0001364 |
| 105944 | A_68_P31062754 | Wdr27 | chr17:014677338-014677397 | INSIDE | 1.018599929 | 0.0001366 |
| 6062 | A_68_P29659745 | Neil2 | chr14:062149523-062149582 | PROMOTER | 0.999375075 | 0.0001429 |
| 82651 | A_68_P24091271 | Vkorc1l1 | chr5:130222998-130223057 | PROMOTER | 1.134659802 | 0.0001452 |
| 121673 | A_68_P28100476 | Ftsj3 | chr11:106069291-106069350 | INSIDE | 1.113940997 | 0.0001454 |
| 210300 | A_68_P26035055 | Cbln1 | chr8:090365618-090365677 | PROMOTER | 1.093471871 | 0.0001458 |
| 121724 | A_68_P31630215 | Pcdhgc5 | chr18:037946190-037946239 | INSIDE | 0.917176792 | 0.0001463 |
| 197925 | A_68_P28582813 | Map3k9 | chr12:082700455-082700511 | PROMOTER | 1.022296712 | 0.0001474 |
| 18136 | A_68_P31771014 | Txnl1 | chr18:063818294-063818349 | PROMOTER | 0.989194785 | 0.0001478 |
| 144430 | A_68_P24060081 | Pitpnm2 | chr5:124478399-124478450 | PROMOTER | 1.353720373 | 0.0001503 |
| 49573 | A_68_P29650871 | Trim13 | chr14:060552565-060552624 | PROMOTER | 0.97151909 | 0.0001543 |
| 231292 | A_68_P24222167 | Asns | chr6:007647542-007647601 | PROMOTER | 1.092610235 | 0.0001577 |
| 141980 | A_68_P25503053 | Kctd13 | chr7:126721237-126721296 | INSIDE | 1.263784592 | 0.0001617 |
| 225873 | A_68_P32669415 | Tspan6 | chrX:129242913-129242972 | INSIDE | 1.060047145 | 0.0001623 |
| 34140 | A_68_P22332645 | Sprrl7 | chr3:092772152-092772209 | INSIDE | 0.906187177 | 0.0001623 |
| 119144 | A_68_P30649793 | Mfi2 | chr16:031795204-031795263 | PROMOTER | 1.496788895 | 0.0001643 |
| 61257 | A_68_P28051875 | Mllt6 | chr11:097476808-097476865 | PROMOTER | 0.982401299 | 0.0001655 |
| 3986 | A_68_P22163448 | Tm4sf4 | chr3:057508585-057508644 | PROMOTER | 1.331296536 | 0.0001741 |
| 128884 | A_68_P30685170 | Nr1i2 | chr16:038212966-038213024 | INSIDE | 0.898126942 | 0.0001764 |
| 129048 | A_68_P24421978 | Cntnap2 | chr6:047173214-047173273 | PROMOTER | 1.056210047 | 0.0001921 |
| 186921 | A_68_P21371780 | Pde1a | chr2:079707279-079707338 | INSIDE | 0.91294309 | 0.0002002 |
| 178651 | A_68_P24861372 | Bcl2l14 | chr6:134361237-134361292 | PROMOTER | 1.022842458 | 0.0002013 |
| 143746 | A_68_P20451906 | Ankmy1-Dusp28 | chr1:094733637-094733683 | DIVERGENT_PROMOTER | 1.133801338 | 0.0002027 |
| 10844 | A_68_P30742866 | Retnlg | chr16:048793979-048794038 | INSIDE | 1.116597884 | 0.0002067 |
| 211867 | A_68_P31959482 | Olfr1426 | chr19:012165060-012165119 | PROMOTER | 1.065427497 | 0.0002172 |
| 175733 | A_68_P31473750 | Epc1 | chr18:006514995-006515054 | INSIDE | 0.929945102 | 0.0002189 |
| 66928 | A_68_P24160814 | Baiap2l1 | chr5:144615087-144615146 | PROMOTER | 1.390818562 | 0.0002207 |
| 77937 | A_68_P22615015 | Gng5 | chr3:146442486-146442545 | INSIDE | 0.918646857 | 0.000238 |
| 5921 | A_68_P23254145 | Zfp69 | chr4:120448913-120448968 | PROMOTER | 1.076390094 | 0.0002385 |
| 179716 | A_68_P30502587 | Smug1 | chr15:103001055-103001112 | PROMOTER | 1.193325667 | 0.0002389 |
| 81452 | A_68_P23943606 | Mapk10 | chr5:103154681-103154740 | INSIDE | 1.186565716 | 0.0002447 |
| 180172 | A_68_P21079225 | Notch1 | chr2:026326836-026326885 | PROMOTER | 0.90188688 | 0.0002467 |
| 100039 | A_68_P26721535 | Zic1-Zic4 | chr9:091166753-091166812 | DIVERGENT_PROMOTER | 0.938653532 | 0.0002468 |
| 41700 | A_68_P27293201 | Glt8d2 | chr10:082118749-082118808 | INSIDE | 0.971830012 | 0.0002473 |
| 121286 | A_68_P21373977 | Dnajc10 | chr2:080111217-080111276 | PROMOTER | 0.910481471 | 0.0002487 |
| 110038 | A_68_P21068812 | Ehmt1 | chr2:024739327-024739386 | INSIDE | 0.859918071 | 0.0002503 |
| 66829 | A_68_P28058977 | Wipf2 | chr11:098675389-098675441 | PROMOTER | 0.86438839 | 0.0002563 |
| 38161 | A_68_P20409819 | Spata3 | chr1:087855178-087855233 | INSIDE | 1.061373085 | 0.0002567 |
| 31181 | A_68_P31101799 | Clcn7 | chr17:024863466-024863519 | INSIDE | 0.946151906 | 0.0002596 |
| 83744 | A_68_P26951516 | Fuca2 | chr10:013187158-013187208 | PROMOTER | 1.054602763 | 0.0002662 |
| 50456 | A_68_P23506444 | Sema3c | chr5:017088485-017088544 | INSIDE | 0.971561328 | 0.0002711 |
| 41251 | A_68_P30342876 | Gpihbp1 | chr15:075426377-075426426 | DOWNSTREAM | 0.955405057 | 0.0002721 |
| 168361 | A_68_P22308965 | Arhgef11 | chr3:087706592-087706649 | INSIDE | 1.045980448 | 0.0002746 |
| 134714 | A_68_P23621201 | Slc2a9 | chr5:038791855-038791913 | PROMOTER | 1.013919006 | 0.0002771 |
| 234421 | A_68_P23440746 | Ccnl2 | chr4:154657642-154657701 | INSIDE | 1.003979225 | 0.0002948 |
| 83800 | A_68_P22566729 | EG329763 | chr3:138009984-138010043 | PROMOTER | 0.936286335 | 0.0002977 |
| 176824 | A_68_P27843127 | Drg2 | chr11:060273008-060273061 | INSIDE | 0.961744963 | 0.0002993 |
| 4696 | A_68_P25151174 | Gabra5 | chr7:057381090-057381148 | PROMOTER | 0.91607869 | 0.0003051 |
| 20367 | A_68_P28989930 | Nol7 | chr13:043408907-043408966 | PROMOTER | 1.441278897 | 0.0003077 |
| 53750 | A_68_P21404987 | Olfr1111 | chr2:086951341-086951400 | INSIDE | 0.859999622 | 0.0003122 |
| 225981 | A_68_P20923482 | Kctd3 | chr1:190712106-190712165 | PROMOTER | 0.945697346 | 0.0003126 |
| 35859 | A_68_P32135916 | Lzts2 | chr19:045072456-045072514 | PROMOTER | 1.008413577 | 0.0003127 |
| 88194 | A_68_P22882378 | Wdr32 | chr4:045362750-045362809 | PROMOTER | 1.028463699 | 0.0003214 |
| 44445 | A_68_P26432851 | Olfr145 | chr9:037644346-037644405 | PROMOTER | 0.904770448 | 0.0003265 |
| 85959 | A_68_P23482810 | Sema3d | chr5:012387118-012387176 | PROMOTER | 1.112374738 | 0.0003311 |
| 82364 | A_68_P20604281 | Thsd7b | chr1:131097841-131097900 | PROMOTER | 1.043052439 | 0.0003428 |
| 182410 | A_68_P29237513 | Otp | chr13:095974888-095974947 | PROMOTER | 0.975308628 | 0.0003481 |
| 61793 | A_68_P25505576 | Zfp688 | chr7:127216824-127216879 | PROMOTER | 0.952929902 | 0.0003589 |
| 16024 | A_68_P31934066 | Men1 | chr19:006339069-006339128 | INSIDE | 1.104931279 | 0.0003644 |
| 81675 | A_68_P20791086 | Dpt | chr1:166634686-166634741 | INSIDE | 1.006717553 | 0.0003644 |
| 55328 | A_68_P28033919 | BC018371 | chr11:094419050-094419103 | PROMOTER | 1.043793249 | 0.0003669 |
| 103478 | A_68_P26522407 | Slc35f2 | chr9:053571113-053571172 | INSIDE | 1.091250756 | 0.0003752 |
| 56099 | A_68_P28903546 | Prlpo | chr13:027787922-027787978 | INSIDE | 1.240381723 | 0.0003832 |
| 59295 | A_68_P21874347 | Gnas | chr2:173922311-173922370 | PROMOTER | 0.933571939 | 0.0003854 |
| 109398 | A_68_P25671107 | Fbxo25 | chr8:013903233-013903288 | PROMOTER | 1.117088927 | 0.0003912 |
| 112643 | A_68_P23059440 | Cer1 | chr4:082359964-082360023 | PROMOTER | 0.911094315 | 0.0004006 |
| 157939 | A_68_P26546346 | Islr2 | chr9:057997913-057997960 | INSIDE | 1.069635775 | 0.0004047 |
| 202677 | A_68_P25406038 | Ric3 | chr7:108879878-108879937 | PROMOTER | 0.893135 | 0.0004084 |
| 193035 | A_68_P28958230 | Riok1 | chr13:038047505-038047562 | INSIDE | 1.238247064 | 0.0004091 |
| 67499 | A_68_P26977298 | D10Bwg1379e | chr10:018435364-018435423 | PROMOTER | 0.869436714 | 0.0004094 |
| 38378 | A_68_P24063252 | Ccdc92 | chr5:125149773-125149832 | INSIDE | 0.939598446 | 0.0004232 |
| 75507 | A_68_P27826615 | Galnt10 | chr11:057459685-057459744 | PROMOTER | 0.916203076 | 0.0004256 |
| 110182 | A_68_P26801786 | Ppm1m | chr9:106055532-106055591 | INSIDE | 1.053974028 | 0.000427 |
| 119213 | A_68_P32673504 | Armcx6-Armcx3 | chrX:130100235-130100293 | DIVERGENT_PROMOTER | 1.000399611 | 0.0004375 |
| 78427 | A_68_P24510962 | Ptgds2 | chr6:065073436-065073495 | INSIDE | 1.225137307 | 0.0004581 |
| 23179 | A_68_P32829923 | Rbmy1a1 | chrY:002113466-002113524 | PROMOTER | 1.135108243 | 0.0004633 |
| 15177 | A_68_P28873533 | Olfr1365 | chr13:021565288-021565347 | PROMOTER | 0.997138335 | 0.0004643 |
| 146509 | A_68_P23695575 | Lgi2 | chr5:052858765-052858817 | PROMOTER | 0.947235484 | 0.0004765 |
| 235284 | A_68_P25594384 | Ctsd | chr7:142200703-142200756 | PROMOTER | 1.16234267 | 0.0004782 |
| 86908 | A_68_P29234328 | Ap3b1 | chr13:095460515-095460574 | INSIDE | 0.905156168 | 0.0005012 |
| 183085 | A_68_P21484008 | Abtb2 | chr2:103369384-103369440 | INSIDE | 0.892251414 | 0.0005316 |

|  |  |  |  |  |  |  |
| --- | --- | --- | --- | --- | --- | --- |
| *TableS1b. Target genes at E19.5 (log(2) >0.75, p* ≤ *0.001)* | | | | | | |
| ***ProbeUID*** | ***ProbeName*** | ***GeneName*** | ***SystematicName*** | ***Description*** | ***AverageLog*** | ***Average P-value*** |
| 213748 | A_68_P20425265 | Arl4c | chr1:090532783-090532827 | INSIDE | 1.832073102 | 8.50333E-23 |
| 38686 | A_68_P23815278 | Clock | chr5:077379328-077379372 | PROMOTER | 1.592800909 | 7.19367E-22 |
| 159052 | A_68_P25793616 | Sgcz | chr8:039424285-039424344 | PROMOTER | 1.439898712 | 3.69E-21 |
| 63501 | A_68_P30207154 | Trps1 | chr15:050716940-050716999 | INSIDE | 1.346006323 | 4.90233E-21 |
| 27828 | A_68_P20449476 | Olfr1415 | chr1:094327065-094327118 | PROMOTER | 1.224418245 | 1.36833E-20 |
| 59137 | A_68_P24024978 | Fbxw | chr5:118419619-118419670 | PROMOTER | 1.157126031 | 2.80667E-20 |
| 111411 | A_68_P31111049 | Ergic1 | chr17:026290242-026290294 | INSIDE | 1.130382768 | 3.55333E-20 |
| 12168 | A_68_P25665676 | F7 | chr8:013032373-013032426 | INSIDE | 1.136189008 | 3.69667E-20 |
| 18489 | A_68_P21771109 | Epb4.1l1 | chr2:156109977-156110036 | PROMOTER | 1.214729005 | 5.56333E-20 |
| 109796 | A_68_P31663437 | Scgb3a2 | chr18:043884464-043884523 | PROMOTER | 1.142721586 | 5.78667E-20 |
| 194210 | A_68_P25422984 | Dkk3 | chr7:111950738-111950793 | PROMOTER | 1.160618523 | 6.23667E-20 |
| 132936 | A_68_P21740937 | Pygb | chr2:150475306-150475364 | PROMOTER | 1.092930424 | 6.56333E-20 |
| 104212 | A_68_P32766333 | Phex | chrX:152762381-152762440 | PROMOTER | 1.331922723 | 9.12667E-20 |
| 235602 | A_68_P25807713 | Slc7a2 | chr8:042360040-042360099 | PROMOTER | 1.173901334 | 1.08033E-19 |
| 180284 | A_68_P27006221 | Taar6 | chr10:023677791-023677850 | PROMOTER | 1.24718284 | 1.32867E-19 |
| 139160 | A_68_P29531039 | Opn4 | chr14:033434051-033434110 | PROMOTER | 1.426858936 | 1.48895E-19 |
| 201248 | A_68_P27889290 | Pik3r5 | chr11:068243310-068243366 | PROMOTER | 1.11990653 | 1.811E-19 |
| 208247 | A_68_P20442689 | Per2 | chr1:093293071-093293130 | PROMOTER | 1.400074724 | 1.91667E-19 |
| 139323 | A_68_P31953456 | Tmem109-Prpf19 | chr19:010957555-010957614 | DIVERGENT_PROMOTER | 1.182077306 | 1.97433E-19 |
| 38454 | A_68_P23360402 | Padi3 | chr4:140084564-140084621 | PROMOTER | 1.080887216 | 2.01933E-19 |
| 227991 | A_68_P27211053 | Nrbf2 | chr10:066678227-066678285 | INSIDE | 1.111575843 | 2.802E-19 |
| 118778 | A_68_P32691804 | Nrk | chrX:134259765-134259822 | PROMOTER | 1.200473405 | 2.86067E-19 |
| 117452 | A_68_P22168827 | Eif2a | chr3:058617626-058617685 | INSIDE | 1.245409145 | 2.87367E-19 |
| 217204 | A_68_P22282638 | Fga | chr3:083108840-083108899 | PROMOTER | 1.224920988 | 3.22E-19 |
| 7546 | A_68_P32143351 | Cuedc2 | chr19:046394043-046394099 | PROMOTER | 1.166513132 | 3.53867E-19 |
| 158145 | A_68_P22046066 | Dnajc19 | chr3:034271190-034271249 | INSIDE | 1.022038051 | 4.11333E-19 |
| 94241 | A_68_P21819919 | Zswim3 | chr2:164509296-164509355 | INSIDE | 1.045588787 | 4.84667E-19 |
| 146629 | A_68_P32081021 | Ifit3 | chr19:034645566-034645625 | PROMOTER | 1.185971834 | 4.915E-19 |
| 107369 | A_68_P23698162 | Slc34a2 | chr5:053336067-053336126 | PROMOTER | 1.038839308 | 5.77667E-19 |
| 28715 | A_68_P23199432 | Osbpl9 | chr4:108619250-108619306 | PROMOTER | 1.026784958 | 6.17E-19 |
| 65328 | A_68_P32374359 | Rap2c | chrX:047264882-047264932 | PROMOTER | 1.163527238 | 6.25693E-19 |
| 30951 | A_68_P31162071 | Prr3 | chr17:035591835-035591889 | PROMOTER | 1.033044754 | 7.16667E-19 |
| 198592 | A_68_P22308518 | Etv3 | chr3:087608101-087608160 | PROMOTER | 1.157175995 | 7.51E-19 |
| 219325 | A_68_P27212573 | Gm237 | chr10:066949225-066949274 | PROMOTER | 1.158892462 | 7.78667E-19 |
| 106056 | A_68_P27932285 | Abr | chr11:076286279-076286333 | INSIDE | 1.210103198 | 7.996E-19 |
| 128159 | A_68_P21244678 | Galnt5 | chr2:057813900-057813952 | INSIDE | 1.041380176 | 9.43333E-19 |
| 188702 | A_68_P28186847 | Dus1l | chr11:120616469-120616518 | PROMOTER | 1.002208622 | 1.00967E-18 |
| 60800 | A_68_P25354375 | Capn5 | chr7:098056592-098056651 | PROMOTER | 1.154481491 | 1.058E-18 |
| 40407 | A_68_P31301712 | Vapa | chr17:065518470-065518529 | PROMOTER | 1.055604258 | 1.24727E-18 |
| 182165 | A_68_P23441913 | Tnfrsf18 | chr4:154869723-154869782 | PROMOTER | 1.124024786 | 1.267E-18 |
| 37171 | A_68_P23754701 | Klhl5 | chr5:065411612-065411671 | INSIDE | 1.128409201 | 1.304E-18 |
| 41154 | A_68_P25385704 | Olfr631 | chr7:103790217-103790276 | INSIDE | 1.008736702 | 1.36667E-18 |
| 99442 | A_68_P22864037 | Arid3c | chr4:041919022-041919081 | PROMOTER | 1.056143343 | 1.383E-18 |
| 204627 | A_68_P20453458 | Agxt | chr1:094965888-094965936 | INSIDE | 1.009415478 | 1.58533E-18 |
| 158076 | A_68_P22480138 | Abca4 | chr3:122038831-122038885 | INSIDE | 1.143553041 | 1.70667E-18 |
| 15594 | A_68_P25819789 | Zfp42 | chr8:044804807-044804866 | INSIDE | 1.155505453 | 1.71667E-18 |
| 88368 | A_68_P28057388 | Ormdl3 | chr11:098408281-098408329 | PROMOTER | 0.981493832 | 1.94467E-18 |
| 226641 | A_68_P23284638 | Gja4 | chr4:126813976-126814024 | INSIDE | 0.959285338 | 1.98E-18 |
| 207971 | A_68_P26892630 | Tmem42 | chr9:122871256-122871315 | INSIDE | 0.966836663 | 2.11667E-18 |
| 204287 | A_68_P25090679 | AI480556-Rras | chr7:044885049-044885108 | DIVERGENT_PROMOTER | 0.966101756 | 2.40967E-18 |
| 238350 | A_68_P32247073 | Cybb | chrX:008624065-008624124 | INSIDE | 1.051240303 | 2.40967E-18 |
| 109355 | A_68_P28578889 | Smoc1 | chr12:081947104-081947158 | INSIDE | 1.04014737 | 2.4474E-18 |
| 22670 | A_68_P25391253 | Olfr686 | chr7:105077732-105077783 | INSIDE | 0.973561135 | 2.65567E-18 |
| 154566 | A_68_P31960199 | Olfr1434 | chr19:012352358-012352417 | DOWNSTREAM | 0.9911389 | 2.742E-18 |
| 59039 | A_68_P20441686 | Espnl | chr1:093153655-093153708 | INSIDE | 0.971573727 | 2.88667E-18 |
| 14906 | A_68_P22223819 | Gm414 | chr3:070093949-070094008 | PROMOTER | 1.096931927 | 2.89333E-18 |
| 150915 | A_68_P25464306 | Acsm2 | chr7:119350050-119350109 | PROMOTER | 1.198667696 | 3.04133E-18 |
| 217618 | A_68_P32287976 | Slc9a7 | chrX:019449446-019449503 | PROMOTER | 1.146132093 | 3.20107E-18 |
| 212824 | A_68_P25265458 | Cib1 | chr7:080102246-080102295 | INSIDE | 1.101944419 | 3.74167E-18 |
| 104302 | A_68_P25379494 | Trim21 | chr7:102438346-102438404 | INSIDE | 0.928209276 | 4.01333E-18 |
| 91072 | A_68_P21821351 | Cd40 | chr2:164744003-164744062 | PROMOTER | 0.928505991 | 4.15667E-18 |
| 224624 | A_68_P25510679 | Rgs10 | chr7:128208164-128208223 | INSIDE | 1.021165359 | 4.16233E-18 |
| 100917 | A_68_P24430349 | Svs1 | chr6:048915983-048916039 | PROMOTER | 1.085745306 | 4.246E-18 |
| 63351 | A_68_P22342935 | Mllt11-Cdc42se1 | chr3:095313847-095313906 | DIVERGENT_PROMOTER | 1.110036225 | 4.73333E-18 |
| 76594 | A_68_P32277725 | Ndph | chrX:016067164-016067223 | INSIDE | 1.119996075 | 4.83637E-18 |
| 184832 | A_68_P22393362 | Mov10 | chr3:104948563-104948622 | PROMOTER | 1.095803597 | 5.11667E-18 |
| 94238 | A_68_P26982186 | Il22ra2 | chr10:019312601-019312660 | INSIDE | 1.11393848 | 5.34967E-18 |
| 51740 | A_68_P26465733 | C1qtnf5 | chr9:043857053-043857101 | PROMOTER | 0.92990759 | 5.4E-18 |
| 97168 | A_68_P20999188 | Itih5 | chr2:010070702-010070761 | PROMOTER | 0.945139482 | 6.06667E-18 |
| 90393 | A_68_P21628947 | Idh3b-Ebf4 | chr2:129978350-129978403 | DIVERGENT_PROMOTER | 1.017117523 | 6.32333E-18 |
| 31925 | A_68_P23006675 | Megf9 | chr4:070023676-070023734 | PROMOTER | 1.354308198 | 6.43E-18 |
| 129627 | A_68_P23331523 | Lypla2 | chr4:135246178-135246236 | PROMOTER | 1.0006934 | 6.88E-18 |
| 207208 | A_68_P29050160 | Arl10 | chr13:054585813-054585871 | INSIDE | 1.015117726 | 7.14E-18 |
| 213922 | A_68_P24728919 | Lrrn1 | chr6:107493494-107493547 | PROMOTER | 1.029493274 | 7.25333E-18 |
| 66364 | A_68_P22568649 | Adh4 | chr3:138347579-138347638 | PROMOTER | 0.938380649 | 7.28E-18 |
| 144780 | A_68_P21069057 | Wdr85 | chr2:024786238-024786296 | INSIDE | 0.940665082 | 8.02667E-18 |
| 30752 | A_68_P21624110 | Ckap2l | chr2:128988288-128988339 | INSIDE | 0.897503193 | 8.39667E-18 |
| 88710 | A_68_P28048244 | Scrn2 | chr11:096844264-096844320 | PROMOTER | 1.020417 | 8.893E-18 |
| 218575 | A_68_P25188315 | Aldh1a3 | chr7:066311039-066311098 | PROMOTER | 0.937990879 | 8.89667E-18 |
| 196701 | A_68_P20261948 | Als2cr11 | chr1:059043573-059043632 | PROMOTER | 0.97967109 | 9.01167E-18 |
| 3124 | A_68_P21409398 | Olfr1176 | chr2:088142536-088142595 | DOWNSTREAM | 1.05551026 | 9.077E-18 |
| 52672 | A_68_P25386377 | Olfr642 | chr7:103923647-103923694 | INSIDE | 0.97217705 | 9.50333E-18 |
| 86210 | A_68_P22332484 | Sprrl3 | chr3:092740260-092740319 | PROMOTER | 1.086831847 | 9.84667E-18 |
| 124781 | A_68_P28122168 | Abca8a | chr11:109908837-109908892 | PROMOTER | 1.10951997 | 1.00533E-17 |
| 56096 | A_68_P23379947 | Dhrs3 | chr4:144156243-144156297 | PROMOTER | 0.899403387 | 1.043E-17 |
| 100604 | A_68_P30494855 | Krt4 | chr15:101750598-101750657 | INSIDE | 1.065137827 | 1.17957E-17 |
| 114946 | A_68_P21562098 | Thbs1 | chr2:117804943-117805000 | INSIDE | 1.023917187 | 1.384E-17 |
| 111087 | A_68_P31093921 | Tceb2 | chr17:023559922-023559970 | PROMOTER | 0.95222102 | 1.43713E-17 |
| 21125 | A_68_P28917620 | Uqcrfs1 | chr13:030554100-030554151 | PROMOTER | 0.973952422 | 1.4718E-17 |
| 198670 | A_68_P30514006 | Ppl | chr16:005049351-005049404 | PROMOTER | 1.064427508 | 1.48833E-17 |
| 10194 | A_68_P31931659 | Cdc42ep2 | chr19:005917838-005917897 | INSIDE | 1.238851925 | 1.53017E-17 |
| 80556 | A_68_P24152575 | Wipi2 | chr5:142876700-142876750 | PROMOTER | 0.958050269 | 1.53633E-17 |
| 227444 | A_68_P20441489 | Scly | chr1:093123510-093123569 | PROMOTER | 0.904309814 | 1.53653E-17 |
| 11050 | A_68_P32017864 | Mamdc2 | chr19:023513892-023513951 | INSIDE | 1.17368518 | 1.62003E-17 |
| 207443 | A_68_P25415589 | Rnf141 | chr7:110634699-110634757 | INSIDE | 0.872869856 | 1.69267E-17 |
| 106843 | A_68_P21776615 | Manbal | chr2:157057161-157057220 | PROMOTER | 1.188275979 | 1.77007E-17 |
| 213105 | A_68_P26512793 | Rdx | chr9:051795473-051795532 | PROMOTER | 1.294341441 | 1.83699E-17 |
| 21519 | A_68_P23141892 | Angptl3 | chr4:098519447-098519506 | PROMOTER | 0.873518862 | 1.929E-17 |
| 79655 | A_68_P31354408 | Ltbp1 | chr17:074911534-074911580 | INSIDE | 0.943176413 | 2.03267E-17 |
| 238937 | A_68_P23392299 | Agtrap | chr4:146929477-146929533 | INSIDE | 0.95939602 | 2.03573E-17 |
| 222429 | A_68_P24389480 | Olfr460 | chr6:040499705-040499764 | PROMOTER | 1.182188656 | 2.05357E-17 |
| 131452 | A_68_P25993820 | Mmaa | chr8:082188838-082188897 | INSIDE | 1.036015742 | 2.05833E-17 |
| 23600 | A_68_P21320143 | Dlx2 | chr2:071347878-071347937 | PROMOTER | 1.017357425 | 2.11567E-17 |
| 153593 | A_68_P23566399 | Rnf32 | chr5:029522542-029522597 | PROMOTER | 0.92776839 | 2.3E-17 |
| 238783 | A_68_P22061887 | Il2 | chr3:037320758-037320804 | PROMOTER | 0.856854993 | 2.74333E-17 |
| 189512 | A_68_P25583445 | Olfr60 | chr7:140199063-140199122 | PROMOTER | 1.083300386 | 2.91033E-17 |
| 96054 | A_68_P26991416 | Myb | chr10:020854064-020854118 | PROMOTER | 0.911184919 | 3.04667E-17 |
| 202103 | A_68_P21752011 | Fkhl18 | chr2:152627164-152627208 | PROMOTER | 0.952644098 | 3.12067E-17 |
| 120708 | A_68_P22409816 | Gstm3 | chr3:108096914-108096969 | INSIDE | 0.92607796 | 3.37667E-17 |
| 81564 | A_68_P27807863 | Slc22a4 | chr11:053874064-053874118 | PROMOTER | 0.902866745 | 3.3969E-17 |
| 88974 | A_68_P25005895 | Lypd3 | chr7:024340600-024340659 | PROMOTER | 0.896288073 | 3.40333E-17 |
| 101462 | A_68_P21066261 | Pax8 | chr2:024302791-024302848 | PROMOTER | 0.892500913 | 3.475E-17 |
| 134019 | A_68_P24946383 | Tfpt | chr7:003224064-003224113 | INSIDE | 0.861719162 | 3.56333E-17 |
| 86795 | A_68_P30325002 | Kcnk9 | chr15:072371775-072371829 | INSIDE | 0.890007107 | 3.57867E-17 |
| 14233 | A_68_P32203560 | Vwa2 | chr19:056923448-056923501 | PROMOTER | 0.903386544 | 3.632E-17 |
| 194024 | A_68_P24991558 | Fbxo46 | chr7:018276694-018276742 | PROMOTER | 0.877210388 | 3.71E-17 |
| 156924 | A_68_P32151503 | Col17a1 | chr19:047749339-047749397 | PROMOTER | 0.892158761 | 3.81133E-17 |
| 107284 | A_68_P25350660 | Ints4 | chr7:097353013-097353067 | PROMOTER | 0.858481782 | 4.11867E-17 |
| 79289 | A_68_P24454696 | Jazf1 | chr6:052999724-052999777 | PROMOTER | 0.944701837 | 4.46E-17 |
| 179556 | A_68_P31610636 | Epb4.1l4a | chr18:034136628-034136687 | PROMOTER | 1.001069561 | 4.60267E-17 |
| 177986 | A_68_P25957226 | Plvap | chr8:074440596-074440643 | INSIDE | 0.881912389 | 4.80333E-17 |
| 208458 | A_68_P22410470 | Ampd2-Gnat2 | chr3:108220132-108220184 | DIVERGENT_PROMOTER | 0.836353516 | 4.85E-17 |
| 66390 | A_68_P27292908 | Tdg | chr10:082057871-082057925 | PROMOTER | 0.939118612 | 5.044E-17 |
| 168361 | A_68_P22308965 | Arhgef11 | chr3:087706592-087706649 | INSIDE | 0.891409133 | 5.08333E-17 |
| 203914 | A_68_P20971496 | Mcm10 | chr2:004933070-004933127 | PROMOTER | 0.884036311 | 5.09333E-17 |
| 106760 | A_68_P22442162 | Olfm3 | chr3:115074388-115074447 | INSIDE | 1.171683834 | 5.34333E-17 |
| 53614 | A_68_P21256615 | Baz2b | chr2:059926672-059926731 | PROMOTER | 1.102171809 | 5.35E-17 |
| 140841 | A_68_P32457081 | Magea4 | chrX:068470109-068470165 | PROMOTER | 1.107655581 | 5.39887E-17 |
| 189498 | A_68_P21741364 | Abhd12 | chr2:150534724-150534773 | PROMOTER | 0.939163183 | 5.408E-17 |
| 24888 | A_68_P31994415 | Rorb | chr19:019072160-019072219 | PROMOTER | 0.988119254 | 5.424E-17 |
| 71881 | A_68_P23360916 | Padi2 | chr4:140175428-140175477 | PROMOTER | 0.846599564 | 5.48333E-17 |
| 39956 | A_68_P31099190 | Rnf151-Rps2 | chr17:024446229-024446284 | DIVERGENT_PROMOTER | 0.96444275 | 5.51979E-17 |
| 36282 | A_68_P20728383 | Rgs16 | chr1:155503583-155503639 | INSIDE | 0.991445162 | 5.61067E-17 |
| 215121 | A_68_P30152107 | Slc25a32 | chr15:038940674-038940733 | INSIDE | 0.972455714 | 5.88213E-17 |
| 161907 | A_68_P28883335 | Hist1h4b | chr13:023764410-023764468 | PROMOTER | 0.929027598 | 5.88767E-17 |
| 215831 | A_68_P21137863 | Lhx6 | chr2:035930593-035930652 | PROMOTER | 0.901137953 | 6.01333E-17 |
| 168913 | A_68_P21141621 | Pdcl | chr2:037179915-037179969 | INSIDE | 0.917996736 | 6.033E-17 |
| 207025 | A_68_P25598355 | R74862 | chr7:142865796-142865844 | PROMOTER | 0.846741481 | 6.06333E-17 |
| 88051 | A_68_P24776827 | Alox5 | chr6:116424449-116424508 | INSIDE | 1.107166288 | 6.20767E-17 |
| 204261 | A_68_P20351390 | Ccdc108 | chr1:074868212-074868271 | INSIDE | 1.010379973 | 6.34E-17 |
| 3758 | A_68_P24803641 | Slc6a12 | chr6:121310817-121310873 | PROMOTER | 0.923655542 | 6.86333E-17 |
| 193281 | A_68_P30577447 | Prodh | chr16:018002143-018002195 | INSIDE | 1.005262715 | 6.96667E-17 |
| 152753 | A_68_P24921065 | Lrmp | chr6:145073579-145073638 | PROMOTER | 1.013552816 | 7.01E-17 |
| 177100 | A_68_P32464057 | Slc6a8 | chrX:069925429-069925480 | PROMOTER | 1.000134882 | 7.279E-17 |
| 51596 | A_68_P27290286 | Gna11 | chr10:080932091-080932150 | INSIDE | 0.858867917 | 7.53933E-17 |
| 230840 | A_68_P22333817 | Sprrl1 | chr3:093030154-093030203 | INSIDE | 0.885422405 | 7.687E-17 |
| 47610 | A_68_P23694239 | Sod3 | chr5:052650248-052650307 | PROMOTER | 0.871851943 | 8.03333E-17 |
| 100633 | A_68_P32412898 | Mcf2 | chrX:056493385-056493444 | INSIDE | 1.060395147 | 8.08983E-17 |
| 91927 | A_68_P20827004 | B4galt3 | chr1:173106749-173106804 | PROMOTER | 0.981161674 | 8.18833E-17 |
| 30590 | A_68_P23331243 | Fuca1 | chr4:135194861-135194917 | INSIDE | 0.828286752 | 9.21E-17 |
| 32709 | A_68_P25034938 | Fxyd3 | chr7:030789549-030789594 | PROMOTER | 1.104269536 | 9.33033E-17 |
| 193848 | A_68_P26435346 | Olfr914 | chr9:038352076-038352135 | PROMOTER | 1.207842036 | 9.43533E-17 |
| 46817 | A_68_P23198170 | Rab3b | chr4:108377819-108377867 | INSIDE | 0.839225367 | 9.57433E-17 |
| 159008 | A_68_P31022599 | Vil2 | chr17:006634936-006634995 | PROMOTER | 0.999829799 | 9.7712E-17 |
| 4960 | A_68_P28698627 | Serpina10 | chr12:104029260-104029319 | INSIDE | 1.007800014 | 1.00817E-16 |
| 58131 | A_68_P27228585 | Slc16a9 | chr10:069635877-069635934 | PROMOTER | 1.105883821 | 1.02736E-16 |
| 141894 | A_68_P29754583 | Olfm4 | chr14:078732591-078732650 | PROMOTER | 0.911303242 | 1.06233E-16 |
| 182425 | A_68_P29589391 | Otx2 | chr14:047587881-047587932 | PROMOTER | 0.977720084 | 1.06353E-16 |
| 220801 | A_68_P21732393 | Cst13 | chr2:148514001-148514051 | INSIDE | 0.867535073 | 1.08803E-16 |
| 206782 | A_68_P21070746 | Gm757 | chr2:025058240-025058289 | INSIDE | 0.803512129 | 1.15867E-16 |
| 210407 | A_68_P23440451 | Atad3a | chr4:154602929-154602988 | INSIDE | 0.86349903 | 1.18033E-16 |
| 188725 | A_68_P22187590 | Dhx36 | chr3:062593368-062593427 | INSIDE | 1.068870715 | 1.20813E-16 |
| 111636 | A_68_P31601534 | Map3k2 | chr18:032307913-032307972 | INSIDE | 1.028855699 | 1.24243E-16 |
| 222170 | A_68_P24221499 | Tac1 | chr6:007507024-007507080 | INSIDE | 0.95526031 | 1.28E-16 |
| 99929 | A_68_P27884872 | Rcvrn | chr11:067511641-067511686 | INSIDE | 0.895125533 | 1.28193E-16 |
| 4271 | A_68_P24877012 | Ptpro | chr6:137214831-137214890 | PROMOTER | 0.98934565 | 1.30267E-16 |
| 40329 | A_68_P24993170 | Ckm | chr7:018570294-018570350 | INSIDE | 0.886846438 | 1.30887E-16 |
| 96945 | A_68_P27184980 | Tacr2 | chr10:061647901-061647946 | PROMOTER | 0.7951191 | 1.30933E-16 |
| 130471 | A_68_P24839029 | Fkbp4 | chr6:128404967-128405026 | PROMOTER | 0.832850442 | 1.31633E-16 |
| 7221 | A_68_P20321255 | Ikzf2 | chr1:069622760-069622819 | PROMOTER | 0.942591119 | 1.31967E-16 |
| 3950 | A_68_P24595342 | Ankrd53 | chr6:083728287-083728346 | INSIDE | 0.81839917 | 1.344E-16 |
| 92253 | A_68_P24426874 | Krba1 | chr6:048326072-048326125 | INSIDE | 0.887982409 | 1.3621E-16 |
| 80648 | A_68_P21129968 | Fbxw2 | chr2:034648809-034648861 | PROMOTER | 0.880888006 | 1.378E-16 |
| 69108 | A_68_P26700015 | Rwdd2 | chr9:086368963-086369008 | INSIDE | 0.88214942 | 1.40163E-16 |
| 218580 | A_68_P25665713 | F10 | chr8:013036432-013036482 | PROMOTER | 0.826832631 | 1.40303E-16 |
| 195093 | A_68_P30947628 | Krtap13-1 | chr16:088619125-088619184 | DOWNSTREAM | 1.015491044 | 1.40453E-16 |
| 192362 | A_68_P20786744 | Sele | chr1:165886158-165886217 | INSIDE | 0.86729571 | 1.481E-16 |
| 70053 | A_68_P26079823 | Coq9 | chr8:097741709-097741768 | INSIDE | 0.950403331 | 1.48267E-16 |
| 237248 | A_68_P21073567 | Lcn8 | chr2:025472056-025472115 | PROMOTER | 1.154267475 | 1.51277E-16 |
| 95884 | A_68_P28530888 | Rtn1 | chr12:073157170-073157221 | PROMOTER | 0.888134733 | 1.53007E-16 |
| 75527 | A_68_P22218914 | Trim59 | chr3:069133711-069133770 | PROMOTER | 1.043085084 | 1.536E-16 |
| 203059 | A_68_P32415813 | Sox3 | chrX:057154629-057154673 | PROMOTER | 0.912942027 | 1.53673E-16 |
| 107979 | A_68_P20729136 | EG433365 | chr1:155637808-155637859 | INSIDE | 0.819428434 | 1.629E-16 |
| 225289 | A_68_P26127966 | Cmtm3 | chr8:107224773-107224831 | PROMOTER | 0.903627538 | 1.65823E-16 |
| 232005 | A_68_P28540846 | Snapc1 | chr12:074883841-074883897 | INSIDE | 0.888428645 | 1.709E-16 |
| 34549 | A_68_P27255978 | Adora2a | chr10:074767558-074767613 | PROMOTER | 0.989382827 | 1.74527E-16 |
| 143328 | A_68_P26467664 | Upk2 | chr9:044204922-044204973 | INSIDE | 0.863412817 | 1.75663E-16 |
| 113257 | A_68_P30572538 | Ube2l3-Gm603 | chr16:017121893-017121942 | DIVERGENT_PROMOTER | 0.943312753 | 1.775E-16 |
| 160531 | A_68_P20454230 | Sned1 | chr1:095065485-095065544 | PROMOTER | 0.808229403 | 1.795E-16 |
| 145767 | A_68_P21932517 | Stmn2 | chr3:008489920-008489979 | PROMOTER | 1.004489568 | 1.82533E-16 |
| 184847 | A_68_P29648483 | Sacs | chr14:060091798-060091846 | PROMOTER | 0.814228658 | 1.92767E-16 |
| 124865 | A_68_P25100866 | Saa3 | chr7:046583793-046583852 | PROMOTER | 0.826703467 | 1.943E-16 |
| 153924 | A_68_P30358263 | Rabl4 | chr15:078002089-078002148 | PROMOTER | 0.865931166 | 1.94317E-16 |
| 237114 | A_68_P32217670 | Vax1 | chr19:059221088-059221146 | INSIDE | 0.879694758 | 1.96767E-16 |
| 131465 | A_68_P26023024 | Junb-Hook2 | chr8:087876666-087876723 | DIVERGENT_PROMOTER | 0.791668486 | 2.04667E-16 |
| 58183 | A_68_P22246562 | Pdcd10 | chr3:075637957-075638016 | INSIDE | 0.952918923 | 2.067E-16 |
| 43674 | A_68_P31845677 | Katnal2 | chr18:077253609-077253660 | PROMOTER | 0.942823124 | 2.128E-16 |
| 70823 | A_68_P24122120 | Cutl1 | chr5:136855143-136855200 | PROMOTER | 0.816859941 | 2.14947E-16 |
| 240030 | A_68_P22535302 | Dkk2 | chr3:132018882-132018930 | PROMOTER | 0.833867489 | 2.19843E-16 |
| 21121 | A_68_P21220343 | Cacnb4 | chr2:052381938-052381996 | PROMOTER | 0.83157364 | 2.23367E-16 |
| 93919 | A_68_P29086223 | Dapk1 | chr13:060606272-060606324 | PROMOTER | 0.816335353 | 2.31633E-16 |
| 196578 | A_68_P24954805 | Zfp667 | chr7:005890462-005890521 | INSIDE | 0.840718631 | 2.71E-16 |
| 14022 | A_68_P24631241 | V1ra8 | chr6:090166843-090166902 | PROMOTER | 0.937843141 | 2.879E-16 |
| 125861 | A_68_P29716879 | Rb1 | chr14:072057759-072057818 | INSIDE | 1.064338602 | 3.01203E-16 |
| 177930 | A_68_P32588990 | Tbx22 | chrX:103877852-103877911 | PROMOTER | 0.922477612 | 3.0728E-16 |
| 150860 | A_68_P24817413 | Gpr162 | chr6:124825936-124825985 | INSIDE | 0.79544532 | 3.13967E-16 |
| 210577 | A_68_P30343460 | Rhpn1 | chr15:075530416-075530468 | PROMOTER | 0.777321474 | 3.52667E-16 |
| 141174 | A_68_P26659226 | Dppa5 | chr9:078154495-078154554 | PROMOTER | 0.927692286 | 3.582E-16 |
| 122800 | A_68_P28902752 | Prlpc4 | chr13:027593638-027593697 | PROMOTER | 1.213746884 | 3.68519E-16 |
| 22172 | A_68_P32127548 | Nkx2-3 | chr19:043662918-043662977 | PROMOTER | 0.943944122 | 3.72214E-16 |
| 21486 | A_68_P27005976 | Taar3 | chr10:023638948-023639007 | INSIDE | 0.840995771 | 3.73933E-16 |
| 64657 | A_68_P27279508 | Ela2 | chr10:079291302-079291359 | INSIDE | 0.8224791 | 3.869E-16 |
| 221606 | A_68_P30493026 | EG406223 | chr15:101455943-101455994 | INSIDE | 0.803619026 | 3.95633E-16 |
| 9752 | A_68_P31920581 | Doc2g | chr19:004004218-004004277 | INSIDE | 0.822087987 | 4.01633E-16 |
| 9227 | A_68_P23337028 | Epha8 | chr4:136230944-136230993 | PROMOTER | 0.812278543 | 4.171E-16 |
| 108030 | A_68_P24486169 | Gprin3 | chr6:059356170-059356220 | PROMOTER | 0.869690166 | 4.24267E-16 |
| 214045 | A_68_P25378007 | Stim1 | chr7:102143344-102143403 | INSIDE | 0.858432534 | 4.46E-16 |
| 113749 | A_68_P28452157 | Nfkbia | chr12:056412050-056412103 | PROMOTER | 0.846453712 | 4.46993E-16 |
| 161922 | A_68_P24156987 | Rac1 | chr5:143788678-143788735 | PROMOTER | 0.883718979 | 4.66633E-16 |
| 29923 | A_68_P32262602 | Atp6ap2 | chrX:011745605-011745664 | INSIDE | 1.200710541 | 4.7699E-16 |
| 10766 | A_68_P24170117 | Usp12 | chr5:147103639-147103689 | INSIDE | 0.792858968 | 4.826E-16 |
| 216794 | A_68_P28730306 | AI132487 | chr12:109297858-109297909 | PROMOTER | 0.861527729 | 4.9472E-16 |
| 224569 | A_68_P28530013 | Gpr135 | chr12:072990841-072990894 | PROMOTER | 0.792866281 | 4.979E-16 |
| 152591 | A_68_P24397346 | Clcn1 | chr6:042215633-042215687 | PROMOTER | 0.895155764 | 5.257E-16 |
| 205732 | A_68_P30013439 | C1qtnf3 | chr15:010896855-010896914 | PROMOTER | 0.931569129 | 5.27457E-16 |
| 97307 | A_68_P21388669 | Zc3h15 | chr2:083441587-083441646 | PROMOTER | 0.862943597 | 5.44E-16 |
| 16124 | A_68_P20644023 | Kif21b | chr1:137946485-137946543 | PROMOTER | 0.79233545 | 5.54E-16 |
| 189377 | A_68_P20736171 | Stx6 | chr1:156920575-156920634 | PROMOTER | 0.865326632 | 5.64333E-16 |
| 74443 | A_68_P26169631 | Ctrb1 | chr8:114577875-114577922 | PROMOTER | 0.782275519 | 5.69E-16 |
| 136463 | A_68_P31171367 | Olfr135 | chr17:037812233-037812288 | PROMOTER | 0.846509563 | 5.75333E-16 |
| 156580 | A_68_P25294419 | Olfr305 | chr7:086243273-086243332 | PROMOTER | 0.957408903 | 6.116E-16 |
| 38516 | A_68_P28186426 | Dcxr | chr11:120542159-120542207 | INSIDE | 0.948334375 | 6.25667E-16 |
| 51773 | A_68_P26288076 | Mmp1a | chr9:007464082-007464141 | PROMOTER | 1.303226275 | 6.26E-16 |
| 153551 | A_68_P26460552 | Oaf | chr9:042995420-042995470 | PROMOTER | 0.811493463 | 6.38767E-16 |
| 74410 | A_68_P24173619 | Pan3 | chr5:147739338-147739397 | PROMOTER | 0.797744641 | 6.39403E-16 |
| 238200 | A_68_P32588003 | Itm2a | chrX:103607017-103607075 | PROMOTER | 0.993814935 | 6.45227E-16 |
| 236392 | A_68_P21912040 | Tcea2 | chr2:181610537-181610592 | INSIDE | 0.795074418 | 6.61667E-16 |
| 225820 | A_68_P27056249 | Tspyl4 | chr10:033989591-033989644 | INSIDE | 0.91079153 | 6.75943E-16 |
| 163660 | A_68_P24811801 | Clec4n | chr6:123192805-123192864 | PROMOTER | 0.829943505 | 6.83E-16 |
| 3795 | A_68_P29060094 | Neurog1 | chr13:056259225-056259281 | DOWNSTREAM | 0.934637552 | 7.241E-16 |
| 164364 | A_68_P21757435 | Spag4l | chr2:153562180-153562239 | INSIDE | 0.811118316 | 7.29833E-16 |
| 136503 | A_68_P23756862 | Hip2 | chr5:065817259-065817318 | INSIDE | 0.902006169 | 7.94333E-16 |
| 114062 | A_68_P28040172 | Phb | chr11:095484861-095484917 | INSIDE | 0.98469541 | 8.38118E-16 |
| 40606 | A_68_P30570747 | Top3b | chr16:016784772-016784825 | INSIDE | 1.08535845 | 8.68983E-16 |
| 14867 | A_68_P20391701 | Irs1 | chr1:082168211-082168261 | INSIDE | 0.885191927 | 8.88333E-16 |
| 227381 | A_68_P24613272 | Aak1 | chr6:086960772-086960829 | DOWNSTREAM | 0.840836625 | 9.15E-16 |
| 94091 | A_68_P21079845 | Egfl7 | chr2:026414653-026414707 | INSIDE | 0.862083142 | 9.9272E-16 |
| 227281 | A_68_P22332958 | Sprrl9 | chr3:092855187-092855243 | PROMOTER | 0.807781866 | 1.02633E-15 |
| 216321 | A_68_P23390929 | MGC67181 | chr4:146690363-146690421 | INSIDE | 0.939875239 | 1.04767E-15 |
| 72385 | A_68_P24983333 | Strn4 | chr7:015969159-015969203 | PROMOTER | 0.790047354 | 1.0619E-15 |
| 195829 | A_68_P26783246 | Slco2a1 | chr9:102862128-102862178 | PROMOTER | 0.808355068 | 1.078E-15 |
| 18575 | A_68_P21833112 | Ddx27 | chr2:166702400-166702459 | PROMOTER | 0.824935075 | 1.1246E-15 |
| 155299 | A_68_P23891985 | Cdkl2 | chr5:093120290-093120349 | PROMOTER | 1.044429612 | 1.1551E-15 |
| 119301 | A_68_P24975614 | Cabp5 | chr7:012296831-012296890 | PROMOTER | 0.918697193 | 1.16083E-15 |
| 180842 | A_68_P21766485 | Gss | chr2:155288622-155288670 | PROMOTER | 0.848515915 | 1.16493E-15 |
| 104080 | A_68_P27914540 | P2rx1 | chr11:072815767-072815817 | INSIDE | 0.829823394 | 1.20773E-15 |
| 159621 | A_68_P27560525 | Nefh | chr11:004850374-004850427 | PROMOTER | 0.897622224 | 1.28683E-15 |
| 137405 | A_68_P26820786 | Cdc25a | chr9:109733619-109733663 | INSIDE | 0.8173937 | 1.28763E-15 |
| 92039 | A_68_P24947745 | Ttyh1 | chr7:003722080-003722139 | PROMOTER | 0.773961974 | 1.28967E-15 |
| 144624 | A_68_P25004993 | Plaur | chr7:024168987-024169036 | PROMOTER | 0.884509641 | 1.31707E-15 |
| 99444 | A_68_P21822960 | Elmo2 | chr2:164984438-164984496 | INSIDE | 0.816711651 | 1.31933E-15 |
| 72054 | A_68_P20147172 | Ugcgl1 | chr1:036190511-036190570 | PROMOTER | 0.780458907 | 1.3759E-15 |
| 38565 | A_68_P27445230 | E2f7 | chr10:110151143-110151197 | INSIDE | 0.978698272 | 1.40563E-15 |
| 181528 | A_68_P24973964 | Zfp446 | chr7:011879378-011879433 | INSIDE | 0.750774847 | 1.42133E-15 |
| 195094 | A_68_P25937661 | Nat3 | chr8:070452361-070452417 | PROMOTER | 0.797170829 | 1.47183E-15 |
| 112255 | A_68_P23991434 | Tfip11 | chr5:112564456-112564512 | PROMOTER | 0.83543969 | 1.502E-15 |
| 141936 | A_68_P27902125 | Camta2 | chr11:070502489-070502542 | INSIDE | 0.774025823 | 1.50287E-15 |
| 137761 | A_68_P32611512 | EG237009 | chrX:111017754-111017813 | PROMOTER | 0.912085714 | 1.51863E-15 |
| 6117 | A_68_P21902647 | Cables2 | chr2:180204532-180204584 | PROMOTER | 0.78933986 | 1.57247E-15 |
| 195365 | A_68_P24979802 | Kptn | chr7:015285160-015285219 | INSIDE | 0.799161233 | 1.58477E-15 |
| 64579 | A_68_P32412253 | F9 | chrX:056347750-056347809 | INSIDE | 0.98106834 | 1.59789E-15 |
| 25728 | A_68_P32141759 | Ldb1 | chr19:046092086-046092137 | INSIDE | 0.965467248 | 1.61217E-15 |
| 144860 | A_68_P27836025 | Wnt3a | chr11:059106180-059106224 | INSIDE | 0.775711386 | 1.63467E-15 |
| 37489 | A_68_P30340444 | Ly6e | chr15:074780321-074780380 | PROMOTER | 0.840512157 | 1.68007E-15 |
| 79740 | A_68_P32468043 | G6pdx | chrX:070681144-070681203 | INSIDE | 0.835044298 | 1.701E-15 |
| 117950 | A_68_P24468762 | Aqp1 | chr6:055264629-055264685 | PROMOTER | 0.801557485 | 1.712E-15 |
| 201780 | A_68_P21089670 | Olfm1 | chr2:028024992-028025051 | PROMOTER | 0.756865852 | 1.718E-15 |
| 134615 | A_68_P27836149 | Wnt9a | chr11:059124918-059124966 | INSIDE | 0.822786098 | 1.7362E-15 |
| 39116 | A_68_P31838835 | Gm672 | chr18:075823567-075823626 | PROMOTER | 0.887328505 | 1.76185E-15 |
| 77303 | A_68_P27927067 | Pitpna | chr11:075414028-075414087 | INSIDE | 0.877547678 | 1.76629E-15 |
| 87392 | A_68_P24359949 | Cnot4 | chr6:035062994-035063048 | INSIDE | 0.751483636 | 1.78833E-15 |
| 79115 | A_68_P24051495 | P2rx7 | chr5:122903083-122903142 | PROMOTER | 0.88471636 | 1.81357E-15 |
| 24033 | A_68_P24062135 | Gtf2h3 | chr5:124855548-124855598 | INSIDE | 0.91840462 | 1.839E-15 |
| 188230 | A_68_P22727607 | Plekhf2 | chr4:010940081-010940139 | PROMOTER | 0.791505647 | 1.84E-15 |
| 166996 | A_68_P31207957 | Ptk7 | chr17:046090455-046090514 | INSIDE | 0.920361394 | 1.84313E-15 |
| 182435 | A_68_P24785604 | Ret | chr6:118167145-118167190 | PROMOTER | 0.747829301 | 1.87433E-15 |
| 15440 | A_68_P25951448 | Comp | chr8:073304889-073304943 | INSIDE | 0.863016623 | 1.88E-15 |
| 191207 | A_68_P25576313 | Stk32c | chr7:139040401-139040460 | PROMOTER | 1.060671298 | 1.93333E-15 |
| 134931 | A_68_P30931886 | Adamts5 | chr16:085788223-085788282 | INSIDE | 0.845373664 | 1.9768E-15 |
| 5768 | A_68_P23532189 | Orc5l | chr5:022061552-022061611 | INSIDE | 0.844418525 | 1.98E-15 |
| 192720 | A_68_P25034023 | Mag | chr7:030621200-030621259 | INSIDE | 0.828386748 | 2.013E-15 |
| 234098 | A_68_P25380512 | Olfr560 | chr7:102630809-102630868 | PROMOTER | 0.798314702 | 2.02567E-15 |
| 66118 | A_68_P23163707 | Tctex1d1 | chr4:102483514-102483573 | PROMOTER | 0.825356226 | 2.05967E-15 |
| 39080 | A_68_P24362078 | Mtpn | chr6:035473232-035473291 | PROMOTER | 0.830507537 | 2.12333E-15 |
| 36423 | A_68_P30386185 | Nfam1 | chr15:082852063-082852114 | PROMOTER | 0.925512625 | 2.20691E-15 |
| 54076 | A_68_P25051008 | Dpy19l3 | chr7:035463483-035463537 | PROMOTER | 0.802869062 | 2.212E-15 |
| 127535 | A_68_P31147469 | BC066107 | chr17:032630038-032630097 | PROMOTER | 0.976812399 | 2.28167E-15 |
| 51944 | A_68_P28280398 | Adam17 | chr12:021625066-021625125 | PROMOTER | 0.948636348 | 2.28503E-15 |
| 224378 | A_68_P26814171 | Tcta | chr9:108161326-108161379 | INSIDE | 0.777480159 | 2.29167E-15 |
| 178149 | A_68_P24469046 | Ghrhr | chr6:055307317-055307373 | INSIDE | 0.767743224 | 2.309E-15 |
| 128204 | A_68_P23318811 | Pigv | chr4:132946904-132946963 | PROMOTER | 0.94288157 | 2.33067E-15 |
| 208396 | A_68_P32453967 | Cd99l2 | chrX:067753591-067753637 | PROMOTER | 0.7887737 | 2.34731E-15 |
| 82564 | A_68_P20874646 | Psen2 | chr1:182086630-182086685 | PROMOTER | 0.772532355 | 2.37233E-15 |
| 26233 | A_68_P25945217 | Lpl | chr8:071811019-071811078 | INSIDE | 1.027491618 | 2.41333E-15 |
| 214709 | A_68_P28856833 | Pou6f2 | chr13:018393182-018393226 | PROMOTER | 0.793503095 | 2.428E-15 |
| 59970 | A_68_P24905350 | Kcnj8 | chr6:142529189-142529248 | PROMOTER | 0.879024255 | 2.4445E-15 |
| 178521 | A_68_P31952512 | Cd5 | chr19:010805597-010805648 | INSIDE | 0.844862479 | 2.45833E-15 |
| 103526 | A_68_P28954024 | Ly86 | chr13:037351547-037351606 | PROMOTER | 0.805344438 | 2.477E-15 |
| 66129 | A_68_P32242251 | Porcn | chrX:007362441-007362500 | INSIDE | 0.995170169 | 2.48855E-15 |
| 121993 | A_68_P28699046 | Serpina1f | chr12:104094286-104094345 | INSIDE | 1.098543487 | 2.50863E-15 |
| 47431 | A_68_P22325706 | S100a13 | chr3:090609358-090609414 | INSIDE | 0.943274335 | 2.57673E-15 |
| 32519 | A_68_P31626458 | Pcdha7 | chr18:037099858-037099917 | PROMOTER | 1.025105987 | 2.59333E-15 |
| 131868 | A_68_P27789373 | Adamts2 | chr11:050445278-050445322 | PROMOTER | 0.810249474 | 2.75434E-15 |
| 193412 | A_68_P31095244 | Pdpk1 | chr17:023805428-023805481 | INSIDE | 0.835197143 | 2.7891E-15 |
| 167837 | A_68_P30422917 | Trabd | chr15:088901122-088901171 | PROMOTER | 0.791158612 | 2.92733E-15 |
| 138392 | A_68_P31932888 | Snx15 | chr19:006132824-006132878 | PROMOTER | 0.925260231 | 2.9639E-15 |
| 75167 | A_68_P23950911 | Dmp1 | chr5:104441539-104441595 | PROMOTER | 0.778066007 | 2.98933E-15 |
| 228562 | A_68_P25032456 | Zbtb32 | chr7:030304977-030305036 | PROMOTER | 0.922218217 | 3.09867E-15 |
| 210576 | A_68_P28035542 | Col1a1 | chr11:094749386-094749435 | PROMOTER | 0.76572354 | 3.10733E-15 |
| 193426 | A_68_P27926069 | Serpinf2 | chr11:075253611-075253661 | INSIDE | 0.765233382 | 3.20033E-15 |
| 119586 | A_68_P20900469 | Iars2 | chr1:187014371-187014419 | INSIDE | 0.752678916 | 3.23E-15 |
| 53898 | A_68_P27007446 | Moxd1 | chr10:023913840-023913895 | INSIDE | 0.939141418 | 3.27977E-15 |
| 88232 | A_68_P22342342 | Tmod4 | chr3:095210959-095211014 | INSIDE | 0.795429765 | 3.28667E-15 |
| 202563 | A_68_P21820806 | Slc12a5 | chr2:164660889-164660943 | INSIDE | 0.752909843 | 3.34633E-15 |
| 62093 | A_68_P25804783 | Fgf20 | chr8:041787488-041787547 | PROMOTER | 0.792091748 | 3.42667E-15 |
| 228516 | A_68_P24449011 | Hoxa3 | chr6:052130515-052130571 | PROMOTER | 0.849221425 | 3.54E-15 |
| 185955 | A_68_P23874883 | Slc4a4 | chr5:090011033-090011092 | INSIDE | 0.912702644 | 3.66E-15 |
| 48425 | A_68_P27997827 | Cuedc1 | chr11:087913300-087913355 | PROMOTER | 0.788563575 | 3.7216E-15 |
| 126942 | A_68_P22378978 | Casq2 | chr3:102214849-102214904 | PROMOTER | 0.755600092 | 3.77833E-15 |
| 211272 | A_68_P26039925 | Adcy7 | chr8:091163310-091163356 | INSIDE | 0.826122305 | 3.8271E-15 |
| 3151 | A_68_P30348985 | Fbxl6 | chr15:076364251-076364295 | INSIDE | 0.804961434 | 3.8335E-15 |
| 46999 | A_68_P24389221 | Tas2r108 | chr6:040425331-040425390 | DOWNSTREAM | 1.082111692 | 3.88167E-15 |
| 3423 | A_68_P25264905 | Idh2 | chr7:079990091-079990139 | PROMOTER | 0.767082472 | 3.97367E-15 |
| 202880 | A_68_P32452513 | Mtm1 | chrX:067477740-067477795 | INSIDE | 0.973847211 | 4.20523E-15 |
| 98228 | A_68_P24290524 | Wnt16 | chr6:022238093-022238140 | PROMOTER | 0.74819485 | 4.28067E-15 |
| 197323 | A_68_P29936403 | Dct | chr14:116931985-116932044 | INSIDE | 1.005450328 | 4.28177E-15 |
| 239688 | A_68_P23457353 | Steap1 | chr5:005756172-005756227 | PROMOTER | 0.989790227 | 4.37167E-15 |
| 167048 | A_68_P24928962 | Itpr2 | chr6:146459015-146459062 | INSIDE | 0.788379268 | 4.401E-15 |
| 188217 | A_68_P22348499 | Hist2h4-Hist2h3c1 | chr3:096349789-096349848 | DIVERGENT_PROMOTER | 0.753097094 | 4.41167E-15 |
| 88828 | A_68_P20350260 | Prkag3 | chr1:074686356-074686402 | PROMOTER | 0.818018131 | 4.55367E-15 |
| 168358 | A_68_P27401919 | Nts | chr10:101923771-101923830 | PROMOTER | 0.827063668 | 4.5736E-15 |
| 190470 | A_68_P27897392 | Nlgn2 | chr11:069648907-069648959 | INSIDE | 0.767328903 | 4.629E-15 |
| 184378 | A_68_P27267023 | Pofut2 | chr10:076704026-076704083 | INSIDE | 0.816967011 | 4.67393E-15 |
| 96791 | A_68_P25362442 | Arrb1 | chr7:099423013-099423065 | INSIDE | 0.799676483 | 4.79933E-15 |
| 49379 | A_68_P29033179 | Shc3 | chr13:051584006-051584063 | PROMOTER | 0.790390414 | 4.86653E-15 |
| 147595 | A_68_P32051813 | Jak2 | chr19:029315319-029315378 | PROMOTER | 1.035946206 | 4.94187E-15 |
| 176820 | A_68_P20604268 | Thsd7b | chr1:131095986-131096045 | PROMOTER | 0.862381398 | 5.05011E-15 |
| 104965 | A_68_P25383933 | Olfr616 | chr7:103443108-103443167 | PROMOTER | 0.777443919 | 5.12667E-15 |
| 31993 | A_68_P27565284 | Aebp1 | chr11:005759877-005759925 | PROMOTER | 0.849507436 | 5.22267E-15 |
| 153776 | A_68_P30968213 | Kcne2 | chr16:092183051-092183110 | INSIDE | 0.794890901 | 5.28033E-15 |
| 2867 | A_68_P24141333 | Eif3s9 | chr5:140674155-140674214 | INSIDE | 0.951478132 | 5.30567E-15 |
| 22209 | A_68_P27290592 | Tle2 | chr10:080975740-080975790 | PROMOTER | 0.786210913 | 5.38E-15 |
| 57399 | A_68_P26128323 | Cmtm4 | chr8:107283074-107283130 | INSIDE | 1.081653756 | 5.41767E-15 |
| 106105 | A_68_P23260039 | Mfsd2 | chr4:122466291-122466337 | PROMOTER | 0.750910807 | 5.48667E-15 |
| 39266 | A_68_P28127005 | Kcnj16 | chr11:110781116-110781175 | PROMOTER | 0.87850532 | 5.49207E-15 |
| 103140 | A_68_P21572798 | Pla2g4b | chr2:119723758-119723806 | PROMOTER | 1.025683578 | 5.51633E-15 |
| 59552 | A_68_P28574343 | Actn1 | chr12:081108780-081108835 | INSIDE | 0.781357368 | 5.62867E-15 |
| 107713 | A_68_P31157826 | Bat3 | chr17:034744843-034744902 | INSIDE | 0.975768046 | 5.62903E-15 |
| 19379 | A_68_P25265680 | Vps33b | chr7:080143823-080143881 | INSIDE | 0.940132034 | 5.648E-15 |
| 157391 | A_68_P31151111 | Rgl2 | chr17:033548091-033548147 | INSIDE | 0.768724098 | 5.6988E-15 |
| 34619 | A_68_P31351288 | Slc30a6 | chr17:074297739-074297798 | PROMOTER | 0.782279535 | 6.20667E-15 |
| 191549 | A_68_P31959327 | Olfr1424 | chr19:012129178-012129237 | PROMOTER | 0.94263884 | 6.21333E-15 |
| 66174 | A_68_P24300384 | Slc13a1 | chr6:024120134-024120193 | PROMOTER | 0.771578173 | 6.36E-15 |
| 68268 | A_68_P25933420 | Tktl2 | chr8:069442520-069442572 | INSIDE | 0.789261707 | 6.45667E-15 |
| 109463 | A_68_P24760543 | Vhlh | chr6:113586058-113586117 | PROMOTER | 0.911596567 | 6.66667E-15 |
| 170823 | A_68_P28947146 | Fars2 | chr13:036127645-036127697 | INSIDE | 0.795213366 | 6.68703E-15 |
| 9474 | A_68_P28767027 | Wdr60 | chr12:116706249-116706300 | PROMOTER | 0.76885327 | 6.76823E-15 |
| 170732 | A_68_P26079991 | Dok4 | chr8:097763076-097763130 | INSIDE | 0.749027021 | 6.836E-15 |
| 86903 | A_68_P25006322 | EG210155 | chr7:024434885-024434937 | PROMOTER | 0.888627391 | 6.84E-15 |
| 19264 | A_68_P30493728 | Krt74 | chr15:101590556-101590612 | INSIDE | 0.818710942 | 6.8657E-15 |
| 81083 | A_68_P24839197 | Ppih | chr6:128437145-128437198 | DOWNSTREAM | 0.7702704 | 7.136E-15 |
| 222718 | A_68_P23996984 | Gm854 | chr5:113469000-113469056 | INSIDE | 1.007455213 | 7.25678E-15 |
| 87787 | A_68_P25114631 | Prmt3 | chr7:049647321-049647379 | INSIDE | 0.768138528 | 7.29667E-15 |
| 54671 | A_68_P26023661 | Man2b1 | chr8:087975784-087975837 | INSIDE | 0.817025149 | 7.32333E-15 |
| 39966 | A_68_P26227582 | Fbxo31-Map1lc3b | chr8:124466924-124466983 | DIVERGENT_PROMOTER | 0.873551411 | 7.41667E-15 |
| 104371 | A_68_P32401482 | Gpr101 | chrX:053852083-053852134 | PROMOTER | 0.80423716 | 7.76647E-15 |
| 224009 | A_68_P29448459 | Chchd1 | chr14:019490681-019490740 | PROMOTER | 0.964236203 | 7.8452E-15 |
| 150887 | A_68_P23720358 | Pcdh7 | chr5:058006815-058006868 | INSIDE | 0.787890385 | 7.87033E-15 |
| 52775 | A_68_P29033182 | Shc3 | chr13:051584451-051584510 | PROMOTER | 0.774359119 | 8.04E-15 |
| 3314 | A_68_P32127017 | Got1 | chr19:043575697-043575756 | INSIDE | 0.779090377 | 8.0747E-15 |
| 123008 | A_68_P25002847 | V1rd13 | chr7:023602186-023602241 | PROMOTER | 0.996687974 | 8.08367E-15 |
| 53268 | A_68_P27849302 | Epn2 | chr11:061398229-061398288 | PROMOTER | 1.011344515 | 8.14041E-15 |
| 73524 | A_68_P28614759 | Tmed8 | chr12:088090515-088090574 | PROMOTER | 0.946334845 | 8.54037E-15 |
| 118471 | A_68_P30252126 | Mtss1 | chr15:058910127-058910183 | INSIDE | 0.946781858 | 9.03377E-15 |
| 137661 | A_68_P21742425 | Nanp | chr2:150729398-150729457 | INSIDE | 0.783283562 | 9.08167E-15 |
| 123033 | A_68_P32289639 | Pctk1 | chrX:019846009-019846064 | INSIDE | 0.774863946 | 9.67193E-15 |
| 48254 | A_68_P25396784 | Olfr702 | chr7:106619149-106619208 | PROMOTER | 1.043170953 | 9.673E-15 |
| 105110 | A_68_P22357015 | Sec22b | chr3:097984125-097984182 | PROMOTER | 0.765693321 | 9.94E-15 |
| 71620 | A_68_P29599715 | Rnase9 | chr14:049962575-049962634 | INSIDE | 1.192148285 | 1.0026E-14 |
| 43787 | A_68_P26886011 | Higd1a | chr9:121704102-121704161 | INSIDE | 0.809192901 | 1.02531E-14 |
| 146278 | A_68_P20162932 | Nms | chr1:038878868-038878927 | PROMOTER | 1.282566947 | 1.03214E-14 |
| 83745 | A_68_P25391805 | Olfr691 | chr7:105214138-105214197 | PROMOTER | 0.821927336 | 1.0365E-14 |
| 183342 | A_68_P27226656 | Ank3 | chr10:069315413-069315472 | PROMOTER | 0.845005792 | 1.1E-14 |
| 40962 | A_68_P31934664 | Nrxn2 | chr19:006428736-006428780 | INSIDE | 0.829774243 | 1.10897E-14 |
| 117073 | A_68_P32145477 | As3mt | chr19:046760604-046760663 | PROMOTER | 0.840289735 | 1.12573E-14 |
| 121393 | A_68_P27644756 | Xpo1 | chr11:023155092-023155150 | PROMOTER | 0.815982531 | 1.17483E-14 |
| 173568 | A_68_P27286426 | Gadd45b | chr10:080335476-080335525 | DOWNSTREAM | 0.812788073 | 1.22915E-14 |
| 40840 | A_68_P22615590 | Uox | chr3:146538874-146538929 | INSIDE | 0.764144493 | 1.24467E-14 |
| 149930 | A_68_P29228445 | Homer1 | chr13:094409231-094409290 | INSIDE | 0.920818106 | 1.25036E-14 |
| 30993 | A_68_P21634292 | Pank2 | chr2:130975413-130975472 | INSIDE | 0.782122377 | 1.253E-14 |
| 81853 | A_68_P25693363 | Csmd1 | chr8:017537600-017537659 | PROMOTER | 0.759341331 | 1.26733E-14 |
| 231472 | A_68_P26372676 | BC038479 | chr9:026560756-026560815 | PROMOTER | 0.860780695 | 1.26867E-14 |
| 191315 | A_68_P32327917 | Lamp2 | chrX:034702821-034702880 | PROMOTER | 1.146991812 | 1.2728E-14 |
| 104771 | A_68_P24466839 | Gars | chr6:054968881-054968940 | INSIDE | 0.955929981 | 1.27407E-14 |
| 215852 | A_68_P30465078 | Slc38a2 | chr15:096527624-096527683 | INSIDE | 0.790523922 | 1.2971E-14 |
| 36963 | A_68_P24983879 | Ptgir | chr7:016063304-016063363 | PROMOTER | 0.910002031 | 1.32833E-14 |
| 169892 | A_68_P25415713 | Xlkd1 | chr7:110654159-110654215 | PROMOTER | 0.975279048 | 1.3347E-14 |
| 11327 | A_68_P29108023 | Zfp458 | chr13:067768184-067768229 | PROMOTER | 0.818132796 | 1.33889E-14 |
| 9195 | A_68_P25201725 | Arrdc4 | chr7:068620181-068620235 | INSIDE | 0.749744914 | 1.34017E-14 |
| 189776 | A_68_P27845438 | Gtlf3b | chr11:060729447-060729496 | INSIDE | 0.767035902 | 1.34959E-14 |
| 115777 | A_68_P22311013 | Nes | chr3:088058819-088058865 | INSIDE | 0.767860056 | 1.35015E-14 |
| 140303 | A_68_P25093407 | Dhdh | chr7:045356729-045356773 | INSIDE | 0.74903819 | 1.36623E-14 |
| 25269 | A_68_P22323309 | Tpm3 | chr3:090158158-090158211 | PROMOTER | 0.925654049 | 1.371E-14 |
| 203202 | A_68_P23409707 | Per3 | chr4:149886446-149886505 | INSIDE | 0.793392757 | 1.39667E-14 |
| 46644 | A_68_P25381468 | Olfr575-Olfr576 | chr7:102838002-102838061 | DIVERGENT_PROMOTER | 0.795450929 | 1.4E-14 |
| 147529 | A_68_P25501260 | Apob48r | chr7:126379925-126379973 | INSIDE | 0.788004384 | 1.406E-14 |
| 37273 | A_68_P25386228 | Olfr640 | chr7:103895283-103895342 | INSIDE | 0.769225006 | 1.43763E-14 |
| 205928 | A_68_P31942646 | Slc22a6 | chr19:008682366-008682415 | PROMOTER | 0.772884059 | 1.4486E-14 |
| 197382 | A_68_P28961241 | Txndc5 | chr13:038540761-038540820 | PROMOTER | 0.810691516 | 1.4486E-14 |
| 51174 | A_68_P27572175 | Igfbp1 | chr11:007096294-007096348 | PROMOTER | 0.819867073 | 1.45746E-14 |
| 160087 | A_68_P25005663 | Zfp575 | chr7:024296537-024296589 | PROMOTER | 0.795035514 | 1.48933E-14 |
| 91381 | A_68_P20638507 | Rnpep | chr1:137099580-137099639 | INSIDE | 0.925181603 | 1.49167E-14 |
| 187749 | A_68_P27736827 | Mat2b | chr11:040534729-040534788 | INSIDE | 0.88155327 | 1.51533E-14 |
| 85643 | A_68_P21082823 | Dbh | chr2:026987707-026987757 | INSIDE | 0.759261611 | 1.56867E-14 |
| 94897 | A_68_P25083512 | Siglece | chr7:043529986-043530045 | PROMOTER | 0.84755876 | 1.578E-14 |
| 190111 | A_68_P28301969 | Rsad2-Tyki | chr12:027045912-027045971 | DIVERGENT_PROMOTER | 1.28522618 | 1.6272E-14 |
| 24602 | A_68_P25295797 | Folh1 | chr7:086651355-086651404 | INSIDE | 0.907962238 | 1.62946E-14 |
| 35548 | A_68_P32598403 | Pou3f4 | chrX:107019393-107019442 | DOWNSTREAM | 0.798802394 | 1.65836E-14 |
| 30872 | A_68_P20452340 | Gpr35 | chr1:094804374-094804428 | PROMOTER | 0.760833209 | 1.662E-14 |
| 39760 | A_68_P21868403 | Zbp1 | chr2:172866346-172866399 | PROMOTER | 0.760257024 | 1.683E-14 |
| 15689 | A_68_P29526801 | Gdf10 | chr14:032747624-032747678 | PROMOTER | 0.869348888 | 1.68497E-14 |
| 184108 | A_68_P32142333 | Pitx3-Gbf1 | chr19:046205044-046205103 | DIVERGENT_PROMOTER | 0.749438026 | 1.68998E-14 |
| 148893 | A_68_P31626765 | Pcdha12 | chr18:037147181-037147235 | INSIDE | 0.863593194 | 1.69073E-14 |
| 199776 | A_68_P23347860 | Pla2g2d | chr4:138048953-138048999 | INSIDE | 0.862877534 | 1.69233E-14 |
| 34571 | A_68_P27564957 | Dbnl | chr11:005689133-005689181 | INSIDE | 0.818605847 | 1.76766E-14 |
| 177217 | A_68_P31401128 | Haao | chr17:083754792-083754845 | INSIDE | 0.878150106 | 1.7879E-14 |
| 230785 | A_68_P30968888 | Dscr1 | chr16:092290701-092290753 | PROMOTER | 0.797525132 | 1.832E-14 |
| 115463 | A_68_P29700043 | Sorbs3 | chr14:068947829-068947888 | PROMOTER | 1.047165503 | 1.8881E-14 |
| 69138 | A_68_P30371103 | Atf4 | chr15:080084469-080084528 | INSIDE | 0.847602947 | 1.90327E-14 |
| 131876 | A_68_P24172316 | Gsh1 | chr5:147498910-147498956 | PROMOTER | 0.836256272 | 1.9047E-14 |
| 213031 | A_68_P23807609 | Pdgfra | chr5:075432728-075432785 | PROMOTER | 0.879521077 | 1.918E-14 |
| 119931 | A_68_P21098336 | Rapgef1 | chr2:029441003-029441062 | PROMOTER | 0.780006528 | 2E-14 |
| 51736 | A_68_P25186091 | Snrpa1 | chr7:065934496-065934555 | PROMOTER | 1.063267878 | 2.02927E-14 |
| 145211 | A_68_P32087885 | Htr7 | chr19:036121295-036121354 | INSIDE | 0.937156374 | 2.1791E-14 |
| 24993 | A_68_P30474086 | Olfr285 | chr15:098141046-098141102 | INSIDE | 0.76271828 | 2.18417E-14 |
| 107554 | A_68_P25914013 | Anxa10 | chr8:065017356-065017415 | PROMOTER | 0.94110047 | 2.269E-14 |
| 181451 | A_68_P25949958 | Cspg3 | chr8:073048623-073048680 | INSIDE | 0.77713646 | 2.328E-14 |
| 82661 | A_68_P31553003 | Nol4 | chr18:023183400-023183459 | PROMOTER | 0.764321348 | 2.34447E-14 |
| 32299 | A_68_P29447516 | Ppp3cb | chr14:019335694-019335749 | PROMOTER | 0.815415658 | 2.35817E-14 |
| 227260 | A_68_P24010690 | Rab35 | chr5:115894076-115894127 | INSIDE | 0.815079183 | 2.43833E-14 |
| 34283 | A_68_P24569982 | Reg2 | chr6:078335814-078335873 | INSIDE | 0.812114439 | 2.48267E-14 |
| 134867 | A_68_P22081031 | Slc25a31 | chr3:040805300-040805345 | PROMOTER | 0.800952716 | 2.53274E-14 |
| 141648 | A_68_P24434491 | Npy | chr6:049749159-049749216 | PROMOTER | 0.991578593 | 2.54343E-14 |
| 36427 | A_68_P28328441 | Acp1 | chr12:031496728-031496787 | INSIDE | 0.844383631 | 2.69857E-14 |
| 101893 | A_68_P30728109 | Tagln3 | chr16:045643222-045643280 | INSIDE | 1.043420924 | 2.70183E-14 |
| 96854 | A_68_P25476915 | Gga2 | chr7:121811735-121811792 | INSIDE | 0.780754269 | 2.77333E-14 |
| 236240 | A_68_P21394777 | Smtnl1 | chr2:084621298-084621354 | INSIDE | 0.771245178 | 2.81433E-14 |
| 178507 | A_68_P31625131 | Eif4ebp3 | chr18:036788701-036788760 | PROMOTER | 0.75634127 | 2.86677E-14 |
| 17698 | A_68_P28752311 | Pld4 | chr12:113207457-113207510 | PROMOTER | 0.897381838 | 2.92383E-14 |
| 28734 | A_68_P21321472 | Itga6 | chr2:071585896-071585947 | PROMOTER | 0.781246137 | 2.928E-14 |
| 193927 | A_68_P31161474 | Dhx16 | chr17:035489822-035489868 | INSIDE | 0.755191382 | 2.92867E-14 |
| 20997 | A_68_P21040751 | Commd3 | chr2:018590974-018591033 | INSIDE | 0.843547919 | 2.97567E-14 |
| 209599 | A_68_P27194560 | Lrrtm3 | chr10:063490287-063490346 | PROMOTER | 0.746710268 | 3.01867E-14 |
| 169855 | A_68_P23284745 | Gjb3 | chr4:126832981-126833025 | PROMOTER | 0.801423232 | 3.3944E-14 |
| 189406 | A_68_P26235252 | Cbfa2t3h | chr8:125586010-125586069 | PROMOTER | 0.908933197 | 3.473E-14 |
| 69641 | A_68_P32535332 | Pcyt1b | chrX:089928641-089928700 | INSIDE | 0.794842093 | 3.58767E-14 |
| 68662 | A_68_P21476815 | Trim44 | chr2:102200520-102200579 | INSIDE | 0.966903113 | 3.59067E-14 |
| 239235 | A_68_P20630971 | Fmod | chr1:135854550-135854609 | INSIDE | 0.785558037 | 3.62733E-14 |
| 24059 | A_68_P24128739 | Zkscan1 | chr5:138315373-138315423 | INSIDE | 0.931513868 | 3.708E-14 |
| 111197 | A_68_P29532477 | Grid1 | chr14:033722791-033722837 | INSIDE | 0.761939235 | 3.73267E-14 |
| 57151 | A_68_P30650122 | Pigz | chr16:031849499-031849558 | PROMOTER | 1.023430897 | 3.76732E-14 |
| 10949 | A_68_P26825757 | Myl3 | chr9:110610553-110610607 | INSIDE | 0.867687913 | 4.00889E-14 |
| 231767 | A_68_P29062344 | Tgfbi | chr13:056619961-056620009 | INSIDE | 0.849113238 | 4.0432E-14 |
| 24639 | A_68_P28185113 | Gcgr | chr11:120349279-120349335 | PROMOTER | 0.754914944 | 4.137E-14 |
| 227590 | A_68_P21604412 | Shc4 | chr2:125419146-125419201 | PROMOTER | 1.021725484 | 4.139E-14 |
| 18373 | A_68_P27788196 | Hnrph1 | chr11:050220251-050220306 | PROMOTER | 0.888964213 | 4.18123E-14 |
| 84947 | A_68_P26079274 | Ccl22 | chr8:097636739-097636787 | INSIDE | 0.786392989 | 4.23267E-14 |
| 67197 | A_68_P24594845 | Cd207 | chr6:083643490-083643543 | PROMOTER | 0.75823066 | 4.23733E-14 |
| 218408 | A_68_P29529268 | Glud1 | chr14:033142189-033142248 | INSIDE | 0.895994529 | 4.4803E-14 |
| 238018 | A_68_P27101050 | Cd24a | chr10:043268049-043268095 | INSIDE | 0.781252059 | 4.55023E-14 |
| 46833 | A_68_P26716258 | Adamts7 | chr9:089962974-089963030 | INSIDE | 0.801867413 | 5.15033E-14 |
| 199379 | A_68_P27848691 | Mapk7-Eppb9 | chr11:061311687-061311746 | DIVERGENT_PROMOTER | 1.112852482 | 5.343E-14 |
| 39027 | A_68_P20916917 | Esrrg | chr1:189695191-189695250 | PROMOTER | 0.817059019 | 5.37667E-14 |
| 22284 | A_68_P31613829 | Brd8 | chr18:034746515-034746566 | INSIDE | 0.793860582 | 5.46287E-14 |
| 1156 | A_68_P24864670 | Ddx47 | chr6:134977630-134977681 | INSIDE | 0.751593886 | 5.617E-14 |
| 29194 | A_68_P27168756 | Oit3 | chr10:058841264-058841315 | PROMOTER | 1.053912632 | 5.669E-14 |
| 221410 | A_68_P21774571 | Dsn1 | chr2:156688907-156688966 | INSIDE | 0.884492369 | 5.67133E-14 |
| 204555 | A_68_P28225588 | Wdr35 | chr12:009001050-009001103 | INSIDE | 0.945792602 | 5.68867E-14 |
| 80566 | A_68_P28899214 | Hdgfl1 | chr13:026778529-026778587 | PROMOTER | 0.787543086 | 5.87167E-14 |
| 209746 | A_68_P30966890 | Slc5a3 | chr16:091966583-091966639 | INSIDE | 0.861239281 | 5.87867E-14 |
| 126433 | A_68_P26815976 | Arih2 | chr9:108506955-108507014 | PROMOTER | 0.875705188 | 5.9222E-14 |
| 232852 | A_68_P21400243 | Olfr1030 | chr2:085785268-085785327 | INSIDE | 0.911373638 | 6.04E-14 |
| 197691 | A_68_P26974291 | Ccdc28a | chr10:017926190-017926247 | PROMOTER | 0.868300547 | 6.10097E-14 |
| 188055 | A_68_P24129259 | Taf6 | chr5:138416329-138416388 | INSIDE | 1.233093045 | 6.23847E-14 |
| 207144 | A_68_P23898558 | Ccni | chr5:094280454-094280513 | INSIDE | 0.847099461 | 6.39133E-14 |
| 13998 | A_68_P29267254 | Serf1 | chr13:101210820-101210879 | INSIDE | 0.811384523 | 6.56592E-14 |
| 144584 | A_68_P27258691 | Gstt1 | chr10:075242482-075242538 | PROMOTER | 0.953182688 | 6.8694E-14 |
| 240112 | A_68_P22606628 | Odf2l | chr3:145053356-145053415 | PROMOTER | 0.881903773 | 6.99663E-14 |
| 130240 | A_68_P20656183 | Atp6v1g3 | chr1:140091724-140091783 | INSIDE | 0.950154413 | 7.01333E-14 |
| 93936 | A_68_P20611022 | C4bp | chr1:132492555-132492614 | PROMOTER | 0.787751565 | 7.18333E-14 |
| 136782 | A_68_P28100257 | Ddx42 | chr11:106036256-106036315 | INSIDE | 1.02386147 | 7.25967E-14 |
| 152896 | A_68_P30677457 | Iqcb1 | chr16:036750240-036750299 | INSIDE | 0.750666491 | 7.33833E-14 |
| 97611 | A_68_P25595134 | Tnnt3 | chr7:142308647-142308705 | INSIDE | 1.496293527 | 7.42287E-14 |
| 66274 | A_68_P28171059 | Lgals3bp | chr11:118216927-118216986 | INSIDE | 0.868261567 | 7.61613E-14 |
| 2051 | A_68_P27837351 | Zfp496 | chr11:059327743-059327787 | PROMOTER | 0.764553926 | 7.6615E-14 |
| 228215 | A_68_P29659276 | Ctsb | chr14:062077509-062077564 | INSIDE | 0.805114923 | 7.78687E-14 |
| 116804 | A_68_P29508063 | Tmem110 | chr14:029649839-029649898 | PROMOTER | 0.912600927 | 7.80573E-14 |
| 239825 | A_68_P27791272 | Zfp2 | chr11:050762296-050762355 | PROMOTER | 0.767103692 | 7.865E-14 |
| 153868 | A_68_P23331881 | Rpl11 | chr4:135330067-135330121 | PROMOTER | 0.761605954 | 7.90667E-14 |
| 74665 | A_68_P20229146 | Inpp1 | chr1:052763614-052763673 | PROMOTER | 0.824997157 | 8.32667E-14 |
| 40693 | A_68_P24267826 | Met | chr6:017440057-017440116 | PROMOTER | 1.01320576 | 8.32773E-14 |
| 214437 | A_68_P28049271 | Kpnb1 | chr11:097008193-097008252 | PROMOTER | 1.032675636 | 8.47533E-14 |
| 41082 | A_68_P26313285 | Mtmr2 | chr9:013498193-013498252 | PROMOTER | 0.914245692 | 8.80423E-14 |
| 57260 | A_68_P20837434 | Igsf4b | chr1:175175769-175175828 | INSIDE | 0.814934601 | 9.51333E-14 |
| 41337 | A_68_P28011480 | Hlf | chr11:090208486-090208542 | PROMOTER | 0.826974814 | 9.61733E-14 |
| 151825 | A_68_P28063038 | Krtap3-3 | chr11:099366467-099366526 | INSIDE | 1.019059182 | 1.02936E-13 |
| 63407 | A_68_P21740938 | Pygb | chr2:150475445-150475504 | PROMOTER | 0.761741033 | 1.058E-13 |
| 182229 | A_68_P27020979 | Arhgap18 | chr10:026463899-026463958 | INSIDE | 0.75080661 | 1.06013E-13 |
| 209485 | A_68_P23237381 | Artn | chr4:117427658-117427705 | INSIDE | 0.78905674 | 1.07633E-13 |
| 175513 | A_68_P21044507 | Msrb2 | chr2:019286687-019286745 | PROMOTER | 0.801788621 | 1.12E-13 |
| 146171 | A_68_P22341996 | Psmd4 | chr3:095128925-095128984 | PROMOTER | 0.779477427 | 1.19793E-13 |
| 92092 | A_68_P32118942 | BC023055-Pi4k2a | chr19:042136975-042137034 | DIVERGENT_PROMOTER | 0.827344722 | 1.20667E-13 |
| 4088 | A_68_P29447130 | Anxa7 | chr14:019272093-019272152 | PROMOTER | 0.797303856 | 1.22751E-13 |
| 19366 | A_68_P21630504 | Oxt | chr2:130265120-130265179 | PROMOTER | 1.013830502 | 1.23723E-13 |
| 36388 | A_68_P29011298 | Aof1 | chr13:047057212-047057271 | INSIDE | 0.85560546 | 1.28917E-13 |
| 96609 | A_68_P31150925 | Daxx | chr17:033521071-033521125 | INSIDE | 1.144442134 | 1.31054E-13 |
| 32898 | A_68_P21332900 | Chn1 | chr2:073464157-073464216 | PROMOTER | 1.003011364 | 1.32333E-13 |
| 236268 | A_68_P24760049 | Tmem111 | chr6:113496661-113496720 | INSIDE | 0.875720786 | 1.4075E-13 |
| 148006 | A_68_P31830412 | Mbd1 | chr18:074395123-074395182 | INSIDE | 0.897829104 | 1.41049E-13 |
| 128217 | A_68_P32464962 | Pdzd4 | chrX:070074771-070074830 | INSIDE | 1.020045845 | 1.42215E-13 |
| 168150 | A_68_P32387379 | Mospd1 | chrX:049617677-049617736 | PROMOTER | 0.858412292 | 1.54873E-13 |
| 95868 | A_68_P26238364 | Dpep1 | chr8:126073851-126073900 | INSIDE | 0.78478096 | 1.56773E-13 |
| 157724 | A_68_P24925085 | Rassf8 | chr6:145760243-145760300 | PROMOTER | 1.024422783 | 1.5824E-13 |
| 92791 | A_68_P24808001 | M6pr | chr6:122277040-122277099 | INSIDE | 0.78698936 | 1.66887E-13 |
| 88938 | A_68_P24983797 | Gng8 | chr7:016049171-016049230 | PROMOTER | 0.821672331 | 1.72367E-13 |
| 145469 | A_68_P23222821 | Stil | chr4:114500036-114500095 | INSIDE | 0.839148989 | 1.75333E-13 |
| 221956 | A_68_P25574407 | Mapk1ip1 | chr7:138682998-138683057 | INSIDE | 0.765248931 | 1.78333E-13 |
| 99470 | A_68_P23904960 | Cnot6l | chr5:096400617-096400676 | INSIDE | 0.755882093 | 1.78567E-13 |
| 240054 | A_68_P26144619 | Nqo1 | chr8:110293307-110293366 | PROMOTER | 0.785917134 | 1.84247E-13 |
| 120471 | A_68_P24428084 | Rarres2 | chr6:048501178-048501231 | INSIDE | 0.799566863 | 1.87333E-13 |
| 129325 | A_68_P24158554 | Jtv1 | chr5:144162537-144162596 | INSIDE | 0.762769932 | 1.87653E-13 |
| 146956 | A_68_P21524739 | Olfr1278 | chr2:111093577-111093636 | INSIDE | 0.878428885 | 1.91143E-13 |
| 188594 | A_68_P23465843 | Steap4 | chr5:007966143-007966201 | PROMOTER | 0.750116527 | 1.9134E-13 |
| 216905 | A_68_P28707016 | Glrx5 | chr12:105433598-105433647 | PROMOTER | 0.989968913 | 1.97447E-13 |
| 70551 | A_68_P32326096 | Rhox10 | chrX:034310810-034310869 | PROMOTER | 0.763254829 | 2.0546E-13 |
| 184254 | A_68_P25063550 | Zfp536 | chr7:037484581-037484638 | PROMOTER | 0.746505683 | 2.10067E-13 |
| 36561 | A_68_P28729898 | Yy1 | chr12:109237437-109237496 | PROMOTER | 0.778826408 | 2.12205E-13 |
| 108778 | A_68_P24861354 | Bcl2l14 | chr6:134358519-134358573 | PROMOTER | 0.74786524 | 2.16733E-13 |
| 45137 | A_68_P23573885 | Cenpa | chr5:030942817-030942876 | PROMOTER | 0.857695625 | 2.18277E-13 |
| 88646 | A_68_P26779745 | Ephb1 | chr9:102213195-102213254 | PROMOTER | 0.838108646 | 2.256E-13 |
| 238369 | A_68_P24541270 | Usp39-Tmem150 | chr6:072284041-072284094 | DIVERGENT_PROMOTER | 0.868973521 | 2.30883E-13 |
| 22948 | A_68_P27540162 | Spryd4-Mip | chr10:127624143-127624202 | DIVERGENT_PROMOTER | 0.955792307 | 2.34602E-13 |
| 173689 | A_68_P30675972 | Casr | chr16:036483714-036483772 | PROMOTER | 0.752166264 | 2.38467E-13 |
| 20177 | A_68_P22406456 | Kcnc4 | chr3:107585846-107585899 | INSIDE | 0.869607073 | 2.42833E-13 |
| 19035 | A_68_P32370306 | Igsf1 | chrX:046040767-046040826 | PROMOTER | 1.166746844 | 2.45569E-13 |
| 54130 | A_68_P27287585 | Zbtb7a | chr10:080534062-080534121 | PROMOTER | 0.805305696 | 2.54182E-13 |
| 220446 | A_68_P28902666 | Prlpc2 | chr13:027574995-027575054 | PROMOTER | 0.754547087 | 2.55386E-13 |
| 10556 | A_68_P20193899 | Gulp1 | chr1:044492659-044492718 | PROMOTER | 0.96590424 | 2.61167E-13 |
| 132829 | A_68_P29513201 | Btd | chr14:030472732-030472791 | INSIDE | 0.862572489 | 2.736E-13 |
| 170652 | A_68_P32130509 | Chuk | chr19:044165401-044165453 | PROMOTER | 0.895370025 | 2.75408E-13 |
| 172439 | A_68_P32241175 | Gata1 | chrX:007124069-007124124 | INSIDE | 0.823133978 | 2.76803E-13 |
| 38784 | A_68_P30572260 | Ube2l3 | chr16:017064904-017064958 | DOWNSTREAM | 0.754457262 | 2.78932E-13 |
| 53969 | A_68_P25095582 | Grin2d-Kdelr1 | chr7:045739435-045739480 | DIVERGENT_PROMOTER | 0.762416398 | 2.93456E-13 |
| 190453 | A_68_P32289005 | Rgn | chrX:019704724-019704783 | PROMOTER | 0.75392444 | 2.9576E-13 |
| 116694 | A_68_P29649844 | Ebpl | chr14:060314772-060314828 | PROMOTER | 0.775729335 | 3.01135E-13 |
| 86345 | A_68_P30507825 | Dnase1 | chr16:003947662-003947717 | PROMOTER | 0.792086439 | 3.2505E-13 |
| 237435 | A_68_P23440905 | Mxra8 | chr4:154683786-154683838 | INSIDE | 0.805552235 | 3.26177E-13 |
| 158093 | A_68_P31789939 | Gnal | chr18:067214904-067214953 | INSIDE | 0.919050241 | 3.34896E-13 |
| 150512 | A_68_P28547072 | Kcnh5 | chr12:076094472-076094531 | INSIDE | 0.756327394 | 3.40083E-13 |
| 48582 | A_68_P27918655 | Olfr397 | chr11:073779494-073779553 | PROMOTER | 0.796390311 | 3.47032E-13 |
| 71743 | A_68_P26886369 | Cyp8b1 | chr9:121767380-121767439 | PROMOTER | 0.807342216 | 3.5146E-13 |
| 61862 | A_68_P23917048 | Fgf5 | chr5:098496669-098496726 | INSIDE | 0.88225321 | 3.51667E-13 |
| 192295 | A_68_P30788355 | Col8a1 | chr16:057677907-057677966 | PROMOTER | 0.91838599 | 3.56867E-13 |
| 66231 | A_68_P21007453 | Il15ra | chr2:011623247-011623299 | PROMOTER | 0.766551746 | 3.60303E-13 |
| 119676 | A_68_P28054319 | Fbxl20 | chr11:097899972-097900026 | INSIDE | 0.855929215 | 3.64237E-13 |
| 216281 | A_68_P32553109 | Stard8 | chrX:095241154-095241208 | PROMOTER | 0.819374583 | 3.673E-13 |
| 179819 | A_68_P28882233 | Hist1h3e | chr13:023571740-023571799 | PROMOTER | 0.799312335 | 3.897E-13 |
| 17570 | A_68_P30658275 | Slc12a8 | chr16:033432644-033432692 | PROMOTER | 0.78217084 | 3.94533E-13 |
| 66705 | A_68_P25583537 | Olfr530 | chr7:140225280-140225335 | PROMOTER | 0.790784112 | 3.94743E-13 |
| 114074 | A_68_P29959659 | Tm9sf2 | chr14:121240829-121240883 | PROMOTER | 0.769106959 | 4.1167E-13 |
| 52553 | A_68_P24603577 | Emx1 | chr6:085155616-085155663 | INSIDE | 0.882362647 | 4.45333E-13 |
| 3533 | A_68_P24774345 | Plxnd1 | chr6:115964898-115964952 | PROMOTER | 0.93388961 | 4.59667E-13 |
| 128623 | A_68_P26350516 | Elof1 | chr9:021864531-021864588 | INSIDE | 0.75144801 | 4.60411E-13 |
| 83807 | A_68_P23993068 | Sez6l | chr5:112818979-112819033 | PROMOTER | 0.788298167 | 4.61E-13 |
| 184376 | A_68_P28066783 | Krt14 | chr11:100025223-100025272 | PROMOTER | 1.068911517 | 4.83386E-13 |
| 113059 | A_68_P24324875 | Rbm28 | chr6:029104994-029105041 | INSIDE | 0.771176035 | 4.90067E-13 |
| 187805 | A_68_P25960293 | Ap1m1 | chr8:075169634-075169692 | INSIDE | 0.82294721 | 4.99333E-13 |
| 208946 | A_68_P29080118 | Agtpbp1 | chr13:059552702-059552754 | INSIDE | 1.399254866 | 5.027E-13 |
| 208823 | A_68_P24299376 | Tas2r118 | chr6:023921741-023921795 | PROMOTER | 1.011596386 | 5.18507E-13 |
| 215785 | A_68_P21818564 | Wfdc2 | chr2:164253573-164253626 | PROMOTER | 0.955437193 | 5.27967E-13 |
| 207672 | A_68_P30213276 | Eif3s3 | chr15:051693767-051693826 | INSIDE | 0.757295291 | 5.292E-13 |
| 84844 | A_68_P20745880 | Ralgps2 | chr1:158778014-158778073 | PROMOTER | 0.779502949 | 5.30667E-13 |
| 6701 | A_68_P23570399 | Il6 | chr5:030344532-030344586 | INSIDE | 0.799840924 | 5.37667E-13 |
| 216716 | A_68_P24396733 | Tas2r139 | chr6:042072166-042072225 | DOWNSTREAM | 0.879334532 | 5.39347E-13 |
| 131124 | A_68_P32227611 | Sfxn4 | chr19:060918549-060918608 | PROMOTER | 0.903324632 | 5.63656E-13 |
| 44108 | A_68_P21873471 | Npepl1 | chr2:173753473-173753517 | INSIDE | 0.748738351 | 5.67028E-13 |
| 158045 | A_68_P31934042 | Men1 | chr19:006336469-006336528 | INSIDE | 0.771400048 | 5.7354E-13 |
| 198215 | A_68_P28730643 | Wars | chr12:109346967-109347026 | PROMOTER | 0.863600469 | 6.05057E-13 |
| 231114 | A_68_P24624553 | Podxl2 | chr6:088812887-088812946 | INSIDE | 0.784743312 | 6.21333E-13 |
| 86151 | A_68_P25644696 | Tmem28 | chr8:009770263-009770321 | INSIDE | 0.767138784 | 6.28333E-13 |
| 170621 | A_68_P21483580 | Cat | chr2:103286036-103286080 | PROMOTER | 0.825027049 | 6.39407E-13 |
| 238541 | A_68_P21400074 | Olfr1028 | chr2:085746439-085746498 | PROMOTER | 0.753062864 | 6.40833E-13 |
| 197771 | A_68_P22400966 | Chi3l3 | chr3:106298660-106298717 | PROMOTER | 0.823411512 | 6.43733E-13 |
| 50300 | A_68_P24882360 | Mgst1 | chr6:138105303-138105362 | INSIDE | 0.88416981 | 6.47433E-13 |
| 112629 | A_68_P25391252 | Olfr686 | chr7:105077612-105077669 | INSIDE | 0.780619584 | 6.541E-13 |
| 130689 | A_68_P28871058 | Olfr1370 | chr13:021078583-021078642 | DOWNSTREAM | 0.947440816 | 6.601E-13 |
| 89150 | A_68_P20335902 | Tmem169 | chr1:072219122-072219181 | INSIDE | 0.816580407 | 6.60667E-13 |
| 70144 | A_68_P26349578 | BC010787 | chr9:021677568-021677623 | INSIDE | 0.755507343 | 6.61167E-13 |
| 57459 | A_68_P31955585 | Ms4a7 | chr19:011401539-011401598 | INSIDE | 0.872933003 | 6.6743E-13 |
| 77767 | A_68_P28248081 | Ddx1 | chr12:013275301-013275356 | PROMOTER | 0.755089113 | 6.72457E-13 |
| 114437 | A_68_P24879630 | Eps8 | chr6:137617517-137617573 | PROMOTER | 0.760271544 | 6.851E-13 |
| 22900 | A_68_P26756460 | Clstn2 | chr9:097845380-097845439 | PROMOTER | 0.787339295 | 6.92437E-13 |
| 237091 | A_68_P31791344 | Cidea | chr18:067466920-067466979 | PROMOTER | 0.787815079 | 7.0177E-13 |
| 92022 | A_68_P30229372 | Enpp2 | chr15:054754182-054754241 | PROMOTER | 0.898882832 | 7.023E-13 |
| 3718 | A_68_P26203073 | Plcg2 | chr8:120379370-120379417 | PROMOTER | 0.795000319 | 7.06E-13 |
| 36743 | A_68_P32470777 | Mpp1 | chrX:071383083-071383142 | INSIDE | 0.942791464 | 7.07401E-13 |
| 90388 | A_68_P23966388 | Brdt | chr5:107571197-107571256 | PROMOTER | 0.768653621 | 7.14E-13 |
| 99518 | A_68_P22342454 | Tnfaip8l2 | chr3:095226803-095226862 | INSIDE | 0.896336238 | 7.23667E-13 |
| 109534 | A_68_P20819695 | Hsd17b7 | chr1:171809108-171809167 | PROMOTER | 0.963137283 | 7.41333E-13 |
| 151066 | A_68_P32721216 | Lhfpl1 | chrX:140593104-140593156 | INSIDE | 0.748906059 | 7.7154E-13 |
| 157712 | A_68_P21615842 | Bub1 | chr2:127521164-127521223 | INSIDE | 0.793573832 | 7.72633E-13 |
| 112986 | A_68_P26803399 | Abhd14a | chr9:106301989-106302039 | INSIDE | 0.798284256 | 7.73333E-13 |
| 12038 | A_68_P25922751 | Tll1 | chr8:067096129-067096188 | INSIDE | 1.010955123 | 7.92133E-13 |
| 99392 | A_68_P23314127 | BC013712 | chr4:132069981-132070039 | PROMOTER | 0.958208347 | 7.93557E-13 |
| 82551 | A_68_P27104604 | Atg5 | chr10:043958215-043958274 | INSIDE | 0.829131059 | 8.5494E-13 |
| 219974 | A_68_P21115133 | Lcn2-Ptges2 | chr2:032214356-032214407 | DIVERGENT_PROMOTER | 0.764281594 | 8.65133E-13 |
| 142319 | A_68_P28190425 | BC032265 | chr11:121238257-121238316 | INSIDE | 0.753052507 | 8.65527E-13 |
| 135555 | A_68_P20630352 | Prelp | chr1:135741337-135741396 | PROMOTER | 1.003907599 | 8.68337E-13 |
| 177606 | A_68_P31663349 | Spink3 | chr18:043865477-043865536 | PROMOTER | 0.905209055 | 8.77587E-13 |
| 189579 | A_68_P30978876 | Cldn14 | chr16:093897818-093897877 | PROMOTER | 0.784214623 | 9.07543E-13 |
| 11305 | A_68_P29619780 | Ltb4r1 | chr14:054719252-054719310 | PROMOTER | 0.759038145 | 9.16407E-13 |
| 202234 | A_68_P21283770 | Grb14 | chr2:064821690-064821741 | INSIDE | 0.85278734 | 9.17667E-13 |
| 232404 | A_68_P30294148 | Sla | chr15:066645126-066645185 | PROMOTER | 0.773135697 | 9.35369E-13 |
| 151145 | A_68_P30475242 | Ccnt1 | chr15:098397088-098397147 | PROMOTER | 0.754459957 | 9.4629E-13 |
| 164579 | A_68_P28518518 | Nin | chr12:071029323-071029379 | INSIDE | 0.889454061 | 9.73917E-13 |
| 177196 | A_68_P26820666 | Camp | chr9:109706154-109706199 | INSIDE | 0.788752419 | 1.07933E-12 |
| 237840 | A_68_P23567831 | Hlxb9 | chr5:029814446-029814505 | PROMOTER | 0.851205402 | 1.08867E-12 |
| 239724 | A_68_P28880581 | V1rh16 | chr13:023244176-023244232 | PROMOTER | 0.844753871 | 1.09232E-12 |
| 127949 | A_68_P20567344 | Htr5b | chr1:123357349-123357402 | PROMOTER | 0.845055242 | 1.10439E-12 |
| 217826 | A_68_P27466127 | Trhde | chr10:114204916-114204965 | INSIDE | 0.752756681 | 1.13733E-12 |
| 84427 | A_68_P32388187 | Cxx1a | chrX:049840323-049840382 | PROMOTER | 0.942911134 | 1.16471E-12 |
| 165254 | A_68_P30006037 | Il7r | chr15:009477528-009477587 | PROMOTER | 0.963635051 | 1.16681E-12 |
| 195539 | A_68_P29730839 | Kctd4 | chr14:074683937-074683996 | PROMOTER | 0.953926339 | 1.17042E-12 |
| 164501 | A_68_P26744240 | AI427122 | chr9:095657479-095657538 | PROMOTER | 0.95295869 | 1.17413E-12 |
| 178714 | A_68_P25637645 | Efnb2 | chr8:008663623-008663682 | PROMOTER | 0.788848154 | 1.23273E-12 |
| 71072 | A_68_P20742463 | Nphs2 | chr1:158142094-158142153 | PROMOTER | 0.920808829 | 1.30189E-12 |
| 118841 | A_68_P30999101 | Mx1 | chr16:097573055-097573109 | PROMOTER | 0.812539454 | 1.30421E-12 |
| 143921 | A_68_P22694592 | Rps20 | chr4:003763787-003763846 | PROMOTER | 0.783899427 | 1.31347E-12 |
| 173695 | A_68_P30963204 | Ifnar1 | chr16:091375519-091375578 | INSIDE | 0.90933126 | 1.33897E-12 |
| 51443 | A_68_P25420930 | Galntl4 | chr7:111576542-111576596 | PROMOTER | 0.767433788 | 1.443E-12 |
| 224552 | A_68_P32467339 | Atp6ap1 | chrX:070550458-070550509 | INSIDE | 0.779947464 | 1.53243E-12 |
| 90720 | A_68_P25426500 | Tead1 | chr7:112548429-112548478 | INSIDE | 0.783081082 | 1.58957E-12 |
| 193725 | A_68_P25244601 | EG244071 | chr7:076286633-076286692 | INSIDE | 0.768514254 | 1.60507E-12 |
| 16579 | A_68_P27917308 | Olfr382 | chr11:073332681-073332740 | INSIDE | 0.963289238 | 1.62044E-12 |
| 100352 | A_68_P26023042 | Hook2 | chr8:087879453-087879508 | PROMOTER | 0.784190695 | 1.63953E-12 |
| 88940 | A_68_P31121141 | Def6 | chr17:027937044-027937094 | INSIDE | 0.90208144 | 1.64167E-12 |
| 5490 | A_68_P26322129 | Josd3 | chr9:015058207-015058266 | INSIDE | 0.803421138 | 1.68333E-12 |
| 237014 | A_68_P22647413 | Ptgfr | chr3:151773322-151773381 | INSIDE | 0.841213377 | 1.69833E-12 |
| 180828 | A_68_P29701951 | Reep4 | chr14:069279732-069279783 | PROMOTER | 0.771765546 | 1.71872E-12 |
| 81522 | A_68_P28071207 | Ptrf | chr11:100784973-100785028 | INSIDE | 0.786414306 | 1.76742E-12 |
| 96165 | A_68_P31473610 | Epc1 | chr18:006494491-006494550 | PROMOTER | 0.821274611 | 1.7693E-12 |
| 127711 | A_68_P27558682 | OTTMUSG00000005065 | chr11:004527577-004527636 | INSIDE | 0.912420942 | 1.89967E-12 |
| 18057 | A_68_P28538309 | Prkch | chr12:074502623-074502676 | PROMOTER | 0.750426175 | 1.90667E-12 |
| 169131 | A_68_P24537497 | Jmjd1a | chr6:071567860-071567919 | PROMOTER | 0.930184809 | 1.9152E-12 |
| 132079 | A_68_P29054310 | Rab24 | chr13:055329757-055329806 | INSIDE | 0.866944985 | 1.96267E-12 |
| 101318 | A_68_P26976016 | Nhsl1 | chr10:018214829-018214875 | INSIDE | 0.943005446 | 2.00756E-12 |
| 202817 | A_68_P22767944 | Ggh | chr4:020163353-020163411 | PROMOTER | 1.099860068 | 2.0253E-12 |
| 232275 | A_68_P21819270 | Spint4 | chr2:164389849-164389905 | INSIDE | 0.778554211 | 2.17377E-12 |
| 140346 | A_68_P27005228 | Rps12 | chr10:023477869-023477928 | PROMOTER | 1.177610044 | 2.22709E-12 |
| 233545 | A_68_P24400278 | Olfr450 | chr6:042746689-042746748 | PROMOTER | 1.010966916 | 2.316E-12 |
| 50968 | A_68_P30795087 | Olfr204 | chr16:059258956-059259015 | PROMOTER | 0.789834033 | 2.4481E-12 |
| 119369 | A_68_P26915920 | Plekhg1 | chr10:006473363-006473417 | INSIDE | 0.89928653 | 2.59933E-12 |
| 101717 | A_68_P26648638 | Hcrtr2 | chr9:076111868-076111927 | PROMOTER | 0.766240105 | 2.62218E-12 |
| 120881 | A_68_P23798797 | Sgcb | chr5:073926943-073926991 | PROMOTER | 0.820018511 | 2.625E-12 |
| 221668 | A_68_P31128443 | Tbc1d22b | chr17:029276169-029276228 | PROMOTER | 0.864807451 | 2.62892E-12 |
| 229901 | A_68_P30725564 | Btla | chr16:045138805-045138864 | PROMOTER | 0.961878583 | 2.68635E-12 |
| 79991 | A_68_P23615086 | Stk32b | chr5:038004782-038004833 | INSIDE | 0.801931876 | 2.936E-12 |
| 101212 | A_68_P31098030 | Pkd1 | chr17:024279703-024279760 | INSIDE | 0.967869889 | 2.95239E-12 |
| 214298 | A_68_P23506442 | Sema3c | chr5:017088212-017088271 | INSIDE | 0.824120135 | 3.06067E-12 |
| 221986 | A_68_P23091486 | C87499 | chr4:088108799-088108858 | PROMOTER | 1.385455089 | 3.1667E-12 |
| 36799 | A_68_P29621264 | Mcpt4 | chr14:055015179-055015229 | INSIDE | 1.063018036 | 3.27067E-12 |
| 171407 | A_68_P20170231 | Il1r1 | chr1:040170139-040170198 | INSIDE | 0.821015909 | 3.3E-12 |
| 77947 | A_68_P21911122 | Dnajc5 | chr2:181451461-181451520 | INSIDE | 0.804371246 | 3.44E-12 |
| 128660 | A_68_P31537145 | Dsc3 | chr18:020150362-020150420 | PROMOTER | 0.856064723 | 3.4837E-12 |
| 78820 | A_68_P24966290 | Zik1 | chr7:009371329-009371388 | PROMOTER | 0.797015818 | 3.54133E-12 |
| 121774 | A_68_P22341474 | Rfx5 | chr3:095042423-095042482 | INSIDE | 1.064642403 | 3.705E-12 |
| 102530 | A_68_P24023699 | Fbxo21 | chr5:118234806-118234865 | PROMOTER | 0.807348119 | 3.87333E-12 |
| 210340 | A_68_P29528336 | Ppyr1 | chr14:032981180-032981235 | INSIDE | 0.762699143 | 3.88887E-12 |
| 43883 | A_68_P23308786 | Mecr | chr4:131140696-131140755 | DOWNSTREAM | 1.012109356 | 3.907E-12 |
| 102954 | A_68_P25952102 | Klhl26 | chr8:073407621-073407680 | PROMOTER | 0.889263896 | 3.99303E-12 |
| 52530 | A_68_P20972057 | Ccdc3 | chr2:005055733-005055792 | INSIDE | 0.918294109 | 4.25E-12 |
| 137328 | A_68_P30058752 | Acot10 | chr15:020610541-020610600 | INSIDE | 0.830059732 | 4.29967E-12 |
| 217856 | A_68_P32099759 | Pde6c | chr19:038198529-038198583 | INSIDE | 0.829563661 | 4.32547E-12 |
| 68010 | A_68_P31929972 | Sipa1 | chr19:005661756-005661812 | INSIDE | 0.848697664 | 4.34678E-12 |
| 161010 | A_68_P23175447 | C8a | chr4:104376682-104376741 | PROMOTER | 0.907694385 | 4.35E-12 |
| 112355 | A_68_P31921509 | Rps6kb2 | chr19:004166487-004166537 | PROMOTER | 0.814801631 | 4.54503E-12 |
| 91930 | A_68_P26526541 | Idh3a | chr9:054381310-054381369 | PROMOTER | 0.779738763 | 4.56653E-12 |
| 31167 | A_68_P31661267 | Dpysl3 | chr18:043522363-043522422 | PROMOTER | 0.815394805 | 4.60287E-12 |
| 212554 | A_68_P27943723 | Og9x | chr11:078319446-078319499 | PROMOTER | 0.755853438 | 4.90267E-12 |
| 130447 | A_68_P20778502 | Vamp4 | chr1:164408494-164408553 | INSIDE | 0.969807972 | 5.11333E-12 |
| 54969 | A_68_P22694745 | Mos | chr4:003799736-003799791 | PROMOTER | 0.753133326 | 5.11538E-12 |
| 233975 | A_68_P31106114 | Narfl | chr17:025496759-025496813 | PROMOTER | 0.825035185 | 5.13414E-12 |
| 80321 | A_68_P27804840 | Shroom1 | chr11:053296163-053296219 | PROMOTER | 0.754199834 | 5.14126E-12 |
| 162631 | A_68_P28902246 | Prlpc1 | chr13:027445351-027445410 | INSIDE | 1.13986925 | 5.15748E-12 |
| 172491 | A_68_P32185193 | Pdcd4 | chr19:053953434-053953493 | INSIDE | 0.93422016 | 5.245E-12 |
| 211511 | A_68_P21940357 | Fabp5 | chr3:009992076-009992131 | PROMOTER | 0.808824978 | 5.31733E-12 |
| 32818 | A_68_P24020333 | Rfc5 | chr5:117649732-117649782 | INSIDE | 0.925432051 | 5.5289E-12 |
| 145303 | A_68_P25505005 | Itgal | chr7:127089621-127089680 | INSIDE | 0.750358694 | 5.619E-12 |
| 70203 | A_68_P30506397 | Zfp263 | chr16:003657348-003657407 | PROMOTER | 0.757127434 | 5.70998E-12 |
| 161706 | A_68_P29054786 | Slc34a1 | chr13:055409005-055409056 | PROMOTER | 0.884090485 | 5.85942E-12 |
| 9082 | A_68_P24971933 | Zfp551 | chr7:011328986-011329041 | PROMOTER | 0.916229225 | 5.893E-12 |
| 22488 | A_68_P31462767 | Map3k8 | chr18:004354222-004354281 | PROMOTER | 0.964311651 | 5.92363E-12 |
| 238520 | A_68_P31502469 | Cabyr | chr18:012885102-012885161 | INSIDE | 0.786814436 | 6.01077E-12 |
| 120231 | A_68_P27996752 | Epx | chr11:087692332-087692376 | PROMOTER | 0.761301072 | 6.0352E-12 |
| 126643 | A_68_P29157495 | Ankrd32 | chr13:077553093-077553152 | INSIDE | 1.021385933 | 6.17002E-12 |
| 23135 | A_68_P25372582 | Stard10 | chr7:101191625-101191678 | PROMOTER | 0.763854114 | 6.18667E-12 |
| 27004 | A_68_P21071583 | Dpp7 | chr2:025176379-025176430 | INSIDE | 0.77040455 | 6.31517E-12 |
| 29935 | A_68_P31752314 | Myoz3 | chr18:060720252-060720308 | PROMOTER | 0.799674418 | 6.32517E-12 |
| 78689 | A_68_P25926122 | Klhl2 | chr8:067781874-067781933 | PROMOTER | 0.845946837 | 6.32833E-12 |
| 93657 | A_68_P28326638 | Tmem18 | chr12:031167948-031168001 | PROMOTER | 0.772312167 | 6.36633E-12 |
| 141059 | A_68_P26397727 | Adamts8 | chr9:030687056-030687115 | PROMOTER | 0.75356038 | 6.49933E-12 |
| 148577 | A_68_P29700867 | Slc39a14 | chr14:069088692-069088751 | PROMOTER | 0.891418964 | 6.61645E-12 |
| 69230 | A_68_P31188386 | Gpr110 | chr17:042735047-042735106 | PROMOTER | 1.033828769 | 6.83867E-12 |
| 143687 | A_68_P30784301 | Tomm70a | chr16:057045008-057045067 | INSIDE | 0.861005135 | 6.8685E-12 |
| 83617 | A_68_P27080363 | Rev3l | chr10:039424012-039424071 | INSIDE | 0.865395383 | 6.938E-12 |
| 190 | A_68_P25405539 | Tub | chr7:108799369-108799428 | PROMOTER | 1.023110371 | 7.03567E-12 |
| 92973 | A_68_P26657810 | Gcm1 | chr9:077834475-077834534 | PROMOTER | 0.974930977 | 7.201E-12 |
| 116901 | A_68_P29561447 | Sftpa1 | chr14:040037356-040037411 | PROMOTER | 0.895142558 | 7.3783E-12 |
| 212955 | A_68_P32119277 | Marveld1 | chr19:042199459-042199518 | PROMOTER | 0.773857549 | 7.4539E-12 |
| 206922 | A_68_P24637122 | Hdac11 | chr6:091122559-091122606 | INSIDE | 1.115421078 | 7.56667E-12 |
| 200579 | A_68_P27972090 | Ccl5 | chr11:083348372-083348430 | PROMOTER | 0.836336763 | 7.66646E-12 |
| 162520 | A_68_P27446097 | Csrp2 | chr10:110321605-110321664 | PROMOTER | 0.892640135 | 7.78296E-12 |
| 202442 | A_68_P21761444 | Pxmp4-Zfp341 | chr2:154304623-154304668 | DIVERGENT_PROMOTER | 0.80937783 | 7.9E-12 |
| 115075 | A_68_P22180909 | P2ry1 | chr3:061088109-061088167 | PROMOTER | 0.865295865 | 8.0196E-12 |
| 9885 | A_68_P29600624 | Ear14 | chr14:050126492-050126551 | DOWNSTREAM | 0.760986388 | 8.13475E-12 |
| 124080 | A_68_P31041987 | Park2 | chr17:010688438-010688497 | INSIDE | 0.765031901 | 8.31567E-12 |
| 125846 | A_68_P32470510 | Gab3 | chrX:071338105-071338164 | PROMOTER | 0.860822035 | 8.42123E-12 |
| 14844 | A_68_P27624019 | Meis1 | chr11:018922300-018922359 | PROMOTER | 0.807134998 | 8.76399E-12 |
| 147361 | A_68_P25663111 | Tubgcp3 | chr8:012677398-012677457 | PROMOTER | 1.075427633 | 8.847E-12 |
| 200360 | A_68_P31965285 | Olfr1489 | chr19:013701418-013701477 | DOWNSTREAM | 0.748054374 | 9.18987E-12 |
| 196621 | A_68_P25588215 | Tmem80 | chr7:141185381-141185433 | INSIDE | 0.851515348 | 9.75133E-12 |
| 154449 | A_68_P26906074 | Vip | chr10:004712079-004712138 | PROMOTER | 0.783787178 | 9.7608E-12 |
| 206055 | A_68_P27475770 | Kcnmb4 | chr10:115875990-115876049 | INSIDE | 0.797852712 | 9.77089E-12 |
| 34731 | A_68_P29597142 | Tlr11 | chr14:049280135-049280194 | PROMOTER | 0.815280075 | 9.93423E-12 |
| 179637 | A_68_P32368414 | Cova1 | chrX:045536571-045536630 | PROMOTER | 0.939323216 | 1.02102E-11 |
| 134242 | A_68_P22970060 | AI597013 | chr4:062862986-062863045 | INSIDE | 0.911101168 | 1.03683E-11 |
| 66356 | A_68_P21139936 | Olfr348 | chr2:036605681-036605740 | PROMOTER | 1.01397432 | 1.05533E-11 |
| 153021 | A_68_P26892119 | Zfp105 | chr9:122773774-122773831 | INSIDE | 0.852200216 | 1.07844E-11 |
| 3598 | A_68_P26714877 | Rasgrf1 | chr9:089704753-089704812 | PROMOTER | 0.807016515 | 1.10873E-11 |
| 73562 | A_68_P24617785 | AB041550 | chr6:087692243-087692302 | PROMOTER | 0.9720306 | 1.12533E-11 |
| 20450 | A_68_P23230637 | Tesk2 | chr4:116220557-116220616 | INSIDE | 0.751026698 | 1.1271E-11 |
| 120149 | A_68_P30811326 | Arl13b-Pros1 | chr16:062790869-062790928 | DIVERGENT_PROMOTER | 0.849449376 | 1.14789E-11 |
| 39225 | A_68_P32346688 | Odz1 | chrX:039520349-039520408 | PROMOTER | 0.786452658 | 1.15987E-11 |
| 152310 | A_68_P25094062 | Fut1 | chr7:045487775-045487830 | INSIDE | 0.751340529 | 1.19128E-11 |
| 225831 | A_68_P30986143 | Kcnj15 | chr16:095367923-095367979 | INSIDE | 0.85956046 | 1.19693E-11 |
| 67872 | A_68_P29515529 | Dph3-Oxnad1 | chr14:030914858-030914908 | DIVERGENT_PROMOTER | 0.820846485 | 1.24387E-11 |
| 22882 | A_68_P29302875 | Kif2a | chr13:108141371-108141430 | INSIDE | 0.774306957 | 1.31826E-11 |
| 161499 | A_68_P22905430 | Baat | chr4:049525313-049525372 | INSIDE | 0.963553082 | 1.3288E-11 |
| 6183 | A_68_P21611749 | Ncaph | chr2:126823196-126823255 | INSIDE | 0.787969734 | 1.397E-11 |
| 45314 | A_68_P30578518 | Zdhhc8 | chr16:018151544-018151603 | PROMOTER | 0.919728075 | 1.58755E-11 |
| 92279 | A_68_P25194956 | Lrrc28 | chr7:067518142-067518201 | INSIDE | 0.779154791 | 1.6E-11 |
| 40713 | A_68_P25948068 | Atp13a1 | chr8:072734867-072734918 | INSIDE | 0.915989108 | 1.62833E-11 |
| 186712 | A_68_P32069119 | Tmem23 | chr19:032312099-032312158 | INSIDE | 1.011816439 | 1.6415E-11 |
| 157485 | A_68_P25495197 | Il4ra | chr7:125343763-125343813 | INSIDE | 0.797882542 | 1.65238E-11 |
| 33622 | A_68_P27296949 | Slc41a2 | chr10:082766408-082766467 | INSIDE | 0.844556489 | 1.69636E-11 |
| 102423 | A_68_P28314947 | Tssc1 | chr12:029338889-029338943 | INSIDE | 0.756434422 | 1.71811E-11 |
| 24289 | A_68_P30795161 | Olfr206 | chr16:059286166-059286225 | DOWNSTREAM | 0.789154934 | 1.78807E-11 |
| 201950 | A_68_P24437064 | Dfna5h | chr6:050194324-050194381 | PROMOTER | 0.778055589 | 1.8177E-11 |
| 171589 | A_68_P31056435 | Tcte2 | chr17:013503209-013503267 | PROMOTER | 0.979781703 | 1.83672E-11 |
| 131219 | A_68_P28036772 | Dlx3 | chr11:094936657-094936701 | INSIDE | 0.848633648 | 1.857E-11 |
| 197304 | A_68_P23610092 | Ppp2r2c | chr5:037157630-037157680 | INSIDE | 0.93162411 | 1.89423E-11 |
| 180280 | A_68_P25879797 | Spata4 | chr8:056098759-056098818 | PROMOTER | 0.759665132 | 1.92633E-11 |
| 173739 | A_68_P25414343 | Adm | chr7:110417727-110417778 | PROMOTER | 0.754931073 | 1.93837E-11 |
| 46411 | A_68_P30398214 | Upk3a | chr15:084841177-084841236 | PROMOTER | 0.970840589 | 2.00362E-11 |
| 221236 | A_68_P32131672 | Scd4 | chr19:044385715-044385770 | PROMOTER | 0.901172031 | 2.12717E-11 |
| 193419 | A_68_P24843233 | Clec1b | chr6:129360117-129360176 | PROMOTER | 0.788472375 | 2.15583E-11 |
| 222243 | A_68_P22165646 | Pfn2 | chr3:057934354-057934413 | INSIDE | 0.746436868 | 2.20867E-11 |
| 155503 | A_68_P22332177 | Smcp | chr3:092675571-092675630 | PROMOTER | 0.773312524 | 2.46767E-11 |
| 122508 | A_68_P31627739 | Pcdhb4 | chr18:037431776-037431835 | PROMOTER | 0.846675097 | 2.53067E-11 |
| 44419 | A_68_P24052436 | Anapc5 | chr5:123086729-123086780 | PROMOTER | 0.767228257 | 2.6071E-11 |
| 3911 | A_68_P26473382 | Fxyd2 | chr9:045155440-045155499 | PROMOTER | 0.902966968 | 2.64709E-11 |
| 6611 | A_68_P23152981 | Raver2 | chr4:100568570-100568629 | INSIDE | 1.219145763 | 2.65961E-11 |
| 182580 | A_68_P24895725 | Aebp2 | chr6:140588843-140588902 | INSIDE | 0.795529535 | 2.7334E-11 |
| 115943 | A_68_P28701420 | Serpina5 | chr12:104504422-104504476 | INSIDE | 0.9049196 | 2.83268E-11 |
| 42989 | A_68_P29596532 | Olfr728 | chr14:049066386-049066441 | PROMOTER | 0.778558854 | 2.89589E-11 |
| 23598 | A_68_P22894873 | Alg2 | chr4:047493792-047493848 | INSIDE | 0.790058242 | 2.904E-11 |
| 116906 | A_68_P31945442 | Scgb1a1 | chr19:009155482-009155541 | PROMOTER | 0.760110727 | 2.93527E-11 |
| 120080 | A_68_P28733686 | Rtl1 | chr12:110040112-110040163 | INSIDE | 0.756674221 | 2.97366E-11 |
| 99073 | A_68_P27784480 | Cnot6 | chr11:049559378-049559435 | PROMOTER | 0.790146563 | 2.97808E-11 |
| 181945 | A_68_P26588647 | Rbpms2 | chr9:065422338-065422383 | PROMOTER | 0.991798511 | 3.0806E-11 |
| 80771 | A_68_P29262725 | Ptcd2 | chr13:100441663-100441721 | INSIDE | 0.917036572 | 3.1383E-11 |
| 182 | A_68_P21389120 | Itgav | chr2:083523940-083523999 | PROMOTER | 0.797159056 | 3.14167E-11 |
| 8016 | A_68_P28961834 | Muted | chr13:038641747-038641806 | INSIDE | 0.794010683 | 3.19065E-11 |
| 231788 | A_68_P27687350 | Il9r-3300001G02Rik | chr11:032100644-032100703 | DIVERGENT_PROMOTER | 0.937084605 | 3.3474E-11 |
| 103960 | A_68_P28142769 | Slc39a11 | chr11:113387062-113387121 | PROMOTER | 1.00530627 | 3.45242E-11 |
| 122705 | A_68_P20600066 | Mcm6 | chr1:130186350-130186409 | INSIDE | 0.749475072 | 3.46333E-11 |
| 208597 | A_68_P32268385 | Cask | chrX:013008449-013008503 | PROMOTER | 0.987492239 | 3.47888E-11 |
| 137392 | A_68_P28940983 | Prpf4b | chr13:034879724-034879783 | PROMOTER | 0.818918654 | 3.7495E-11 |
| 3547 | A_68_P25542188 | Bccip | chr7:133547701-133547745 | INSIDE | 0.919117433 | 4.03638E-11 |
| 218599 | A_68_P21608685 | Slc27a2 | chr2:126246751-126246809 | INSIDE | 0.983040146 | 4.04767E-11 |
| 115115 | A_68_P25024689 | Eif3s12 | chr7:028688782-028688841 | INSIDE | 0.757623535 | 4.31367E-11 |
| 200961 | A_68_P24776914 | Olfr211 | chr6:116456092-116456148 | PROMOTER | 0.810573751 | 4.51304E-11 |
| 187893 | A_68_P20266450 | Als2cr13 | chr1:059856408-059856467 | PROMOTER | 0.763490215 | 4.546E-11 |
| 239212 | A_68_P28958218 | Riok1 | chr13:038045719-038045778 | INSIDE | 0.784473804 | 4.64795E-11 |
| 93475 | A_68_P25436589 | Cyp2r1 | chr7:114355883-114355942 | PROMOTER | 0.750021802 | 5.15E-11 |
| 65118 | A_68_P28194523 | Rab10 | chr12:003307541-003307593 | INSIDE | 0.839033454 | 5.18255E-11 |
| 56600 | A_68_P27263178 | Col6a2 | chr10:076069393-076069444 | PROMOTER | 0.756210846 | 5.64368E-11 |
| 133075 | A_68_P28036274 | Pdk2 | chr11:094856036-094856090 | INSIDE | 0.792684795 | 5.65867E-11 |
| 151079 | A_68_P21853394 | Pfdn4 | chr2:170184372-170184431 | PROMOTER | 0.775970227 | 5.68533E-11 |
| 65225 | A_68_P26319139 | Fut4 | chr9:014501650-014501694 | INSIDE | 0.809351586 | 5.94E-11 |
| 87654 | A_68_P20840567 | Olfr432 | chr1:175884140-175884199 | PROMOTER | 0.975125861 | 6.09667E-11 |
| 136102 | A_68_P24925827 | Sspn | chr6:145886633-145886692 | PROMOTER | 0.833619256 | 6.28133E-11 |
| 84910 | A_68_P29510212 | Stab1 | chr14:030000907-030000956 | PROMOTER | 0.891544945 | 6.52002E-11 |
| 188849 | A_68_P28033369 | Mycbpap | chr11:094317429-094317488 | INSIDE | 0.923618079 | 6.621E-11 |
| 223075 | A_68_P23303300 | Laptm5 | chr4:130185086-130185143 | PROMOTER | 1.129602774 | 6.62733E-11 |
| 142171 | A_68_P25313070 | Sytl2 | chr7:090220962-090221021 | PROMOTER | 0.761722216 | 6.67717E-11 |
| 17206 | A_68_P27934863 | Blmh | chr11:076762432-076762487 | INSIDE | 0.746300895 | 6.78305E-11 |
| 170612 | A_68_P24591610 | Znhit4-Rtkn | chr6:083098446-083098505 | DIVERGENT_PROMOTER | 0.793867955 | 6.90533E-11 |
| 52554 | A_68_P30244851 | Derl1 | chr15:057724675-057724734 | PROMOTER | 1.083131456 | 7.2689E-11 |
| 28025 | A_68_P26429032 | Pknox2 | chr9:036898072-036898127 | PROMOTER | 0.799290066 | 7.61119E-11 |
| 91633 | A_68_P30481644 | Accn2 | chr15:099500223-099500269 | INSIDE | 0.748108764 | 7.61207E-11 |
| 51474 | A_68_P27546534 | Olfr798 | chr10:129027377-129027436 | DOWNSTREAM | 0.770449626 | 8.38187E-11 |
| 209323 | A_68_P30295306 | Ndrl | chr15:066802722-066802779 | PROMOTER | 1.13558521 | 8.4298E-11 |
| 199372 | A_68_P29723687 | Esd | chr14:073465947-073465997 | PROMOTER | 0.853682375 | 8.67477E-11 |
| 122291 | A_68_P31161060 | Ier3 | chr17:035424468-035424520 | PROMOTER | 1.024666545 | 8.88033E-11 |
| 90990 | A_68_P21426774 | Phf21a | chr2:091980228-091980287 | PROMOTER | 0.82615527 | 8.94067E-11 |
| 219355 | A_68_P24239844 | Phf14 | chr6:011878053-011878109 | INSIDE | 0.86348166 | 8.96667E-11 |
| 111187 | A_68_P26596770 | Tpm1 | chr9:066850040-066850099 | PROMOTER | 0.773066011 | 9.38233E-11 |
| 170534 | A_68_P27385144 | Wdr51b | chr10:098553438-098553497 | INSIDE | 0.85553164 | 9.65965E-11 |
| 98655 | A_68_P23174723 | C8b | chr4:104263607-104263666 | PROMOTER | 0.857307583 | 9.742E-11 |
| 189512 | A_68_P31201739 | Gm323-Nfkbie | chr17:045013618-045013675 | DIVERGENT_PROMOTER | 0.753013115 | 9.93142E-11 |
| 74637 | A_68_P23242201 | Olfr1337 | chr4:118285951-118286009 | PROMOTER | 0.774972676 | 1.01133E-10 |
| 214821 | A_68_P29848006 | Klf12 | chr14:099032929-099032988 | PROMOTER | 0.798085364 | 1.02066E-10 |
| 129319 | A_68_P32396644 | Tmem32 | chrX:052945579-052945638 | PROMOTER | 0.848158903 | 1.09339E-10 |
| 85074 | A_68_P28556969 | Max | chr12:077883956-077884015 | PROMOTER | 0.770174155 | 1.10126E-10 |
| 124562 | A_68_P30423310 | Hdac10 | chr15:088954969-088955019 | INSIDE | 0.921440271 | 1.20872E-10 |
| 71181 | A_68_P32684303 | Esx1 | chrX:132469053-132469112 | PROMOTER | 0.747658325 | 1.21546E-10 |
| 216350 | A_68_P27956338 | Myo1d | chr11:080596601-080596656 | PROMOTER | 0.851411882 | 1.2628E-10 |
| 185229 | A_68_P20918822 | Ush2a | chr1:189963467-189963526 | PROMOTER | 0.811369686 | 1.27667E-10 |
| 194833 | A_68_P23189542 | Lrrc42 | chr4:106749925-106749984 | INSIDE | 0.749792347 | 1.278E-10 |
| 234325 | A_68_P25100383 | Sergef | chr7:046505736-046505795 | INSIDE | 0.784941256 | 1.29587E-10 |
| 219970 | A_68_P32681974 | Tceal1 | chrX:132053478-132053537 | PROMOTER | 0.76606593 | 1.34182E-10 |
| 55983 | A_68_P30380528 | Polr3h-Csdc2 | chr15:081760260-081760316 | DIVERGENT_PROMOTER | 0.762701247 | 1.34933E-10 |
| 68545 | A_68_P31253491 | Sh3gl1-Chaf1a | chr17:055633435-055633494 | DIVERGENT_PROMOTER | 0.787114602 | 1.37533E-10 |
| 10288 | A_68_P27325804 | Chpt1 | chr10:087932026-087932085 | INSIDE | 0.770970565 | 1.38384E-10 |
| 1957 | A_68_P26593998 | Usp3 | chr9:066391232-066391281 | PROMOTER | 0.866390192 | 1.38442E-10 |
| 76984 | A_68_P22591163 | Gbp5 | chr3:142433399-142433458 | PROMOTER | 1.107383977 | 1.39261E-10 |
| 166045 | A_68_P24215216 | Slc25a13 | chr6:006170247-006170306 | PROMOTER | 0.747904525 | 1.39793E-10 |
| 125940 | A_68_P32745103 | ORF34 | chrX:146683981-146684040 | PROMOTER | 0.886111647 | 1.46737E-10 |
| 82316 | A_68_P29659742 | Neil2 | chr14:062149214-062149271 | PROMOTER | 0.792031757 | 1.49093E-10 |
| 164943 | A_68_P22831610 | Orc3l | chr4:034799159-034799218 | INSIDE | 0.787606777 | 1.51337E-10 |
| 49140 | A_68_P25702256 | Spag11 | chr8:019159098-019159157 | INSIDE | 1.090855248 | 1.52267E-10 |
| 21801 | A_68_P31101415 | BC054438 | chr17:024807060-024807107 | INSIDE | 0.773258147 | 1.53146E-10 |
| 121344 | A_68_P25271583 | Rps17 | chr7:081221968-081222027 | PROMOTER | 0.768428378 | 1.53933E-10 |
| 52629 | A_68_P32670527 | Nox1 | chrX:129457971-129458030 | INSIDE | 1.074680265 | 1.56078E-10 |
| 106179 | A_68_P24402002 | Olfr435 | chr6:043127849-043127907 | PROMOTER | 0.811789693 | 1.5744E-10 |
| 218934 | A_68_P20836774 | Olfr1404 | chr1:175053824-175053883 | DOWNSTREAM | 1.066425317 | 1.60967E-10 |
| 135612 | A_68_P24425112 | AI894139 | chr6:047885330-047885384 | INSIDE | 0.76166895 | 1.64207E-10 |
| 47596 | A_68_P25313850 | Tmem126b | chr7:090353967-090354026 | PROMOTER | 0.829458429 | 1.64633E-10 |
| 102477 | A_68_P21131541 | AI182371 | chr2:034924465-034924524 | PROMOTER | 0.775307906 | 1.65E-10 |
| 97332 | A_68_P31677650 | Ticam2 | chr18:046698591-046698650 | INSIDE | 0.81204658 | 1.85201E-10 |
| 199207 | A_68_P23232396 | Ptch2 | chr4:116594397-116594452 | INSIDE | 1.504617765 | 1.93667E-10 |
| 170306 | A_68_P29510426 | Nisch | chr14:030032926-030032976 | INSIDE | 0.937295657 | 1.99663E-10 |
| 155771 | A_68_P31653319 | Sh3rf2 | chr18:042180373-042180432 | INSIDE | 0.827980682 | 2.02815E-10 |
| 180095 | A_68_P30351113 | Rpl8 | chr15:076733755-076733810 | DOWNSTREAM | 0.875000664 | 2.04061E-10 |
| 59557 | A_68_P30427164 | Syt10 | chr15:089668981-089669040 | INSIDE | 0.915535609 | 2.07328E-10 |
| 178190 | A_68_P31357213 | Rasgrp3 | chr17:075366681-075366740 | PROMOTER | 0.829665951 | 2.1031E-10 |
| 233859 | A_68_P29006981 | Gm1574 | chr13:046286238-046286297 | INSIDE | 0.753052806 | 2.10916E-10 |
| 134519 | A_68_P32027269 | Dock8 | chr19:025119730-025119788 | INSIDE | 0.827592182 | 2.11847E-10 |
| 232219 | A_68_P24890041 | Pik3c2g | chr6:139549798-139549857 | PROMOTER | 0.827120978 | 2.1315E-10 |
| 85441 | A_68_P20168243 | Map4k4 | chr1:039840643-039840702 | PROMOTER | 1.063086299 | 2.20437E-10 |
| 191618 | A_68_P20558170 | Epb4.1l5 | chr1:121474158-121474217 | INSIDE | 0.792555159 | 2.22009E-10 |
| 155399 | A_68_P30061051 | Cdh12 | chr15:021055707-021055766 | PROMOTER | 0.862380471 | 2.22078E-10 |
| 197583 | A_68_P24269364 | St7 | chr6:017698061-017698120 | PROMOTER | 0.880112958 | 2.28343E-10 |
| 135798 | A_68_P20561102 | Steap3 | chr1:122095399-122095455 | PROMOTER | 0.7877515 | 2.30993E-10 |
| 89662 | A_68_P28864269 | Gpr141 | chr13:019829629-019829688 | INSIDE | 0.885678176 | 2.35483E-10 |
| 236310 | A_68_P25962815 | F2rl3 | chr8:075654418-075654477 | PROMOTER | 0.887938743 | 2.35924E-10 |
| 65246 | A_68_P29230934 | Arsb | chr13:094871819-094871878 | PROMOTER | 0.758345885 | 2.35955E-10 |
| 35610 | A_68_P25678409 | BB014433 | chr8:015044855-015044914 | INSIDE | 0.78673821 | 2.39333E-10 |
| 196174 | A_68_P22351759 | Gpr89 | chr3:096992405-096992464 | PROMOTER | 0.848547031 | 2.46628E-10 |
| 89542 | A_68_P30955569 | Sod1 | chr16:090106331-090106390 | PROMOTER | 0.850599441 | 2.5193E-10 |
| 230104 | A_68_P23247336 | Hivep3 | chr4:119308858-119308917 | PROMOTER | 0.875482002 | 2.78867E-10 |
| 28691 | A_68_P32463015 | Atp2b3 | chrX:069750771-069750821 | PROMOTER | 0.890443399 | 2.81039E-10 |
| 84822 | A_68_P29144988 | Ahrr | chr13:074679481-074679540 | INSIDE | 0.805031894 | 2.82344E-10 |
| 180363 | A_68_P26630598 | Ccpg1 | chr9:072778311-072778370 | PROMOTER | 0.937998329 | 2.85044E-10 |
| 123812 | A_68_P28041133 | Abi3 | chr11:095657874-095657933 | INSIDE | 0.834455099 | 2.90402E-10 |
| 238187 | A_68_P29628407 | Il17d | chr14:056477718-056477777 | PROMOTER | 0.864701813 | 3.14932E-10 |
| 27356 | A_68_P25023769 | Ech1 | chr7:028532642-028532699 | PROMOTER | 0.798371073 | 3.23646E-10 |
| 73219 | A_68_P28635707 | Gtf2a1 | chr12:091992978-091993037 | INSIDE | 0.826427358 | 3.35543E-10 |
| 198733 | A_68_P24154032 | Actb | chr5:143176427-143176478 | PROMOTER | 0.904667424 | 3.38251E-10 |
| 229924 | A_68_P28832905 | Ero1lb | chr13:012620305-012620364 | PROMOTER | 0.913557972 | 3.38962E-10 |
| 157897 | A_68_P27452765 | Caps2 | chr10:111565098-111565157 | PROMOTER | 0.789327127 | 3.422E-10 |
| 31083 | A_68_P23645911 | C1qtnf7 | chr5:043803080-043803139 | PROMOTER | 0.785461447 | 3.50533E-10 |
| 198630 | A_68_P21224241 | Arl6ip6 | chr2:053016336-053016395 | INSIDE | 0.839761109 | 3.5172E-10 |
| 43004 | A_68_P27920030 | Olfr411 | chr11:074169013-074169072 | PROMOTER | 0.795173068 | 3.56739E-10 |
| 217070 | A_68_P26521226 | Atm | chr9:053301658-053301717 | PROMOTER | 0.789282474 | 3.67E-10 |
| 41047 | A_68_P32370538 | Olfr1322 | chrX:046130203-046130262 | INSIDE | 1.04816775 | 3.6784E-10 |
| 121341 | A_68_P25366107 | Kcne3 | chr7:100050803-100050854 | PROMOTER | 0.751220515 | 3.692E-10 |
| 235973 | A_68_P20192797 | Ercc5 | chr1:044090203-044090261 | PROMOTER | 0.815626804 | 3.8442E-10 |
| 154123 | A_68_P21071901 | Npdc1 | chr2:025223580-025223632 | PROMOTER | 0.810544275 | 4.00867E-10 |
| 213736 | A_68_P31936735 | Kcnk4 | chr19:006996280-006996339 | INSIDE | 0.859659696 | 4.02038E-10 |
| 44526 | A_68_P24354182 | Slc35b4 | chr6:034106128-034106187 | INSIDE | 0.75513255 | 4.48167E-10 |
| 7469 | A_68_P26898872 | Cnksr3 | chr10:003132479-003132538 | PROMOTER | 0.793176188 | 4.5072E-10 |
| 146412 | A_68_P24117185 | Rhbdd2 | chr5:135931731-135931775 | DOWNSTREAM | 0.973927806 | 4.93394E-10 |
| 219078 | A_68_P28883030 | Hfe | chr13:023718288-023718334 | PROMOTER | 0.869457016 | 5.06958E-10 |
| 50996 | A_68_P29904236 | Slitrk5 | chr14:110553714-110553773 | PROMOTER | 0.819239485 | 5.11664E-10 |
| 186759 | A_68_P32669429 | Tspan6 | chrX:129244560-129244619 | INSIDE | 0.893169629 | 5.4113E-10 |
| 47309 | A_68_P30678369 | Fbxo40 | chr16:036899833-036899888 | PROMOTER | 0.828973861 | 5.43434E-10 |
| 56447 | A_68_P26167402 | Glg1 | chr8:114149366-114149424 | PROMOTER | 0.82854321 | 5.53767E-10 |
| 47215 | A_68_P30092462 | Ank | chr15:027410871-027410918 | PROMOTER | 0.754138681 | 5.57644E-10 |
| 34532 | A_68_P24990779 | P42pop | chr7:018149386-018149442 | PROMOTER | 1.068592302 | 5.60597E-10 |
| 149187 | A_68_P30137018 | Polr2k | chr15:036109074-036109132 | PROMOTER | 0.851722036 | 5.61805E-10 |
| 165176 | A_68_P22729525 | Rbm35a | chr4:011311267-011311326 | INSIDE | 0.999477383 | 5.67113E-10 |
| 142197 | A_68_P22388934 | Rsbn1 | chr3:104094709-104094768 | INSIDE | 1.011571436 | 5.781E-10 |
| 153647 | A_68_P30142993 | Grhl2 | chr15:037179560-037179616 | INSIDE | 1.12617968 | 6.01841E-10 |
| 173925 | A_68_P28814390 | Larp5 | chr13:009091678-009091737 | PROMOTER | 0.925938947 | 6.15595E-10 |
| 23020 | A_68_P27898191 | Cldn7 | chr11:069779951-069780010 | PROMOTER | 0.772612498 | 6.38662E-10 |
| 232722 | A_68_P29941868 | Dnajc3 | chr14:118086950-118087008 | INSIDE | 0.760976169 | 7.0399E-10 |
| 24605 | A_68_P32090663 | Hectd2 | chr19:036615190-036615243 | PROMOTER | 0.858747468 | 7.40254E-10 |
| 47642 | A_68_P30183883 | Kcnv1 | chr15:044942416-044942475 | INSIDE | 0.807052549 | 7.64258E-10 |
| 152537 | A_68_P32200563 | Nrap-Casp7 | chr19:056449119-056449178 | DIVERGENT_PROMOTER | 0.865889385 | 7.89613E-10 |
| 189455 | A_68_P21001424 | Sfmbt2 | chr2:010427288-010427347 | INSIDE | 1.026600159 | 7.93358E-10 |
| 29157 | A_68_P26351660 | Anln | chr9:022140664-022140723 | PROMOTER | 0.843848672 | 8.29267E-10 |
| 40222 | A_68_P28122582 | Abca9 | chr11:109982548-109982607 | INSIDE | 0.8320728 | 8.66033E-10 |
| 52805 | A_68_P31678579 | Ap3s1 | chr18:046870720-046870779 | INSIDE | 1.457028759 | 8.77057E-10 |
| 190450 | A_68_P27453278 | Kcnc2 | chr10:111670894-111670953 | PROMOTER | 1.034728099 | 9.06807E-10 |
| 141890 | A_68_P21410135 | Olfr1186 | chr2:088323196-088323255 | PROMOTER | 0.838862492 | 9.11333E-10 |
| 72407 | A_68_P31108423 | Tmem8 | chr17:025848884-025848931 | INSIDE | 0.774364588 | 9.4373E-10 |
| 149813 | A_68_P26497843 | Ttc12 | chr9:049242476-049242535 | PROMOTER | 0.941613265 | 1.03918E-09 |
| 60104 | A_68_P30250688 | Rnf139 | chr15:058715235-058715294 | PROMOTER | 0.751608733 | 1.06083E-09 |
| 7485 | A_68_P28184012 | Actg1-Fscn2 | chr11:120172538-120172587 | DIVERGENT_PROMOTER | 0.790219095 | 1.08393E-09 |
| 176323 | A_68_P20230123 | Gdf8 | chr1:053003253-053003312 | PROMOTER | 0.866477865 | 1.10117E-09 |
| 26588 | A_68_P29600019 | Rnase4 | chr14:050012001-050012060 | PROMOTER | 0.811660729 | 1.13008E-09 |
| 231072 | A_68_P29139996 | Aytl2 | chr13:073932915-073932974 | PROMOTER | 0.806405295 | 1.14534E-09 |
| 173584 | A_68_P24863742 | Crebl2 | chr6:134790487-134790546 | PROMOTER | 0.920353607 | 1.19847E-09 |
| 228229 | A_68_P29952546 | Ranbp5 | chr14:120042356-120042415 | PROMOTER | 0.830753672 | 1.25657E-09 |
| 226310 | A_68_P23966246 | Tgfbr3 | chr5:107533718-107533777 | PROMOTER | 0.842793361 | 1.26297E-09 |
| 28637 | A_68_P25047334 | Cebpg | chr7:034768041-034768100 | PROMOTER | 0.811421631 | 1.27233E-09 |
| 99397 | A_68_P28118812 | Slc16a6 | chr11:109292167-109292226 | PROMOTER | 0.760535022 | 1.27543E-09 |
| 83603 | A_68_P24389171 | Tas2r137 | chr6:040417475-040417534 | PROMOTER | 0.785143969 | 1.28667E-09 |
| 13205 | A_68_P24398463 | Olfr458 | chr6:042389334-042389393 | DOWNSTREAM | 0.756702137 | 1.39923E-09 |
| 103416 | A_68_P20457745 | Neu4 | chr1:095846563-095846622 | PROMOTER | 0.773500265 | 1.41267E-09 |
| 206702 | A_68_P31208525 | Pex6 | chr17:046175679-046175738 | INSIDE | 0.847215478 | 1.44925E-09 |
| 169240 | A_68_P26268794 | Nrp1 | chr8:131246622-131246681 | INSIDE | 0.767991431 | 1.54133E-09 |
| 17450 | A_68_P22882291 | Exosc3 | chr4:045342435-045342487 | PROMOTER | 0.889607154 | 1.546E-09 |
| 80010 | A_68_P20353320 | Dnpep | chr1:075201421-075201480 | PROMOTER | 0.806084825 | 1.548E-09 |
| 227058 | A_68_P23474527 | Grm3 | chr5:009731730-009731782 | PROMOTER | 0.808516436 | 1.56182E-09 |
| 236594 | A_68_P24275508 | Lsm8 | chr6:018799232-018799291 | INSIDE | 0.753699868 | 1.577E-09 |
| 117192 | A_68_P29650866 | Trim13 | chr14:060551997-060552056 | PROMOTER | 0.809872528 | 1.68073E-09 |
| 214631 | A_68_P22399848 | Adora3 | chr3:106029888-106029947 | PROMOTER | 0.75421824 | 1.71777E-09 |
| 81014 | A_68_P26742988 | Pcolce2 | chr9:095449188-095449247 | INSIDE | 0.890627941 | 1.73332E-09 |
| 16689 | A_68_P23973586 | Fgfrl1 | chr5:108931300-108931359 | PROMOTER | 1.054153043 | 1.84067E-09 |
| 62322 | A_68_P30742841 | Retnlg | chr16:048788521-048788580 | PROMOTER | 1.100098579 | 1.84613E-09 |
| 239445 | A_68_P22409632 | Gstm6 | chr3:108069802-108069857 | INSIDE | 0.805849301 | 1.9239E-09 |
| 38298 | A_68_P22552026 | Zcd2-Ube2d3 | chr3:135370705-135370764 | DIVERGENT_PROMOTER | 0.762665455 | 1.94633E-09 |
| 194291 | A_68_P20442001 | BC056923 | chr1:093195856-093195908 | PROMOTER | 0.826307841 | 1.9812E-09 |
| 3834 | A_68_P23155614 | Dnajc6 | chr4:101048855-101048909 | INSIDE | 0.95982997 | 2.02394E-09 |
| 206112 | A_68_P29875601 | Rbm26 | chr14:104061614-104061673 | INSIDE | 0.939848701 | 2.11563E-09 |
| 171332 | A_68_P25810925 | Frg1 | chr8:042916349-042916408 | PROMOTER | 0.994981919 | 2.30833E-09 |
| 109341 | A_68_P30486147 | BC035295 | chr15:100323627-100323686 | PROMOTER | 0.834138681 | 2.33037E-09 |
| 10951 | A_68_P25836695 | Mlf1ip | chr8:048050504-048050556 | PROMOTER | 0.757000694 | 2.34167E-09 |
| 122958 | A_68_P28883981 | Slc17a1 | chr13:023877732-023877789 | PROMOTER | 0.870986611 | 2.39413E-09 |
| 198960 | A_68_P22422601 | Ntng1 | chr3:110274803-110274862 | PROMOTER | 0.96458016 | 2.50357E-09 |
| 30927 | A_68_P31845724 | Pias2 | chr18:077270407-077270461 | INSIDE | 0.904785812 | 2.65833E-09 |
| 164544 | A_68_P28901883 | Dtprp | chr13:027350245-027350304 | PROMOTER | 0.767584477 | 2.81534E-09 |
| 164333 | A_68_P27891974 | Rpl26 | chr11:068712707-068712766 | PROMOTER | 1.078740015 | 2.8334E-09 |
| 117538 | A_68_P20849164 | Fh1-Kmo | chr1:177464663-177464721 | DIVERGENT_PROMOTER | 1.085581395 | 3.10001E-09 |
| 86160 | A_68_P28615066 | Ahsa1 | chr12:088156219-088156263 | INSIDE | 0.864633166 | 3.12038E-09 |
| 45286 | A_68_P20411402 | B3gnt7 | chr1:088129409-088129468 | PROMOTER | 0.890054011 | 3.22558E-09 |
| 25582 | A_68_P21127578 | Mapkap1 | chr2:034252161-034252220 | PROMOTER | 0.968256212 | 3.41097E-09 |
| 99490 | A_68_P32182234 | Smndc1 | chr19:053441788-053441847 | INSIDE | 0.923680352 | 3.51043E-09 |
| 34833 | A_68_P20820937 | Uap1 | chr1:172013153-172013203 | PROMOTER | 0.925660179 | 3.63469E-09 |
| 239027 | A_68_P32039556 | Vldlr | chr19:027279538-027279595 | PROMOTER | 0.884704014 | 4.1738E-09 |
| 143002 | A_68_P32675855 | Tceal6 | chrX:130557491-130557550 | PROMOTER | 0.863372549 | 4.44743E-09 |
| 232868 | A_68_P24168919 | Gpr12 | chr5:146897151-146897204 | PROMOTER | 0.779816707 | 4.622E-09 |
| 34403 | A_68_P28469877 | Sip1 | chr12:059932989-059933048 | INSIDE | 0.750336779 | 5.06585E-09 |
| 119024 | A_68_P30363976 | Galr3 | chr15:078866415-078866459 | PROMOTER | 0.871634881 | 5.06755E-09 |
| 140472 | A_68_P20439610 | Rab17 | chr1:092803619-092803674 | PROMOTER | 0.811761095 | 5.08747E-09 |
| 8548 | A_68_P26467012 | Tmem24-Dpagt1 | chr9:044076939-044076988 | DIVERGENT_PROMOTER | 0.802168977 | 5.133E-09 |
| 198818 | A_68_P27965950 | Tmem132e | chr11:082207339-082207398 | INSIDE | 0.916693323 | 5.17033E-09 |
| 40943 | A_68_P24265227 | Tes | chr6:017013853-017013912 | PROMOTER | 0.937751108 | 5.4958E-09 |
| 187641 | A_68_P22152023 | Sohlh2 | chr3:055271269-055271328 | INSIDE | 0.75581934 | 5.56909E-09 |
| 110133 | A_68_P25511154 | Bag3 | chr7:128313809-128313868 | PROMOTER | 1.026754385 | 5.578E-09 |
| 167292 | A_68_P31351698 | Yipf4 | chr17:074396731-074396790 | INSIDE | 0.748931766 | 5.59289E-09 |
| 101295 | A_68_P24811546 | Clec4a2 | chr6:123090797-123090856 | INSIDE | 0.834034793 | 6.07933E-09 |
| 202948 | A_68_P26476656 | Pcsk7 | chr9:045656561-045656620 | PROMOTER | 1.014860269 | 6.10003E-09 |
| 88725 | A_68_P23176942 | Ppap2b | chr4:104650334-104650390 | PROMOTER | 0.754737685 | 6.19167E-09 |
| 35361 | A_68_P23541903 | Tmub1 | chr5:023956467-023956526 | INSIDE | 0.748874472 | 6.377E-09 |
| 137295 | A_68_P25798758 | Tusc3 | chr8:040476170-040476229 | INSIDE | 0.761241122 | 6.529E-09 |
| 183795 | A_68_P31193422 | Clic5 | chr17:043647636-043647695 | PROMOTER | 1.132441889 | 7.9118E-09 |
| 85655 | A_68_P27558553 | Mtmr3 | chr11:004496704-004496759 | PROMOTER | 0.895646959 | 8.24946E-09 |
| 77156 | A_68_P31676059 | Pggt1b | chr18:046406760-046406819 | PROMOTER | 1.180690302 | 8.53761E-09 |
| 139641 | A_68_P23523147 | Phtf2 | chr5:020392932-020392991 | INSIDE | 0.84240657 | 8.56834E-09 |
| 77780 | A_68_P31210120 | Ubr2 | chr17:046473062-046473121 | INSIDE | 0.788845576 | 8.61617E-09 |
| 201428 | A_68_P26478709 | Apoc3-Apoa4 | chr9:045988283-045988342 | DIVERGENT_PROMOTER | 1.030107513 | 8.70456E-09 |
| 112642 | A_68_P29420245 | Olfr31 | chr14:013120865-013120924 | PROMOTER | 0.895660065 | 8.81E-09 |
| 94782 | A_68_P31103121 | Tpsab1 | chr17:025071827-025071885 | INSIDE | 1.373513691 | 9.16667E-09 |
| 76092 | A_68_P32319264 | Pgrmc1 | chrX:033029081-033029136 | PROMOTER | 0.870005026 | 9.1847E-09 |
| 37134 | A_68_P24613535 | Gfpt1 | chr6:087009830-087009889 | INSIDE | 0.825026125 | 9.42317E-09 |
| 216881 | A_68_P31933633 | Ehd1 | chr19:006276617-006276665 | PROMOTER | 0.857649597 | 9.49367E-09 |
| 190037 | A_68_P27893929 | Gucy2e | chr11:069057647-069057706 | PROMOTER | 0.820700315 | 9.68833E-09 |
| 219110 | A_68_P28593199 | Wdr21 | chr12:084414330-084414383 | PROMOTER | 0.808959316 | 9.818E-09 |
| 142413 | A_68_P22460920 | Dpyd | chr3:118550548-118550607 | PROMOTER | 1.111157909 | 9.98697E-09 |
| 84868 | A_68_P22373295 | Ptgfrn | chr3:101242520-101242579 | PROMOTER | 0.796239962 | 1.02667E-08 |
| 104078 | A_68_P23796139 | Fryl | chr5:073449385-073449443 | PROMOTER | 1.239105063 | 1.04111E-08 |
| 30781 | A_68_P20449366 | Ndufa10 | chr1:094307998-094308057 | PROMOTER | 0.869036014 | 1.06567E-08 |
| 20891 | A_68_P21221455 | Stam2 | chr2:052567580-052567639 | PROMOTER | 0.850765527 | 1.06727E-08 |
| 240056 | A_68_P20946950 | Syt14 | chr1:194741452-194741511 | PROMOTER | 1.155434943 | 1.07679E-08 |
| 189328 | A_68_P31102463 | Gnptg | chr17:024963525-024963577 | INSIDE | 0.846818133 | 1.14334E-08 |
| 39486 | A_68_P21332459 | Chrna1 | chr2:073381334-073381393 | PROMOTER | 0.781357063 | 1.23246E-08 |
| 189507 | A_68_P32176240 | Ins1 | chr19:052316112-052316171 | PROMOTER | 0.892531176 | 1.32793E-08 |
| 195637 | A_68_P20036894 | Sulf1 | chr1:012698664-012698723 | PROMOTER | 0.760541379 | 1.3919E-08 |
| 19216 | A_68_P28063006 | Krt40 | chr11:099358640-099358699 | INSIDE | 0.766408563 | 1.53314E-08 |
| 151614 | A_68_P23351071 | Akr7a5 | chr4:138587421-138587480 | INSIDE | 0.788636113 | 1.637E-08 |
| 239434 | A_68_P31932529 | Tm7sf2 | chr19:006070412-006070462 | PROMOTER | 0.985594168 | 1.76001E-08 |
| 51922 | A_68_P28301685 | Rnf144 | chr12:027001965-027002024 | PROMOTER | 1.026920115 | 1.79334E-08 |
| 92870 | A_68_P29020463 | AW456874 | chr13:048636193-048636247 | INSIDE | 0.78803277 | 1.86387E-08 |
| 140205 | A_68_P25272398 | BC048679 | chr7:081370920-081370967 | INSIDE | 0.837768714 | 1.87253E-08 |
| 39725 | A_68_P29596696 | Olfr730 | chr14:049108998-049109056 | INSIDE | 0.791411379 | 2.01567E-08 |
| 148482 | A_68_P26398432 | Zbtb44 | chr9:030817938-030817997 | INSIDE | 0.946540834 | 2.32527E-08 |
| 46653 | A_68_P32458868 | Gabra3 | chrX:068912214-068912273 | PROMOTER | 0.749964637 | 2.34067E-08 |
| 12804 | A_68_P24942886 | Tera | chr6:148901595-148901650 | INSIDE | 0.918360582 | 2.35767E-08 |
| 88616 | A_68_P25829983 | BC035537 | chr8:046878817-046878876 | INSIDE | 0.76679738 | 2.50767E-08 |
| 69954 | A_68_P26816159 | Prkar2a | chr9:108547826-108547885 | PROMOTER | 0.806120828 | 2.5886E-08 |
| 139194 | A_68_P21040949 | BC061194 | chr2:018614408-018614467 | PROMOTER | 1.016519438 | 2.6395E-08 |
| 118329 | A_68_P31781195 | Malt1 | chr18:065551065-065551124 | PROMOTER | 0.819637698 | 2.67924E-08 |
| 130097 | A_68_P20186975 | Tgfbrap1 | chr1:043041633-043041692 | INSIDE | 0.758554416 | 2.79869E-08 |
| 219535 | A_68_P25591639 | Tollip | chr7:141714095-141714154 | PROMOTER | 0.753027557 | 2.93333E-08 |
| 33726 | A_68_P23868407 | Sult1d1 | chr5:088641064-088641123 | INSIDE | 1.008656644 | 2.97352E-08 |
| 181842 | A_68_P24222137 | Asns | chr6:007643599-007643658 | PROMOTER | 0.908445262 | 3.02334E-08 |
| 189 | A_68_P31168471 | Olfr105 | chr17:036989033-036989089 | PROMOTER | 0.783537374 | 3.10277E-08 |
| 134463 | A_68_P31083691 | V1re6 | chr17:020434819-020434878 | PROMOTER | 1.051917449 | 4.05246E-08 |
| 173330 | A_68_P28963692 | Slc35b3 | chr13:038967235-038967294 | INSIDE | 0.870814003 | 4.08181E-08 |
| 82326 | A_68_P26013524 | Tbc1d9 | chr8:086056738-086056797 | INSIDE | 0.998292447 | 4.19433E-08 |
| 203906 | A_68_P31755234 | Cdx1-Pdgfrb | chr18:061165403-061165457 | DIVERGENT_PROMOTER | 1.055231531 | 4.22667E-08 |
| 46044 | A_68_P25095748 | Tmem143 | chr7:045769072-045769131 | INSIDE | 0.917547558 | 4.27823E-08 |
| 43709 | A_68_P20830822 | Ncstn | chr1:173915783-173915842 | INSIDE | 0.994803606 | 4.282E-08 |
| 3905 | A_68_P23226789 | Dmbx1 | chr4:115435778-115435835 | INSIDE | 1.177992896 | 4.4498E-08 |
| 142829 | A_68_P32710921 | Ammecr1 | chrX:138217501-138217560 | PROMOTER | 1.014096773 | 4.83677E-08 |
| 112503 | A_68_P32682227 | BC065397 | chrX:132092214-132092273 | DOWNSTREAM | 0.79922561 | 4.87326E-08 |
| 119440 | A_68_P25542022 | Mmp21 | chr7:133520472-133520531 | PROMOTER | 0.786711374 | 5.02333E-08 |
| 51073 | A_68_P24467538 | Crhr2 | chr6:055067185-055067234 | PROMOTER | 0.907001896 | 5.11131E-08 |
| 109422 | A_68_P24840150 | Klrb1a | chr6:128593352-128593411 | PROMOTER | 0.914850149 | 5.13655E-08 |
| 88594 | A_68_P23059258 | Zdhhc21 | chr4:082335098-082335157 | PROMOTER | 0.913643877 | 5.38867E-08 |
| 78562 | A_68_P31920106 | Aldh3b1 | chr19:003928894-003928938 | INSIDE | 0.878307349 | 5.7E-08 |
| 6611 | A_68_P28555613 | Spnb1 | chr12:077627128-077627184 | INSIDE | 0.869300417 | 5.90131E-08 |
| 198192 | A_68_P22331237 | Sprr2i | chr3:092488545-092488604 | PROMOTER | 0.859227216 | 5.97343E-08 |
| 239822 | A_68_P27708850 | Rars | chr11:035676472-035676531 | INSIDE | 0.829550999 | 6.00533E-08 |
| 92663 | A_68_P21103126 | Ppp2r4 | chr2:030241622-030241681 | INSIDE | 0.838122222 | 6.0879E-08 |
| 134698 | A_68_P26700790 | Mod1 | chr9:086494112-086494171 | PROMOTER | 1.097527605 | 6.18461E-08 |
| 51933 | A_68_P25389540 | Dub1a | chr7:104713845-104713904 | DOWNSTREAM | 0.808722575 | 6.22627E-08 |
| 99391 | A_68_P23803981 | Fip1l1 | chr5:074818866-074818925 | INSIDE | 0.842656765 | 6.46334E-08 |
| 215295 | A_68_P21116128 | Dpm2 | chr2:032393979-032394038 | INSIDE | 0.849097229 | 6.76689E-08 |
| 154432 | A_68_P25025805 | Psmd8 | chr7:028889428-028889474 | PROMOTER | 0.883949061 | 6.79773E-08 |
| 41963 | A_68_P29025857 | Aspn | chr13:049554812-049554871 | PROMOTER | 0.999819888 | 7.04908E-08 |
| 237632 | A_68_P22972938 | Tnfsf8 | chr4:063349574-063349633 | PROMOTER | 0.850884427 | 7.28617E-08 |
| 6492 | A_68_P20935088 | Rps6kc1 | chr1:192611477-192611536 | INSIDE | 0.803371363 | 7.47333E-08 |
| 187385 | A_68_P25260035 | Abhd2 | chr7:079143810-079143859 | PROMOTER | 0.793214782 | 7.60322E-08 |
| 222393 | A_68_P29503295 | Chdh | chr14:028848045-028848100 | PROMOTER | 0.757353603 | 7.63333E-08 |
| 148461 | A_68_P31964162 | Olfr1466 | chr19:013407196-013407255 | PROMOTER | 0.801361243 | 7.98443E-08 |
| 18491 | A_68_P29215852 | Rps23 | chr13:091399386-091399445 | DOWNSTREAM | 0.84084841 | 8.38233E-08 |
| 113039 | A_68_P27469172 | Tmem19 | chr10:114769173-114769230 | PROMOTER | 0.786277797 | 8.433E-08 |
| 50748 | A_68_P25842955 | Ing2 | chr8:049174693-049174742 | PROMOTER | 0.768722999 | 8.71333E-08 |
| 109244 | A_68_P25471471 | Cdr2 | chr7:120775129-120775188 | PROMOTER | 1.074425199 | 9.1241E-08 |
| 205988 | A_68_P25259331 | Hapln3 | chr7:079006123-079006169 | PROMOTER | 0.82617335 | 9.20069E-08 |
| 147114 | A_68_P20421164 | Usp40 | chr1:089837313-089837372 | INSIDE | 0.752203062 | 9.48133E-08 |
| 1220 | A_68_P24398556 | Olfr457 | chr6:042402205-042402264 | PROMOTER | 0.777774658 | 9.68333E-08 |
| 185869 | A_68_P27010251 | Enpp1 | chr10:024403681-024403740 | PROMOTER | 1.031106093 | 9.70443E-08 |
| 127275 | A_68_P20240121 | Coq10b | chr1:054992792-054992851 | PROMOTER | 0.774288121 | 9.75133E-08 |
| 77817 | A_68_P32559465 | Dgat2l4 | chrX:096620034-096620090 | PROMOTER | 0.771031637 | 9.93485E-08 |
| 166359 | A_68_P30780909 | Abi3bp | chr16:056398181-056398239 | PROMOTER | 0.764031144 | 1.01774E-07 |
| 93619 | A_68_P23470073 | Abcb4 | chr5:008899514-008899567 | PROMOTER | 0.766545806 | 1.0193E-07 |
| 95430 | A_68_P26432409 | Olfr875 | chr9:037521888-037521947 | PROMOTER | 0.863073015 | 1.04605E-07 |
| 77044 | A_68_P28167172 | Tmc6 | chr11:117596676-117596731 | PROMOTER | 0.966250595 | 1.09E-07 |
| 202954 | A_68_P31382207 | Dhx57 | chr17:080200018-080200074 | PROMOTER | 0.761973757 | 1.09065E-07 |
| 82089 | A_68_P32365828 | Gpr119 | chrX:044917004-044917063 | INSIDE | 0.764572409 | 1.12006E-07 |
| 160790 | A_68_P28697291 | Otub2 | chr12:103791498-103791551 | INSIDE | 1.024885416 | 1.13667E-07 |
| 68000 | A_68_P27996141 | Bzrap1 | chr11:087593233-087593283 | PROMOTER | 0.986584865 | 1.16336E-07 |
| 14971 | A_68_P23883547 | Afm | chr5:091593986-091594045 | PROMOTER | 0.769986304 | 1.17016E-07 |
| 63603 | A_68_P24875247 | Arhgdib | chr6:136889130-136889189 | INSIDE | 0.822285031 | 1.18467E-07 |
| 98145 | A_68_P29599129 | Osgep | chr14:049841522-049841581 | INSIDE | 0.809333769 | 1.21854E-07 |
| 179320 | A_68_P21042859 | Pip5k2a | chr2:018914195-018914254 | INSIDE | 0.764005218 | 1.245E-07 |
| 147594 | A_68_P29937497 | Sox21 | chr14:117121498-117121557 | PROMOTER | 0.825366788 | 1.25018E-07 |
| 40967 | A_68_P20956480 | Plxna2 | chr1:196321822-196321881 | INSIDE | 0.812438149 | 1.313E-07 |
| 68304 | A_68_P23297736 | Txlna | chr4:129141056-129141111 | INSIDE | 0.888574238 | 1.33333E-07 |
| 164160 | A_68_P22788550 | Gpr63 | chr4:025065291-025065347 | INSIDE | 0.968311591 | 1.33467E-07 |
| 143476 | A_68_P23869637 | Csn1s2b | chr5:088885145-088885204 | INSIDE | 0.886785723 | 1.34133E-07 |
| 158118 | A_68_P28901677 | Prlpb | chr13:027317440-027317499 | PROMOTER | 0.769269846 | 1.41697E-07 |
| 98090 | A_68_P24569766 | Pap | chr6:078296381-078296440 | PROMOTER | 0.772267527 | 1.46127E-07 |
| 17624 | A_68_P30486842 | BC004728 | chr15:100466109-100466156 | PROMOTER | 0.932620934 | 1.4653E-07 |
| 84117 | A_68_P31167790 | Olfr94 | chr17:036805572-036805617 | INSIDE | 1.029515638 | 1.50223E-07 |
| 180334 | A_68_P28581719 | Med6 | chr12:082517723-082517782 | PROMOTER | 0.921816238 | 1.52347E-07 |
| 30291 | A_68_P27782292 | Olfr1393 | chr11:049121323-049121382 | PROMOTER | 0.839992609 | 1.58991E-07 |
| 108637 | A_68_P23854781 | Epha5 | chr5:085490022-085490074 | INSIDE | 0.844766383 | 1.72047E-07 |
| 198991 | A_68_P26248368 | Exoc8 | chr8:127788884-127788938 | PROMOTER | 0.77884853 | 1.77883E-07 |
| 67889 | A_68_P30625126 | Uts2d | chr16:027287993-027288050 | PROMOTER | 0.890543525 | 1.88667E-07 |
| 226624 | A_68_P24903957 | BC027061 | chr6:142304018-142304077 | INSIDE | 0.749080684 | 1.93753E-07 |
| 181296 | A_68_P21575392 | Capn3 | chr2:120153332-120153391 | PROMOTER | 0.78478239 | 2.03067E-07 |
| 10847 | A_68_P21579933 | Trp53bp1 | chr2:120968232-120968284 | PROMOTER | 0.756019986 | 2.044E-07 |
| 45860 | A_68_P30721010 | Ccdc52 | chr16:044262447-044262506 | PROMOTER | 0.77529861 | 2.13005E-07 |
| 7721 | A_68_P24538869 | Ptcd3 | chr6:071834868-071834927 | INSIDE | 0.761026687 | 2.2048E-07 |
| 234622 | A_68_P26587463 | Rasl12 | chr9:065195743-065195801 | PROMOTER | 0.796700837 | 2.21538E-07 |
| 169303 | A_68_P26492125 | Rbm7 | chr9:048245497-048245556 | INSIDE | 0.827429719 | 2.3845E-07 |
| 223670 | A_68_P30008960 | Prlr | chr15:010120344-010120393 | PROMOTER | 0.788740015 | 2.42062E-07 |
| 94662 | A_68_P21866734 | Bmp7 | chr2:172586477-172586535 | PROMOTER | 0.931682249 | 2.42773E-07 |
| 51704 | A_68_P29618391 | Pck2 | chr14:054496126-054496185 | INSIDE | 0.830296652 | 2.4291E-07 |
| 179470 | A_68_P21633720 | Cenpb-Cdc25b | chr2:130876336-130876394 | DIVERGENT_PROMOTER | 0.753956378 | 2.43833E-07 |
| 89848 | A_68_P20348191 | Slc11a1 | chr1:074306191-074306244 | PROMOTER | 1.001712972 | 2.44667E-07 |
| 114778 | A_68_P27882252 | Myh8 | chr11:067094255-067094306 | INSIDE | 1.083804095 | 2.49744E-07 |
| 22856 | A_68_P20597377 | Rab3gap1 | chr1:129691532-129691591 | PROMOTER | 0.76197706 | 2.6033E-07 |
| 202330 | A_68_P31229029 | Plcl2 | chr17:049975837-049975896 | INSIDE | 0.811854232 | 2.77217E-07 |
| 135407 | A_68_P24063303 | Ccdc92 | chr5:125155599-125155658 | PROMOTER | 0.7983977 | 2.835E-07 |
| 196948 | A_68_P26288306 | Mmp8 | chr9:007553851-007553910 | PROMOTER | 0.805442879 | 2.97027E-07 |
| 24090 | A_68_P24817079 | Tpi1 | chr6:124777239-124777298 | INSIDE | 1.050269445 | 3.0775E-07 |
| 56192 | A_68_P28190127 | Rab40b | chr11:121174843-121174902 | INSIDE | 0.868155506 | 3.16937E-07 |
| 20917 | A_68_P22193742 | Plch1 | chr3:063944034-063944091 | PROMOTER | 1.028339037 | 3.51477E-07 |
| 164925 | A_68_P23278135 | Lsm10 | chr4:125597292-125597348 | PROMOTER | 0.896731901 | 3.6122E-07 |
| 19658 | A_68_P24402720 | Nobox | chr6:043238369-043238428 | INSIDE | 0.808594935 | 3.92053E-07 |
| 7576 | A_68_P32680332 | Ngfrap1 | chrX:131612785-131612844 | PROMOTER | 0.757350019 | 4.33333E-07 |
| 163354 | A_68_P30685167 | Nr1i2 | chr16:038212513-038212571 | INSIDE | 0.877182812 | 4.40488E-07 |
| 204985 | A_68_P21266383 | Tbr1 | chr2:061606204-061606258 | INSIDE | 0.908705024 | 4.50567E-07 |
| 107450 | A_68_P28397159 | Pnpla8 | chr12:045140613-045140672 | INSIDE | 0.887267757 | 4.58009E-07 |
| 143303 | A_68_P27898533 | Acadvl-Dlgh4 | chr11:069831762-069831821 | DIVERGENT_PROMOTER | 0.772159963 | 4.61595E-07 |
| 12620 | A_68_P30503171 | Copz1 | chr15:103102271-103102330 | INSIDE | 0.759961048 | 5.16803E-07 |
| 37178 | A_68_P20829392 | Slamf1 | chr1:173605468-173605527 | INSIDE | 1.218960497 | 5.47819E-07 |
| 19805 | A_68_P27614837 | AI553587 | chr11:017113286-017113345 | INSIDE | 0.746395092 | 5.50059E-07 |
| 179912 | A_68_P22317909 | Thbs3 | chr3:089302127-089302186 | INSIDE | 0.874437049 | 5.67477E-07 |
| 157212 | A_68_P26527478 | Wdr61 | chr9:054537125-054537174 | PROMOTER | 1.179250634 | 5.83333E-07 |
| 237031 | A_68_P23223370 | Pdzk1ip1 | chr4:114587715-114587774 | INSIDE | 1.018323409 | 6.67633E-07 |
| 23149 | A_68_P25837102 | Casp3 | chr8:048120723-048120779 | INSIDE | 0.794194048 | 6.71033E-07 |
| 235193 | A_68_P28149087 | Ttyh2 | chr11:114490598-114490647 | PROMOTER | 0.833968218 | 6.8076E-07 |
| 31066 | A_68_P24854492 | Tas2r124 | chr6:132718015-132718074 | PROMOTER | 0.75599614 | 6.8661E-07 |
| 100708 | A_68_P31375048 | Prkcn | chr17:078923185-078923244 | PROMOTER | 1.11370821 | 6.91629E-07 |
| 211907 | A_68_P30672914 | Dtx3l | chr16:035860889-035860948 | PROMOTER | 0.894842637 | 6.93139E-07 |
| 217373 | A_68_P31734250 | Megf10 | chr18:057260159-057260211 | INSIDE | 1.00839702 | 7.00703E-07 |
| 5507 | A_68_P23567490 | Gm1040 | chr5:029762792-029762851 | PROMOTER | 1.187888056 | 7.26667E-07 |
| 120977 | A_68_P27804065 | Hspa4 | chr11:053141780-053141839 | INSIDE | 0.815753815 | 7.47563E-07 |
| 139908 | A_68_P28077824 | Tmem101 | chr11:101970246-101970294 | INSIDE | 0.900087331 | 8.06667E-07 |
| 66262 | A_68_P21350018 | Osbpl6 | chr2:076207586-076207630 | INSIDE | 1.009565016 | 8.23367E-07 |
| 119530 | A_68_P21568755 | Rhov | chr2:118967460-118967519 | PROMOTER | 0.813151131 | 8.33373E-07 |
| 101537 | A_68_P26423661 | Svs7 | chr9:035362401-035362460 | PROMOTER | 0.750872224 | 8.7906E-07 |
| 197592 | A_68_P32746480 | Huwe1 | chrX:147146103-147146162 | INSIDE | 1.188037976 | 9.26683E-07 |
| 173270 | A_68_P25391170 | Olfr685 | chr7:105053332-105053391 | DOWNSTREAM | 0.770313787 | 9.47897E-07 |
| 1285 | A_68_P21395970 | Ssrp1 | chr2:084840546-084840605 | INSIDE | 0.761240319 | 1.03867E-06 |
| 103965 | A_68_P26136817 | Ctrl | chr8:108821343-108821387 | INSIDE | 0.814849499 | 1.08006E-06 |
| 36772 | A_68_P20841262 | Spna1 | chr1:176007350-176007406 | PROMOTER | 0.911350639 | 1.08663E-06 |
| 238863 | A_68_P31540758 | Ttr | chr18:020805219-020805278 | PROMOTER | 0.757586498 | 1.11405E-06 |
| 41764 | A_68_P23867765 | Ugt2b38 | chr5:088502983-088503042 | PROMOTER | 0.913071809 | 0.000001128 |
| 167622 | A_68_P25360306 | Dgat2 | chr7:099057154-099057207 | INSIDE | 0.764102736 | 1.1301E-06 |
| 33560 | A_68_P26434673 | Olfr901 | chr9:038182971-038183030 | DOWNSTREAM | 0.88033805 | 1.14693E-06 |
| 151762 | A_68_P24388849 | BC052883 | chr6:040373607-040373662 | INSIDE | 0.755570094 | 1.15617E-06 |
| 207595 | A_68_P32778421 | Pdha1 | chrX:155484942-155485001 | PROMOTER | 0.816226 | 1.1642E-06 |
| 14971 | A_68_P28631195 | Dio2 | chr12:091145586-091145645 | PROMOTER | 0.757695949 | 1.18079E-06 |
| 114475 | A_68_P21122732 | Lmx1b | chr2:033459763-033459822 | INSIDE | 0.755811223 | 1.21931E-06 |
| 47957 | A_68_P23198007 | Txndc12 | chr4:108348241-108348300 | INSIDE | 0.907786347 | 0.000001235 |
| 117157 | A_68_P23336629 | C1qa | chr4:136172754-136172813 | PROMOTER | 0.779406142 | 1.24577E-06 |
| 229591 | A_68_P24609764 | Snrpg | chr6:086337054-086337104 | PROMOTER | 0.895731936 | 1.2673E-06 |
| 5239 | A_68_P28233068 | Nt5c1b | chr12:010391845-010391904 | PROMOTER | 0.754705677 | 1.27432E-06 |
| 207958 | A_68_P30284257 | Adcy8 | chr15:064751250-064751294 | INSIDE | 0.882062856 | 1.28057E-06 |
| 232039 | A_68_P23091223 | Ifna14 | chr4:088042315-088042374 | DOWNSTREAM | 0.960522969 | 1.42767E-06 |
| 217312 | A_68_P25009531 | Pafah1b3 | chr7:025004995-025005051 | INSIDE | 0.959419005 | 1.50567E-06 |
| 204088 | A_68_P25402951 | Olfr491 | chr7:108104067-108104126 | PROMOTER | 0.779607395 | 0.000001541 |
| 47395 | A_68_P32204516 | AU041783 | chr19:057059831-057059890 | INSIDE | 0.806268342 | 1.54201E-06 |
| 212566 | A_68_P25381615 | Olfr578 | chr7:102862055-102862114 | PROMOTER | 0.856637221 | 0.000001548 |
| 59784 | A_68_P28157958 | Acox1 | chr11:116017880-116017939 | PROMOTER | 1.184869573 | 1.55605E-06 |
| 112479 | A_68_P22653950 | St6galnac5 | chr3:152921996-152922055 | PROMOTER | 0.829543111 | 1.65667E-06 |
| 51444 | A_68_P28073251 | Psme3 | chr11:101132799-101132847 | INSIDE | 0.751043731 | 1.66334E-06 |
| 11874 | A_68_P32555657 | Pja1 | chrX:095673754-095673813 | PROMOTER | 1.051805066 | 1.74005E-06 |
| 158954 | A_68_P21130456 | Phf19 | chr2:034737237-034737285 | PROMOTER | 0.939880676 | 1.76983E-06 |
| 17408 | A_68_P28875429 | Zfp184 | chr13:021953768-021953827 | INSIDE | 0.776767949 | 1.80637E-06 |
| 204297 | A_68_P23418030 | Rpl22 | chr4:151170727-151170776 | INSIDE | 0.796756493 | 1.81767E-06 |
| 49281 | A_68_P32001785 | Aldh1a1 | chr19:020665098-020665157 | PROMOTER | 0.797071162 | 1.83368E-06 |
| 4522 | A_68_P26992624 | Aldh8a1 | chr10:021066225-021066284 | PROMOTER | 0.756437521 | 1.8496E-06 |
| 1137 | A_68_P24128888 | Zscan21 | chr5:138342980-138343039 | PROMOTER | 0.787103513 | 1.86733E-06 |
| 192557 | A_68_P23962888 | Zfp644 | chr5:106937702-106937757 | PROMOTER | 0.99234264 | 1.88441E-06 |
| 92596 | A_68_P32460584 | Pnma3 | chrX:069313916-069313975 | PROMOTER | 0.936225361 | 2.03229E-06 |
| 26448 | A_68_P23197903 | Btf3l4 | chr4:108330765-108330824 | INSIDE | 0.775573249 | 2.03783E-06 |
| 7992 | A_68_P20617864 | Slc41a1 | chr1:133656945-133656989 | INSIDE | 0.765117485 | 2.0867E-06 |
| 217913 | A_68_P20594518 | Mgat5 | chr1:129132147-129132206 | PROMOTER | 0.761616944 | 2.09467E-06 |
| 151811 | A_68_P32465419 | Arhgap4 | chrX:070161805-070161864 | INSIDE | 0.867470295 | 2.12334E-06 |
| 230649 | A_68_P27831171 | Olfr30 | chr11:058277160-058277204 | PROMOTER | 1.026125115 | 2.15333E-06 |
| 111352 | A_68_P27918157 | Olfr392 | chr11:073631694-073631753 | PROMOTER | 1.008749938 | 2.19912E-06 |
| 23692 | A_68_P25777229 | Tnks | chr8:036436962-036437021 | PROMOTER | 0.866562558 | 2.20794E-06 |
| 138009 | A_68_P30824963 | Pou1f1 | chr16:065437833-065437892 | PROMOTER | 0.804625799 | 0.000002273 |
| 172473 | A_68_P20074972 | Paqr8 | chr1:020871213-020871272 | PROMOTER | 0.767407377 | 2.36363E-06 |
| 84127 | A_68_P30255537 | Trib1 | chr15:059480731-059480783 | INSIDE | 0.886197145 | 2.69167E-06 |
| 16898 | A_68_P23981828 | Pus1 | chr5:111015866-111015915 | INSIDE | 0.767327932 | 2.69931E-06 |
| 34105 | A_68_P31672374 | Kcnn2 | chr18:045681743-045681802 | PROMOTER | 0.777516925 | 2.72546E-06 |
| 102382 | A_68_P29152778 | Gpr150 | chr13:076526199-076526258 | PROMOTER | 0.854224967 | 2.91298E-06 |
| 88172 | A_68_P25171239 | Chrna7 | chr7:063088980-063089039 | INSIDE | 0.918816607 | 2.91767E-06 |
| 144861 | A_68_P24428207 | Repin1 | chr6:048525293-048525345 | INSIDE | 0.759654398 | 0.000002983 |
| 151397 | A_68_P30367941 | Gtpbp1 | chr15:079520255-079520313 | INSIDE | 0.843269477 | 3.07063E-06 |
| 64196 | A_68_P24633135 | Aldh1l1 | chr6:090516158-090516205 | PROMOTER | 0.87215565 | 3.10413E-06 |
| 199394 | A_68_P31602498 | Bin1 | chr18:032520560-032520604 | INSIDE | 0.767103231 | 3.12573E-06 |
| 39193 | A_68_P26207400 | Cdh13 | chr8:121166757-121166816 | PROMOTER | 0.80500371 | 3.2519E-06 |
| 23608 | A_68_P28068835 | Cnp1 | chr11:100393495-100393554 | INSIDE | 0.95818142 | 3.30333E-06 |
| 7753 | A_68_P26157745 | Dhx38 | chr8:112453924-112453983 | INSIDE | 0.765009688 | 0.000003359 |
| 145985 | A_68_P24973515 | Zscan22 | chr7:011799694-011799753 | INSIDE | 0.84685067 | 3.36673E-06 |
| 32642 | A_68_P21563062 | Gpr176 | chr2:117969538-117969595 | INSIDE | 0.885575834 | 3.40023E-06 |
| 8277 | A_68_P22379795 | Vangl1 | chr3:102337833-102337892 | PROMOTER | 0.89847218 | 0.00000341 |
| 136025 | A_68_P32562374 | Slc7a3 | chrX:097286737-097286785 | INSIDE | 0.952788568 | 3.46667E-06 |
| 126313 | A_68_P27260911 | S100b | chr10:075699240-075699299 | INSIDE | 0.93644455 | 3.51957E-06 |
| 121862 | A_68_P30421457 | Creld2 | chr15:088651546-088651591 | INSIDE | 0.747137639 | 3.60312E-06 |
| 199386 | A_68_P23893720 | Cxcl10 | chr5:093423076-093423135 | INSIDE | 0.793551713 | 3.6859E-06 |
| 161849 | A_68_P24471038 | Neurod6 | chr6:055612742-055612801 | PROMOTER | 0.92407993 | 0.000003963 |
| 110187 | A_68_P30980986 | Pigp | chr16:094482197-094482256 | PROMOTER | 0.882025688 | 4.0744E-06 |
| 129048 | A_68_P24421978 | Cntnap2 | chr6:047173214-047173273 | PROMOTER | 1.046102208 | 4.08026E-06 |
| 226389 | A_68_P25456719 | Arl6ip1 | chr7:117925829-117925888 | PROMOTER | 0.889361618 | 0.000004159 |
| 85123 | A_68_P20799893 | Tada1l | chr1:168217058-168217113 | INSIDE | 0.765957955 | 4.20863E-06 |
| 78158 | A_68_P32683825 | BC031748 | chrX:132391381-132391440 | PROMOTER | 0.752499901 | 4.23224E-06 |
| 164035 | A_68_P31503692 | Osbpl1a | chr18:013084080-013084139 | INSIDE | 0.831190198 | 0.000004241 |
| 185180 | A_68_P28517357 | Spg3a | chr12:070852013-070852072 | INSIDE | 0.859789876 | 4.64927E-06 |
| 113842 | A_68_P32288212 | Rp2h | chrX:019520751-019520810 | PROMOTER | 0.910907302 | 4.72931E-06 |
| 157101 | A_68_P30013674 | Amacr | chr15:010939919-010939976 | INSIDE | 0.766636835 | 4.81221E-06 |
| 172793 | A_68_P29596903 | Olfr732 | chr14:049207151-049207210 | PROMOTER | 0.7469723 | 5.01373E-06 |
| 88092 | A_68_P22346088 | Mrps21 | chr3:095954038-095954097 | INSIDE | 0.752586404 | 0.000005086 |
| 35108 | A_68_P26847732 | Osbpl10 | chr9:114912953-114913012 | PROMOTER | 0.900246964 | 5.29363E-06 |
| 181292 | A_68_P31099833 | Fahd1 | chr17:024575290-024575349 | DOWNSTREAM | 0.781358466 | 5.33137E-06 |
| 193261 | A_68_P25114503 | Htatip2 | chr7:049627643-049627694 | INSIDE | 0.783397923 | 5.5913E-06 |
| 37661 | A_68_P30718946 | Gramd1c | chr16:043902597-043902656 | INSIDE | 0.796182957 | 5.6668E-06 |
| 88234 | A_68_P27991078 | Dhx40 | chr11:086621945-086622004 | INSIDE | 0.850681979 | 5.8222E-06 |
| 183076 | A_68_P28186427 | Dcxr | chr11:120542273-120542329 | INSIDE | 0.776794885 | 5.9708E-06 |
| 115783 | A_68_P22312820 | Rhbg | chr3:088344027-088344081 | PROMOTER | 0.749911367 | 6.03344E-06 |
| 31776 | A_68_P27919465 | Olfr43 | chr11:074024502-074024561 | PROMOTER | 0.768200955 | 6.05357E-06 |
| 144537 | A_68_P29329260 | Gpbp1 | chr13:112611620-112611679 | PROMOTER | 1.027176773 | 0.00000634 |
| 214585 | A_68_P32213490 | Gfra1 | chr19:058505692-058505745 | INSIDE | 0.840141522 | 7.73921E-06 |
| 171324 | A_68_P27544876 | Olfr770 | chr10:128541440-128541499 | PROMOTER | 0.928174889 | 8.15943E-06 |
| 112579 | A_68_P22147220 | Postn | chr3:054448604-054448663 | PROMOTER | 0.798176265 | 8.2339E-06 |
| 105146 | A_68_P22920338 | Olfr270 | chr4:052989765-052989824 | PROMOTER | 0.781532284 | 0.000008282 |
| 225551 | A_68_P28873855 | Olfr11 | chr13:021651291-021651350 | PROMOTER | 0.926085369 | 8.34067E-06 |
| 104040 | A_68_P31401144 | Haao | chr17:083756814-083756873 | PROMOTER | 0.843826265 | 8.48167E-06 |
| 103928 | A_68_P26542026 | Scamp5 | chr9:057268908-057268967 | PROMOTER | 0.804170439 | 8.76473E-06 |
| 40845 | A_68_P25503052 | Kctd13 | chr7:126721107-126721166 | INSIDE | 0.764886273 | 0.000008955 |
| 197435 | A_68_P23575610 | Abhd1 | chr5:031220799-031220858 | PROMOTER | 0.787010726 | 9.29683E-06 |
| 37899 | A_68_P32696412 | Morc4 | chrX:135221221-135221280 | PROMOTER | 0.795736678 | 0.00000959 |
| 164918 | A_68_P23525877 | AI847670 | chr5:020933955-020934014 | PROMOTER | 0.746296824 | 9.67433E-06 |
| 103186 | A_68_P24318665 | Grm8 | chr6:028081450-028081509 | INSIDE | 0.752558719 | 0.000009778 |
| 232655 | A_68_P30014079 | Adamts12 | chr15:011005880-011005939 | PROMOTER | 0.757809829 | 9.83477E-06 |
| 70507 | A_68_P30562922 | Snai2 | chr16:014614741-014614798 | PROMOTER | 0.817310787 | 1.01697E-05 |
| 21647 | A_68_P25834929 | Slc25a4 | chr8:047715069-047715128 | PROMOTER | 0.746750518 | 1.03534E-05 |
| 65747 | A_68_P26748157 | Rasa2 | chr9:096441176-096441233 | PROMOTER | 0.836017014 | 1.03773E-05 |
| 164679 | A_68_P29596929 | Olfr733 | chr14:049219290-049219349 | DOWNSTREAM | 0.812860134 | 1.11843E-05 |
| 82357 | A_68_P25528918 | Hmx2 | chr7:131336046-131336105 | PROMOTER | 0.768112721 | 1.14967E-05 |
| 96735 | A_68_P22351870 | Gja8 | chr3:097009858-097009917 | INSIDE | 0.961790546 | 0.0000127 |
| 105834 | A_68_P26282979 | Pdgfd | chr9:006166273-006166332 | PROMOTER | 0.945592699 | 1.28439E-05 |
| 203728 | A_68_P31656250 | Gpr151 | chr18:042708051-042708106 | PROMOTER | 0.758004339 | 1.30198E-05 |
| 235006 | A_68_P24643648 | Mrps25 | chr6:092150788-092150845 | PROMOTER | 0.983258069 | 1.34237E-05 |
| 212409 | A_68_P28874247 | Hist1h2bm | chr13:021727639-021727698 | PROMOTER | 0.992795997 | 1.38668E-05 |
| 46210 | A_68_P28671802 | Ptpn21 | chr12:099133802-099133861 | INSIDE | 1.012657643 | 1.43912E-05 |
| 79572 | A_68_P27933941 | Gosr1 | chr11:076578350-076578409 | INSIDE | 0.851912897 | 1.45126E-05 |
| 85958 | A_68_P23289839 | Hmgb4 | chr4:127765422-127765481 | PROMOTER | 0.75383508 | 0.0000147 |
| 132533 | A_68_P28328470 | Sh3yl1 | chr12:031500049-031500108 | INSIDE | 1.034994845 | 1.53439E-05 |
| 47142 | A_68_P23438173 | Prkcz | chr4:154208200-154208259 | PROMOTER | 0.780958118 | 1.53691E-05 |
| 237407 | A_68_P21116161 | Pip5kl1 | chr2:032398488-032398542 | INSIDE | 0.79435293 | 1.55467E-05 |
| 93198 | A_68_P29621334 | Mcpt8 | chr14:055038503-055038562 | INSIDE | 0.878769062 | 0.000015557 |
| 95790 | A_68_P29942822 | Hs6st3 | chr14:118269565-118269624 | PROMOTER | 0.839379063 | 1.55987E-05 |
| 3578 | A_68_P22276702 | Tdo2 | chr3:082061865-082061924 | PROMOTER | 0.82365727 | 1.57967E-05 |
| 207567 | A_68_P27662579 | Fancl | chr11:026283515-026283570 | PROMOTER | 0.783763457 | 1.58094E-05 |
| 231753 | A_68_P26530344 | Nrg4 | chr9:055078684-055078743 | INSIDE | 0.77977884 | 1.6043E-05 |
| 236405 | A_68_P20886148 | Fbxo28 | chr1:184176769-184176828 | INSIDE | 0.835524926 | 1.61433E-05 |
| 103180 | A_68_P24992062 | Gpr4 | chr7:018370813-018370865 | PROMOTER | 0.978724624 | 1.69667E-05 |
| 16114 | A_68_P21904938 | Ogfr | chr2:180524848-180524894 | INSIDE | 0.873946763 | 1.76401E-05 |
| 143419 | A_68_P22666338 | Tnni3k | chr3:154989676-154989735 | INSIDE | 0.788132964 | 0.000018179 |
| 113244 | A_68_P32728749 | Htr2c | chrX:142404962-142405021 | INSIDE | 0.870642075 | 1.83878E-05 |
| 101167 | A_68_P24851596 | Tas2r107 | chr6:131625495-131625554 | INSIDE | 0.761814844 | 1.88167E-05 |
| 233791 | A_68_P27478493 | Rab3ip | chr10:116352903-116352962 | INSIDE | 0.9563467 | 1.89048E-05 |
| 119305 | A_68_P20646965 | Zfp281 | chr1:138437657-138437716 | PROMOTER | 1.194038526 | 1.9434E-05 |
| 104057 | A_68_P29715854 | Rcbtb2 | chr14:071872614-071872673 | PROMOTER | 0.879342279 | 1.9516E-05 |
| 97691 | A_68_P31708223 | Lox | chr18:052659197-052659252 | PROMOTER | 0.784016651 | 1.97667E-05 |
| 226041 | A_68_P25582652 | Olfr522-Olfr523 | chr7:140023198-140023257 | DIVERGENT_PROMOTER | 0.903129682 | 2.07798E-05 |
| 72575 | A_68_P27285688 | Oaz1 | chr10:080229118-080229177 | PROMOTER | 0.751834434 | 2.13585E-05 |
| 182287 | A_68_P25367265 | Ppme1 | chr7:100243579-100243638 | INSIDE | 0.820227041 | 2.23817E-05 |
| 152939 | A_68_P29600283 | Rnase6 | chr14:050053353-050053412 | DOWNSTREAM | 0.82860909 | 2.29699E-05 |
| 227179 | A_68_P30570954 | Ppm1f | chr16:016813554-016813607 | PROMOTER | 0.932371078 | 2.41333E-05 |
| 213895 | A_68_P20610046 | Daf2 | chr1:132250741-132250800 | PROMOTER | 0.748462525 | 0.000024461 |
| 134793 | A_68_P26336185 | Chordc1 | chr9:018040021-018040080 | PROMOTER | 0.80241106 | 2.52033E-05 |
| 168625 | A_68_P22413533 | Wdr47 | chr3:108720780-108720839 | INSIDE | 0.912019444 | 2.54076E-05 |
| 230789 | A_68_P28059230 | Cdc6 | chr11:098724463-098724522 | INSIDE | 0.937010717 | 2.55039E-05 |
| 75202 | A_68_P23012926 | Tle1 | chr4:071687729-071687779 | PROMOTER | 0.881793133 | 2.58E-05 |
| 199019 | A_68_P20285552 | Gpr1 | chr1:063116281-063116340 | DOWNSTREAM | 0.770444197 | 2.59457E-05 |
| 152631 | A_68_P28547649 | Rhoj | chr12:076223491-076223550 | PROMOTER | 0.780301115 | 2.67313E-05 |
| 219931 | A_68_P30568838 | Yars2 | chr16:016215834-016215893 | PROMOTER | 1.037649883 | 2.88423E-05 |
| 220277 | A_68_P27850808 | Ulk2 | chr11:061669358-061669417 | INSIDE | 0.828068316 | 2.94583E-05 |
| 199592 | A_68_P23301311 | Serinc2 | chr4:129781783-129781839 | PROMOTER | 0.975471028 | 0.000030171 |
| 200814 | A_68_P23973492 | Slc26a1 | chr5:108916349-108916408 | PROMOTER | 0.882505681 | 3.18585E-05 |
| 144666 | A_68_P27340141 | Slc25a3 | chr10:090554165-090554224 | PROMOTER | 0.783284492 | 3.21733E-05 |
| 75716 | A_68_P29072980 | Rmi1 | chr13:058402433-058402492 | PROMOTER | 0.816445579 | 0.00003397 |
| 43912 | A_68_P26434827 | Olfr905 | chr9:038220381-038220440 | PROMOTER | 0.81967073 | 3.44767E-05 |
| 6460 | A_68_P20373145 | Kcne4 | chr1:078694730-078694789 | PROMOTER | 0.798796825 | 3.47667E-05 |
| 92647 | A_68_P32530331 | MGC118250 | chrX:088740731-088740790 | PROMOTER | 0.847176745 | 3.50027E-05 |
| 75061 | A_68_P30500774 | Hoxc12 | chr15:102760672-102760731 | PROMOTER | 0.80534326 | 3.59758E-05 |
| 234377 | A_68_P24183706 | Uspl1 | chr5:149496858-149496917 | INSIDE | 0.800747989 | 3.64573E-05 |
| 120206 | A_68_P26075781 | Mt3 | chr8:097043249-097043301 | DOWNSTREAM | 0.749434616 | 0.000039383 |
| 54414 | A_68_P25363534 | Slco2b1 | chr7:099585711-099585760 | PROMOTER | 0.774707208 | 4.23703E-05 |
| 186921 | A_68_P21371780 | Pde1a | chr2:079707279-079707338 | INSIDE | 0.832052197 | 4.32567E-05 |
| 60195 | A_68_P30080928 | EG432939 | chr15:025355671-025355730 | PROMOTER | 0.804764034 | 4.49574E-05 |
| 121564 | A_68_P29101900 | Hiatl1 | chr13:065132236-065132295 | PROMOTER | 0.78890096 | 4.84209E-05 |
| 114814 | A_68_P23321263 | Ccdc21 | chr4:133403100-133403159 | INSIDE | 0.852116848 | 4.98403E-05 |
| 82350 | A_68_P26989962 | Ahi1 | chr10:020639365-020639424 | PROMOTER | 0.894053557 | 5.38707E-05 |
| 110706 | A_68_P22346143 | Gm129 | chr3:095965919-095965978 | INSIDE | 0.773940215 | 5.47043E-05 |
| 217374 | A_68_P21796131 | Top1 | chr2:160335118-160335177 | PROMOTER | 0.804066841 | 0.00005877 |
| 80318 | A_68_P21639375 | Pcna-Cds2 | chr2:131944970-131945029 | DIVERGENT_PROMOTER | 0.802544738 | 5.89251E-05 |
| 97031 | A_68_P32055554 | Il33 | chr19:029993505-029993564 | INSIDE | 0.746316603 | 6.21829E-05 |
| 68295 | A_68_P29337705 | Dhx29 | chr13:114049773-114049832 | INSIDE | 0.866178942 | 6.27973E-05 |
| 16866 | A_68_P32581169 | Fgf16 | chrX:101964917-101964976 | PROMOTER | 0.763671783 | 6.49281E-05 |
| 140988 | A_68_P30793506 | Olfr174 | chr16:058767279-058767338 | INSIDE | 0.922201853 | 6.83528E-05 |
| 190402 | A_68_P31762256 | Adrb2 | chr18:062306966-062307025 | PROMOTER | 0.781459698 | 7.34577E-05 |
| 44487 | A_68_P32227261 | Eif3s10 | chr19:060847373-060847432 | PROMOTER | 0.860468996 | 7.81587E-05 |
| 64958 | A_68_P31604492 | Tslp | chr18:032955744-032955803 | PROMOTER | 0.80832031 | 8.45823E-05 |
| 127500 | A_68_P26524434 | Cyp19a1 | chr9:053994836-053994895 | PROMOTER | 0.872139123 | 0.000087555 |
| 10974 | A_68_P32026463 | Foxd4 | chr19:024972128-024972187 | PROMOTER | 0.838775464 | 9.16174E-05 |
| 153672 | A_68_P25892556 | Galnt7 | chr8:060549227-060549286 | PROMOTER | 0.987166522 | 9.90377E-05 |
| 210205 | A_68_P27928962 | Rph3al | chr11:075726003-075726049 | INSIDE | 0.927413363 | 0.000101195 |
| 12384 | A_68_P32466449 | Opn1mw | chrX:070379876-070379935 | PROMOTER | 0.803307849 | 0.000109957 |
| 21768 | A_68_P22249111 | Fstl5 | chr3:076161591-076161650 | INSIDE | 0.813440925 | 0.000111781 |
| 231460 | A_68_P23592720 | BC023882 | chr5:034484398-034484457 | INSIDE | 1.039320577 | 0.000122061 |
| 216721 | A_68_P22789546 | Fhl5 | chr4:025332113-025332172 | INSIDE | 0.992051135 | 0.000125787 |
| 20935 | A_68_P30925861 | Gabpa | chr16:084716732-084716791 | PROMOTER | 0.884615702 | 0.000130695 |
| 172658 | A_68_P21310287 | Bbs5 | chr2:069448463-069448522 | INSIDE | 0.778110363 | 0.000132954 |
| 7823 | A_68_P21054153 | Thnsl1 | chr2:021126915-021126974 | INSIDE | 0.749420349 | 0.000135745 |
| 33134 | A_68_P32610321 | Klhl4 | chrX:110592504-110592563 | INSIDE | 1.051771131 | 0.00014401 |
| 200144 | A_68_P20005216 | Rp1h | chr1:004348782-004348841 | INSIDE | 0.818681895 | 0.000146597 |
| 56177 | A_68_P23088480 | Mllt3 | chr4:087507611-087507670 | PROMOTER | 0.878917371 | 0.000149868 |
| 55033 | A_68_P26434021 | Olfr894 | chr9:037966332-037966391 | PROMOTER | 0.777736278 | 0.000151015 |
| 223103 | A_68_P25389909 | Olfr667 | chr7:104794639-104794698 | PROMOTER | 0.872995087 | 0.000158856 |
| 227062 | A_68_P30652748 | Osta-Zdhhc19 | chr16:032408014-032408070 | DIVERGENT_PROMOTER | 0.821208484 | 0.000165386 |
| 128836 | A_68_P21979264 | Cpb1 | chr3:020467852-020467910 | PROMOTER | 0.864172014 | 0.000170468 |
| 120172 | A_68_P26254631 | BC021891 | chr8:128797511-128797555 | INSIDE | 0.762908231 | 0.000171097 |
| 17388 | A_68_P20349767 | Ttll4 | chr1:074597190-074597249 | INSIDE | 0.792043411 | 0.00017121 |
| 191152 | A_68_P24298787 | Cadps2 | chr6:023794737-023794796 | PROMOTER | 0.992242532 | 0.000193442 |
| 98593 | A_68_P21406301 | Olfr153 | chr2:087330162-087330221 | PROMOTER | 0.961686105 | 0.000202964 |
| 131688 | A_68_P29107631 | Rsl1 | chr13:067671024-067671083 | PROMOTER | 0.84919842 | 0.000218543 |
| 23455 | A_68_P32693753 | Trap1a | chrX:134680239-134680292 | INSIDE | 0.818809323 | 0.000219619 |
| 189647 | A_68_P28821616 | Chrm3 | chr13:010360299-010360343 | PROMOTER | 1.03159948 | 0.000231671 |
| 199416 | A_68_P21403595 | Olfr1093 | chr2:086584585-086584644 | PROMOTER | 0.781282483 | 0.000237127 |
| 117408 | A_68_P30382856 | Naga | chr15:082165447-082165502 | INSIDE | 0.758849696 | 0.000244991 |
| 59971 | A_68_P30360628 | C1qtnf6 | chr15:078356180-078356233 | INSIDE | 0.905669419 | 0.000253018 |
| 231958 | A_68_P27269188 | Sumo3 | chr10:077048530-077048589 | PROMOTER | 0.790797915 | 0.000253332 |
| 9099 | A_68_P31152687 | Psmb8 | chr17:033810133-033810192 | INSIDE | 0.76743424 | 0.00026316 |
| 31690 | A_68_P31206443 | Abcc10 | chr17:045792913-045792968 | PROMOTER | 0.749826124 | 0.000271262 |
| 29924 | A_68_P22606543 | Clca5 | chr3:145035245-145035303 | INSIDE | 0.804833475 | 0.000273132 |
| 233584 | A_68_P24568061 | Ctnna2 | chr6:077906793-077906852 | INSIDE | 0.851948586 | 0.000286469 |
| 153263 | A_68_P22376191 | Atp1a1 | chr3:101732947-101733003 | INSIDE | 0.759652949 | 0.000295024 |
| 27071 | A_68_P20270767 | Cd28 | chr1:060693353-060693412 | INSIDE | 0.866344126 | 0.000299119 |
| 72955 | A_68_P29667833 | Msra | chr14:063409522-063409575 | INSIDE | 0.997894035 | 0.00032559 |
| 138042 | A_68_P24854951 | Tas2r116 | chr6:132818760-132818819 | PROMOTER | 0.756340303 | 0.000340596 |
| 206510 | A_68_P22291942 | Tigd4 | chr3:084680687-084680746 | INSIDE | 0.765718922 | 0.000363049 |
| 115936 | A_68_P28552894 | Mthfd1 | chr12:077174755-077174812 | INSIDE | 0.921890662 | 0.000381829 |
| 169626 | A_68_P32703653 | Col4a6 | chrX:136718080-136718139 | INSIDE | 0.792061875 | 0.000394063 |
| 108426 | A_68_P23820405 | Spink2 | chr5:078284586-078284645 | INSIDE | 0.749570094 | 0.000400283 |
| 25475 | A_68_P23885222 | Gm1960 | chr5:091862871-091862930 | INSIDE | 0.88998321 | 0.000514736 |
| 100671 | A_68_P29751187 | Narg1l-Mtrf1 | chr14:078130818-078130877 | DIVERGENT_PROMOTER | 0.766828054 | 0.000552427 |
| 163604 | A_68_P31165723 | Trim26 | chr17:036440320-036440379 | PROMOTER | 1.003220279 | 0.000580499 |
| 204825 | A_68_P27110502 | Popdc3 | chr10:044979941-044980000 | INSIDE | 0.790392203 | 0.000646338 |
| 108393 | A_68_P27323502 | Pmch | chr10:087518349-087518408 | PROMOTER | 0.80684361 | 0.000844651 |
| 83688 | A_68_P26436938 | Olfr935 | chr9:038749137-038749196 | PROMOTER | 0.752509884 | 0.000947487 |
| 7279 | A_68_P27916763 | Olfr20 | chr11:073169893-073169952 | PROMOTER | 0.794477764 | 0.000985488 |
|  |  |  |  |  |  |  |
