# Supplementary material for: Genome-Wide Analyses of Nkx2-1 Binding to Transcriptional Target Genes Uncover Novel Regulatory Patterns Conserved in Lung Development and Tumors
Source: PLoS One. 2012 Jan 5;7(1):e29907. doi: 10.1371/journal.pone.0029907 (PMC3252372; doi:10.1371/journal.pone.0029907)
Supplement: Table S2 — Nkx2-1 target genes expressed in lung development and correlated to NKX2-1 levels in human lung tumor datasets. (DOC) [file pone.0029907.s007.doc]

| ***Table S2 Nkx2-1 target genes expressed in lung development and correlated to NKX2-1 levels in human lung tumor datasets*** | | | | | | | | | |
| --- | --- | --- | --- | --- | --- | --- | --- | --- | --- |
| ***E11.5 lung targets*** | | | | | ***E19.5 lung targets*** | | | | |
|  |  | ***Pearson correlation coefficients*** | | |  |  | ***Pearson correlation coefficients*** | | |
| ***Symbol*** | ***Entrez Gene ID*** | ***GSE12667*** | ***Bhattacharjee adenocarcinomas*** | ***Shedden UMich*** | ***Symbol*** | ***Entrez Gene ID*** | ***GSE12667*** | ***Bhattacharjee adenocarcinomas*** | ***Shedden UMich*** |
| TMPRSS2 | 7113 | 0.624 | 0.560 | 0.539 | SLC34A2 | 10568 | 0.749 | NA | 0.608 |
| NISCH | 11188 | 0.522 | 0.136 | 0.432 | WFDC2 | 10406 | 0.608 | 0.452 | 0.357 |
| CTSD | 1509 | 0.475 | 0.345 | 0.158 | ARRB1 | 408 | 0.547 | NA | 0.431 |
| CRY2 | 1408 | 0.472 | -0.113 | 0.380 | RGL2 | 5863 | 0.520 | 0.164 | 0.110 |
| CREBBP | 1387 | 0.456 | 0.155 | 0.334 | VAPA | 9218 | 0.464 | 0.267 | 0.177 |
| NFIB | 4781 | 0.449 | 0.276 | 0.459 | ABR | 29 | 0.436 | 0.058 | 0.437 |
| CLPTM1L | 81037 | 0.440 | NA | NA | NFKBIA | 4792 | 0.422 | 0.426 | 0.245 |
| TMED4 | 222068 | 0.432 | NA | NA | PRKCZ | 5590 | 0.413 | 0.331 | 0.210 |
| CCNL2 | 81669 | 0.420 | NA | 0.225 | RAB17 | 64284 | 0.405 | NA | 0.332 |
| ALCAM | 214 | 0.417 | 0.188 | 0.241 | MRPS25 | 64432 | 0.402 | NA | NA |
| ROCK1 | 6093 | 0.408 | NA | 0.088 | SLC25A4 | 291 | 0.397 | 0.157 | 0.453 |
| MXD4 | 10608 | 0.401 | 0.169 | 0.251 | NRP1 | 8829 | 0.394 | 0.431 | 0.263 |
| PPM1A | 5494 | 0.400 | 0.041 | 0.120 | PKD1 | 5310 | 0.388 | -0.130 | 0.109 |
| CYB561 | 1534 | 0.400 | 0.187 | 0.274 | SLC41A1 | 254428 | 0.366 | NA | NA |
| YAP1 | 10413 | 0.388 | 0.247 | 0.120 | REV3L | 5980 | 0.357 | 0.024 | 0.316 |
| SMAD7 | 4092 | 0.387 | 0.297 | 0.236 | LY6E | 4061 | 0.357 | 0.081 | 0.240 |
| SF3A3 | 10946 | 0.386 | -0.220 | 0.309 | GGA2 | 23062 | 0.349 | 0.173 | 0.377 |
| ZC3H11A | 9877 | 0.385 | 0.114 | 0.310 | LPL | 4023 | 0.342 | 0.300 | 0.386 |
| RANBP9 | 10048 | 0.381 | 0.156 | 0.289 | TOP1 | 7150 | 0.326 | 0.320 | 0.240 |
| DHRS1 | 115817 | 0.356 | 0.096 | 0.347 | CLOCK | 9575 | 0.319 | -0.108 | 0.307 |
| NFYC | 4802 | 0.352 | -0.166 | 0.146 | TCTA | 6988 | 0.314 | -0.077 | 0.212 |
| INTS3 | 65123 | 0.347 | -0.071 | 0.239 | PEX6 | 5190 | 0.312 | -0.178 | 0.055 |
| LDOC1L | 84247 | 0.345 | NA | NA | DHX40 | 79665 | 0.308 | NA | 0.311 |
| AGPAT3 | 56894 | 0.341 | NA | -0.024 | ADCY7 | 113 | 0.295 | 0.336 | 0.259 |
| FOXA1 | 3169 | 0.340 | 0.242 | 0.188 | ANK3 | 288 | 0.290 | 0.197 | 0.340 |
| ZBTB22 | 9278 | 0.339 | 0.228 | 0.152 | DPM2 | 8818 | 0.286 | 0.201 | 0.251 |
| AP4S1 | 11154 | 0.335 | NA | 0.134 | EXOC8 | 149371 | 0.282 | NA | NA |
| THUMPD1 | 55623 | 0.335 | 0.245 | 0.361 | S100A13 | 6284 | 0.268 | 0.500 | 0.395 |
| AHCYL1 | 10768 | 0.329 | 0.268 | 0.329 | LDB1 | 8861 | 0.267 | 0.051 | -0.086 |
| SRRM1 | 10250 | 0.328 | NA | 0.128 | RASGRF1 | 5923 | 0.260 | 0.295 | 0.274 |
| ZFP90 | 146198 | 0.327 | NA | NA | SERPINF2 | 5345 | 0.259 | -0.031 | 0.008 |
| KLF9 | 687 | 0.325 | 0.217 | 0.207 | CAT | 847 | 0.259 | 0.179 | 0.253 |
| GPR177 | 79971 | 0.323 | NA | 0.421 | IL1R1 | 3554 | 0.249 | 0.373 | 0.220 |
| RAB33B | 83452 | 0.314 | NA | 0.283 | ATP1A1 | 476 | 0.247 | 0.077 | 0.320 |
| ANGEL2 | 90806 | 0.311 | 0.039 | 0.326 | ATP6AP1 | 537 | 0.246 | -0.057 | 0.238 |
| PLD4 | 122618 | 0.307 | NA | NA | AQP1 | 358 | 0.229 | 0.419 | 0.355 |
| APBB1 | 322 | 0.306 | 0.004 | -0.001 | ATM | 472 | 0.218 | 0.021 | -0.189 |
| RAB25 | 57111 | 0.306 | NA | 0.231 | TSPYL4 | 23270 | 0.205 | -0.295 | 0.124 |
| WDR26 | 80232 | 0.301 | NA | -0.028 | RNASE4 | 6038 | 0.194 | 0.127 | 0.111 |
| AKTIP | 64400 | 0.298 | NA | 0.222 | ZNF644 | 84146 | 0.194 | NA | NA |
| CD63 | 967 | 0.290 | 0.439 | 0.240 | PRPF4B | 8899 | 0.193 | -0.076 | 0.168 |
| AKAP8L | 26993 | 0.287 | -0.091 | 0.233 | STARD10 | 10809 | 0.192 | NA | NA |
| DPM2 | 8818 | 0.286 | 0.201 | 0.251 | EPS8 | 2059 | 0.192 | 0.324 | 0.282 |
| SCP2 | 6342 | 0.285 | 0.215 | 0.357 | TOLLIP | 54472 | 0.182 | NA | -0.098 |
| ZNF23 | 7571 | 0.285 | 0.047 | 0.135 | ADRB2 | 154 | 0.179 | 0.212 | 0.261 |
| CSDE1 | 7812 | 0.284 | NA | -0.038 | OSBPL9 | 114883 | 0.177 | NA | 0.205 |
| USP4 | 7375 | 0.283 | 0.177 | 0.375 | PITPNA | 5306 | 0.167 | 0.098 | 0.022 |
| TPP1 | 1200 | 0.281 | 0.155 | 0.026 | MYO1D | 4642 | 0.165 | 0.052 | 0.077 |
| ERBB3 | 2065 | 0.274 | 0.232 | 0.227 | GNA11 | 2767 | 0.165 | -0.119 | 0.222 |
| RGS3 | 5998 | 0.270 | 0.270 | 0.202 | BAG3 | 9531 | 0.160 | NA | 0.227 |
| BLZF1 | 8548 | 0.270 | NA | NA | YY1 | 7528 | 0.158 | -0.064 | -0.038 |
| ARMCX2 | 9823 | 0.268 | -0.050 | 0.031 | SLC7A2 | 6542 | 0.156 | 0.175 | -0.037 |
| SLC39A8 | 64116 | 0.261 | 0.511 | 0.321 | FBXW2 | 26190 | 0.156 | NA | 0.262 |
| RFNG | 5986 | 0.261 | -0.014 | 0.088 | ACTB | 60 | 0.150 | 0.147 | 0.056 |
| PCBD1 | 5092 | 0.254 | 0.085 | 0.177 | GPBP1 | 65056 | 0.143 | NA | NA |
| PTOV1 | 53635 | 0.252 | -0.232 | -0.019 | ZNF263 | 10127 | 0.142 | -0.080 | 0.319 |
| CDCA7L | 55536 | 0.244 | NA | NA | DUS1L | 64118 | 0.139 | NA | 0.115 |
| DHRS7B | 25979 | 0.244 | NA | 0.246 | RPL22 | 6146 | 0.136 | NA | 0.316 |
| AASS | 10157 | 0.241 | 0.246 | 0.120 | ARIH2 | 10425 | 0.135 | 0.131 | 0.131 |
| PTPRF | 5792 | 0.240 | -0.179 | 0.150 | MAX | 4149 | 0.133 | -0.096 | -0.010 |
| BCKDK | 10295 | 0.232 | 0.130 | 0.032 | CRELD2 | 79174 | 0.126 | NA | -0.179 |
| SLTM | 79811 | 0.226 | NA | 0.216 | ACOX1 | 51 | 0.118 | 0.104 | -0.021 |
| XPR1 | 9213 | 0.220 | NA | NA | SCGB1A1 | 7356 | 0.107 | 0.188 | 0.056 |
| ATP13A1 | 57130 | 0.219 | NA | 0.076 | BTD | 686 | 0.100 | 0.078 | 0.073 |
| GUSB | 2990 | 0.218 | 0.222 | 0.158 | SUMO3 | 6612 | 0.092 | -0.121 | 0.021 |
| PHF13 | 148479 | 0.216 | NA | NA | PPP2R4 | 5524 | 0.089 | -0.175 | -0.150 |
| NUCKS1 | 64710 | 0.215 | NA | NA | CKM | 1158 | 0.088 | 0.068 | 0.050 |
| EPC1 | 80314 | 0.212 | NA | NA | IARS2 | 55699 | 0.087 | 0.110 | 0.142 |
| EFNA4 | 1945 | 0.212 | 0.133 | 0.230 | B4GALT3 | 8703 | 0.084 | 0.148 | 0.057 |
| SNTA1 | 6640 | 0.211 | 0.006 | 0.057 | PIAS2 | 9063 | 0.081 | -0.033 | -0.109 |
| KLC4 | 89953 | 0.208 | NA | NA | HUWE1 | 10075 | 0.081 | -0.272 | -0.035 |
| SOX4 | 6659 | 0.201 | 0.083 | 0.142 | IRS1 | 3667 | 0.072 | -0.010 | 0.032 |
| MVK | 4598 | 0.195 | 0.073 | 0.133 | TADA1L | 117143 | 0.068 | NA | NA |
| POLR2C | 5432 | 0.192 | 0.210 | 0.315 | GLG1 | 2734 | 0.066 | NA | -0.140 |
| LPP | 4026 | 0.188 | 0.236 | 0.278 | RDX | 5962 | 0.060 | 0.084 | -0.148 |
| HGS | 9146 | 0.187 | -0.011 | 0.092 | FARS2 | 10667 | 0.056 | 0.044 | 0.079 |
| EDF1 | 8721 | 0.180 | NA | -0.041 | LAMP2 | 3920 | 0.051 | -0.002 | -0.043 |
| RHOB | 388 | 0.177 | 0.363 | 0.279 | RAB24 | 53917 | 0.048 | NA | NA |
| DDB1 | 1642 | 0.165 | -0.341 | -0.015 | SSRP1 | 6749 | 0.036 | -0.353 | -0.053 |
| MYO1D | 4642 | 0.165 | 0.052 | 0.077 | DPYSL3 | 1809 | 0.034 | -0.010 | 0.073 |
| GNA11 | 2767 | 0.165 | -0.119 | 0.222 | RARRES2 | 5919 | 0.032 | 0.114 | -0.179 |
| MLLT3 | 4300 | 0.160 | 0.031 | 0.201 | SSPN | 8082 | 0.021 | 0.002 | 0.016 |
| NONO | 4841 | 0.147 | -0.172 | 0.116 | TSPAN6 | 7105 | 0.019 | -0.079 | 0.064 |
| GTF2H4 | 2968 | 0.143 | -0.073 | -0.024 | ENPP2 | 5168 | 0.016 | 0.165 | -0.172 |
| PUM2 | 23369 | 0.143 | -0.013 | 0.297 | KCNJ8 | 3764 | 0.013 | 0.231 | -0.023 |
| MLH1 | 4292 | 0.137 | -0.022 | -0.085 | ECH1 | 1891 | 0.012 | -0.043 | 0.102 |
| BRD4 | 23476 | 0.136 | -0.255 | 0.004 | RAC1 | 5879 | 0.005 | 0.042 | -0.099 |
| UBP1 | 7342 | 0.132 | NA | 0.077 | PDZK1IP1 | 10158 | -0.005 | 0.179 | -0.063 |
| USP9X | 8239 | 0.124 | -0.015 | 0.098 | PAFAH1B3 | 5050 | -0.007 | -0.147 | -0.156 |
| ERO1LB | 56605 | 0.123 | NA | 0.089 | ANXA7 | 310 | -0.010 | 0.015 | -0.009 |
| LONP2 | 83752 | 0.116 | NA | NA | GADD45B | 4616 | -0.019 | 0.141 | 0.054 |
| SPSB1 | 80176 | 0.115 | NA | 0.130 | PTRF | 284119 | -0.020 | 0.018 | -0.032 |
| MFGE8 | 4240 | 0.114 | 0.051 | 0.138 | ZKSCAN1 | 7586 | -0.032 | -0.354 | -0.267 |
| CAMLG | 819 | 0.113 | 0.042 | 0.382 | TAF6 | 6878 | -0.033 | -0.228 | -0.119 |
| ELAVL1 | 1994 | 0.112 | -0.025 | 0.024 | CCDC52 | 152185 | -0.045 | -0.064 | 0.056 |
| PARP16 | 54956 | 0.106 | NA | -0.003 | COL1A1 | 1277 | -0.052 | -0.125 | -0.175 |
| DDX51 | 317781 | 0.101 | -0.022 | 0.129 | PSMD4 | 5710 | -0.054 | 0.014 | -0.109 |
| PRPF38B | 55119 | 0.099 | NA | 0.250 | MEIS1 | 4211 | -0.054 | -0.175 | -0.001 |
| CAPZB | 832 | 0.098 | -0.138 | -0.143 | VLDLR | 7436 | -0.058 | -0.227 | -0.074 |
| GBAS | 2631 | 0.094 | 0.179 | 0.225 | PTGIR | 5739 | -0.064 | 0.108 | -0.027 |
| ERH | 2079 | 0.092 | -0.186 | -0.200 | ERCC5 | 2073 | -0.068 | -0.001 | -0.050 |
| DNAJC8 | 22826 | 0.088 | -0.018 | 0.104 | COL6A2 | 1292 | -0.068 | -0.085 | -0.161 |
| GORASP2 | 26003 | 0.086 | 0.139 | 0.095 | TUBGCP3 | 10426 | -0.069 | 0.070 | -0.020 |
| DYNLL2 | 140735 | 0.086 | NA | NA | DAXX | 1616 | -0.071 | -0.248 | -0.277 |
| PRDX6 | 9588 | 0.085 | 0.191 | 0.148 | ADAM17 | 6868 | -0.071 | -0.093 | -0.148 |
| TBL3 | 10607 | 0.084 | -0.040 | 0.222 | PYGB | 5834 | -0.073 | 0.087 | 0.185 |
| HUWE1 | 10075 | 0.081 | -0.272 | -0.035 | UBE2L3 | 7332 | -0.077 | -0.134 | -0.076 |
| CSTF3 | 1479 | 0.078 | -0.034 | 0.064 | DHX36 | 170506 | -0.079 | NA | NA |
| UPK3B | 80761 | 0.078 | NA | -0.226 | ANKH | 56172 | -0.081 | NA | NA |
| TGFB2 | 7042 | 0.076 | -0.082 | -0.126 | PDCD4 | 27250 | -0.081 | -0.101 | -0.129 |
| FBXW5 | 54461 | 0.075 | NA | NA | PDGFRA | 5156 | -0.082 | 0.042 | -0.035 |
| TNPO2 | 30000 | 0.075 | -0.085 | 0.080 | TM9SF2 | 9375 | -0.083 | 0.171 | -0.173 |
| VRK3 | 51231 | 0.072 | NA | -0.078 | PCTK1 | 5127 | -0.087 | -0.311 | -0.196 |
| BEX1 | 55859 | 0.064 | NA | -0.290 | ZSCAN21 | 7589 | -0.089 | NA | NA |
| FGFR3 | 2261 | 0.064 | -0.257 | -0.084 | XPO1 | 7514 | -0.098 | -0.324 | 0.004 |
| REST | 5978 | 0.062 | NA | -0.032 | GJA4 | 2701 | -0.099 | 0.205 | -0.185 |
| ARL2BP | 23568 | 0.061 | 0.072 | 0.205 | GRID1 | 2894 | -0.106 | NA | NA |
| MYBBP1A | 10514 | 0.060 | NA | 0.006 | GPR12 | 2835 | -0.109 | 0.017 | -0.026 |
| SMARCA5 | 8467 | 0.058 | NA | -0.085 | KPNB1 | 3837 | -0.112 | NA | -0.135 |
| ENTPD4 | 9583 | 0.057 | NA | -0.050 | GARS | 2617 | -0.117 | -0.183 | -0.229 |
| NEK6 | 10783 | 0.052 | NA | NA | ARL6IP1 | 23204 | -0.117 | -0.118 | -0.148 |
| GPC3 | 2719 | 0.051 | 0.065 | 0.134 | STAB1 | 23166 | -0.125 | 0.056 | -0.178 |
| ANK1 | 286 | 0.046 | 0.026 | -0.065 | HAUS3 | 79441 | -0.128 | -0.131 | -0.063 |
| HMHA1 | 23526 | 0.046 | 0.343 | 0.236 | MTPN | 136319 | -0.133 | NA | NA |
| PIGK | 10026 | 0.044 | -0.113 | -0.172 | IL4R | 3566 | -0.144 | 0.219 | -0.081 |
| DSCR3 | 10311 | 0.039 | 0.045 | 0.008 | OGFR | 11054 | -0.148 | -0.183 | -0.217 |
| ACTG1 | 71 | 0.034 | -0.119 | 0.033 | ALDH1A1 | 216 | -0.152 | -0.202 | -0.275 |
| GLTP | 51228 | 0.032 | NA | 0.077 | EFNB2 | 1948 | -0.156 | 0.056 | -0.129 |
| EIF4G1 | 1981 | 0.027 | -0.241 | -0.033 | WARS | 7453 | -0.160 | 0.026 | -0.311 |
| HERC2 | 8924 | 0.025 | NA | 0.103 | GLUD1 | 2746 | -0.174 | -0.146 | -0.080 |
| LRRC8A | 56262 | 0.024 | NA | NA | F7 | 2155 | -0.189 | 0.093 | -0.172 |
| HADHB | 3032 | 0.024 | -0.104 | 0.015 | HSPA4 | 3308 | -0.192 | -0.089 | -0.034 |
| ASXL1 | 171023 | 0.024 | -0.177 | 0.138 | MORC4 | 79710 | -0.205 | NA | -0.042 |
| TCEB2 | 6923 | 0.023 | -0.121 | 0.205 | MPP1 | 4354 | -0.209 | -0.030 | -0.149 |
| TSPAN6 | 7105 | 0.019 | -0.079 | 0.064 | ITM2A | 9452 | -0.215 | 0.193 | -0.032 |
| LAPTM4A | 9741 | 0.019 | 0.198 | 0.159 | POSTN | 10631 | -0.217 | -0.134 | -0.255 |
| RAD50 | 10111 | 0.010 | 0.005 | 0.296 | HIATL1 | 84641 | -0.220 | NA | NA |
| METAP1 | 23173 | 0.008 | -0.087 | 0.169 | LOX | 4015 | -0.220 | NA | -0.301 |
| FZD2 | 2535 | 0.007 | 0.070 | -0.059 | TOMM70A | 9868 | -0.222 | -0.277 | -0.240 |
| SARS | 6301 | 0.007 | 0.002 | 0.168 | RB1 | 5925 | -0.234 | 0.017 | -0.174 |
| HRAS | 3265 | 0.007 | -0.377 | -0.205 | LAPTM5 | 7805 | -0.234 | 0.155 | -0.225 |
| NOTCH1 | 4851 | 0.006 | NA | 0.042 | EIF2A | 83939 | -0.254 | NA | NA |
| SFRS2 | 6427 | 0.004 | -0.113 | -0.027 | CTSB | 1508 | -0.259 | NA | -0.324 |
| SP3 | 6670 | 0.002 | -0.126 | 0.143 | G6PD | 2539 | -0.263 | NA | -0.252 |
| PFTK1 | 5218 | -0.005 | -0.198 | -0.192 | DDX47 | 51202 | -0.267 | NA | -0.261 |
| ODC1 | 4953 | -0.007 | 0.041 | 0.004 | TLE1 | 7088 | -0.269 | -0.301 | -0.232 |
| RPS11 | 6205 | -0.008 | -0.045 | 0.197 | COQ9 | 57017 | -0.269 | -0.240 | -0.084 |
| HRC | 3270 | -0.009 | -0.117 | 0.026 | OAZ1 | 4946 | -0.270 | -0.074 | 0.022 |
| CANT1 | 124583 | -0.012 | NA | -0.006 | UAP1 | 6675 | -0.271 | 0.100 | -0.060 |
| PLEC1 | 5339 | -0.013 | -0.226 | -0.060 | DPEP1 | 1800 | -0.272 | -0.228 | -0.197 |
| LIN7C | 55327 | -0.016 | NA | -0.084 | ANAPC5 | 51433 | -0.278 | -0.467 | -0.246 |
| PPTC7 | 160760 | -0.021 | NA | NA | GRM8 | 2918 | -0.279 | -0.084 | -0.048 |
| BAG1 | 573 | -0.022 | 0.051 | -0.007 | IDH2 | 3418 | -0.280 | -0.143 | -0.195 |
| NEDD8 | 4738 | -0.023 | 0.090 | -0.063 | DNPEP | 23549 | -0.282 | -0.198 | -0.190 |
| GPR37 | 2861 | -0.025 | 0.061 | -0.135 | CHORDC1 | 26973 | -0.290 | NA | -0.098 |
| GUK1 | 2987 | -0.026 | 0.095 | 0.046 | INPP1 | 3628 | -0.297 | -0.245 | -0.143 |
| ZKSCAN1 | 7586 | -0.032 | -0.354 | -0.267 | FIP1L1 | 81608 | -0.298 | NA | -0.183 |
| TCOF1 | 6949 | -0.034 | -0.231 | -0.021 | PSMD8 | 5714 | -0.300 | -0.227 | -0.180 |
| NUDT19 | 390916 | -0.040 | NA | NA | TGFBI | 7045 | -0.318 | -0.047 | -0.162 |
| NR6A1 | 2649 | -0.045 | NA | 0.012 | GSS | 2937 | -0.320 | -0.133 | -0.417 |
| CCDC52 | 152185 | -0.045 | -0.064 | 0.056 | DDX1 | 1653 | -0.323 | -0.312 | -0.142 |
| WNT10A | 80326 | -0.045 | NA | NA | BLMH | 642 | -0.338 | -0.325 | -0.189 |
| RAP2B | 5912 | -0.049 | 0.173 | -0.090 | PDHA1 | 5160 | -0.345 | NA | -0.113 |
| TUBB | 203068 | -0.050 | NA | -0.364 | ANLN | 54443 | -0.350 | NA | NA |
| HDGF2 | 84717 | -0.051 | NA | NA | IER3 | 8870 | -0.353 | -0.034 | -0.026 |
| EI24 | 9538 | -0.055 | -0.133 | -0.005 | ORC5L | 5001 | -0.358 | -0.108 | -0.170 |
| SLC39A6 | 25800 | -0.057 | -0.211 | -0.048 | SNAI2 | 6591 | -0.361 | -0.292 | -0.282 |
| ANKRD10 | 55608 | -0.057 | NA | 0.169 | IGFBP1 | 3484 | -0.361 | -0.151 | -0.284 |
| SLC25A1 | 6576 | -0.057 | -0.212 | -0.159 | CASP3 | 836 | -0.372 | -0.200 | -0.209 |
| STT3A | 3703 | -0.058 | 0.127 | -0.086 | FRG1 | 2483 | -0.410 | -0.251 | -0.223 |
| CHRAC1 | 54108 | -0.059 | NA | NA | COPZ1 | 22818 | -0.418 | NA | 0.089 |
| ACVR2A | 92 | -0.061 | -0.256 | -0.145 | ZC3H15 | 55854 | -0.422 | -0.315 | -0.270 |
| RABL4 | 11020 | -0.061 | -0.151 | -0.080 | FKBP4 | 2288 | -0.438 | -0.533 | -0.229 |
| BCL2 | 596 | -0.062 | -0.017 | 0.083 | CENPA | 1058 | -0.462 | -0.443 | -0.385 |
| SART3 | 9733 | -0.064 | -0.228 | 0.052 | NCAPH | 23397 | -0.471 | -0.173 | -0.352 |
| BCAP31 | 10134 | -0.064 | 0.044 | 0.145 | ADM | 133 | -0.493 | -0.415 | -0.413 |
| UPF3B | 65109 | -0.064 | NA | -0.031 | RAB10 | 10890 | -0.529 | NA | NA |
| PPP2R2B | 5521 | -0.064 | NA | -0.186 | IDH3A | 3419 | -0.541 | -0.152 | -0.219 |
| CHAF1B | 8208 | -0.065 | -0.238 | -0.325 | ITGA6 | 3655 | -0.641 | -0.539 | -0.404 |
| MMP14 | 4323 | -0.067 | -0.241 | -0.231 |  |  |  |  |  |
| ARF6 | 382 | -0.069 | 0.096 | -0.104 |  |  |  |  |  |
| EMP1 | 2012 | -0.069 | -0.028 | 0.071 |  |  |  |  |  |
| SLC35B1 | 10237 | -0.071 | 0.163 | -0.126 |  |  |  |  |  |
| DAG1 | 1605 | -0.076 | -0.042 | -0.149 |  |  |  |  |  |
| NSMCE1 | 197370 | -0.077 | NA | NA |  |  |  |  |  |
| IDE | 3416 | -0.077 | 0.049 | -0.117 |  |  |  |  |  |
| MAPK1 | 5594 | -0.078 | -0.012 | -0.146 |  |  |  |  |  |
| CCT3 | 7203 | -0.081 | -0.210 | -0.085 |  |  |  |  |  |
| MAP3K11 | 4296 | -0.081 | 0.015 | -0.155 |  |  |  |  |  |
| FTSJ3 | 117246 | -0.082 | NA | -0.122 |  |  |  |  |  |
| PDK3 | 5165 | -0.082 | -0.257 | -0.170 |  |  |  |  |  |
| NDUFAF1 | 51103 | -0.083 | -0.050 | 0.186 |  |  |  |  |  |
| NFYB | 4801 | -0.086 | -0.066 | 0.128 |  |  |  |  |  |
| HSD17B12 | 51144 | -0.089 | NA | -0.017 |  |  |  |  |  |
| AKR1A1 | 10327 | -0.093 | -0.030 | -0.188 |  |  |  |  |  |
| GNG12 | 55970 | -0.094 | 0.124 | 0.255 |  |  |  |  |  |
| PARD3 | 56288 | -0.094 | -0.108 | -0.037 |  |  |  |  |  |
| FURIN | 5045 | -0.094 | 0.193 | -0.107 |  |  |  |  |  |
| HELB | 92797 | -0.095 | NA | NA |  |  |  |  |  |
| FXN | 2395 | -0.096 | -0.138 | -0.201 |  |  |  |  |  |
| SOX2 | 6657 | -0.098 | -0.339 | -0.132 |  |  |  |  |  |
| DEK | 7913 | -0.098 | -0.144 | 0.009 |  |  |  |  |  |
| TEX261 | 113419 | -0.102 | -0.028 | 0.116 |  |  |  |  |  |
| PAWR | 5074 | -0.102 | -0.188 | -0.165 |  |  |  |  |  |
| TRNT1 | 51095 | -0.103 | NA | NA |  |  |  |  |  |
| ABCE1 | 6059 | -0.109 | -0.078 | -0.138 |  |  |  |  |  |
| PRPF19 | 27339 | -0.110 | -0.335 | -0.308 |  |  |  |  |  |
| ACAD9 | 28976 | -0.112 | NA | NA |  |  |  |  |  |
| HAX1 | 10456 | -0.113 | 0.141 | 0.008 |  |  |  |  |  |
| PXN | 5829 | -0.114 | NA | -0.198 |  |  |  |  |  |
| ARPC1B | 10095 | -0.121 | 0.126 | -0.148 |  |  |  |  |  |
| PKM2 | 5315 | -0.121 | -0.418 | -0.327 |  |  |  |  |  |
| MSH2 | 4436 | -0.123 | -0.244 | -0.045 |  |  |  |  |  |
| CLK3 | 1198 | -0.124 | NA | -0.126 |  |  |  |  |  |
| CCDC80 | 151887 | -0.124 | NA | NA |  |  |  |  |  |
| RNASEH2B | 79621 | -0.124 | -0.101 | 0.078 |  |  |  |  |  |
| HN1 | 51155 | -0.125 | NA | -0.176 |  |  |  |  |  |
| VPS35 | 55737 | -0.125 | NA | -0.047 |  |  |  |  |  |
| ALK | 238 | -0.126 | 0.079 | -0.118 |  |  |  |  |  |
| CCKAR | 886 | -0.130 | -0.013 | -0.128 |  |  |  |  |  |
| ARL4C | 10123 | -0.132 | -0.013 | -0.167 |  |  |  |  |  |
| DAP3 | 7818 | -0.134 | 0.141 | 0.090 |  |  |  |  |  |
| SEMA4D | 10507 | -0.138 | NA | -0.167 |  |  |  |  |  |
| ORC2L | 4999 | -0.141 | -0.229 | -0.115 |  |  |  |  |  |
| ATF1 | 466 | -0.144 | 0.024 | 0.097 |  |  |  |  |  |
| VBP1 | 7411 | -0.144 | -0.114 | 0.090 |  |  |  |  |  |
| EIF2B5 | 8893 | -0.145 | -0.373 | -0.037 |  |  |  |  |  |
| RPIA | 22934 | -0.146 | -0.039 | 0.103 |  |  |  |  |  |
| TES | 26136 | -0.148 | 0.110 | -0.138 |  |  |  |  |  |
| EIF5 | 1983 | -0.153 | -0.251 | -0.223 |  |  |  |  |  |
| RBBP7 | 5931 | -0.155 | NA | -0.170 |  |  |  |  |  |
| EFNB2 | 1948 | -0.156 | 0.056 | -0.129 |  |  |  |  |  |
| PRDX1 | 5052 | -0.158 | -0.079 | -0.247 |  |  |  |  |  |
| WARS | 7453 | -0.160 | 0.026 | -0.311 |  |  |  |  |  |
| SLC38A2 | 54407 | -0.160 | NA | -0.222 |  |  |  |  |  |
| WRN | 7486 | -0.162 | -0.144 | 0.004 |  |  |  |  |  |
| CLCN3 | 1182 | -0.163 | NA | -0.016 |  |  |  |  |  |
| FDX1 | 2230 | -0.165 | -0.042 | -0.157 |  |  |  |  |  |
| RCL1 | 10171 | -0.165 | NA | -0.062 |  |  |  |  |  |
| ILVBL | 10994 | -0.166 | -0.214 | 0.046 |  |  |  |  |  |
| SFXN1 | 94081 | -0.169 | NA | -0.075 |  |  |  |  |  |
| PSMD9 | 5715 | -0.169 | -0.246 | -0.077 |  |  |  |  |  |
| ORMDL2 | 29095 | -0.175 | NA | -0.141 |  |  |  |  |  |
| HMOX1 | 3162 | -0.176 | 0.044 | -0.303 |  |  |  |  |  |
| DAD1 | 1603 | -0.180 | 0.078 | -0.233 |  |  |  |  |  |
| AP3B1 | 8546 | -0.181 | 0.128 | -0.049 |  |  |  |  |  |
| TCF4 | 6925 | -0.182 | -0.057 | -0.091 |  |  |  |  |  |
| SLC6A13 | 6540 | -0.183 | 0.113 | -0.035 |  |  |  |  |  |
| BPGM | 669 | -0.184 | -0.145 | -0.124 |  |  |  |  |  |
| MAP7 | 9053 | -0.186 | -0.233 | 0.042 |  |  |  |  |  |
| IMMP1L | 196294 | -0.189 | NA | NA |  |  |  |  |  |
| MRPL16 | 54948 | -0.189 | NA | -0.116 |  |  |  |  |  |
| CDKN2C | 1031 | -0.192 | -0.159 | -0.134 |  |  |  |  |  |
| SLC3A2 | 6520 | -0.192 | -0.333 | -0.186 |  |  |  |  |  |
| ATP5G2 | 517 | -0.192 | NA | -0.049 |  |  |  |  |  |
| SSBP1 | 6742 | -0.192 | -0.283 | -0.260 |  |  |  |  |  |
| BAG2 | 9532 | -0.194 | -0.143 | -0.206 |  |  |  |  |  |
| TXNL1 | 9352 | -0.196 | -0.149 | -0.224 |  |  |  |  |  |
| GDI2 | 2665 | -0.210 | -0.092 | -0.137 |  |  |  |  |  |
| LIMS1 | 3987 | -0.212 | -0.162 | -0.232 |  |  |  |  |  |
| FLT3 | 2322 | -0.213 | -0.039 | -0.117 |  |  |  |  |  |
| COMMD2 | 51122 | -0.215 | NA | NA |  |  |  |  |  |
| PTPN9 | 5780 | -0.216 | -0.252 | -0.269 |  |  |  |  |  |
| ICT1 | 3396 | -0.217 | 0.034 | -0.041 |  |  |  |  |  |
| NOL7 | 51406 | -0.220 | NA | -0.142 |  |  |  |  |  |
| RAB7A | 7879 | -0.222 | NA | -0.117 |  |  |  |  |  |
| ACTN1 | 87 | -0.228 | -0.403 | -0.390 |  |  |  |  |  |
| SEL1L | 6400 | -0.234 | -0.105 | -0.324 |  |  |  |  |  |
| LMNB1 | 4001 | -0.235 | -0.335 | -0.098 |  |  |  |  |  |
| MAFG | 4097 | -0.236 | NA | -0.149 |  |  |  |  |  |
| BACH1 | 571 | -0.240 | -0.023 | -0.269 |  |  |  |  |  |
| LAS1L | 81887 | -0.247 | -0.165 | 0.134 |  |  |  |  |  |
| BNIP2 | 663 | -0.250 | -0.065 | -0.119 |  |  |  |  |  |
| CSTF2T | 23283 | -0.252 | 0.028 | 0.023 |  |  |  |  |  |
| MRPS18A | 55168 | -0.254 | NA | -0.096 |  |  |  |  |  |
| GPC1 | 2817 | -0.254 | -0.413 | -0.206 |  |  |  |  |  |
| RAE1 | 8480 | -0.257 | -0.366 | -0.249 |  |  |  |  |  |
| MCM2 | 4171 | -0.258 | -0.399 | -0.332 |  |  |  |  |  |
| COPB2 | 9276 | -0.260 | -0.088 | -0.273 |  |  |  |  |  |
| CACYBP | 27101 | -0.262 | 0.077 | 0.052 |  |  |  |  |  |
| DNAJC10 | 54431 | -0.263 | NA | -0.428 |  |  |  |  |  |
| SUCLA2 | 8803 | -0.267 | -0.171 | -0.088 |  |  |  |  |  |
| DDX47 | 51202 | -0.267 | NA | -0.261 |  |  |  |  |  |
| B2M | 567 | -0.270 | 0.061 | -0.135 |  |  |  |  |  |
| GMNN | 51053 | -0.274 | NA | -0.300 |  |  |  |  |  |
| H2AFX | 3014 | -0.280 | -0.326 | -0.291 |  |  |  |  |  |
| ARIH1 | 25820 | -0.285 | NA | -0.169 |  |  |  |  |  |
| ASNS | 440 | -0.286 | -0.266 | -0.176 |  |  |  |  |  |
| CPSF3 | 51692 | -0.287 | NA | NA |  |  |  |  |  |
| MRPL30 | 51263 | -0.294 | NA | NA |  |  |  |  |  |
| PSMC2 | 5701 | -0.294 | -0.145 | -0.297 |  |  |  |  |  |
| LAMB1 | 3912 | -0.295 | -0.239 | -0.436 |  |  |  |  |  |
| INPP1 | 3628 | -0.297 | -0.245 | -0.143 |  |  |  |  |  |
| DCTN5 | 84516 | -0.297 | 0.053 | -0.130 |  |  |  |  |  |
| CD27 | 939 | -0.299 | 0.054 | -0.146 |  |  |  |  |  |
| PSMD8 | 5714 | -0.300 | -0.227 | -0.180 |  |  |  |  |  |
| PIK3CA | 5290 | -0.307 | NA | -0.085 |  |  |  |  |  |
| KIF11 | 3832 | -0.309 | -0.343 | -0.302 |  |  |  |  |  |
| LBP | 3929 | -0.317 | -0.089 | -0.236 |  |  |  |  |  |
| MRPS17 | 51373 | -0.336 | NA | -0.206 |  |  |  |  |  |
| PFKM | 5213 | -0.348 | -0.361 | 0.173 |  |  |  |  |  |
| PTGES3 | 10728 | -0.349 | -0.221 | -0.123 |  |  |  |  |  |
| ANLN | 54443 | -0.350 | NA | NA |  |  |  |  |  |
| FKBP1A | 2280 | -0.350 | -0.021 | -0.217 |  |  |  |  |  |
| MRPL48 | 51642 | -0.353 | NA | -0.191 |  |  |  |  |  |
| CNBP | 7555 | -0.362 | -0.204 | -0.061 |  |  |  |  |  |
| DDX21 | 9188 | -0.370 | -0.082 | -0.148 |  |  |  |  |  |
| NDUFA8 | 4702 | -0.370 | NA | -0.208 |  |  |  |  |  |
| DNAJC1 | 64215 | -0.371 | NA | -0.454 |  |  |  |  |  |
| SMC2 | 10592 | -0.379 | -0.157 | -0.315 |  |  |  |  |  |
| KIF4A | 24137 | -0.381 | NA | -0.378 |  |  |  |  |  |
| PHLDB2 | 90102 | -0.384 | NA | NA |  |  |  |  |  |
| NEDD4 | 4734 | -0.391 | -0.088 | -0.133 |  |  |  |  |  |
| NDUFS3 | 4722 | -0.395 | -0.335 | -0.202 |  |  |  |  |  |
| CCNB1 | 891 | -0.403 | -0.297 | -0.328 |  |  |  |  |  |
| H2AFJ | 55766 | -0.406 | NA | -0.018 |  |  |  |  |  |
| UBE2C | 11065 | -0.409 | -0.276 | -0.201 |  |  |  |  |  |
| ERO1L | 30001 | -0.410 | NA | -0.325 |  |  |  |  |  |
| R3HDM1 | 23518 | -0.415 | -0.450 | -0.391 |  |  |  |  |  |
| HSD17B10 | 3028 | -0.418 | -0.428 | -0.317 |  |  |  |  |  |
| CDC2 | 983 | -0.424 | -0.313 | -0.356 |  |  |  |  |  |
| MYO1E | 4643 | -0.427 | -0.072 | -0.229 |  |  |  |  |  |
| H2AFZ | 3015 | -0.439 | -0.304 | -0.359 |  |  |  |  |  |
| PSMA5 | 5686 | -0.470 | -0.159 | -0.402 |  |  |  |  |  |
| NCAPH | 23397 | -0.471 | -0.173 | -0.352 |  |  |  |  |  |
| CYB5R4 | 51167 | -0.481 | NA | -0.208 |  |  |  |  |  |
| DNAJB11 | 51726 | -0.482 | NA | NA |  |  |  |  |  |
| CCNB2 | 9133 | -0.484 | -0.433 | -0.399 |  |  |  |  |  |
| ATP5C1 | 509 | -0.511 | -0.276 | -0.326 |  |  |  |  |  |
| TFF1 | 7031 | -0.518 | -0.122 | -0.416 |  |  |  |  |  |
| RACGAP1 | 29127 | -0.528 | NA | -0.351 |  |  |  |  |  |
| ADA | 100 | -0.534 | -0.421 | -0.409 |  |  |  |  |  |
| COX7A2 | 1347 | -0.591 | -0.368 | -0.195 |  |  |  |  |  |
| CTSL2 | 1515 | -0.610 | -0.415 | -0.547 |  |  |  |  |  |
| UCK2 | 7371 | -0.642 | -0.639 | -0.650 |  |  |  |  |  |
