# Supplementary material for: Genome-Wide Analyses of Nkx2-1 Binding to Transcriptional Target Genes Uncover Novel Regulatory Patterns Conserved in Lung Development and Tumors
Source: PLoS One. 2012 Jan 5;7(1):e29907. doi: 10.1371/journal.pone.0029907 (PMC3252372; doi:10.1371/journal.pone.0029907)
Supplement: Table S3 — Genes bound and regulated by Nkx2-1 in human fetal lung epithelial cells. (DOC) [file pone.0029907.s008.doc]

| *Table S3. Genes bound and regulated by Nkx2-1 in human fetal lung epithelial cells* | | |
| --- | --- | --- |
| **E11.5** | **E19.5** | **Both** |
| Abca3 | Abca8a | Abcc4 |
| Abca8b | Abcc6 | Aldh3b1 |
| Abcb4 | Edn3 | Aqp4 |
| Cacna2d2 | Fbln5 | Atp1b1 |
| Fgfr2 | Flot1 | Calca |
| Mlph | Gas1 | Cit |
| Pecam1 | Il18r1 | Cldn18 |
|  | Ltbr | Clic5 |
|  | Papss2 | Cyb5 |
|  | Pdk4 | Gpx3 |
|  | Pgc | Gusb |
|  | Sftpc | Hif3a |
|  | Slc1a1 | Igf2 |
|  | Tm7sf2 | Il1rl1 |
|  | Wfdc2 | Impa2 |
|  |  | Lpl |
|  |  | Ncor2 |
|  |  | Nr4a2 |
|  |  | Ptp4a1 |
|  |  | Rapgef5 |
|  |  | Sftpa1 |
|  |  | Sftpb |
|  |  | Sh3bp5 |
|  |  | Slc1a1 |
|  |  | Slc39a8 |
|  |  | Socs3 |
|  |  | Titf1 |
|  |  | Tmprss2 |
|  |  | Tst |
|  |  | Wif1 |
