# Supplementary material for: Genome-Wide Analyses of Nkx2-1 Binding to Transcriptional Target Genes Uncover Novel Regulatory Patterns Conserved in Lung Development and Tumors
Source: PLoS One. 2012 Jan 5;7(1):e29907. doi: 10.1371/journal.pone.0029907 (PMC3252372; doi:10.1371/journal.pone.0029907)
Supplement: Table S4 — (a) E11.5 overrepresented biological processes identified by EASE analysis p<0.05. (b) E19.5 overrepresented biological processes identified by EASE analysis (p<0.05). (DOC) [file pone.0029907.s009.doc]

| *Table S4a. E11.5 overrepresented biological processes identified by EASE analysis p<0.05* | | | | | | | | |
| --- | --- | --- | --- | --- | --- | --- | --- | --- |
| *Term* | *Count* | *%* | *PValue* | *List Total* | *Pop Hits* | *Pop Total* | *Fold Enrichment* |  |
| GO:0007154~cell communication | 335 | 26.25% | 4.01E-04 | 1006 | 4270 | 14977 | 1.168002524 |  |
| GO:0007165~signal transduction | 314 | 24.61% | 8.11E-04 | 1006 | 4012 | 14977 | 1.165186845 |  |
| **GO:0008284~positive regulation of cell proliferation** | 25 | 1.96% | 0.0037735 | 1006 | 199 | 14977 | 1.870310799 |  |
| GO:0006464~protein modification process | 137 | 10.74% | 0.0053806 | 1006 | 1648 | 14977 | 1.237628235 |  |
| GO:0006464~protein modification process | 137 | 10.74% | 0.0053806 | 1006 | 1648 | 14977 | 1.237628235 |  |
| **GO:0007265~Ras protein signal transduction** | 24 | 1.88% | 0.0055631 | 1006 | 194 | 14977 | 1.841774098 |  |
| **GO:0042127~regulation of cell proliferation** | 39 | 3.06% | 0.006971 | 1006 | 374 | 14977 | 1.552457979 |  |
| GO:0043524~negative regulation of neuron apoptosis | 7 | 0.55% | 0.0094547 | 1006 | 28 | 14977 | 3.721918489 |  |
| GO:0043412~biopolymer modification | 140 | 10.97% | 0.0096747 | 1006 | 1717 | 14977 | 1.213904691 |  |
| GO:0043412~biopolymer modification | 140 | 10.97% | 0.0096747 | 1006 | 1717 | 14977 | 1.213904691 |  |
| GO:0051246~regulation of protein metabolic process | 31 | 2.43% | 0.010002 | 1006 | 286 | 14977 | 1.613698925 |  |
| GO:0043687~post-translational protein modification | 119 | 9.33% | 0.0107721 | 1006 | 1435 | 14977 | 1.234587596 |  |
| GO:0043687~post-translational protein modification | 119 | 9.33% | 0.0107721 | 1006 | 1435 | 14977 | 1.234587596 |  |
| GO:0042129~regulation of T cell proliferation | 9 | 0.71% | 0.0121182 | 1006 | 47 | 14977 | 2.850831183 |  |
| GO:0006468~protein amino acid phosphorylation | 60 | 4.70% | 0.0143286 | 1006 | 660 | 14977 | 1.353424905 |  |
| GO:0006796~phosphate metabolic process | 79 | 6.19% | 0.0153481 | 1006 | 912 | 14977 | 1.289612108 |  |
| GO:0006793~phosphorus metabolic process | 79 | 6.19% | 0.0153481 | 1006 | 912 | 14977 | 1.289612108 |  |
| GO:0016126~sterol biosynthetic process | 7 | 0.55% | 0.0155745 | 1006 | 31 | 14977 | 3.361732829 |  |
| GO:0042327~positive regulation of phosphorylation | 7 | 0.55% | 0.0181104 | 1006 | 32 | 14977 | 3.256678678 |  |
| GO:0007229~integrin-mediated signaling pathway | 13 | 1.02% | 0.0182769 | 1006 | 91 | 14977 | 2.126810565 |  |
| GO:0006695~cholesterol biosynthetic process | 6 | 0.47% | 0.0196416 | 1006 | 24 | 14977 | 3.721918489 |  |
| GO:0045937~positive regulation of phosphate metabolic process | 7 | 0.55% | 0.0209151 | 1006 | 33 | 14977 | 3.157991445 |  |
| GO:0016310~phosphorylation | 67 | 5.25% | 0.0211782 | 1006 | 766 | 14977 | 1.302185581 |  |
| GO:0018346~protein amino acid prenylation | 4 | 0.31% | 0.0253143 | 1006 | 10 | 14977 | 5.955069583 |  |
| GO:0043523~regulation of neuron apoptosis | 8 | 0.63% | 0.0260209 | 1006 | 44 | 14977 | 2.70684981 |  |
| GO:0043066~negative regulation of apoptosis | 21 | 1.65% | 0.0288571 | 1006 | 189 | 14977 | 1.654185995 |  |
| GO:0018193~peptidyl-amino acid modification | 15 | 1.18% | 0.0320191 | 1006 | 121 | 14977 | 1.845579416 |  |
| GO:0043069~negative regulation of programmed cell death | 21 | 1.65% | 0.0333524 | 1006 | 192 | 14977 | 1.628339339 |  |
| GO:0042094~interleukin-2 biosynthetic process | 5 | 0.39% | 0.0347616 | 1006 | 19 | 14977 | 3.917808936 |  |
| GO:0042094~interleukin-2 biosynthetic process | 5 | 0.39% | 0.0347616 | 1006 | 19 | 14977 | 3.917808936 |  |
| GO:0050731~positive regulation of peptidyl-tyrosine phosphorylation | 6 | 0.47% | 0.0364395 | 1006 | 28 | 14977 | 3.190215848 |  |
| GO:0009966~regulation of signal transduction | 41 | 3.21% | 0.0403923 | 1006 | 449 | 14977 | 1.359453524 |  |
| GO:0032623~interleukin-2 production | 5 | 0.39% | 0.0412153 | 1006 | 20 | 14977 | 3.721918489 |  |
| GO:0042168~heme metabolic process | 5 | 0.39% | 0.0412153 | 1006 | 20 | 14977 | 3.721918489 |  |
| GO:0032623~interleukin-2 production | 5 | 0.39% | 0.0412153 | 1006 | 20 | 14977 | 3.721918489 |  |
| GO:0042403~thyroid hormone metabolic process | 4 | 0.31% | 0.0419878 | 1006 | 12 | 14977 | 4.962557985 |  |
| GO:0050670~regulation of lymphocyte proliferation | 9 | 0.71% | 0.0461614 | 1006 | 60 | 14977 | 2.233151093 |  |
| GO:0032944~regulation of mononuclear cell proliferation | 9 | 0.71% | 0.0461614 | 1006 | 60 | 14977 | 2.233151093 |  |
| GO:0044260~cellular macromolecule metabolic process | 235 | 18.42% | 0.0464833 | 1006 | 3165 | 14977 | 1.105403911 |  |
| GO:0050769~positive regulation of neurogenesis | 5 | 0.39% | 0.0482999 | 1006 | 21 | 14977 | 3.544684275 |  |

| *Table S4b. E19.5 overrepresented biological processes identified by EASE analysis (p<0.05)* | | | | | | | |
| --- | --- | --- | --- | --- | --- | --- | --- |
| *Term* | *Count* | *%* | *PValue* | *List Total* | *Pop Hits* | *Pop Total* | *Fold Enrichment* |
| **GO:0006811~ion transport** | 81 | 6.40% | 9.77E-05 | 1005 | 784 | 14977 | 1.5396703 |
| GO:0006812~cation transport | 58 | 4.58% | 4.23E-04 | 1005 | 540 | 14977 | 1.6006376 |
| GO:0030001~metal ion transport | 49 | 3.87% | 5.19E-04 | 1005 | 438 | 14977 | 1.6671733 |
| GO:0006817~phosphate transport | 15 | 1.18% | 0.0010232 | 1005 | 82 | 14977 | 2.7260648 |
| GO:0015698~inorganic anion transport | 23 | 1.82% | 0.0011083 | 1005 | 161 | 14977 | 2.1289268 |
| GO:0051239~regulation of multicellular organismal process | 36 | 2.84% | 0.0016127 | 1005 | 309 | 14977 | 1.7362121 |
| GO:0006937~regulation of muscle contraction | 9 | 0.71% | 0.0022229 | 1005 | 36 | 14977 | 3.7256219 |
| GO:0006820~anion transport | 24 | 1.90% | 0.0045445 | 1005 | 191 | 14977 | 1.8725639 |
| GO:0009611~response to wounding | 35 | 2.76% | 0.0046692 | 1005 | 318 | 14977 | 1.6402109 |
| GO:0006954~inflammatory response | 27 | 2.13% | 0.0052242 | 1005 | 227 | 14977 | 1.7725426 |
| GO:0051240~positive regulation of multicellular organismal process | 18 | 1.42% | 0.0070733 | 1005 | 132 | 14977 | 2.0321574 |
| GO:0015672~monovalent inorganic cation transport | 36 | 2.84% | 0.007526 | 1005 | 340 | 14977 | 1.5779104 |
| GO:0009593~detection of chemical stimulus | 7 | 0.55% | 0.0078285 | 1005 | 27 | 14977 | 3.8636079 |
| GO:0007270~nerve-nerve synaptic transmission | 7 | 0.55% | 0.0078285 | 1005 | 27 | 14977 | 3.8636079 |
| GO:0051606~detection of stimulus | 13 | 1.03% | 0.0130081 | 1005 | 87 | 14977 | 2.2268085 |
| GO:0019752~carboxylic acid metabolic process | 48 | 3.79% | 0.0142429 | 1005 | 506 | 14977 | 1.4136747 |
| GO:0006082~organic acid metabolic process | 48 | 3.79% | 0.0149145 | 1005 | 507 | 14977 | 1.4108864 |
| GO:0050906~detection of stimulus during sensory perception | 10 | 0.79% | 0.0196686 | 1005 | 61 | 14977 | 2.4430307 |
| GO:0006813~potassium ion transport | 20 | 1.58% | 0.0206515 | 1005 | 171 | 14977 | 1.742981 |
| GO:0009605~response to external stimulus | 46 | 3.63% | 0.0233973 | 1005 | 496 | 14977 | 1.3820855 |
| GO:0006814~sodium ion transport | 16 | 1.26% | 0.0243042 | 1005 | 128 | 14977 | 1.8628109 |
| GO:0022409~positive regulation of cell-cell adhesion | 3 | 0.24% | 0.024595 | 1005 | 4 | 14977 | 11.176866 |
| GO:0006629~lipid metabolic process | 58 | 4.58% | 0.0258506 | 1005 | 656 | 14977 | 1.317598 |
| GO:0006940~regulation of smooth muscle contraction | 5 | 0.39% | 0.0288439 | 1005 | 18 | 14977 | 4.1395799 |
| GO:0001580~detection of chemical stimulus during sensory perception of bitter taste | 5 | 0.39% | 0.0288439 | 1005 | 18 | 14977 | 4.1395799 |
| GO:0050912~detection of chemical stimulus during sensory perception of taste | 5 | 0.39% | 0.0288439 | 1005 | 18 | 14977 | 4.1395799 |
| GO:0048593~camera-type eye morphogenesis | 5 | 0.39% | 0.0288439 | 1005 | 18 | 14977 | 4.1395799 |
| GO:0007613~memory | 5 | 0.39% | 0.0288439 | 1005 | 18 | 14977 | 4.1395799 |
| GO:0050907~detection of chemical stimulus during sensory perception | 5 | 0.39% | 0.0410869 | 1005 | 20 | 14977 | 3.7256219 |
| GO:0050913~sensory perception of bitter taste | 5 | 0.39% | 0.0410869 | 1005 | 20 | 14977 | 3.7256219 |
| GO:0003012~muscle system process | 12 | 0.95% | 0.0433956 | 1005 | 92 | 14977 | 1.9438027 |
| GO:0006936~muscle contraction | 12 | 0.95% | 0.0433956 | 1005 | 92 | 14977 | 1.9438027 |
| GO:0003018~vascular process in circulatory system | 7 | 0.55% | 0.0437604 | 1005 | 39 | 14977 | 2.6748055 |
| GO:0050880~regulation of blood vessel size | 7 | 0.55% | 0.0437604 | 1005 | 39 | 14977 | 2.6748055 |
| GO:0035150~regulation of tube size | 7 | 0.55% | 0.0437604 | 1005 | 39 | 14977 | 2.6748055 |
| GO:0002526~acute inflammatory response | 11 | 0.87% | 0.046941 | 1005 | 82 | 14977 | 1.9991142 |
| GO:0002526~acute inflammatory response | 11 | 0.87% | 0.046941 | 1005 | 82 | 14977 | 1.9991142 |
| GO:0006950~response to stress | 67 | 5.29% | 0.0963137 | 1005 | 839 | 14977 | 1.1900675 |
