# Supplementary material for: Genome-Wide Analyses of Nkx2-1 Binding to Transcriptional Target Genes Uncover Novel Regulatory Patterns Conserved in Lung Development and Tumors
Source: PLoS One. 2012 Jan 5;7(1):e29907. doi: 10.1371/journal.pone.0029907 (PMC3252372; doi:10.1371/journal.pone.0029907)
Supplement: Table S5 — Overrepresented canonical pathways identified by Ingenuity Pathway Analysis Software. (DOC) [file pone.0029907.s010.doc]

|  |  |  |
| --- | --- | --- |
| ***Table S5. Overrepresented canonical pathways identified by Ingenuity Pathway Analysis Software*** | | |
| ***E11.5 and E19.5 Common Pathways*** | ***E11.5 p-value*** | ***E19.5 p-value*** |
| Thrombin Signaling | 0.0019 | 0.0331 |
| mTOR Signaling | 0.0026 | 0.0068 |
| Germ Cell-Sertoli Cell Junction Signaling | 0.0028 | 0.0295 |
| NRF2-mediated Oxidative Stress Response | 0.0060 | 0.0263 |
| Erythropoietin Signaling | 0.0074 | 0.0224 |
| Production of Nitric Oxide and Reactive Oxygen Species in Macrophages | 0.0078 | 0.0178 |
| Molecular Mechanisms of Cancer | 0.0126 | 0.0380 |
| Growth Hormone Signaling | 0.0174 | 0.0186 |
| G-Protein Coupled Receptor Signaling | 0.0186 | 0.0006 |
| HGF Signaling | 0.0195 | 0.0468 |
| Sphingosine-1-phosphate Signaling | 0.0263 | 0.0282 |
|  |  |  |
| ***E11.5 Unique Pathways*** | ***p-value*** |  |
| Type II Diabetes Mellitus Signaling | 0.0000 |  |
| TR/RXR Activation | 0.0003 |  |
| Cardiac Hypertrophy Signaling | 0.0004 |  |
| AMPK Signaling | 0.0006 |  |
| Reelin Signaling in Neurons | 0.0008 |  |
| CXCR4 Signaling | 0.0014 |  |
| Methane Metabolism | 0.0025 |  |
| Ceramide Signaling | 0.0035 |  |
| IL-8 Signaling | 0.0043 |  |
| IL-1 Signaling | 0.0048 |  |
| Integrin Signaling | 0.0049 |  |
| Role of NFAT in Cardiac Hypertrophy | 0.0050 |  |
| Fcγ Receptor-mediated Phagocytosis in Macrophages and Monocytes | 0.0052 |  |
| Fatty Acid Biosynthesis | 0.0052 |  |
| Phenylalanine Metabolism | 0.0068 |  |
| Clathrin-mediated Endocytosis Signaling | 0.0095 |  |
| RANK Signaling in Osteoclasts | 0.0095 |  |
| p70S6K Signaling | 0.0095 |  |
| Endothelin-1 Signaling | 0.0095 |  |
| Phospholipase C Signaling | 0.0098 |  |
| Neuregulin Signaling | 0.0102 |  |
| Inositol Phosphate Metabolism | 0.0115 |  |
| Antiproliferative Role of Somatostatin Receptor 2 | 0.0126 |  |
| Role of Osteoblasts, Osteoclasts and Chondrocytes in Rheumatoid Arthritis | 0.0129 |  |
| HMGB1 Signaling | 0.0132 |  |
| Biosynthesis of Steroids | 0.0141 |  |
| Thrombopoietin Signaling | 0.0141 |  |
| fMLP Signaling in Neutrophils | 0.0155 |  |
| Axonal Guidance Signaling | 0.0158 |  |
| G Protein Signaling Mediated by Tubby | 0.0162 |  |
| Glioma Signaling | 0.0170 |  |
| Caveolar-mediated Endocytosis Signaling | 0.0182 |  |
| G-Protein Coupled Receptor Signaling | 0.0186 |  |
| HER-2 Signaling in Breast Cancer | 0.0195 |  |
| Glioma Invasiveness Signaling | 0.0200 |  |
| Acute Phase Response Signaling | 0.0204 |  |
| Glioblastoma Multiforme Signaling | 0.0214 |  |
| N-Glycan Biosynthesis | 0.0219 |  |
| GNRH Signaling | 0.0224 |  |
| Cholecystokinin/Gastrin-mediated Signaling | 0.0224 |  |
| CCR5 Signaling in Macrophages | 0.0275 |  |
| HIF1α Signaling | 0.0282 |  |
| G Beta Gamma Signaling | 0.0288 |  |
| Pancreatic Adenocarcinoma Signaling | 0.0295 |  |
| FcγRIIB Signaling in B Lymphocytes | 0.0309 |  |
| RAR Activation | 0.0331 |  |
| Chronic Myeloid Leukemia Signaling | 0.0355 |  |
| Colorectal Cancer Metastasis Signaling | 0.0355 |  |
| Aminosugars Metabolism | 0.0380 |  |
| Fc Epsilon RI Signaling | 0.0380 |  |
| CCR3 Signaling in Eosinophils | 0.0407 |  |
| CD27 Signaling in Lymphocytes | 0.0407 |  |
| Regulation of Actin-based Motility by Rho | 0.0427 |  |
| VEGF Signaling | 0.0427 |  |
| Maturity Onset Diabetes of Young (MODY) Signaling | 0.0468 |  |
| Macropinocytosis Signaling | 0.0490 |  |
|  |  |  |
| ***E19.5 Unique Pathways*** | ***p-value*** |  |
| Renin-Angiotensin Signaling | 0.0060 |  |
| Coagulation System | 0.0083 |  |
| Aldosterone Signaling in Epithelial Cells | 0.0107 |  |
| Pantothenate and CoA Biosynthesis | 0.0138 |  |
| NF-kB Activation by Viruses | 0.0162 |  |
| Regulation of eIF4 and p70S6K Signaling | 0.0170 |  |
| Leptin Signaling in Obesity | 0.0178 |  |
| Neuropathic Pain Signaling In Dorsal Horn Neurons | 0.0182 |  |
| Leukocyte Extravasation Signaling | 0.0288 |  |
| PPARα/RXRα Activation | 0.0363 |  |
| CREB Signaling in Neurons | 0.0380 |  |
| Interferon Signaling | 0.0407 |  |
| Aminoacyl-tRNA Biosynthesis | 0.0407 |  |
| RhoA Signaling | 0.0417 |  |
| Circadian Rhythm Signaling | 0.0457 |  |
|  |  |  |
