# Supplementary material for: Genome-Wide Analyses of Nkx2-1 Binding to Transcriptional Target Genes Uncover Novel Regulatory Patterns Conserved in Lung Development and Tumors
Source: PLoS One. 2012 Jan 5;7(1):e29907. doi: 10.1371/journal.pone.0029907 (PMC3252372; doi:10.1371/journal.pone.0029907)
Supplement: Table S6 — Nkx2-1 target genes genes included in Cancer pathways identified by IPA. (DOC) [file pone.0029907.s011.doc]

| *Table S6. Nkx2-1 target genes genes included in Cancer pathways identified by IPA* | | | |
| --- | --- | --- | --- |
|
| ***Pathway: Mechanisms of Cancer*** | | ***Pathway: HGF Signalling*** | |
| ***E11.5*** | ***E19.5*** | ***E11.5*** | ***E19.5*** |
| ADCY5 | ADCY7 | HRAS | IL6 |
| ADCY8 | ADCY8 | MAP2K7 | MAP3K2 |
| ARHGEF11 | ARHGEF11 | MAP3K11 | MAP3K8 |
| BCL2 | ATM | MAP3K13 | MET |
| BIRC3 | BMP7 | MAP3K7 | PIK3C2G |
| BMP3 | CASP3 | MAP3K9 | PIK3R5 |
| CDKN2C | CDC25A | MAPK10 | PLCG2 |
| E2F3 | CTNNA2 | PIK3CA | PRKCH |
| GAB2 | DAXX | PIK3R1 | PRKCZ |
| GNAS | GNA11 | PRKCH | RAC1 |
| HRAS | GNAL | PRKCQ | RAPGEF1 |
| JAK2 | IRS1 | PXN |  |
| MAP3K7 | JAK2 |  |  |
| MAPK10 | MAX |  |  |
| MDM2 | NCSTN |  |  |
| MYC | NFKBIA |  |  |
| NFKB2 | PIAS2 |  |  |
| NLK | PIK3C2G |  |  |
| NOTCH1 | PIK3R5 |  |  |
| PIK3CA | PRKAR2A |  |  |
| PIK3R1 | PRKCH |  |  |
| PRKCH | PRKCZ |  |  |
| PRKCQ | PSEN2 |  |  |
| RABIF | PTCH2 |  |  |
| RAP2B | RAC1 |  |  |
| RASGRF2 | RAPGEF1 |  |  |
| RBL1 | RASGRF1 |  |  |
| RHOB | RB1 |  |  |
| RHOC | RHOJ |  |  |
| RHOV | RHOV |  |  |
| SRC |  |  |  |
| STK36 |  |  |  |
