# Supplementary material for: Genome-Wide Analyses of Nkx2-1 Binding to Transcriptional Target Genes Uncover Novel Regulatory Patterns Conserved in Lung Development and Tumors
Source: PLoS One. 2012 Jan 5;7(1):e29907. doi: 10.1371/journal.pone.0029907 (PMC3252372; doi:10.1371/journal.pone.0029907)
Supplement: Table S7 — PCR and qPCR Oligonucleotide sequences. (DOC) [file pone.0029907.s012.doc]

| *Table S7. PCR and qPCR Oligonucleotide sequences* | | |
| --- | --- | --- |
| ***Name*** | ***Left Primer*** | ***Right Primer*** |
| Ccnb1_Promoter | TTCCCAGCTCTGAGGACCTA | TGGCAAGACCATTGTGAGAA |
| Ccnb1_Exon1 | TTAAACCTAAGCCCGGCAG | TCCATAAGGTGGGTCCTCAG |
| Ccnb2_Promoter | CAGCAGACACTGCCCATCTA | ACTTGTGCGCTAGCCTGATA |
| Ccnb2_Exon1 | GGTGTCGCAGACCGGAG | CGCTTTAGCTCTGCCAGG |
| E2F3_Promoter | TTGGGAAAAATTTCAGGACTGT | CCAAGAGCAGTCAGAGGAAGA |
| E2F3_Exon1 | AGAGACTTGGAAACTCCGGC | AGGGACAGCAACGAGGC |
| Pols_Promoter | TGGTGTTCATCCCATGTTCA | GCCTACTGCTCTGTCCCTGT |
| Pols_Exon2 | ATCACTCCAGTCCCAGAAGC | CTCAAACTTCCAGCCTTTGC |
| Pik3ca_Promoter | TTGGAAAGACCGTTTTCAGG | CAACAAGCGACTTGTCCTCA |
| Pik3ca_Exon1 | TTTGTTCAGGGCACTGTTTG | GGAGGCATCATAGTTATTTGCAG |
| Hdac8_Promoter | AGGTACTGAGGGGCAGGATT | AGGCCTTTGTTCCCTACGTT |
| Hdac8_Exon1 | GGCTGAGTCTGAAAACTGTTGG | TACTCCCTTTCACACCCTCC |
| Slit1_Promoter | TGGGGATAAGGCCATGTTAG | CTGGGGTGATTGACCAAAAT |
| Slit1_Exon1 | GGACGGGGATGGTCAGC | GTCAGGGTGGGAGAGCAAC |
| Slit3_Promoter | GATTTGGGTCCCTTCCCTAA | TCCATGGGAAAGAGATGAGG |
| Slit3_Exon1 | CTCGGGCTCCTCGTGTC | CCTGAGGGCGACTCTGTAGG |
| Neurod6_Promoter | ACGCTTGAGGAGTGCTTTGT | ATCAGGAGCTTGGGGATTTT |
| Neurod6_Exon2_1 | TGATTGTTGGTCTGTGAAAACTC | GGTCTCTTGCCAATCCTCAG |
| Lhx6_Promoter | AAGTCTTCCCTCCACCCCTA | TCTCGGGTAATTTCCCTCCT |
| Lhx6_Exon1 | CCACTCTGCGCCTCTCTTC | GTTGAATCCCGGCTCCC |
| Olig3_Promoter | AAGAAGGAAACCCCTGTGCT | CTAAGAGGCTGGCCTGAAGA |
| Olig3_Exon1_1 | CAAGATTCTCTTTAATTTCCTGCC | CCCCGTAGATCTCTCCAACC |
| Met_Promoter | TTGCTTGGTGACTTTTGGTG | CGGGGTTGGGTATTGTTATG |
| Met_Exon3_1 | TTCTAATGACGACTAAAGGGCTC | TCAGCAGAATTGTCAGGAGG |
| Nkx2.1_Promoter | GCACACTCTTTTGGTGGTGA | GCAACCAACTTGGGGAGTTA |
|  |  |  |
| ***Taqman probes*** | ***Assay ID*** |  |
| Nkx2.1 | Mm00447558_m1 |  |
| E2f3 | Mm01138833_m1 |  |
| CyclinB1 | Mm00838401_g1 |  |
| CyclinB2 | Mm01171453_m1 |  |
| Pik3ca | Mm004335673_m1 |  |
| Met | Mm01156972_m1 |  |
| Gapdh | Mm99999915_g1 |  |
